# Supplementary material for: A Comprehensive Assessment of Ultraviolet-Radiation-Induced Mutations in Flammulina filiformis Using Whole-Genome Resequencing
Source: J Fungi (Basel). 2024 Mar 20;10(3):228. doi: 10.3390/jof10030228 (PMC10971301; doi:10.3390/jof10030228)

## Slide 1
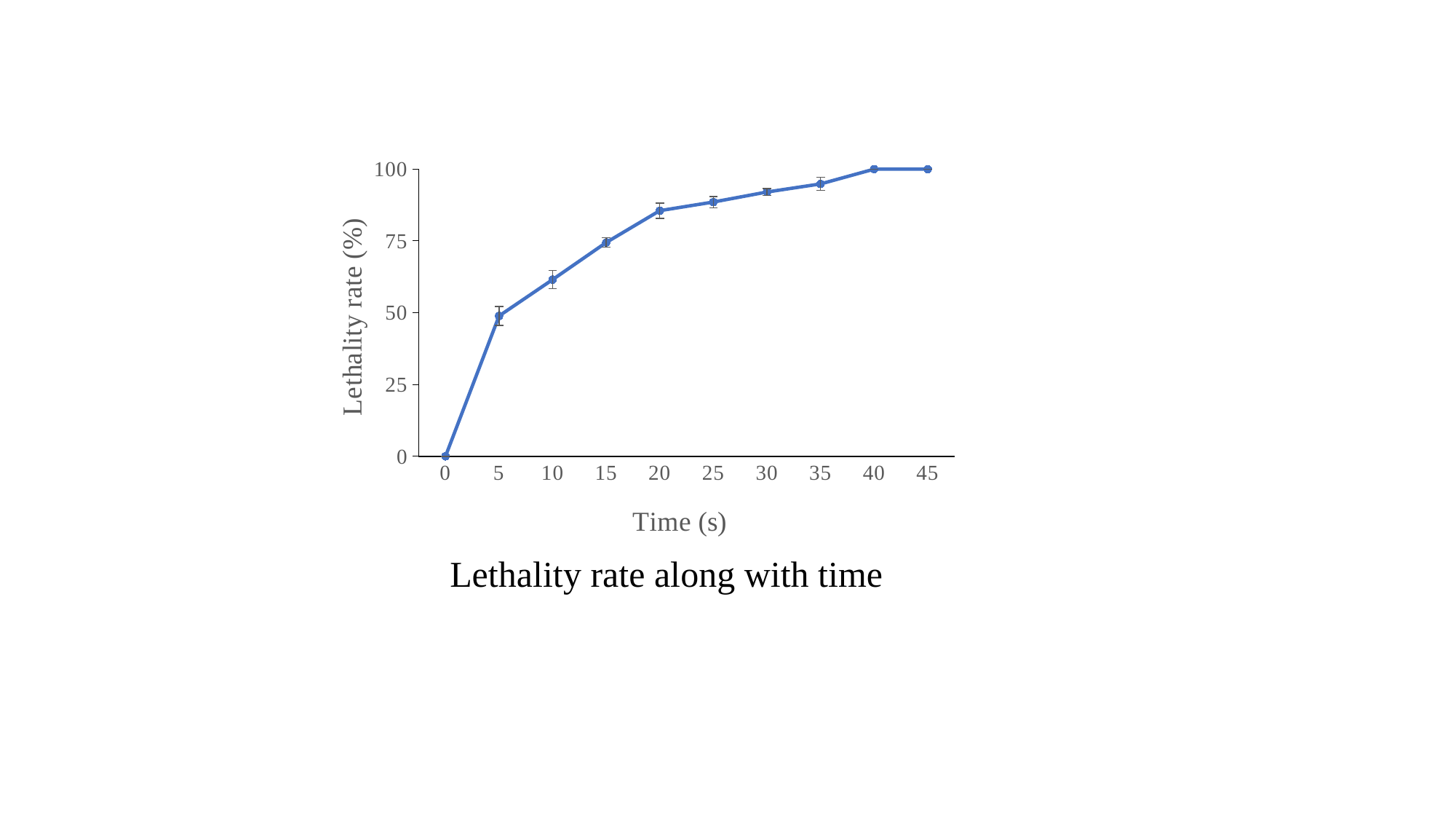

### Chart
| Category | |
|---|---|
| 0 | 0.0 |
| 5 | 48.86666666666667 |
| 10 | 61.53333333333333 |
| 15 | 74.46666666666665 |
| 20 | 85.53333333333335 |
| 25 | 88.53333333333335 |
| 30 | 92.03333333333332 |
| 35 | 94.83333333333333 |
| 40 | 100.0 |
| 45 | 100.0 |Lethality rate along with time

## Slide 2
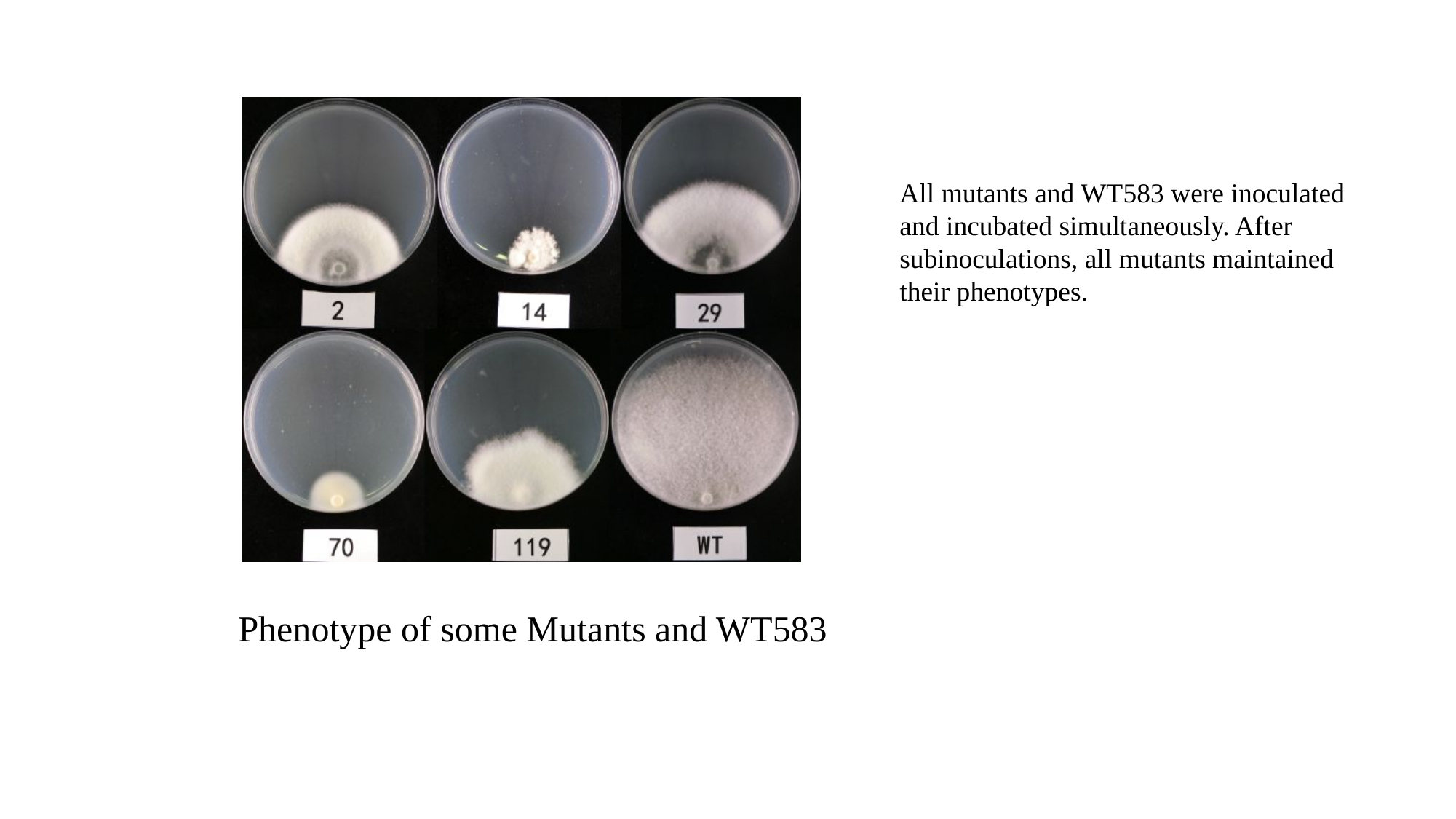

All mutants and WT583 were inoculated and incubated simultaneously. After subinoculations, all mutants maintained their phenotypes.
Phenotype of some Mutants and WT583

## Slide 3
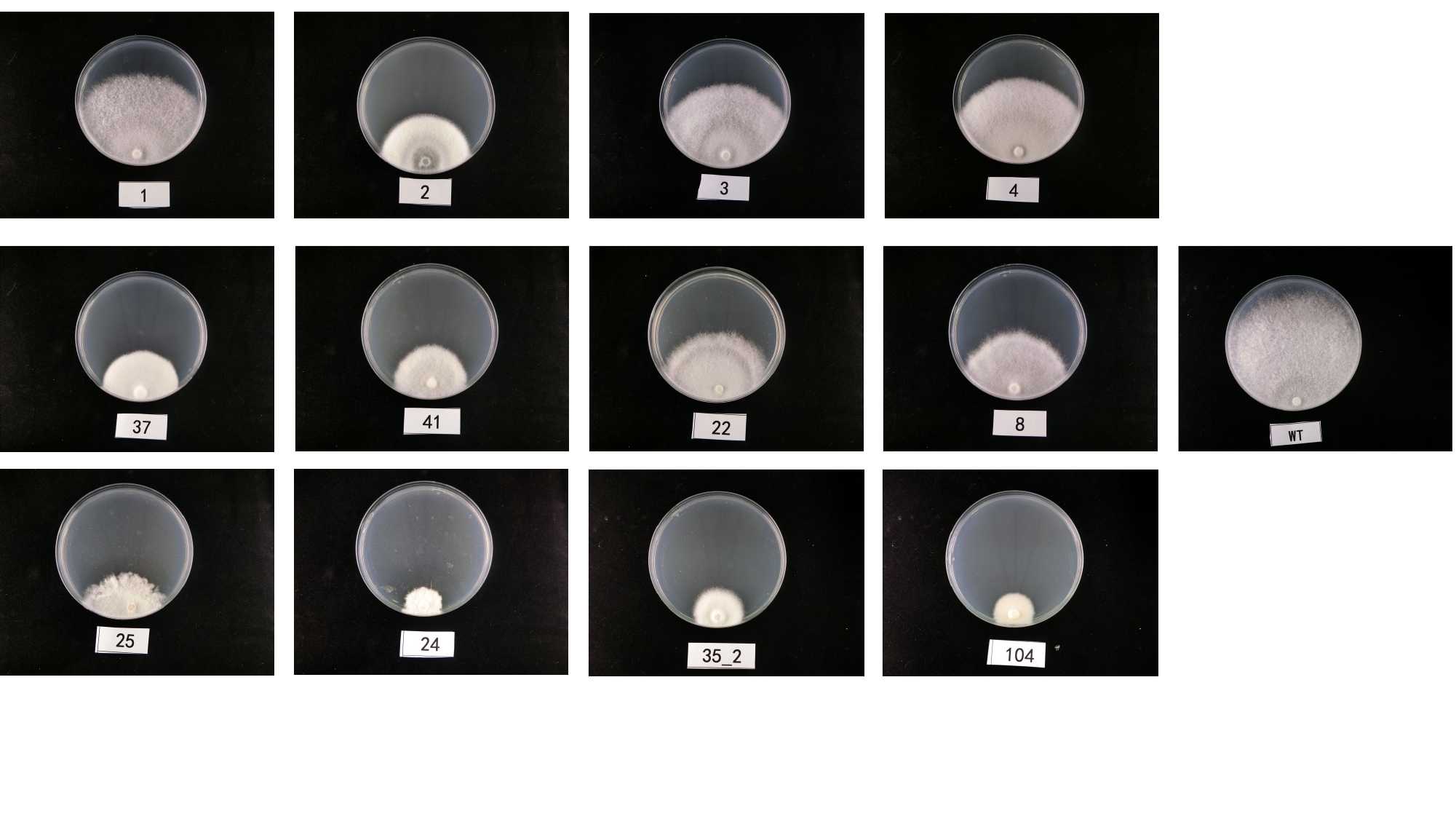

## Slide 4
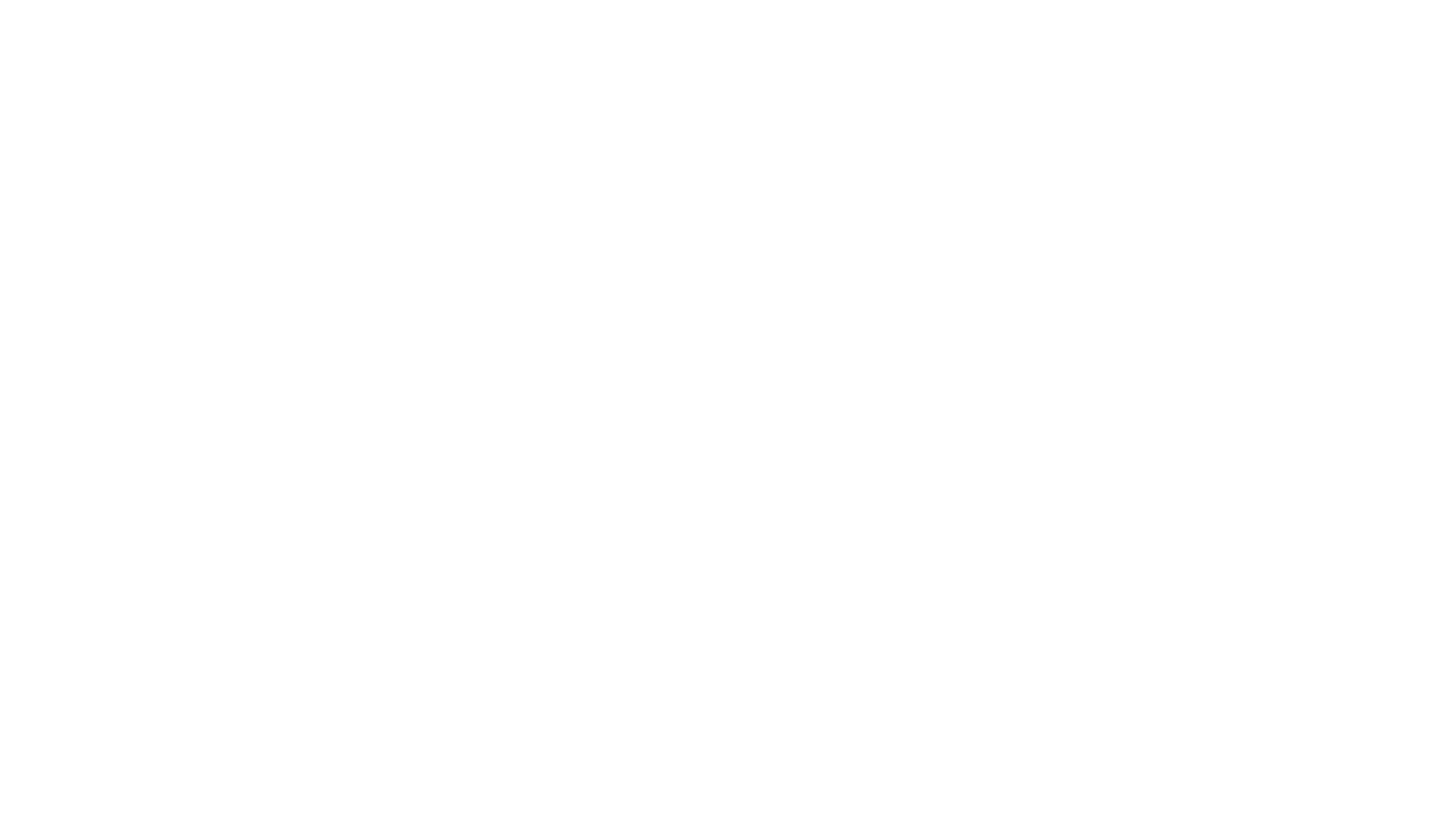

Supplement: Supplementary file 1 [file jof-10-00228-s001.zip › Supplementary Material S2.pptx]
