# Supplementary material for: A Comprehensive Assessment of Ultraviolet-Radiation-Induced Mutations in Flammulina filiformis Using Whole-Genome Resequencing
Source: J Fungi (Basel). 2024 Mar 20;10(3):228. doi: 10.3390/jof10030228 (PMC10971301; doi:10.3390/jof10030228)
Supplement: Supplementary file 1 [file jof-10-00228-s001.zip › Supplementary Material S3.pdf]

Genomic DNA extraction protocol for mushroom mycelia

Buffers used:

2×CTAB buffer

2% CTAB
100 mM Tris-HCl (pH = 8.0)
20 mM EDTA (pH = 8.0)
1.4 M NaCl
1% PVP (polyvinyl pyrrolidone)

Soil DNA extraction buffer

100 mM NaCl
50 mM EDTA (pH = 8.0)
0.25 M Tris-HCl (pH = 8.0)
5% SDS

DNA Extraction Protocol

Powder 0.1-0.5 g freeze dried or fresh mycelium

· Collect 0.5 g mushroom mycelia, and stored in liquid nitrogen until use. Grind all mycelia with mortar and pestle in liquid nitrogen until mycelial powders are fine enough. Fresh mycelia are also suitable for grinding. Add enough mycelial powders to precooled 1.5-ml tube before melting, and put the tubes in liquid nitrogen until all samples ground.

· Add 400 µl soil extraction buffer and 400 µl 2×CTAB buffer to 1.5-ml tube, vortex immediately until all powders are dissolved. All buffers should be added into the tube quickly to avoid that mycelial powders melt without adding buffers. Then, add 500 µl mixture (phenol: chlorophorm: isoamylalcohol = 25: 24: 1) in fumehood, and vortex for 5 min at room temperature.

· After vortex, centrifuge by 13 000 rpm for 5 min at 4℃. Collect 650 µl supernatant and transfer to a clean 1.5-ml tube (try not to take any phenol or interface material). Add 450 µl isopropanol to the collected supernatant and mix them thoroughly. Centrifuge by 13 000 rpm for 10 min at 4℃.

· Remove supernatant and wash the DNA pellet with 900 µl ethanol (make sure that the DNA pellet is detached from bottom of the tube). Centrifuge by 13 000 rpm for 5 min at 4℃, and discard ethanol.

· Make the DNA dry in the air or oven, add 50-100 µl TE buffer with Rnase, and incubate at 37℃ for 30 min. Finally, the DNA samples are stored at 4℃ prepared for use.
