# Supplementary material for: A Comprehensive Assessment of Ultraviolet-Radiation-Induced Mutations in Flammulina filiformis Using Whole-Genome Resequencing
Source: J Fungi (Basel). 2024 Mar 20;10(3):228. doi: 10.3390/jof10030228 (PMC10971301; doi:10.3390/jof10030228)
Supplement: Supplementary file 1 [file jof-10-00228-s001.zip › Supplementary Material S5.pdf]

Table Effects of flanking bases on mutation frequency of adenine

Motif	Base substitution			Total
	A→G	A→T	A→C	
AAA	63	13	11	87
TAA	178	72	66	316
CAA	63	14	7	84
GAA	54	11	2	67
AAT	49	102	14	165
TAT	32	62	10	104
CAT	15	18	3	36
GAT	14	16	4	34
AAC	6	16	4	26
TAC	3	18	2	23
CAC	3	5	2	10
GAC	6	4	0	10
AAG	57	18	3	78
TAG	71	14	5	90
CAG	21	4	1	26
GAG	16	7	1	24
Total	651	394	135	1 180
Mutation frequency	55.17%	33.39%	11.44%	100%

Table Effects of flanking bases on mutation frequency of cytosine

Motif	Base substitution			Total
	C→T	C→A	C→G	
ACA	39	13	3	55
TCA	226	24	8	258
CCA	149	33	5	187
GCA	17	3	6	26
ACT	141	8	3	152
TCT	352	20	6	378
CCT	211	16	4	231
GCT	67	6	0	73
ACC	106	18	2	126
TCC	348	31	4	383
CCC	175	8	5	188
GCC	55	8	2	65
ACG	32	8	7	47
TCG	216	14	5	235
CCG	92	9	5	106
GCG	13	2	2	17
Total	2 239	221	67	2 527
Mutation frequency	88.60%	8.75%	2.65%	100%

Table Total number of motifs in *Flammulina filiformis*

Base at 5′ side of adenine				
motif	AA	TA	CA	GA
number	3 897 395	4 277 842	4 800 411	5 153 977
Base at 3′ side of adenine				
motif	AA	AT	AC	AG
number	3 897 395	4 287 842	4 803 034	5 153 631
Base at 5′ side of cytosine				
motif	AC	TC	CC	GC
number	4 803 034	4 716 410	3 605 656	4 571 199
Base at 3′ side of cytosine				
motif	CA	CT	CC	CG
number	4 800 411	4 976 392	3 605 656	4 573 199
