# Supplementary material for: A Comprehensive Assessment of Ultraviolet-Radiation-Induced Mutations in Flammulina filiformis Using Whole-Genome Resequencing
Source: J Fungi (Basel). 2024 Mar 20;10(3):228. doi: 10.3390/jof10030228 (PMC10971301; doi:10.3390/jof10030228)
Supplement: Supplementary file 1 [file jof-10-00228-s001.zip › Supplementary Material S6.pdf]

Table Components for detection and gradient temperature PCR

Components	Volume (μL)
2×rTaq Mix	12.5
ddH_2O	9.5
Primer1	1
Primer2	1
Template	1

Table PCR components for product sequencing

Components	Volume (μL)
2×rTaq Mix	25
ddH2O	19
Primer1	2
Primer2	2
Template	2

Table Deleted DNA fragments and their detection primers

Amplified fragments	Length (bp)	Tm (℃)	Extension time (s)	Primers (5`→3`)
84-1	1430	55	95	F: CAAGACTTACGGATGGTGGA
				R: AGATTTCTGCTGACGAGGGA
42-3	517	51.3	54	F: TTGTGATGATTATTGGGCAGTT
				R: CTATGTTGAGCCGTCGAACC

Table PCR program for 84-1 fragment

94℃	34 cycles			72℃
	94℃	55℃	72℃	
300 s	30 s	30 s	90 s	600 s

Table PCR program for 42-3 fragment

94℃	34 cycles			72℃
	94℃	52℃	72℃	
300 s	30 s	30 s	35 s	600 s
