# Supplementary material for: A Comprehensive Assessment of Ultraviolet-Radiation-Induced Mutations in Flammulina filiformis Using Whole-Genome Resequencing
Source: J Fungi (Basel). 2024 Mar 20;10(3):228. doi: 10.3390/jof10030228 (PMC10971301; doi:10.3390/jof10030228)
Supplement: Supplementary file 1 [file jof-10-00228-s001.zip › Supplementary Material S7.pdf]

Table Mutational spectra of different base combinations

Before mutation	After mutation	NO.	Total
AA	TT	1	10
	TG	4	
	CG	1	
	GG	4	
AT	TC	1	7
	CA	1	
	CG	1	
	GA	1	
	GC	3	
AC	TT	3	11
	CT	1	
	CG	1	
	GT	6	
AG	TA	6	11
	GA	5	
TA	AT	1	11
	AC	1	
	AG	4	
	CC	1	
	CG	4	
TT	AC	1	5
	CA	2	
	CC	1	
	CG	1	
TC	AT	4	12
	AG	1	
	CT	6	
	CG	1	
TG	AA	4	12
	CA	8	
CA	AG	2	14
	TT	4	
	TC	1	
	TG	7	
CT	TA	2	8
	TC	4	
	TG	2	
CC	AT	2	33
	TA	2	
	TT	28	

	GT	1	
CG	AA	1	17
	TA	14	
	TT	1	
	GT	1	
GA	AT	3	12
	AC	1	
	AG	7	
	TG	1	
GT	AA	7	10
	AC	3	
GC	AA	1	28
	AT	26	
	TT	1	
GG	AA	20	27
	AT	1	
	TA	6	

Table Distribution of tandem mutations in mutants

Times of mutation	Numbers of mutants	Mutants
0	24	R02-1, R09, R02-2, R12, R22, R23, R33, R02-4, R49, R05-7, R05-11, R05-14, R03-1, R05-15, R03-2, R04-16, R02-12, R04-17, R04-19, R03-5, R03-7, R04-2, R03-9, R03-23
1	21	R24, R25, R29, R37, R02-22, R05-1, R05-5, R05-9, 05-13, R02-8, R04-15, R03-4, R04-24, R02-14, R02-24, R04-4, R03-11, R03-12, R06-20, R03-21, R06-22
2	14	R08, R14, R26, R35, R41, R42, R05-3, R02-6, R02-13, R03-3, R04-20, R04-1, R02-15, R02-19
3	17	R02, R13, R15, R16, R02-17, R02-18, R31, R02-21, R04-13, R04-14, R04-18, R06-17, R04-23, R04-3, R02-10, R02-23, R06-23
4	13	R05, R02-5, R05-4, R05-6, R02-7, R06-6, R04-21, R04-22, R02-11, R03-10, R02-3, R02-9, R06-24, R05-2, R06-18, R02-16
5	3	R05-2, R06-18, R02-16
6	4	R47, R48, R05-10, R03-8

7	1	R02-20
8	1	R03-24
9	1	R06-19
13	1	R05-12
