# Supplementary material for: A Comprehensive Assessment of Ultraviolet-Radiation-Induced Mutations in Flammulina filiformis Using Whole-Genome Resequencing
Source: J Fungi (Basel). 2024 Mar 20;10(3):228. doi: 10.3390/jof10030228 (PMC10971301; doi:10.3390/jof10030228)
Supplement: Supplementary file 1 [file jof-10-00228-s001.zip › Supplementary Material S8/GO annotation/out/out.C.html]

 


GO Enrichment Analysis


out GO Enrichment (Cellular Component)

| # | GO ID | Description | GeneRatio (334) | BgRatio (334) | pvalue | fdr |
| 1 | GO:0016020 | membrane | 216 | 216 | 1.000000 | 1.000000 |
| 2 | GO:0005623 | cell | 160 | 160 | 1.000000 | 1.000000 |
| 3 | GO:0044464 | cell part | 157 | 157 | 1.000000 | 1.000000 |
| 4 | GO:0044425 | membrane part | 150 | 150 | 1.000000 | 1.000000 |
| 5 | GO:0005622 | intracellular | 147 | 147 | 1.000000 | 1.000000 |
| 6 | GO:0044424 | intracellular part | 147 | 147 | 1.000000 | 1.000000 |
| 7 | GO:0016021 | integral component of membrane | 145 | 145 | 1.000000 | 1.000000 |
| 8 | GO:0031224 | intrinsic component of membrane | 145 | 145 | 1.000000 | 1.000000 |
| 9 | GO:0043226 | organelle | 119 | 119 | 1.000000 | 1.000000 |
| 10 | GO:0043229 | intracellular organelle | 119 | 119 | 1.000000 | 1.000000 |
| 11 | GO:0043227 | membrane-bounded organelle | 97 | 97 | 1.000000 | 1.000000 |
| 12 | GO:0043231 | intracellular membrane-bounded organelle | 97 | 97 | 1.000000 | 1.000000 |
| 13 | GO:0005737 | cytoplasm | 73 | 73 | 1.000000 | 1.000000 |
| 14 | GO:0032991 | macromolecular complex | 64 | 64 | 1.000000 | 1.000000 |
| 15 | GO:0005634 | nucleus | 62 | 62 | 1.000000 | 1.000000 |
| 16 | GO:0044444 | cytoplasmic part | 58 | 58 | 1.000000 | 1.000000 |
| 17 | GO:0044422 | organelle part | 56 | 56 | 1.000000 | 1.000000 |
| 18 | GO:0044446 | intracellular organelle part | 56 | 56 | 1.000000 | 1.000000 |
| 19 | GO:0043234 | protein complex | 45 | 45 | 1.000000 | 1.000000 |
| 20 | GO:0043228 | non-membrane-bounded organelle | 35 | 35 | 1.000000 | 1.000000 |
| 21 | GO:0043232 | intracellular non-membrane-bounded organelle | 35 | 35 | 1.000000 | 1.000000 |
| 22 | GO:0044428 | nuclear part | 27 | 27 | 1.000000 | 1.000000 |
| 23 | GO:0030529 | intracellular ribonucleoprotein complex | 22 | 22 | 1.000000 | 1.000000 |
| 24 | GO:0031974 | membrane-enclosed lumen | 22 | 22 | 1.000000 | 1.000000 |
| 25 | GO:0043233 | organelle lumen | 22 | 22 | 1.000000 | 1.000000 |
| 26 | GO:0070013 | intracellular organelle lumen | 22 | 22 | 1.000000 | 1.000000 |
| 27 | GO:1990904 | ribonucleoprotein complex | 22 | 22 | 1.000000 | 1.000000 |
| 28 | GO:0031981 | nuclear lumen | 18 | 18 | 1.000000 | 1.000000 |
| 29 | GO:0005739 | mitochondrion | 16 | 16 | 1.000000 | 1.000000 |
| 30 | GO:0012505 | endomembrane system | 16 | 16 | 1.000000 | 1.000000 |
| 31 | GO:0071944 | cell periphery | 16 | 16 | 1.000000 | 1.000000 |
| 32 | GO:0031090 | organelle membrane | 15 | 15 | 1.000000 | 1.000000 |
| 33 | GO:1902494 | catalytic complex | 15 | 15 | 1.000000 | 1.000000 |
| 34 | GO:0005694 | chromosome | 11 | 11 | 1.000000 | 1.000000 |
| 35 | GO:0098588 | bounding membrane of organelle | 11 | 11 | 1.000000 | 1.000000 |
| 36 | GO:0005840 | ribosome | 10 | 10 | 1.000000 | 1.000000 |
| 37 | GO:1990234 | transferase complex | 10 | 10 | 1.000000 | 1.000000 |
| 38 | GO:0005856 | cytoskeleton | 9 | 9 | 1.000000 | 1.000000 |
| 39 | GO:0044427 | chromosomal part | 9 | 9 | 1.000000 | 1.000000 |
| 40 | GO:0044429 | mitochondrial part | 8 | 8 | 1.000000 | 1.000000 |
| 41 | GO:0098805 | whole membrane | 8 | 8 | 1.000000 | 1.000000 |
| 42 | GO:0000228 | nuclear chromosome | 7 | 7 | 1.000000 | 1.000000 |
| 43 | GO:0005654 | nucleoplasm | 7 | 7 | 1.000000 | 1.000000 |
| 44 | GO:0031967 | organelle envelope | 7 | 7 | 1.000000 | 1.000000 |
| 45 | GO:0031975 | envelope | 7 | 7 | 1.000000 | 1.000000 |
| 46 | GO:0044430 | cytoskeletal part | 7 | 7 | 1.000000 | 1.000000 |
| 47 | GO:0044451 | nucleoplasm part | 7 | 7 | 1.000000 | 1.000000 |
| 48 | GO:0005794 | Golgi apparatus | 6 | 6 | 1.000000 | 1.000000 |
| 49 | GO:0005886 | plasma membrane | 6 | 6 | 1.000000 | 1.000000 |
| 50 | GO:0044454 | nuclear chromosome part | 6 | 6 | 1.000000 | 1.000000 |
| 51 | GO:0005618 | cell wall | 5 | 5 | 1.000000 | 1.000000 |
| 52 | GO:0005773 | vacuole | 5 | 5 | 1.000000 | 1.000000 |
| 53 | GO:0005783 | endoplasmic reticulum | 5 | 5 | 1.000000 | 1.000000 |
| 54 | GO:0005829 | cytosol | 5 | 5 | 1.000000 | 1.000000 |
| 55 | GO:0005938 | cell cortex | 5 | 5 | 1.000000 | 1.000000 |
| 56 | GO:0030312 | external encapsulating structure | 5 | 5 | 1.000000 | 1.000000 |
| 57 | GO:0044448 | cell cortex part | 5 | 5 | 1.000000 | 1.000000 |
| 58 | GO:0061695 | transferase complex, transferring phosphorus-containing groups | 5 | 5 | 1.000000 | 1.000000 |
| 59 | GO:0099568 | cytoplasmic region | 5 | 5 | 1.000000 | 1.000000 |
| 60 | GO:0000428 | DNA-directed RNA polymerase complex | 4 | 4 | 1.000000 | 1.000000 |
| 61 | GO:0000775 | chromosome, centromeric region | 4 | 4 | 1.000000 | 1.000000 |
| 62 | GO:0000785 | chromatin | 4 | 4 | 1.000000 | 1.000000 |
| 63 | GO:0005667 | transcription factor complex | 4 | 4 | 1.000000 | 1.000000 |
| 64 | GO:0005740 | mitochondrial envelope | 4 | 4 | 1.000000 | 1.000000 |
| 65 | GO:0005759 | mitochondrial matrix | 4 | 4 | 1.000000 | 1.000000 |
| 66 | GO:0005774 | vacuolar membrane | 4 | 4 | 1.000000 | 1.000000 |
| 67 | GO:0015630 | microtubule cytoskeleton | 4 | 4 | 1.000000 | 1.000000 |
| 68 | GO:0016591 | DNA-directed RNA polymerase II, holoenzyme | 4 | 4 | 1.000000 | 1.000000 |
| 69 | GO:0030880 | RNA polymerase complex | 4 | 4 | 1.000000 | 1.000000 |
| 70 | GO:0031966 | mitochondrial membrane | 4 | 4 | 1.000000 | 1.000000 |
| 71 | GO:0032993 | protein-DNA complex | 4 | 4 | 1.000000 | 1.000000 |
| 72 | GO:0035770 | ribonucleoprotein granule | 4 | 4 | 1.000000 | 1.000000 |
| 73 | GO:0036464 | cytoplasmic ribonucleoprotein granule | 4 | 4 | 1.000000 | 1.000000 |
| 74 | GO:0044431 | Golgi apparatus part | 4 | 4 | 1.000000 | 1.000000 |
| 75 | GO:0044437 | vacuolar part | 4 | 4 | 1.000000 | 1.000000 |
| 76 | GO:0044459 | plasma membrane part | 4 | 4 | 1.000000 | 1.000000 |
| 77 | GO:0044798 | nuclear transcription factor complex | 4 | 4 | 1.000000 | 1.000000 |
| 78 | GO:0055029 | nuclear DNA-directed RNA polymerase complex | 4 | 4 | 1.000000 | 1.000000 |
| 79 | GO:0090575 | RNA polymerase II transcription factor complex | 4 | 4 | 1.000000 | 1.000000 |
| 80 | GO:0098687 | chromosomal region | 4 | 4 | 1.000000 | 1.000000 |
| 81 | GO:0098796 | membrane protein complex | 4 | 4 | 1.000000 | 1.000000 |
| 82 | GO:0000145 | exocyst | 3 | 3 | 1.000000 | 1.000000 |
| 83 | GO:0000322 | storage vacuole | 3 | 3 | 1.000000 | 1.000000 |
| 84 | GO:0000323 | lytic vacuole | 3 | 3 | 1.000000 | 1.000000 |
| 85 | GO:0000324 | fungal-type vacuole | 3 | 3 | 1.000000 | 1.000000 |
| 86 | GO:0000790 | nuclear chromatin | 3 | 3 | 1.000000 | 1.000000 |
| 87 | GO:0000793 | condensed chromosome | 3 | 3 | 1.000000 | 1.000000 |
| 88 | GO:0005635 | nuclear envelope | 3 | 3 | 1.000000 | 1.000000 |
| 89 | GO:0005730 | nucleolus | 3 | 3 | 1.000000 | 1.000000 |
| 90 | GO:0005743 | mitochondrial inner membrane | 3 | 3 | 1.000000 | 1.000000 |
| 91 | GO:0005768 | endosome | 3 | 3 | 1.000000 | 1.000000 |
| 92 | GO:0010494 | cytoplasmic stress granule | 3 | 3 | 1.000000 | 1.000000 |
| 93 | GO:0015629 | actin cytoskeleton | 3 | 3 | 1.000000 | 1.000000 |
| 94 | GO:0019866 | organelle inner membrane | 3 | 3 | 1.000000 | 1.000000 |
| 95 | GO:0044432 | endoplasmic reticulum part | 3 | 3 | 1.000000 | 1.000000 |
| 96 | GO:0000109 | nucleotide-excision repair complex | 2 | 2 | 1.000000 | 1.000000 |
| 97 | GO:0000139 | Golgi membrane | 2 | 2 | 1.000000 | 1.000000 |
| 98 | GO:0000178 | exosome (RNase complex) | 2 | 2 | 1.000000 | 1.000000 |
| 99 | GO:0000329 | fungal-type vacuole membrane | 2 | 2 | 1.000000 | 1.000000 |
| 100 | GO:0000794 | condensed nuclear chromosome | 2 | 2 | 1.000000 | 1.000000 |
| 101 | GO:0005576 | extracellular region | 2 | 2 | 1.000000 | 1.000000 |
| 102 | GO:0005643 | nuclear pore | 2 | 2 | 1.000000 | 1.000000 |
| 103 | GO:0005657 | replication fork | 2 | 2 | 1.000000 | 1.000000 |
| 104 | GO:0005681 | spliceosomal complex | 2 | 2 | 1.000000 | 1.000000 |
| 105 | GO:0005684 | U2-type spliceosomal complex | 2 | 2 | 1.000000 | 1.000000 |
| 106 | GO:0005789 | endoplasmic reticulum membrane | 2 | 2 | 1.000000 | 1.000000 |
| 107 | GO:0005798 | Golgi-associated vesicle | 2 | 2 | 1.000000 | 1.000000 |
| 108 | GO:0005819 | spindle | 2 | 2 | 1.000000 | 1.000000 |
| 109 | GO:0005852 | eukaryotic translation initiation factor 3 complex | 2 | 2 | 1.000000 | 1.000000 |
| 110 | GO:0005874 | microtubule | 2 | 2 | 1.000000 | 1.000000 |
| 111 | GO:0005887 | integral component of plasma membrane | 2 | 2 | 1.000000 | 1.000000 |
| 112 | GO:0009277 | fungal-type cell wall | 2 | 2 | 1.000000 | 1.000000 |
| 113 | GO:0009337 | sulfite reductase complex (NADPH) | 2 | 2 | 1.000000 | 1.000000 |
| 114 | GO:0016023 | cytoplasmic, membrane-bounded vesicle | 2 | 2 | 1.000000 | 1.000000 |
| 115 | GO:0016282 | eukaryotic 43S preinitiation complex | 2 | 2 | 1.000000 | 1.000000 |
| 116 | GO:0016459 | myosin complex | 2 | 2 | 1.000000 | 1.000000 |
| 117 | GO:0030135 | coated vesicle | 2 | 2 | 1.000000 | 1.000000 |
| 118 | GO:0030479 | actin cortical patch | 2 | 2 | 1.000000 | 1.000000 |
| 119 | GO:0030532 | small nuclear ribonucleoprotein complex | 2 | 2 | 1.000000 | 1.000000 |
| 120 | GO:0030684 | preribosome | 2 | 2 | 1.000000 | 1.000000 |
| 121 | GO:0030863 | cortical cytoskeleton | 2 | 2 | 1.000000 | 1.000000 |
| 122 | GO:0030864 | cortical actin cytoskeleton | 2 | 2 | 1.000000 | 1.000000 |
| 123 | GO:0031226 | intrinsic component of plasma membrane | 2 | 2 | 1.000000 | 1.000000 |
| 124 | GO:0031300 | intrinsic component of organelle membrane | 2 | 2 | 1.000000 | 1.000000 |
| 125 | GO:0031410 | cytoplasmic vesicle | 2 | 2 | 1.000000 | 1.000000 |
| 126 | GO:0031982 | vesicle | 2 | 2 | 1.000000 | 1.000000 |
| 127 | GO:0031988 | membrane-bounded vesicle | 2 | 2 | 1.000000 | 1.000000 |
| 128 | GO:0033290 | eukaryotic 48S preinitiation complex | 2 | 2 | 1.000000 | 1.000000 |
| 129 | GO:0034708 | methyltransferase complex | 2 | 2 | 1.000000 | 1.000000 |
| 130 | GO:0035097 | histone methyltransferase complex | 2 | 2 | 1.000000 | 1.000000 |
| 131 | GO:0038201 | TOR complex | 2 | 2 | 1.000000 | 1.000000 |
| 132 | GO:0042175 | nuclear outer membrane-endoplasmic reticulum membrane network | 2 | 2 | 1.000000 | 1.000000 |
| 133 | GO:0043596 | nuclear replication fork | 2 | 2 | 1.000000 | 1.000000 |
| 134 | GO:0044445 | cytosolic part | 2 | 2 | 1.000000 | 1.000000 |
| 135 | GO:0044815 | DNA packaging complex | 2 | 2 | 1.000000 | 1.000000 |
| 136 | GO:0061645 | endocytic patch | 2 | 2 | 1.000000 | 1.000000 |
| 137 | GO:0070993 | translation preinitiation complex | 2 | 2 | 1.000000 | 1.000000 |
| 138 | GO:0097525 | spliceosomal snRNP complex | 2 | 2 | 1.000000 | 1.000000 |
| 139 | GO:0097550 | transcriptional preinitiation complex | 2 | 2 | 1.000000 | 1.000000 |
| 140 | GO:0098852 | lytic vacuole membrane | 2 | 2 | 1.000000 | 1.000000 |
| 141 | GO:0099512 | supramolecular fiber | 2 | 2 | 1.000000 | 1.000000 |
| 142 | GO:0099513 | polymeric cytoskeletal fiber | 2 | 2 | 1.000000 | 1.000000 |
| 143 | GO:1990391 | DNA repair complex | 2 | 2 | 1.000000 | 1.000000 |
| 144 | GO:0000112 | nucleotide-excision repair factor 3 complex | 1 | 1 | 1.000000 | 1.000000 |
| 145 | GO:0000151 | ubiquitin ligase complex | 1 | 1 | 1.000000 | 1.000000 |
| 146 | GO:0000313 | organellar ribosome | 1 | 1 | 1.000000 | 1.000000 |
| 147 | GO:0000439 | core TFIIH complex | 1 | 1 | 1.000000 | 1.000000 |
| 148 | GO:0000502 | proteasome complex | 1 | 1 | 1.000000 | 1.000000 |
| 149 | GO:0000776 | kinetochore | 1 | 1 | 1.000000 | 1.000000 |
| 150 | GO:0000777 | condensed chromosome kinetochore | 1 | 1 | 1.000000 | 1.000000 |
| 151 | GO:0000778 | condensed nuclear chromosome kinetochore | 1 | 1 | 1.000000 | 1.000000 |
| 152 | GO:0000779 | condensed chromosome, centromeric region | 1 | 1 | 1.000000 | 1.000000 |
| 153 | GO:0000780 | condensed nuclear chromosome, centromeric region | 1 | 1 | 1.000000 | 1.000000 |
| 154 | GO:0000786 | nucleosome | 1 | 1 | 1.000000 | 1.000000 |
| 155 | GO:0000792 | heterochromatin | 1 | 1 | 1.000000 | 1.000000 |
| 156 | GO:0000795 | synaptonemal complex | 1 | 1 | 1.000000 | 1.000000 |
| 157 | GO:0000796 | condensin complex | 1 | 1 | 1.000000 | 1.000000 |
| 158 | GO:0000922 | spindle pole | 1 | 1 | 1.000000 | 1.000000 |
| 159 | GO:0000932 | cytoplasmic mRNA processing body | 1 | 1 | 1.000000 | 1.000000 |
| 160 | GO:0000940 | condensed chromosome outer kinetochore | 1 | 1 | 1.000000 | 1.000000 |
| 161 | GO:0000942 | condensed nuclear chromosome outer kinetochore | 1 | 1 | 1.000000 | 1.000000 |
| 162 | GO:0000943 | retrotransposon nucleocapsid | 1 | 1 | 1.000000 | 1.000000 |
| 163 | GO:0001405 | presequence translocase-associated import motor | 1 | 1 | 1.000000 | 1.000000 |
| 164 | GO:0005665 | DNA-directed RNA polymerase II, core complex | 1 | 1 | 1.000000 | 1.000000 |
| 165 | GO:0005669 | transcription factor TFIID complex | 1 | 1 | 1.000000 | 1.000000 |
| 166 | GO:0005675 | holo TFIIH complex | 1 | 1 | 1.000000 | 1.000000 |
| 167 | GO:0005686 | U2 snRNP | 1 | 1 | 1.000000 | 1.000000 |
| 168 | GO:0005720 | nuclear heterochromatin | 1 | 1 | 1.000000 | 1.000000 |
| 169 | GO:0005721 | pericentric heterochromatin | 1 | 1 | 1.000000 | 1.000000 |
| 170 | GO:0005741 | mitochondrial outer membrane | 1 | 1 | 1.000000 | 1.000000 |
| 171 | GO:0005744 | mitochondrial inner membrane presequence translocase complex | 1 | 1 | 1.000000 | 1.000000 |
| 172 | GO:0005761 | mitochondrial ribosome | 1 | 1 | 1.000000 | 1.000000 |
| 173 | GO:0005770 | late endosome | 1 | 1 | 1.000000 | 1.000000 |
| 174 | GO:0005801 | cis-Golgi network | 1 | 1 | 1.000000 | 1.000000 |
| 175 | GO:0005802 | trans-Golgi network | 1 | 1 | 1.000000 | 1.000000 |
| 176 | GO:0005811 | lipid particle | 1 | 1 | 1.000000 | 1.000000 |
| 177 | GO:0005834 | heterotrimeric G-protein complex | 1 | 1 | 1.000000 | 1.000000 |
| 178 | GO:0005835 | fatty acid synthase complex | 1 | 1 | 1.000000 | 1.000000 |
| 179 | GO:0005851 | eukaryotic translation initiation factor 2B complex | 1 | 1 | 1.000000 | 1.000000 |
| 180 | GO:0005875 | microtubule associated complex | 1 | 1 | 1.000000 | 1.000000 |
| 181 | GO:0005885 | Arp2/3 protein complex | 1 | 1 | 1.000000 | 1.000000 |
| 182 | GO:0005956 | protein kinase CK2 complex | 1 | 1 | 1.000000 | 1.000000 |
| 183 | GO:0008023 | transcription elongation factor complex | 1 | 1 | 1.000000 | 1.000000 |
| 184 | GO:0008622 | epsilon DNA polymerase complex | 1 | 1 | 1.000000 | 1.000000 |
| 185 | GO:0009331 | glycerol-3-phosphate dehydrogenase complex | 1 | 1 | 1.000000 | 1.000000 |
| 186 | GO:0009898 | cytoplasmic side of plasma membrane | 1 | 1 | 1.000000 | 1.000000 |
| 187 | GO:0010008 | endosome membrane | 1 | 1 | 1.000000 | 1.000000 |
| 188 | GO:0012506 | vesicle membrane | 1 | 1 | 1.000000 | 1.000000 |
| 189 | GO:0012510 | trans-Golgi network transport vesicle membrane | 1 | 1 | 1.000000 | 1.000000 |
| 190 | GO:0016442 | RISC complex | 1 | 1 | 1.000000 | 1.000000 |
| 191 | GO:0016469 | proton-transporting two-sector ATPase complex | 1 | 1 | 1.000000 | 1.000000 |
| 192 | GO:0016593 | Cdc73/Paf1 complex | 1 | 1 | 1.000000 | 1.000000 |
| 193 | GO:0019005 | SCF ubiquitin ligase complex | 1 | 1 | 1.000000 | 1.000000 |
| 194 | GO:0019867 | outer membrane | 1 | 1 | 1.000000 | 1.000000 |
| 195 | GO:0019897 | extrinsic component of plasma membrane | 1 | 1 | 1.000000 | 1.000000 |
| 196 | GO:0019898 | extrinsic component of membrane | 1 | 1 | 1.000000 | 1.000000 |
| 197 | GO:0030008 | TRAPP complex | 1 | 1 | 1.000000 | 1.000000 |
| 198 | GO:0030117 | membrane coat | 1 | 1 | 1.000000 | 1.000000 |
| 199 | GO:0030118 | clathrin coat | 1 | 1 | 1.000000 | 1.000000 |
| 200 | GO:0030119 | AP-type membrane coat adaptor complex | 1 | 1 | 1.000000 | 1.000000 |
| 201 | GO:0030120 | vesicle coat | 1 | 1 | 1.000000 | 1.000000 |
| 202 | GO:0030121 | AP-1 adaptor complex | 1 | 1 | 1.000000 | 1.000000 |
| 203 | GO:0030125 | clathrin vesicle coat | 1 | 1 | 1.000000 | 1.000000 |
| 204 | GO:0030130 | clathrin coat of trans-Golgi network vesicle | 1 | 1 | 1.000000 | 1.000000 |
| 205 | GO:0030131 | clathrin adaptor complex | 1 | 1 | 1.000000 | 1.000000 |
| 206 | GO:0030133 | transport vesicle | 1 | 1 | 1.000000 | 1.000000 |
| 207 | GO:0030136 | clathrin-coated vesicle | 1 | 1 | 1.000000 | 1.000000 |
| 208 | GO:0030137 | COPI-coated vesicle | 1 | 1 | 1.000000 | 1.000000 |
| 209 | GO:0030140 | trans-Golgi network transport vesicle | 1 | 1 | 1.000000 | 1.000000 |
| 210 | GO:0030176 | integral component of endoplasmic reticulum membrane | 1 | 1 | 1.000000 | 1.000000 |
| 211 | GO:0030658 | transport vesicle membrane | 1 | 1 | 1.000000 | 1.000000 |
| 212 | GO:0030659 | cytoplasmic vesicle membrane | 1 | 1 | 1.000000 | 1.000000 |
| 213 | GO:0030660 | Golgi-associated vesicle membrane | 1 | 1 | 1.000000 | 1.000000 |
| 214 | GO:0030662 | coated vesicle membrane | 1 | 1 | 1.000000 | 1.000000 |
| 215 | GO:0030665 | clathrin-coated vesicle membrane | 1 | 1 | 1.000000 | 1.000000 |
| 216 | GO:0030687 | preribosome, large subunit precursor | 1 | 1 | 1.000000 | 1.000000 |
| 217 | GO:0030870 | Mre11 complex | 1 | 1 | 1.000000 | 1.000000 |
| 218 | GO:0030894 | replisome | 1 | 1 | 1.000000 | 1.000000 |
| 219 | GO:0030907 | MBF transcription complex | 1 | 1 | 1.000000 | 1.000000 |
| 220 | GO:0031011 | Ino80 complex | 1 | 1 | 1.000000 | 1.000000 |
| 221 | GO:0031227 | intrinsic component of endoplasmic reticulum membrane | 1 | 1 | 1.000000 | 1.000000 |
| 222 | GO:0031234 | extrinsic component of cytoplasmic side of plasma membrane | 1 | 1 | 1.000000 | 1.000000 |
| 223 | GO:0031298 | replication fork protection complex | 1 | 1 | 1.000000 | 1.000000 |
| 224 | GO:0031301 | integral component of organelle membrane | 1 | 1 | 1.000000 | 1.000000 |
| 225 | GO:0031310 | intrinsic component of vacuolar membrane | 1 | 1 | 1.000000 | 1.000000 |
| 226 | GO:0031332 | RNAi effector complex | 1 | 1 | 1.000000 | 1.000000 |
| 227 | GO:0031461 | cullin-RING ubiquitin ligase complex | 1 | 1 | 1.000000 | 1.000000 |
| 228 | GO:0031618 | nuclear pericentric heterochromatin | 1 | 1 | 1.000000 | 1.000000 |
| 229 | GO:0031902 | late endosome membrane | 1 | 1 | 1.000000 | 1.000000 |
| 230 | GO:0031931 | TORC1 complex | 1 | 1 | 1.000000 | 1.000000 |
| 231 | GO:0031965 | nuclear membrane | 1 | 1 | 1.000000 | 1.000000 |
| 232 | GO:0031968 | organelle outer membrane | 1 | 1 | 1.000000 | 1.000000 |
| 233 | GO:0031984 | organelle subcompartment | 1 | 1 | 1.000000 | 1.000000 |
| 234 | GO:0032040 | small-subunit processome | 1 | 1 | 1.000000 | 1.000000 |
| 235 | GO:0032153 | cell division site | 1 | 1 | 1.000000 | 1.000000 |
| 236 | GO:0032588 | trans-Golgi network membrane | 1 | 1 | 1.000000 | 1.000000 |
| 237 | GO:0032806 | carboxy-terminal domain protein kinase complex | 1 | 1 | 1.000000 | 1.000000 |
| 238 | GO:0033106 | cis-Golgi network membrane | 1 | 1 | 1.000000 | 1.000000 |
| 239 | GO:0033176 | proton-transporting V-type ATPase complex | 1 | 1 | 1.000000 | 1.000000 |
| 240 | GO:0033177 | proton-transporting two-sector ATPase complex, proton-transporting domain | 1 | 1 | 1.000000 | 1.000000 |
| 241 | GO:0033179 | proton-transporting V-type ATPase, V0 domain | 1 | 1 | 1.000000 | 1.000000 |
| 242 | GO:0033202 | DNA helicase complex | 1 | 1 | 1.000000 | 1.000000 |
| 243 | GO:0033254 | vacuolar transporter chaperone complex | 1 | 1 | 1.000000 | 1.000000 |
| 244 | GO:0034399 | nuclear periphery | 1 | 1 | 1.000000 | 1.000000 |
| 245 | GO:0034506 | chromosome, centromeric core domain | 1 | 1 | 1.000000 | 1.000000 |
| 246 | GO:0035327 | transcriptionally active chromatin | 1 | 1 | 1.000000 | 1.000000 |
| 247 | GO:0035861 | site of double-strand break | 1 | 1 | 1.000000 | 1.000000 |
| 248 | GO:0042575 | DNA polymerase complex | 1 | 1 | 1.000000 | 1.000000 |
| 249 | GO:0042729 | DASH complex | 1 | 1 | 1.000000 | 1.000000 |
| 250 | GO:0043601 | nuclear replisome | 1 | 1 | 1.000000 | 1.000000 |
| 251 | GO:0044433 | cytoplasmic vesicle part | 1 | 1 | 1.000000 | 1.000000 |
| 252 | GO:0044440 | endosomal part | 1 | 1 | 1.000000 | 1.000000 |
| 253 | GO:0044455 | mitochondrial membrane part | 1 | 1 | 1.000000 | 1.000000 |
| 254 | GO:0044665 | MLL1/2 complex | 1 | 1 | 1.000000 | 1.000000 |
| 255 | GO:0044853 | plasma membrane raft | 1 | 1 | 1.000000 | 1.000000 |
| 256 | GO:0045121 | membrane raft | 1 | 1 | 1.000000 | 1.000000 |
| 257 | GO:0046540 | U4/U6 x U5 tri-snRNP complex | 1 | 1 | 1.000000 | 1.000000 |
| 258 | GO:0048188 | Set1C/COMPASS complex | 1 | 1 | 1.000000 | 1.000000 |
| 259 | GO:0048475 | coated membrane | 1 | 1 | 1.000000 | 1.000000 |
| 260 | GO:0051286 | cell tip | 1 | 1 | 1.000000 | 1.000000 |
| 261 | GO:0070603 | SWI/SNF superfamily-type complex | 1 | 1 | 1.000000 | 1.000000 |
| 262 | GO:0070916 | inositol phosphoceramide synthase complex | 1 | 1 | 1.000000 | 1.000000 |
| 263 | GO:0071006 | U2-type catalytic step 1 spliceosome | 1 | 1 | 1.000000 | 1.000000 |
| 264 | GO:0071012 | catalytic step 1 spliceosome | 1 | 1 | 1.000000 | 1.000000 |
| 265 | GO:0071014 | post-mRNA release spliceosomal complex | 1 | 1 | 1.000000 | 1.000000 |
| 266 | GO:0071339 | MLL1 complex | 1 | 1 | 1.000000 | 1.000000 |
| 267 | GO:0072686 | mitotic spindle | 1 | 1 | 1.000000 | 1.000000 |
| 268 | GO:0097047 | DNA replication termination region | 1 | 1 | 1.000000 | 1.000000 |
| 269 | GO:0097346 | INO80-type complex | 1 | 1 | 1.000000 | 1.000000 |
| 270 | GO:0097361 | CIA complex | 1 | 1 | 1.000000 | 1.000000 |
| 271 | GO:0097526 | spliceosomal tri-snRNP complex | 1 | 1 | 1.000000 | 1.000000 |
| 272 | GO:0098552 | side of membrane | 1 | 1 | 1.000000 | 1.000000 |
| 273 | GO:0098562 | cytoplasmic side of membrane | 1 | 1 | 1.000000 | 1.000000 |
| 274 | GO:0098589 | membrane region | 1 | 1 | 1.000000 | 1.000000 |
| 275 | GO:0098590 | plasma membrane region | 1 | 1 | 1.000000 | 1.000000 |
| 276 | GO:0098791 | Golgi subcompartment | 1 | 1 | 1.000000 | 1.000000 |
| 277 | GO:0098797 | plasma membrane protein complex | 1 | 1 | 1.000000 | 1.000000 |
| 278 | GO:0098798 | mitochondrial protein complex | 1 | 1 | 1.000000 | 1.000000 |
| 279 | GO:0098800 | inner mitochondrial membrane protein complex | 1 | 1 | 1.000000 | 1.000000 |
| 280 | GO:0098857 | membrane microdomain | 1 | 1 | 1.000000 | 1.000000 |
| 281 | GO:1902554 | serine/threonine protein kinase complex | 1 | 1 | 1.000000 | 1.000000 |
| 282 | GO:1902911 | protein kinase complex | 1 | 1 | 1.000000 | 1.000000 |
| 283 | GO:1990204 | oxidoreductase complex | 1 | 1 | 1.000000 | 1.000000 |

  

---

out GO Enrichment (Cellular Component) Gene Details

| # | GO ID | geneID |
| 1 | GO:0016020 | g4211 g15457 g9797 g6464 g7440 g527 g470 g2520 g15880 g15282 g13959 g13424 g12113 g10811 g9946 g9578 g9140 g867 g8548 g8545 g848 g8316 g8277 g7919 g7820 g766 g7267 g7095 g6599 g4671 g4514 g3866 g3805 g316 g3060 g2929 g2855 g2737 g2723 g2399 g1884 g15809 g1562 g15420 g14466 g14064 g14022 g13998 g13874 g13864 g12451 g1234 g12048 g11646 g1164 g11476 g11324 g11240 g1110 g11003 g10791 g1057 g10401 g10138 g10417 g13911 g6962 g9271 g8625 g6164 g5377 g4571 g450 g4435 g16240 g15837 g1553 g15156 g10390 g9958 g9915 g9823 g9820 g9672 g96 g918 g9169 g9166 g9126 g9055 g8973 g8957 g8942 g8730 g8610 g8558 g842 g7854 g7810 g7777 g760 g7499 g7416 g7359 g7203 g7185 g7149 g6667 g6632 g6522 g6497 g6410 g6286 g5980 g5810 g5694 g5678 g5636 g562 g5595 g5593 g5564 g5499 g5417 g5404 g5237 g5023 g4989 g4762 g4674 g4665 g4519 g4365 g4305 g4289 g4281 g4232 g4225 g4134 g4093 g4083 g3945 g3660 g3445 g3153 g3128 g3115 g3053 g3034 g2971 g2953 g2857 g2770 g2700 g2610 g2588 g2579 g2508 g2491 g2464 g2357 g2215 g2139 g1931 g1838 g1636 g1632 g16023 g15997 g15829 g15641 g15631 g15584 g1539 g15357 g15123 g15104 g14467 g14284 g13940 g13681 g13321 g13176 g13028 g12991 g12944 g129 g12710 g12425 g12422 g12420 g12224 g12208 g1203 g11986 g11825 g1160 g11568 g11542 g11365 g11279 g11148 g11098 g11016 g10983 g10711 g15452 g589 g475 g10823 g2080 g16101 g4153 g15911 g7665 g795 |
| 2 | GO:0005623 | g4211 g15457 g4217 g16047 g475 g672 g12573 g4832 g5803 g7932 g4970 g11042 g16122 g2556 g2756 g4782 g10417 g2595 g6291 g2324 g5681 g1635 g13911 g6087 g7564 g2663 g3337 g5719 g15889 g14381 g5535 g1118 g9797 g11542 g11324 g918 g7665 g6962 g8973 g9010 g13964 g5997 g15306 g6125 g15891 g10182 g10820 g4023 g11225 g13214 g12620 g12204 g4365 g3822 g6376 g10811 g9081 g7908 g7873 g7711 g7432 g6814 g6342 g5932 g5922 g5393 g4674 g4671 g4094 g3788 g3693 g3076 g2682 g2464 g221 g16063 g16 g15714 g15421 g15316 g14315 g1377 g13320 g11117 g1057 g12208 g218 g9820 g6334 g5849 g4821 g4061 g14291 g11549 g10143 g7203 g6095 g8771 g7633 g7300 g5417 g3457 g2983 g16140 g7282 g6593 g13321 g11098 g15455 g637 g2380 g7746 g3250 g3247 g10823 g16006 g2488 g1160 g8625 g5674 g3988 g8772 g13874 g13864 g4289 g3810 g904 g7449 g5903 g15452 g2080 g16101 g3445 g5023 g2913 g13019 g4910 g5806 g4792 g1598 g15420 g12928 g1491 g8536 g7590 g7416 g3697 g4153 g15911 g9478 g6710 g3137 g3857 g746 g15356 g12037 g11946 g470 g795 g8610 |
| 3 | GO:0044464 | g4211 g15457 g4217 g16047 g475 g672 g12573 g4832 g5803 g7932 g4970 g11042 g16122 g2556 g2756 g4782 g10417 g2595 g6291 g2324 g5681 g1635 g13911 g6087 g7564 g2663 g3337 g5719 g15889 g14381 g5535 g1118 g9797 g11542 g11324 g918 g7665 g6962 g8973 g9010 g13964 g5997 g15306 g6125 g15891 g10182 g10820 g4023 g11225 g13214 g12620 g12204 g4365 g3822 g6376 g10811 g9081 g7908 g7873 g7711 g7432 g6814 g6342 g5932 g5922 g5393 g4674 g4671 g4094 g3788 g3693 g3076 g2682 g2464 g221 g16063 g16 g15714 g15421 g15316 g14315 g1377 g13320 g11117 g1057 g12208 g218 g9820 g6334 g5849 g4821 g4061 g14291 g11549 g10143 g7203 g6095 g8771 g7633 g7300 g5417 g3457 g2983 g16140 g7282 g6593 g13321 g11098 g15455 g637 g2380 g7746 g3250 g3247 g10823 g16006 g2488 g1160 g8625 g5674 g3988 g8772 g13874 g13864 g4289 g3810 g904 g7449 g5903 g15452 g2080 g16101 g3445 g5023 g2913 g13019 g4910 g5806 g4792 g1598 g15420 g12928 g1491 g8536 g7590 g7416 g3697 g4153 g15911 g3857 g746 g15356 g12037 g11946 g470 g795 g8610 |
| 4 | GO:0044425 | g15457 g10417 g9271 g8625 g6164 g5377 g4571 g450 g4435 g16240 g15837 g1553 g15156 g10390 g9958 g9915 g9823 g9820 g9672 g96 g918 g9169 g9166 g9126 g9055 g8973 g8957 g8942 g8730 g8610 g8558 g842 g7854 g7810 g7777 g760 g7499 g7416 g7359 g7203 g7185 g7149 g6667 g6632 g6522 g6497 g6410 g6286 g5980 g5810 g5694 g5678 g5636 g562 g5595 g5593 g5564 g5499 g5417 g5404 g5237 g5023 g4989 g4762 g4674 g4665 g4519 g4365 g4305 g4289 g4281 g4232 g4225 g4211 g4134 g4093 g4083 g3945 g3660 g3445 g3153 g3128 g3115 g3053 g3034 g2971 g2953 g2857 g2770 g2700 g2610 g2588 g2579 g2508 g2491 g2464 g2357 g2215 g2139 g1931 g1838 g1636 g1632 g16023 g15997 g15829 g15641 g15631 g15584 g1539 g15357 g15123 g15104 g14467 g14284 g13940 g13911 g13681 g13321 g13176 g13028 g12991 g12944 g129 g12710 g12425 g12422 g12420 g12224 g12208 g1203 g11986 g11825 g1160 g11568 g11542 g11365 g11279 g11148 g11098 g11016 g10983 g10711 g15452 g589 g10823 g4153 g15911 g7665 g795 |
| 5 | GO:0005622 | g4211 g15457 g4217 g16047 g475 g672 g12573 g4832 g5803 g7932 g4970 g11042 g16122 g2556 g2756 g4782 g10417 g2595 g6291 g2324 g5681 g1635 g13911 g6087 g7564 g2663 g3337 g5719 g15889 g14381 g5535 g1118 g9797 g918 g7665 g6962 g8973 g9010 g13964 g5997 g15306 g6125 g15891 g10182 g10820 g4023 g11225 g13214 g4365 g3822 g6376 g10811 g9081 g7908 g7873 g7711 g7432 g6814 g6342 g5932 g5922 g5393 g4674 g4671 g4094 g3788 g3693 g3076 g2682 g2464 g221 g16063 g16 g15714 g15421 g15316 g14315 g1377 g13320 g11117 g1057 g12208 g218 g9820 g6334 g5849 g4821 g4061 g14291 g11549 g10143 g7203 g6095 g8771 g7633 g7300 g5417 g3457 g2983 g16140 g7282 g6593 g15455 g637 g2380 g7746 g3250 g3247 g10823 g16006 g11098 g2488 g1160 g8625 g5674 g3988 g4289 g3810 g904 g7449 g5903 g15452 g2080 g16101 g3445 g5023 g2913 g13019 g4910 g5806 g4792 g1598 g15420 g12928 g1491 g8536 g7590 g7416 g3697 g3857 g746 g15356 g12037 g11946 g470 g795 g8610 |
| 6 | GO:0044424 | g4211 g15457 g4217 g16047 g475 g672 g12573 g4832 g5803 g7932 g4970 g11042 g16122 g2556 g2756 g4782 g10417 g2595 g6291 g2324 g5681 g1635 g13911 g6087 g7564 g2663 g3337 g5719 g15889 g14381 g5535 g1118 g9797 g918 g7665 g6962 g8973 g9010 g13964 g5997 g15306 g6125 g15891 g10182 g10820 g4023 g11225 g13214 g4365 g3822 g6376 g10811 g9081 g7908 g7873 g7711 g7432 g6814 g6342 g5932 g5922 g5393 g4674 g4671 g4094 g3788 g3693 g3076 g2682 g2464 g221 g16063 g16 g15714 g15421 g15316 g14315 g1377 g13320 g11117 g1057 g12208 g218 g9820 g6334 g5849 g4821 g4061 g14291 g11549 g10143 g7203 g6095 g8771 g7633 g7300 g5417 g3457 g2983 g16140 g7282 g6593 g15455 g637 g2380 g7746 g3250 g3247 g10823 g16006 g11098 g2488 g1160 g8625 g5674 g3988 g4289 g3810 g904 g7449 g5903 g15452 g2080 g16101 g3445 g5023 g2913 g13019 g4910 g5806 g4792 g1598 g15420 g12928 g1491 g8536 g7590 g7416 g3697 g3857 g746 g15356 g12037 g11946 g470 g795 g8610 |
| 7 | GO:0016021 | g10417 g9271 g8625 g6164 g5377 g4571 g450 g4435 g16240 g15837 g1553 g15156 g10390 g9958 g9915 g9823 g9820 g9672 g96 g918 g9169 g9166 g9126 g9055 g8973 g8957 g8942 g8730 g8610 g8558 g842 g7854 g7810 g7777 g760 g7499 g7416 g7359 g7203 g7185 g7149 g6667 g6632 g6522 g6497 g6410 g6286 g5980 g5810 g5694 g5678 g5636 g562 g5595 g5593 g5564 g5499 g5417 g5404 g5237 g5023 g4989 g4762 g4674 g4665 g4519 g4365 g4305 g4289 g4281 g4232 g4225 g4211 g4134 g4093 g4083 g3945 g3660 g3445 g3153 g3128 g3115 g3053 g3034 g2971 g2953 g2857 g2770 g2700 g2610 g2588 g2579 g2508 g2491 g2464 g2357 g2215 g2139 g1931 g1838 g1636 g1632 g16023 g15997 g15829 g15641 g15631 g15584 g15457 g1539 g15357 g15123 g15104 g14467 g14284 g13940 g13911 g13681 g13321 g13176 g13028 g12991 g12944 g129 g12710 g12425 g12422 g12420 g12224 g12208 g1203 g11986 g11825 g1160 g11568 g11542 g11365 g11279 g11148 g11098 g11016 g10983 g10711 g4153 g15911 |
| 8 | GO:0031224 | g15457 g10417 g9271 g8625 g6164 g5377 g4571 g450 g4435 g16240 g15837 g1553 g15156 g10390 g9958 g9915 g9823 g9820 g9672 g96 g918 g9169 g9166 g9126 g9055 g8973 g8957 g8942 g8730 g8610 g8558 g842 g7854 g7810 g7777 g760 g7499 g7416 g7359 g7203 g7185 g7149 g6667 g6632 g6522 g6497 g6410 g6286 g5980 g5810 g5694 g5678 g5636 g562 g5595 g5593 g5564 g5499 g5417 g5404 g5237 g5023 g4989 g4762 g4674 g4665 g4519 g4365 g4305 g4289 g4281 g4232 g4225 g4211 g4134 g4093 g4083 g3945 g3660 g3445 g3153 g3128 g3115 g3053 g3034 g2971 g2953 g2857 g2770 g2700 g2610 g2588 g2579 g2508 g2491 g2464 g2357 g2215 g2139 g1931 g1838 g1636 g1632 g16023 g15997 g15829 g15641 g15631 g15584 g1539 g15357 g15123 g15104 g14467 g14284 g13940 g13911 g13681 g13321 g13176 g13028 g12991 g12944 g129 g12710 g12425 g12422 g12420 g12224 g12208 g1203 g11986 g11825 g1160 g11568 g11542 g11365 g11279 g11148 g11098 g11016 g10983 g10711 g4153 g15911 |
| 9 | GO:0043226 | g4211 g15457 g4217 g9797 g475 g672 g16122 g2556 g2756 g4782 g10417 g2595 g6291 g1635 g13911 g6087 g7564 g3337 g5719 g15889 g5535 g1118 g918 g7665 g6962 g9010 g13964 g5997 g15306 g6125 g15891 g8973 g10182 g10820 g4023 g11225 g13214 g4365 g3822 g6376 g10811 g9081 g7908 g7873 g7711 g7432 g6814 g6342 g5932 g5922 g5393 g4674 g4671 g4094 g3788 g3693 g3076 g2682 g2464 g221 g16063 g16 g15714 g15421 g15316 g14315 g1377 g13320 g11117 g1057 g12208 g7203 g6095 g8771 g7633 g7300 g5417 g4970 g3457 g2983 g16140 g7282 g6593 g637 g2380 g11098 g2488 g1160 g8625 g5674 g3988 g4289 g3810 g15452 g2080 g16101 g3445 g5023 g2913 g13019 g4910 g5806 g4792 g1598 g15420 g12928 g8536 g7416 g3697 g3857 g7449 g5903 g746 g15356 g12037 g11946 g470 g795 g8610 |
| 10 | GO:0043229 | g4211 g15457 g4217 g475 g672 g16122 g2556 g2756 g4782 g10417 g2595 g6291 g1635 g13911 g6087 g7564 g3337 g5719 g15889 g5535 g1118 g9797 g918 g7665 g6962 g9010 g13964 g5997 g15306 g6125 g15891 g8973 g10182 g10820 g4023 g11225 g13214 g4365 g3822 g6376 g10811 g9081 g7908 g7873 g7711 g7432 g6814 g6342 g5932 g5922 g5393 g4674 g4671 g4094 g3788 g3693 g3076 g2682 g2464 g221 g16063 g16 g15714 g15421 g15316 g14315 g1377 g13320 g11117 g1057 g12208 g7203 g6095 g8771 g7633 g7300 g5417 g4970 g3457 g2983 g16140 g7282 g6593 g637 g2380 g11098 g2488 g1160 g8625 g5674 g3988 g4289 g3810 g15452 g2080 g16101 g3445 g5023 g2913 g13019 g4910 g5806 g4792 g1598 g15420 g12928 g8536 g7416 g3697 g3857 g7449 g5903 g746 g15356 g12037 g11946 g470 g795 g8610 |
| 11 | GO:0043227 | g4211 g15457 g9797 g475 g2556 g2756 g4782 g10417 g16122 g2595 g1635 g13911 g6087 g5719 g15889 g918 g7665 g6962 g9010 g13964 g5997 g15306 g6125 g15891 g8973 g10182 g10820 g3822 g6376 g10811 g9081 g7908 g7873 g7711 g7564 g7432 g6814 g672 g6342 g6291 g5932 g5922 g5393 g4674 g4671 g4217 g4094 g3788 g3693 g3076 g2682 g2464 g221 g16063 g16 g15714 g15421 g15316 g14315 g1377 g13320 g11117 g1057 g12208 g7203 g7282 g6593 g11098 g2488 g1160 g8625 g5674 g3988 g11225 g3810 g15452 g2080 g16101 g3445 g2913 g13019 g6095 g4910 g5806 g4792 g4289 g1598 g15420 g12928 g8536 g7416 g3697 g3857 g11946 g470 g795 g8610 |
| 12 | GO:0043231 | g4211 g15457 g475 g2556 g2756 g4782 g10417 g16122 g2595 g1635 g13911 g6087 g5719 g15889 g9797 g918 g7665 g6962 g9010 g13964 g5997 g15306 g6125 g15891 g8973 g10182 g10820 g3822 g6376 g10811 g9081 g7908 g7873 g7711 g7564 g7432 g6814 g672 g6342 g6291 g5932 g5922 g5393 g4674 g4671 g4217 g4094 g3788 g3693 g3076 g2682 g2464 g221 g16063 g16 g15714 g15421 g15316 g14315 g1377 g13320 g11117 g1057 g12208 g7203 g7282 g6593 g11098 g2488 g1160 g8625 g5674 g3988 g11225 g3810 g15452 g2080 g16101 g3445 g2913 g13019 g6095 g4910 g5806 g4792 g4289 g1598 g15420 g12928 g8536 g7416 g3697 g3857 g11946 g470 g795 g8610 |
| 13 | GO:0005737 | g4211 g15457 g16047 g475 g12573 g4832 g10417 g2595 g13911 g14381 g9797 g918 g7665 g6962 g8973 g12208 g5803 g218 g9820 g7564 g6334 g5849 g4821 g4061 g4023 g15714 g14291 g13320 g11549 g1118 g10143 g7203 g6095 g8771 g7633 g7300 g5417 g4970 g3457 g2983 g16140 g15455 g637 g2380 g7746 g3250 g3247 g5719 g16006 g15889 g11098 g1160 g8625 g5674 g3988 g4289 g904 g15452 g2080 g16101 g4910 g5806 g4792 g1598 g15420 g12928 g7416 g7449 g5903 g2488 g470 g795 g8610 |
| 14 | GO:0032991 | g15457 g4217 g16047 g475 g12573 g4832 g5803 g7932 g4970 g11042 g2556 g2756 g4782 g2324 g5681 g6087 g2663 g5719 g15889 g14381 g9010 g6125 g10820 g3788 g2913 g13019 g13214 g3822 g13964 g6095 g8771 g7633 g7300 g5417 g3457 g2983 g16140 g15455 g7746 g3250 g3247 g10823 g2488 g637 g7564 g11225 g4289 g3810 g904 g7449 g5903 g5023 g2380 g8536 g2080 g7590 g7416 g3697 g12208 g1491 g7665 g16122 g7282 g795 |
| 15 | GO:0005634 | g2556 g2756 g4782 g16122 g1635 g6087 g5719 g15889 g9010 g13964 g5997 g15306 g6125 g10820 g3822 g6376 g10811 g9081 g7908 g7873 g7711 g7564 g7432 g6814 g672 g6342 g6291 g5932 g5922 g5393 g4674 g4671 g4217 g4094 g3788 g3693 g3076 g2682 g2464 g221 g16063 g16 g15714 g15421 g15316 g14315 g1377 g13320 g11117 g1057 g7282 g6593 g2488 g11225 g3810 g3445 g2913 g13019 g8536 g3697 g3857 g11946 |
| 16 | GO:0044444 | g4211 g15457 g16047 g475 g12573 g4832 g10417 g2595 g13911 g14381 g9797 g918 g7665 g6962 g8973 g12208 g7203 g6095 g8771 g7633 g7300 g5417 g4970 g3457 g2983 g16140 g15455 g637 g2380 g7746 g3250 g3247 g5719 g16006 g15889 g11098 g1160 g8625 g5674 g3988 g4289 g904 g15452 g2080 g16101 g4910 g5806 g4792 g1598 g15420 g12928 g7416 g7449 g5903 g2488 g470 g795 g8610 |
| 17 | GO:0044422 | g4211 g15457 g4217 g9797 g475 g672 g16122 g2556 g2756 g4782 g10417 g1635 g13911 g6087 g7564 g3337 g5719 g15889 g6962 g9010 g13964 g5997 g15306 g6125 g10820 g4023 g11225 g13214 g3822 g12208 g7203 g7282 g6593 g637 g2380 g2488 g1160 g8625 g5674 g3988 g3810 g15452 g2080 g16101 g3445 g5023 g2913 g13019 g8536 g7416 g3697 g7665 g3857 g11946 g470 g795 |
| 18 | GO:0044446 | g4211 g15457 g4217 g475 g672 g16122 g2556 g2756 g4782 g10417 g1635 g13911 g6087 g7564 g3337 g5719 g15889 g9797 g6962 g9010 g13964 g5997 g15306 g6125 g10820 g4023 g11225 g13214 g3822 g12208 g7203 g7282 g6593 g637 g2380 g2488 g1160 g8625 g5674 g3988 g3810 g15452 g2080 g16101 g3445 g5023 g2913 g13019 g8536 g7416 g3697 g7665 g3857 g11946 g470 g795 |
| 19 | GO:0043234 | g15457 g4217 g16047 g7932 g4970 g11042 g2556 g2756 g5681 g6087 g2663 g5719 g15889 g14381 g9010 g10820 g2913 g13019 g13214 g13964 g15455 g12573 g4832 g7746 g3250 g3247 g10823 g2488 g637 g7564 g11225 g3810 g904 g7449 g5903 g5023 g2380 g8536 g2080 g7590 g7416 g3697 g12208 g7665 g795 |
| 20 | GO:0043228 | g4217 g475 g672 g16122 g2756 g6291 g1635 g7564 g3337 g5719 g5535 g1118 g4023 g11225 g13214 g4365 g6095 g8771 g7633 g7300 g5417 g4970 g3457 g2983 g16140 g7282 g6593 g637 g2380 g4289 g3810 g5023 g7449 g5903 g2488 |
| 21 | GO:0043232 | g4217 g475 g672 g16122 g2756 g6291 g1635 g7564 g3337 g5719 g5535 g1118 g4023 g11225 g13214 g4365 g6095 g8771 g7633 g7300 g5417 g4970 g3457 g2983 g16140 g7282 g6593 g637 g2380 g4289 g3810 g5023 g7449 g5903 g2488 |
| 22 | GO:0044428 | g2556 g2756 g4782 g16122 g1635 g6087 g5719 g15889 g9010 g13964 g5997 g15306 g6125 g10820 g3822 g7282 g6593 g2488 g11225 g3810 g3445 g2913 g13019 g8536 g3697 g3857 g11946 |
| 23 | GO:0030529 | g475 g12573 g4832 g5803 g4782 g2324 g6125 g3822 g6095 g8771 g7633 g7300 g5417 g4970 g3457 g2983 g16140 g4289 g7282 g7449 g5903 g2488 |
| 24 | GO:0031974 | g475 g2556 g2756 g16122 g1635 g6087 g5719 g9010 g13964 g7282 g6593 g2488 g8625 g5674 g3988 g11225 g3810 g15889 g2913 g13019 g8536 g3857 |
| 25 | GO:0043233 | g475 g2556 g2756 g16122 g1635 g6087 g5719 g9010 g13964 g7282 g6593 g2488 g8625 g5674 g3988 g11225 g3810 g15889 g2913 g13019 g8536 g3857 |
| 26 | GO:0070013 | g475 g2556 g2756 g16122 g1635 g6087 g5719 g9010 g13964 g7282 g6593 g2488 g8625 g5674 g3988 g11225 g3810 g15889 g2913 g13019 g8536 g3857 |
| 27 | GO:1990904 | g475 g12573 g4832 g5803 g4782 g2324 g6125 g3822 g6095 g8771 g7633 g7300 g5417 g4970 g3457 g2983 g16140 g4289 g7282 g7449 g5903 g2488 |
| 28 | GO:0031981 | g2556 g2756 g16122 g1635 g6087 g5719 g9010 g13964 g7282 g6593 g2488 g11225 g3810 g15889 g2913 g13019 g8536 g3857 |
| 29 | GO:0005739 | g475 g6962 g7203 g8625 g5674 g3988 g6095 g4910 g5806 g4792 g4289 g1598 g15420 g12928 g470 g795 |
| 30 | GO:0012505 | g4211 g10417 g2595 g15457 g13911 g9797 g918 g7665 g5997 g15306 g8973 g1160 g15452 g3445 g7416 g8610 |
| 31 | GO:0071944 | g11542 g11324 g13321 g11098 g637 g2380 g7746 g3250 g3247 g10823 g918 g8772 g13874 g13864 g4153 g15911 |
| 32 | GO:0031090 | g4211 g15457 g9797 g10417 g13911 g6962 g12208 g7203 g475 g1160 g2080 g16101 g3445 g7665 g795 |
| 33 | GO:1902494 | g11042 g2556 g2756 g6087 g14381 g9010 g2913 g13019 g13964 g10823 g2488 g3810 g15889 g8536 g7416 |
| 34 | GO:0005694 | g4217 g672 g16122 g2756 g6291 g1635 g5719 g13214 g11225 g3810 g2488 |
| 35 | GO:0098588 | g4211 g15457 g9797 g10417 g13911 g6962 g12208 g1160 g2080 g16101 g7665 |
| 36 | GO:0005840 | g475 g6095 g8771 g7633 g7300 g5417 g4970 g3457 g2983 g16140 |
| 37 | GO:1990234 | g11042 g2556 g6087 g9010 g13964 g2488 g3810 g15889 g8536 g7416 |
| 38 | GO:0005856 | g7564 g3337 g5535 g1118 g4023 g11225 g637 g2380 g5023 |
| 39 | GO:0044427 | g4217 g672 g16122 g2756 g5719 g13214 g11225 g3810 g2488 |
| 40 | GO:0044429 | g475 g6962 g7203 g8625 g5674 g3988 g470 g795 |
| 41 | GO:0098805 | g15457 g13911 g6962 g12208 g918 g2080 g16101 g7665 |
| 42 | GO:0000228 | g2756 g16122 g1635 g5719 g11225 g3810 g2488 |
| 43 | GO:0005654 | g2556 g6087 g9010 g13964 g2488 g15889 g8536 |
| 44 | GO:0031967 | g6962 g5997 g15306 g7203 g475 g3445 g795 |
| 45 | GO:0031975 | g6962 g5997 g15306 g7203 g475 g3445 g795 |
| 46 | GO:0044430 | g7564 g3337 g4023 g11225 g637 g2380 g5023 |
| 47 | GO:0044451 | g2556 g6087 g9010 g13964 g2488 g15889 g8536 |
| 48 | GO:0005794 | g4211 g9797 g7416 g7665 g8610 g13911 |
| 49 | GO:0005886 | g13321 g11098 g10823 g918 g4153 g15911 |
| 50 | GO:0044454 | g2756 g16122 g5719 g11225 g3810 g2488 |
| 51 | GO:0005618 | g11542 g11324 g8772 g13874 g13864 |
| 52 | GO:0005773 | g15457 g12208 g11098 g2080 g16101 |
| 53 | GO:0005783 | g10417 g2595 g15457 g1160 g15452 |
| 54 | GO:0005829 | g16047 g15455 g5719 g16006 g15889 |
| 55 | GO:0005938 | g637 g2380 g7746 g3250 g3247 |
| 56 | GO:0030312 | g11542 g11324 g8772 g13874 g13864 |
| 57 | GO:0044448 | g637 g2380 g7746 g3250 g3247 |
| 58 | GO:0061695 | g2556 g6087 g2488 g3810 g15889 |
| 59 | GO:0099568 | g637 g2380 g7746 g3250 g3247 |
| 60 | GO:0000428 | g2556 g6087 g2488 g15889 |
| 61 | GO:0000775 | g672 g11225 g16122 g2488 |
| 62 | GO:0000785 | g4217 g2756 g16122 g2488 |
| 63 | GO:0005667 | g2556 g15889 g3697 g9010 |
| 64 | GO:0005740 | g6962 g7203 g475 g795 |
| 65 | GO:0005759 | g475 g8625 g5674 g3988 |
| 66 | GO:0005774 | g15457 g12208 g2080 g16101 |
| 67 | GO:0015630 | g7564 g4023 g11225 g3337 |
| 68 | GO:0016591 | g2556 g6087 g2488 g15889 |
| 69 | GO:0030880 | g2556 g6087 g2488 g15889 |
| 70 | GO:0031966 | g6962 g7203 g475 g795 |
| 71 | GO:0032993 | g4217 g3788 g15889 g3810 |
| 72 | GO:0035770 | g4289 g7449 g5903 g2488 |
| 73 | GO:0036464 | g4289 g7449 g5903 g2488 |
| 74 | GO:0044431 | g4211 g9797 g7416 g7665 |
| 75 | GO:0044437 | g15457 g12208 g2080 g16101 |
| 76 | GO:0044459 | g10823 g918 g4153 g15911 |
| 77 | GO:0044798 | g2556 g15889 g3697 g9010 |
| 78 | GO:0055029 | g2556 g6087 g2488 g15889 |
| 79 | GO:0090575 | g2556 g15889 g3697 g9010 |
| 80 | GO:0098687 | g672 g11225 g16122 g2488 |
| 81 | GO:0098796 | g10823 g12208 g7665 g795 |
| 82 | GO:0000145 | g7746 g3250 g3247 |
| 83 | GO:0000322 | g11098 g2080 g16101 |
| 84 | GO:0000323 | g11098 g2080 g16101 |
| 85 | GO:0000324 | g11098 g2080 g16101 |
| 86 | GO:0000790 | g2756 g16122 g2488 |
| 87 | GO:0000793 | g16122 g13214 g11225 |
| 88 | GO:0005635 | g5997 g15306 g3445 |
| 89 | GO:0005730 | g7282 g6593 g5719 |
| 90 | GO:0005743 | g7203 g475 g795 |
| 91 | GO:0005768 | g13911 g918 g7665 |
| 92 | GO:0010494 | g7449 g5903 g2488 |
| 93 | GO:0015629 | g637 g2380 g5023 |
| 94 | GO:0019866 | g7203 g475 g795 |
| 95 | GO:0044432 | g10417 g1160 g15452 |
| 96 | GO:0000109 | g15889 g3810 |
| 97 | GO:0000139 | g4211 g7665 |
| 98 | GO:0000178 | g7932 g4970 |
| 99 | GO:0000329 | g2080 g16101 |
| 100 | GO:0000794 | g16122 g11225 |
| 101 | GO:0005576 | g11542 g11324 |
| 102 | GO:0005643 | g5997 g15306 |
| 103 | GO:0005657 | g5719 g3810 |
| 104 | GO:0005681 | g4782 g6125 |
| 105 | GO:0005684 | g4782 g6125 |
| 106 | GO:0005789 | g10417 g1160 |
| 107 | GO:0005798 | g9797 g7665 |
| 108 | GO:0005819 | g7564 g11225 |
| 109 | GO:0005852 | g12573 g4832 |
| 110 | GO:0005874 | g7564 g4023 |
| 111 | GO:0005887 | g4153 g15911 |
| 112 | GO:0009277 | g11542 g11324 |
| 113 | GO:0009337 | g7449 g5903 |
| 114 | GO:0016023 | g9797 g7665 |
| 115 | GO:0016282 | g12573 g4832 |
| 116 | GO:0016459 | g5023 g2380 |
| 117 | GO:0030135 | g9797 g7665 |
| 118 | GO:0030479 | g637 g2380 |
| 119 | GO:0030532 | g3822 g4782 |
| 120 | GO:0030684 | g2324 g7282 |
| 121 | GO:0030863 | g637 g2380 |
| 122 | GO:0030864 | g637 g2380 |
| 123 | GO:0031226 | g4153 g15911 |
| 124 | GO:0031300 | g15457 g10417 |
| 125 | GO:0031410 | g9797 g7665 |
| 126 | GO:0031982 | g9797 g7665 |
| 127 | GO:0031988 | g9797 g7665 |
| 128 | GO:0033290 | g12573 g4832 |
| 129 | GO:0034708 | g9010 g8536 |
| 130 | GO:0035097 | g9010 g8536 |
| 131 | GO:0038201 | g1491 g2080 |
| 132 | GO:0042175 | g10417 g1160 |
| 133 | GO:0043596 | g5719 g3810 |
| 134 | GO:0044445 | g16047 g15455 |
| 135 | GO:0044815 | g4217 g13214 |
| 136 | GO:0061645 | g637 g2380 |
| 137 | GO:0070993 | g12573 g4832 |
| 138 | GO:0097525 | g3822 g4782 |
| 139 | GO:0097550 | g3788 g15889 |
| 140 | GO:0098852 | g2080 g16101 |
| 141 | GO:0099512 | g7564 g4023 |
| 142 | GO:0099513 | g7564 g4023 |
| 143 | GO:1990391 | g15889 g3810 |
| 144 | GO:0000112 | g15889 |
| 145 | GO:0000151 | g11042 |
| 146 | GO:0000313 | g475 |
| 147 | GO:0000439 | g15889 |
| 148 | GO:0000502 | g2663 |
| 149 | GO:0000776 | g11225 |
| 150 | GO:0000777 | g11225 |
| 151 | GO:0000778 | g11225 |
| 152 | GO:0000779 | g11225 |
| 153 | GO:0000780 | g11225 |
| 154 | GO:0000786 | g4217 |
| 155 | GO:0000792 | g2488 |
| 156 | GO:0000795 | g16122 |
| 157 | GO:0000796 | g13214 |
| 158 | GO:0000922 | g7564 |
| 159 | GO:0000932 | g4289 |
| 160 | GO:0000940 | g11225 |
| 161 | GO:0000942 | g11225 |
| 162 | GO:0000943 | g11946 |
| 163 | GO:0001405 | g795 |
| 164 | GO:0005665 | g2488 |
| 165 | GO:0005669 | g2556 |
| 166 | GO:0005675 | g15889 |
| 167 | GO:0005686 | g4782 |
| 168 | GO:0005720 | g2488 |
| 169 | GO:0005721 | g2488 |
| 170 | GO:0005741 | g6962 |
| 171 | GO:0005744 | g795 |
| 172 | GO:0005761 | g475 |
| 173 | GO:0005770 | g13911 |
| 174 | GO:0005801 | g4211 |
| 175 | GO:0005802 | g4211 |
| 176 | GO:0005811 | g4365 |
| 177 | GO:0005834 | g10823 |
| 178 | GO:0005835 | g15455 |
| 179 | GO:0005851 | g904 |
| 180 | GO:0005875 | g7564 |
| 181 | GO:0005885 | g637 |
| 182 | GO:0005956 | g7590 |
| 183 | GO:0008023 | g6087 |
| 184 | GO:0008622 | g3810 |
| 185 | GO:0009331 | g14381 |
| 186 | GO:0009898 | g10823 |
| 187 | GO:0010008 | g13911 |
| 188 | GO:0012506 | g7665 |
| 189 | GO:0012510 | g7665 |
| 190 | GO:0016442 | g5803 |
| 191 | GO:0016469 | g12208 |
| 192 | GO:0016593 | g6087 |
| 193 | GO:0019005 | g11042 |
| 194 | GO:0019867 | g6962 |
| 195 | GO:0019897 | g10823 |
| 196 | GO:0019898 | g10823 |
| 197 | GO:0030008 | g5681 |
| 198 | GO:0030117 | g7665 |
| 199 | GO:0030118 | g7665 |
| 200 | GO:0030119 | g7665 |
| 201 | GO:0030120 | g7665 |
| 202 | GO:0030121 | g7665 |
| 203 | GO:0030125 | g7665 |
| 204 | GO:0030130 | g7665 |
| 205 | GO:0030131 | g7665 |
| 206 | GO:0030133 | g7665 |
| 207 | GO:0030136 | g7665 |
| 208 | GO:0030137 | g9797 |
| 209 | GO:0030140 | g7665 |
| 210 | GO:0030176 | g10417 |
| 211 | GO:0030658 | g7665 |
| 212 | GO:0030659 | g7665 |
| 213 | GO:0030660 | g7665 |
| 214 | GO:0030662 | g7665 |
| 215 | GO:0030665 | g7665 |
| 216 | GO:0030687 | g7282 |
| 217 | GO:0030870 | g10820 |
| 218 | GO:0030894 | g3810 |
| 219 | GO:0030907 | g3697 |
| 220 | GO:0031011 | g2756 |
| 221 | GO:0031227 | g10417 |
| 222 | GO:0031234 | g10823 |
| 223 | GO:0031298 | g5719 |
| 224 | GO:0031301 | g10417 |
| 225 | GO:0031310 | g15457 |
| 226 | GO:0031332 | g5803 |
| 227 | GO:0031461 | g11042 |
| 228 | GO:0031618 | g2488 |
| 229 | GO:0031902 | g13911 |
| 230 | GO:0031931 | g2080 |
| 231 | GO:0031965 | g3445 |
| 232 | GO:0031968 | g6962 |
| 233 | GO:0031984 | g4211 |
| 234 | GO:0032040 | g2324 |
| 235 | GO:0032153 | g918 |
| 236 | GO:0032588 | g4211 |
| 237 | GO:0032806 | g15889 |
| 238 | GO:0033106 | g4211 |
| 239 | GO:0033176 | g12208 |
| 240 | GO:0033177 | g12208 |
| 241 | GO:0033179 | g12208 |
| 242 | GO:0033202 | g2756 |
| 243 | GO:0033254 | g15457 |
| 244 | GO:0034399 | g3857 |
| 245 | GO:0034506 | g16122 |
| 246 | GO:0035327 | g16122 |
| 247 | GO:0035861 | g672 |
| 248 | GO:0042575 | g3810 |
| 249 | GO:0042729 | g11225 |
| 250 | GO:0043601 | g3810 |
| 251 | GO:0044433 | g7665 |
| 252 | GO:0044440 | g13911 |
| 253 | GO:0044455 | g795 |
| 254 | GO:0044665 | g9010 |
| 255 | GO:0044853 | g918 |
| 256 | GO:0045121 | g918 |
| 257 | GO:0046540 | g3822 |
| 258 | GO:0048188 | g8536 |
| 259 | GO:0048475 | g7665 |
| 260 | GO:0051286 | g918 |
| 261 | GO:0070603 | g2756 |
| 262 | GO:0070916 | g7416 |
| 263 | GO:0071006 | g6125 |
| 264 | GO:0071012 | g6125 |
| 265 | GO:0071014 | g4782 |
| 266 | GO:0071339 | g9010 |
| 267 | GO:0072686 | g11225 |
| 268 | GO:0097047 | g16122 |
| 269 | GO:0097346 | g2756 |
| 270 | GO:0097361 | g16047 |
| 271 | GO:0097526 | g3822 |
| 272 | GO:0098552 | g10823 |
| 273 | GO:0098562 | g10823 |
| 274 | GO:0098589 | g918 |
| 275 | GO:0098590 | g918 |
| 276 | GO:0098791 | g4211 |
| 277 | GO:0098797 | g10823 |
| 278 | GO:0098798 | g795 |
| 279 | GO:0098800 | g795 |
| 280 | GO:0098857 | g918 |
| 281 | GO:1902554 | g15889 |
| 282 | GO:1902911 | g15889 |
| 283 | GO:1990204 | g14381 |

  

---

GO Directed Acycline Graph

Back Top
