# Supplementary material for: A Comprehensive Assessment of Ultraviolet-Radiation-Induced Mutations in Flammulina filiformis Using Whole-Genome Resequencing
Source: J Fungi (Basel). 2024 Mar 20;10(3):228. doi: 10.3390/jof10030228 (PMC10971301; doi:10.3390/jof10030228)
Supplement: Supplementary file 1 [file jof-10-00228-s001.zip › Supplementary Material S8/GO annotation/out/out.F.html]

 


GO Enrichment Analysis


out GO Enrichment (Molecular Function)

| # | GO ID | Description | GeneRatio (493) | BgRatio (493) | pvalue | fdr |
| 1 | GO:0003824 | catalytic activity | 361 | 361 | 1.000000 | 1.000000 |
| 2 | GO:0005488 | binding | 282 | 282 | 1.000000 | 1.000000 |
| 3 | GO:0097159 | organic cyclic compound binding | 187 | 187 | 1.000000 | 1.000000 |
| 4 | GO:1901363 | heterocyclic compound binding | 187 | 187 | 1.000000 | 1.000000 |
| 5 | GO:0043167 | ion binding | 171 | 171 | 1.000000 | 1.000000 |
| 6 | GO:0016787 | hydrolase activity | 135 | 135 | 1.000000 | 1.000000 |
| 7 | GO:0016740 | transferase activity | 115 | 115 | 1.000000 | 1.000000 |
| 8 | GO:0036094 | small molecule binding | 114 | 114 | 1.000000 | 1.000000 |
| 9 | GO:0000166 | nucleotide binding | 112 | 112 | 1.000000 | 1.000000 |
| 10 | GO:1901265 | nucleoside phosphate binding | 112 | 112 | 1.000000 | 1.000000 |
| 11 | GO:0043168 | anion binding | 105 | 105 | 1.000000 | 1.000000 |
| 12 | GO:0097367 | carbohydrate derivative binding | 87 | 87 | 1.000000 | 1.000000 |
| 13 | GO:0032553 | ribonucleotide binding | 85 | 85 | 1.000000 | 1.000000 |
| 14 | GO:0003676 | nucleic acid binding | 84 | 84 | 1.000000 | 1.000000 |
| 15 | GO:0016491 | oxidoreductase activity | 83 | 83 | 1.000000 | 1.000000 |
| 16 | GO:0001882 | nucleoside binding | 82 | 82 | 1.000000 | 1.000000 |
| 17 | GO:0017076 | purine nucleotide binding | 81 | 81 | 1.000000 | 1.000000 |
| 18 | GO:0032549 | ribonucleoside binding | 81 | 81 | 1.000000 | 1.000000 |
| 19 | GO:0001883 | purine nucleoside binding | 80 | 80 | 1.000000 | 1.000000 |
| 20 | GO:0032550 | purine ribonucleoside binding | 80 | 80 | 1.000000 | 1.000000 |
| 21 | GO:0032555 | purine ribonucleotide binding | 80 | 80 | 1.000000 | 1.000000 |
| 22 | GO:0035639 | purine ribonucleoside triphosphate binding | 80 | 80 | 1.000000 | 1.000000 |
| 23 | GO:0043169 | cation binding | 79 | 79 | 1.000000 | 1.000000 |
| 24 | GO:0046872 | metal ion binding | 78 | 78 | 1.000000 | 1.000000 |
| 25 | GO:0030554 | adenyl nucleotide binding | 73 | 73 | 1.000000 | 1.000000 |
| 26 | GO:0005524 | ATP binding | 72 | 72 | 1.000000 | 1.000000 |
| 27 | GO:0032559 | adenyl ribonucleotide binding | 72 | 72 | 1.000000 | 1.000000 |
| 28 | GO:0016772 | transferase activity, transferring phosphorus-containing groups | 55 | 55 | 1.000000 | 1.000000 |
| 29 | GO:0016817 | hydrolase activity, acting on acid anhydrides | 46 | 46 | 1.000000 | 1.000000 |
| 30 | GO:0016818 | hydrolase activity, acting on acid anhydrides, in phosphorus-containing anhydrides | 46 | 46 | 1.000000 | 1.000000 |
| 31 | GO:0016301 | kinase activity | 45 | 45 | 1.000000 | 1.000000 |
| 32 | GO:0016462 | pyrophosphatase activity | 43 | 43 | 1.000000 | 1.000000 |
| 33 | GO:0017111 | nucleoside-triphosphatase activity | 42 | 42 | 1.000000 | 1.000000 |
| 34 | GO:0005215 | transporter activity | 40 | 40 | 1.000000 | 1.000000 |
| 35 | GO:0022857 | transmembrane transporter activity | 40 | 40 | 1.000000 | 1.000000 |
| 36 | GO:0005515 | protein binding | 37 | 37 | 1.000000 | 1.000000 |
| 37 | GO:0003677 | DNA binding | 33 | 33 | 1.000000 | 1.000000 |
| 38 | GO:0046914 | transition metal ion binding | 33 | 33 | 1.000000 | 1.000000 |
| 39 | GO:0048037 | cofactor binding | 31 | 31 | 1.000000 | 1.000000 |
| 40 | GO:0003723 | RNA binding | 29 | 29 | 1.000000 | 1.000000 |
| 41 | GO:0016887 | ATPase activity | 24 | 24 | 1.000000 | 1.000000 |
| 42 | GO:0016773 | phosphotransferase activity, alcohol group as acceptor | 23 | 23 | 1.000000 | 1.000000 |
| 43 | GO:0050662 | coenzyme binding | 23 | 23 | 1.000000 | 1.000000 |
| 44 | GO:0008270 | zinc ion binding | 20 | 20 | 1.000000 | 1.000000 |
| 45 | GO:0042623 | ATPase activity, coupled | 20 | 20 | 1.000000 | 1.000000 |
| 46 | GO:0016798 | hydrolase activity, acting on glycosyl bonds | 19 | 19 | 1.000000 | 1.000000 |
| 47 | GO:0022804 | active transmembrane transporter activity | 18 | 18 | 1.000000 | 1.000000 |
| 48 | GO:0004672 | protein kinase activity | 17 | 17 | 1.000000 | 1.000000 |
| 49 | GO:0008233 | peptidase activity | 17 | 17 | 1.000000 | 1.000000 |
| 50 | GO:0016788 | hydrolase activity, acting on ester bonds | 17 | 17 | 1.000000 | 1.000000 |
| 51 | GO:0004553 | hydrolase activity, hydrolyzing O-glycosyl compounds | 16 | 16 | 1.000000 | 1.000000 |
| 52 | GO:0022891 | substrate-specific transmembrane transporter activity | 16 | 16 | 1.000000 | 1.000000 |
| 53 | GO:0022892 | substrate-specific transporter activity | 16 | 16 | 1.000000 | 1.000000 |
| 54 | GO:0070011 | peptidase activity, acting on L-amino acid peptides | 16 | 16 | 1.000000 | 1.000000 |
| 55 | GO:0016874 | ligase activity | 15 | 15 | 1.000000 | 1.000000 |
| 56 | GO:0015075 | ion transmembrane transporter activity | 14 | 14 | 1.000000 | 1.000000 |
| 57 | GO:0004386 | helicase activity | 13 | 13 | 1.000000 | 1.000000 |
| 58 | GO:0015399 | primary active transmembrane transporter activity | 13 | 13 | 1.000000 | 1.000000 |
| 59 | GO:0015405 | P-P-bond-hydrolysis-driven transmembrane transporter activity | 13 | 13 | 1.000000 | 1.000000 |
| 60 | GO:0016820 | hydrolase activity, acting on acid anhydrides, catalyzing transmembrane movement of substances | 13 | 13 | 1.000000 | 1.000000 |
| 61 | GO:0042626 | ATPase activity, coupled to transmembrane movement of substances | 13 | 13 | 1.000000 | 1.000000 |
| 62 | GO:0043492 | ATPase activity, coupled to movement of substances | 13 | 13 | 1.000000 | 1.000000 |
| 63 | GO:0005506 | iron ion binding | 12 | 12 | 1.000000 | 1.000000 |
| 64 | GO:0016741 | transferase activity, transferring one-carbon groups | 12 | 12 | 1.000000 | 1.000000 |
| 65 | GO:0016757 | transferase activity, transferring glycosyl groups | 12 | 12 | 1.000000 | 1.000000 |
| 66 | GO:0005198 | structural molecule activity | 11 | 11 | 1.000000 | 1.000000 |
| 67 | GO:0008168 | methyltransferase activity | 11 | 11 | 1.000000 | 1.000000 |
| 68 | GO:0044877 | macromolecular complex binding | 11 | 11 | 1.000000 | 1.000000 |
| 69 | GO:0046983 | protein dimerization activity | 11 | 11 | 1.000000 | 1.000000 |
| 70 | GO:0098772 | molecular function regulator | 11 | 11 | 1.000000 | 1.000000 |
| 71 | GO:0004175 | endopeptidase activity | 10 | 10 | 1.000000 | 1.000000 |
| 72 | GO:0004497 | monooxygenase activity | 10 | 10 | 1.000000 | 1.000000 |
| 73 | GO:0016746 | transferase activity, transferring acyl groups | 10 | 10 | 1.000000 | 1.000000 |
| 74 | GO:0020037 | heme binding | 10 | 10 | 1.000000 | 1.000000 |
| 75 | GO:0046906 | tetrapyrrole binding | 10 | 10 | 1.000000 | 1.000000 |
| 76 | GO:0001071 | nucleic acid binding transcription factor activity | 9 | 9 | 1.000000 | 1.000000 |
| 77 | GO:0003700 | transcription factor activity, sequence-specific DNA binding | 9 | 9 | 1.000000 | 1.000000 |
| 78 | GO:0003743 | translation initiation factor activity | 9 | 9 | 1.000000 | 1.000000 |
| 79 | GO:0008135 | translation factor activity, RNA binding | 9 | 9 | 1.000000 | 1.000000 |
| 80 | GO:0008324 | cation transmembrane transporter activity | 9 | 9 | 1.000000 | 1.000000 |
| 81 | GO:0016614 | oxidoreductase activity, acting on CH-OH group of donors | 9 | 9 | 1.000000 | 1.000000 |
| 82 | GO:0016758 | transferase activity, transferring hexosyl groups | 9 | 9 | 1.000000 | 1.000000 |
| 83 | GO:0016779 | nucleotidyltransferase activity | 9 | 9 | 1.000000 | 1.000000 |
| 84 | GO:0016853 | isomerase activity | 9 | 9 | 1.000000 | 1.000000 |
| 85 | GO:0042578 | phosphoric ester hydrolase activity | 9 | 9 | 1.000000 | 1.000000 |
| 86 | GO:0050660 | flavin adenine dinucleotide binding | 9 | 9 | 1.000000 | 1.000000 |
| 87 | GO:0051213 | dioxygenase activity | 9 | 9 | 1.000000 | 1.000000 |
| 88 | GO:0000287 | magnesium ion binding | 8 | 8 | 1.000000 | 1.000000 |
| 89 | GO:0005525 | GTP binding | 8 | 8 | 1.000000 | 1.000000 |
| 90 | GO:0016705 | oxidoreductase activity, acting on paired donors, with incorporation or reduction of molecular oxygen | 8 | 8 | 1.000000 | 1.000000 |
| 91 | GO:0016829 | lyase activity | 8 | 8 | 1.000000 | 1.000000 |
| 92 | GO:0019001 | guanyl nucleotide binding | 8 | 8 | 1.000000 | 1.000000 |
| 93 | GO:0022890 | inorganic cation transmembrane transporter activity | 8 | 8 | 1.000000 | 1.000000 |
| 94 | GO:0032561 | guanyl ribonucleotide binding | 8 | 8 | 1.000000 | 1.000000 |
| 95 | GO:0051536 | iron-sulfur cluster binding | 8 | 8 | 1.000000 | 1.000000 |
| 96 | GO:0051540 | metal cluster binding | 8 | 8 | 1.000000 | 1.000000 |
| 97 | GO:0003735 | structural constituent of ribosome | 7 | 7 | 1.000000 | 1.000000 |
| 98 | GO:0003924 | GTPase activity | 7 | 7 | 1.000000 | 1.000000 |
| 99 | GO:0004674 | protein serine/threonine kinase activity | 7 | 7 | 1.000000 | 1.000000 |
| 100 | GO:0008236 | serine-type peptidase activity | 7 | 7 | 1.000000 | 1.000000 |
| 101 | GO:0016616 | oxidoreductase activity, acting on the CH-OH group of donors, NAD or NADP as acceptor | 7 | 7 | 1.000000 | 1.000000 |
| 102 | GO:0016701 | oxidoreductase activity, acting on single donors with incorporation of molecular oxygen | 7 | 7 | 1.000000 | 1.000000 |
| 103 | GO:0016791 | phosphatase activity | 7 | 7 | 1.000000 | 1.000000 |
| 104 | GO:0017171 | serine hydrolase activity | 7 | 7 | 1.000000 | 1.000000 |
| 105 | GO:0000981 | RNA polymerase II transcription factor activity, sequence-specific DNA binding | 6 | 6 | 1.000000 | 1.000000 |
| 106 | GO:0004252 | serine-type endopeptidase activity | 6 | 6 | 1.000000 | 1.000000 |
| 107 | GO:0008509 | anion transmembrane transporter activity | 6 | 6 | 1.000000 | 1.000000 |
| 108 | GO:0015077 | monovalent inorganic cation transmembrane transporter activity | 6 | 6 | 1.000000 | 1.000000 |
| 109 | GO:0016835 | carbon-oxygen lyase activity | 6 | 6 | 1.000000 | 1.000000 |
| 110 | GO:0030170 | pyridoxal phosphate binding | 6 | 6 | 1.000000 | 1.000000 |
| 111 | GO:0030234 | enzyme regulator activity | 6 | 6 | 1.000000 | 1.000000 |
| 112 | GO:0032403 | protein complex binding | 6 | 6 | 1.000000 | 1.000000 |
| 113 | GO:0051287 | NAD binding | 6 | 6 | 1.000000 | 1.000000 |
| 114 | GO:0003779 | actin binding | 5 | 5 | 1.000000 | 1.000000 |
| 115 | GO:0004518 | nuclease activity | 5 | 5 | 1.000000 | 1.000000 |
| 116 | GO:0004721 | phosphoprotein phosphatase activity | 5 | 5 | 1.000000 | 1.000000 |
| 117 | GO:0005085 | guanyl-nucleotide exchange factor activity | 5 | 5 | 1.000000 | 1.000000 |
| 118 | GO:0008092 | cytoskeletal protein binding | 5 | 5 | 1.000000 | 1.000000 |
| 119 | GO:0008289 | lipid binding | 5 | 5 | 1.000000 | 1.000000 |
| 120 | GO:0008483 | transaminase activity | 5 | 5 | 1.000000 | 1.000000 |
| 121 | GO:0008757 | S-adenosylmethionine-dependent methyltransferase activity | 5 | 5 | 1.000000 | 1.000000 |
| 122 | GO:0010181 | FMN binding | 5 | 5 | 1.000000 | 1.000000 |
| 123 | GO:0015078 | hydrogen ion transmembrane transporter activity | 5 | 5 | 1.000000 | 1.000000 |
| 124 | GO:0015291 | secondary active transmembrane transporter activity | 5 | 5 | 1.000000 | 1.000000 |
| 125 | GO:0016651 | oxidoreductase activity, acting on NAD(P)H | 5 | 5 | 1.000000 | 1.000000 |
| 126 | GO:0016702 | oxidoreductase activity, acting on single donors with incorporation of molecular oxygen, incorporation of two atoms of oxygen | 5 | 5 | 1.000000 | 1.000000 |
| 127 | GO:0016747 | transferase activity, transferring acyl groups other than amino-acyl groups | 5 | 5 | 1.000000 | 1.000000 |
| 128 | GO:0016769 | transferase activity, transferring nitrogenous groups | 5 | 5 | 1.000000 | 1.000000 |
| 129 | GO:0016810 | hydrolase activity, acting on carbon-nitrogen (but not peptide) bonds | 5 | 5 | 1.000000 | 1.000000 |
| 130 | GO:0019899 | enzyme binding | 5 | 5 | 1.000000 | 1.000000 |
| 131 | GO:0050661 | NADP binding | 5 | 5 | 1.000000 | 1.000000 |
| 132 | GO:0004842 | ubiquitin-protein transferase activity | 4 | 4 | 1.000000 | 1.000000 |
| 133 | GO:0008094 | DNA-dependent ATPase activity | 4 | 4 | 1.000000 | 1.000000 |
| 134 | GO:0008237 | metallopeptidase activity | 4 | 4 | 1.000000 | 1.000000 |
| 135 | GO:0008276 | protein methyltransferase activity | 4 | 4 | 1.000000 | 1.000000 |
| 136 | GO:0016620 | oxidoreductase activity, acting on the aldehyde or oxo group of donors, NAD or NADP as acceptor | 4 | 4 | 1.000000 | 1.000000 |
| 137 | GO:0016638 | oxidoreductase activity, acting on the CH-NH2 group of donors | 4 | 4 | 1.000000 | 1.000000 |
| 138 | GO:0016836 | hydro-lyase activity | 4 | 4 | 1.000000 | 1.000000 |
| 139 | GO:0016879 | ligase activity, forming carbon-nitrogen bonds | 4 | 4 | 1.000000 | 1.000000 |
| 140 | GO:0016903 | oxidoreductase activity, acting on the aldehyde or oxo group of donors | 4 | 4 | 1.000000 | 1.000000 |
| 141 | GO:0019787 | ubiquitin-like protein transferase activity | 4 | 4 | 1.000000 | 1.000000 |
| 142 | GO:0030246 | carbohydrate binding | 4 | 4 | 1.000000 | 1.000000 |
| 143 | GO:0042802 | identical protein binding | 4 | 4 | 1.000000 | 1.000000 |
| 144 | GO:0043021 | ribonucleoprotein complex binding | 4 | 4 | 1.000000 | 1.000000 |
| 145 | GO:0043565 | sequence-specific DNA binding | 4 | 4 | 1.000000 | 1.000000 |
| 146 | GO:0046873 | metal ion transmembrane transporter activity | 4 | 4 | 1.000000 | 1.000000 |
| 147 | GO:0046982 | protein heterodimerization activity | 4 | 4 | 1.000000 | 1.000000 |
| 148 | GO:0051539 | 4 iron, 4 sulfur cluster binding | 4 | 4 | 1.000000 | 1.000000 |
| 149 | GO:0071949 | FAD binding | 4 | 4 | 1.000000 | 1.000000 |
| 150 | GO:0000049 | tRNA binding | 3 | 3 | 1.000000 | 1.000000 |
| 151 | GO:0003678 | DNA helicase activity | 3 | 3 | 1.000000 | 1.000000 |
| 152 | GO:0003682 | chromatin binding | 3 | 3 | 1.000000 | 1.000000 |
| 153 | GO:0003755 | peptidyl-prolyl cis-trans isomerase activity | 3 | 3 | 1.000000 | 1.000000 |
| 154 | GO:0004527 | exonuclease activity | 3 | 3 | 1.000000 | 1.000000 |
| 155 | GO:0004812 | aminoacyl-tRNA ligase activity | 3 | 3 | 1.000000 | 1.000000 |
| 156 | GO:0005088 | Ras guanyl-nucleotide exchange factor activity | 3 | 3 | 1.000000 | 1.000000 |
| 157 | GO:0005089 | Rho guanyl-nucleotide exchange factor activity | 3 | 3 | 1.000000 | 1.000000 |
| 158 | GO:0005543 | phospholipid binding | 3 | 3 | 1.000000 | 1.000000 |
| 159 | GO:0008047 | enzyme activator activity | 3 | 3 | 1.000000 | 1.000000 |
| 160 | GO:0008170 | N-methyltransferase activity | 3 | 3 | 1.000000 | 1.000000 |
| 161 | GO:0008173 | RNA methyltransferase activity | 3 | 3 | 1.000000 | 1.000000 |
| 162 | GO:0008199 | ferric iron binding | 3 | 3 | 1.000000 | 1.000000 |
| 163 | GO:0008234 | cysteine-type peptidase activity | 3 | 3 | 1.000000 | 1.000000 |
| 164 | GO:0008536 | Ran GTPase binding | 3 | 3 | 1.000000 | 1.000000 |
| 165 | GO:0015297 | antiporter activity | 3 | 3 | 1.000000 | 1.000000 |
| 166 | GO:0015926 | glucosidase activity | 3 | 3 | 1.000000 | 1.000000 |
| 167 | GO:0016627 | oxidoreductase activity, acting on the CH-CH group of donors | 3 | 3 | 1.000000 | 1.000000 |
| 168 | GO:0016838 | carbon-oxygen lyase activity, acting on phosphates | 3 | 3 | 1.000000 | 1.000000 |
| 169 | GO:0016859 | cis-trans isomerase activity | 3 | 3 | 1.000000 | 1.000000 |
| 170 | GO:0016875 | ligase activity, forming carbon-oxygen bonds | 3 | 3 | 1.000000 | 1.000000 |
| 171 | GO:0016876 | ligase activity, forming aminoacyl-tRNA and related compounds | 3 | 3 | 1.000000 | 1.000000 |
| 172 | GO:0017016 | Ras GTPase binding | 3 | 3 | 1.000000 | 1.000000 |
| 173 | GO:0031267 | small GTPase binding | 3 | 3 | 1.000000 | 1.000000 |
| 174 | GO:0043022 | ribosome binding | 3 | 3 | 1.000000 | 1.000000 |
| 175 | GO:0051020 | GTPase binding | 3 | 3 | 1.000000 | 1.000000 |
| 176 | GO:0052689 | carboxylic ester hydrolase activity | 3 | 3 | 1.000000 | 1.000000 |
| 177 | GO:0060589 | nucleoside-triphosphatase regulator activity | 3 | 3 | 1.000000 | 1.000000 |
| 178 | GO:0003684 | damaged DNA binding | 2 | 2 | 1.000000 | 1.000000 |
| 179 | GO:0003690 | double-stranded DNA binding | 2 | 2 | 1.000000 | 1.000000 |
| 180 | GO:0003724 | RNA helicase activity | 2 | 2 | 1.000000 | 1.000000 |
| 181 | GO:0003774 | motor activity | 2 | 2 | 1.000000 | 1.000000 |
| 182 | GO:0003884 | D-amino-acid oxidase activity | 2 | 2 | 1.000000 | 1.000000 |
| 183 | GO:0003887 | DNA-directed DNA polymerase activity | 2 | 2 | 1.000000 | 1.000000 |
| 184 | GO:0003899 | DNA-directed RNA polymerase activity | 2 | 2 | 1.000000 | 1.000000 |
| 185 | GO:0003916 | DNA topoisomerase activity | 2 | 2 | 1.000000 | 1.000000 |
| 186 | GO:0003954 | NADH dehydrogenase activity | 2 | 2 | 1.000000 | 1.000000 |
| 187 | GO:0004003 | ATP-dependent DNA helicase activity | 2 | 2 | 1.000000 | 1.000000 |
| 188 | GO:0004069 | L-aspartate:2-oxoglutarate aminotransferase activity | 2 | 2 | 1.000000 | 1.000000 |
| 189 | GO:0004133 | glycogen debranching enzyme activity | 2 | 2 | 1.000000 | 1.000000 |
| 190 | GO:0004176 | ATP-dependent peptidase activity | 2 | 2 | 1.000000 | 1.000000 |
| 191 | GO:0004177 | aminopeptidase activity | 2 | 2 | 1.000000 | 1.000000 |
| 192 | GO:0004190 | aspartic-type endopeptidase activity | 2 | 2 | 1.000000 | 1.000000 |
| 193 | GO:0004222 | metalloendopeptidase activity | 2 | 2 | 1.000000 | 1.000000 |
| 194 | GO:0004499 | N,N-dimethylaniline monooxygenase activity | 2 | 2 | 1.000000 | 1.000000 |
| 195 | GO:0004725 | protein tyrosine phosphatase activity | 2 | 2 | 1.000000 | 1.000000 |
| 196 | GO:0004783 | sulfite reductase (NADPH) activity | 2 | 2 | 1.000000 | 1.000000 |
| 197 | GO:0005096 | GTPase activator activity | 2 | 2 | 1.000000 | 1.000000 |
| 198 | GO:0005199 | structural constituent of cell wall | 2 | 2 | 1.000000 | 1.000000 |
| 199 | GO:0005216 | ion channel activity | 2 | 2 | 1.000000 | 1.000000 |
| 200 | GO:0005244 | voltage-gated ion channel activity | 2 | 2 | 1.000000 | 1.000000 |
| 201 | GO:0005253 | anion channel activity | 2 | 2 | 1.000000 | 1.000000 |
| 202 | GO:0005516 | calmodulin binding | 2 | 2 | 1.000000 | 1.000000 |
| 203 | GO:0008026 | ATP-dependent helicase activity | 2 | 2 | 1.000000 | 1.000000 |
| 204 | GO:0008081 | phosphoric diester hydrolase activity | 2 | 2 | 1.000000 | 1.000000 |
| 205 | GO:0008134 | transcription factor binding | 2 | 2 | 1.000000 | 1.000000 |
| 206 | GO:0008137 | NADH dehydrogenase (ubiquinone) activity | 2 | 2 | 1.000000 | 1.000000 |
| 207 | GO:0008194 | UDP-glycosyltransferase activity | 2 | 2 | 1.000000 | 1.000000 |
| 208 | GO:0008238 | exopeptidase activity | 2 | 2 | 1.000000 | 1.000000 |
| 209 | GO:0008308 | voltage-gated anion channel activity | 2 | 2 | 1.000000 | 1.000000 |
| 210 | GO:0008375 | acetylglucosaminyltransferase activity | 2 | 2 | 1.000000 | 1.000000 |
| 211 | GO:0008553 | hydrogen-exporting ATPase activity, phosphorylative mechanism | 2 | 2 | 1.000000 | 1.000000 |
| 212 | GO:0010333 | terpene synthase activity | 2 | 2 | 1.000000 | 1.000000 |
| 213 | GO:0015103 | inorganic anion transmembrane transporter activity | 2 | 2 | 1.000000 | 1.000000 |
| 214 | GO:0015267 | channel activity | 2 | 2 | 1.000000 | 1.000000 |
| 215 | GO:0015298 | solute:cation antiporter activity | 2 | 2 | 1.000000 | 1.000000 |
| 216 | GO:0015299 | solute:proton antiporter activity | 2 | 2 | 1.000000 | 1.000000 |
| 217 | GO:0015616 | DNA translocase activity | 2 | 2 | 1.000000 | 1.000000 |
| 218 | GO:0015662 | ATPase activity, coupled to transmembrane movement of ions, phosphorylative mechanism | 2 | 2 | 1.000000 | 1.000000 |
| 219 | GO:0015932 | nucleobase-containing compound transmembrane transporter activity | 2 | 2 | 1.000000 | 1.000000 |
| 220 | GO:0016278 | lysine N-methyltransferase activity | 2 | 2 | 1.000000 | 1.000000 |
| 221 | GO:0016279 | protein-lysine N-methyltransferase activity | 2 | 2 | 1.000000 | 1.000000 |
| 222 | GO:0016410 | N-acyltransferase activity | 2 | 2 | 1.000000 | 1.000000 |
| 223 | GO:0016641 | oxidoreductase activity, acting on the CH-NH2 group of donors, oxygen as acceptor | 2 | 2 | 1.000000 | 1.000000 |
| 224 | GO:0016655 | oxidoreductase activity, acting on NAD(P)H, quinone or similar compound as acceptor | 2 | 2 | 1.000000 | 1.000000 |
| 225 | GO:0016667 | oxidoreductase activity, acting on a sulfur group of donors | 2 | 2 | 1.000000 | 1.000000 |
| 226 | GO:0016668 | oxidoreductase activity, acting on a sulfur group of donors, NAD(P) as acceptor | 2 | 2 | 1.000000 | 1.000000 |
| 227 | GO:0016709 | oxidoreductase activity, acting on paired donors, with incorporation or reduction of molecular oxygen, NAD(P)H as one donor, and incorporation of one atom of oxygen | 2 | 2 | 1.000000 | 1.000000 |
| 228 | GO:0016763 | transferase activity, transferring pentosyl groups | 2 | 2 | 1.000000 | 1.000000 |
| 229 | GO:0016765 | transferase activity, transferring alkyl or aryl (other than methyl) groups | 2 | 2 | 1.000000 | 1.000000 |
| 230 | GO:0016782 | transferase activity, transferring sulfur-containing groups | 2 | 2 | 1.000000 | 1.000000 |
| 231 | GO:0016799 | hydrolase activity, hydrolyzing N-glycosyl compounds | 2 | 2 | 1.000000 | 1.000000 |
| 232 | GO:0016877 | ligase activity, forming carbon-sulfur bonds | 2 | 2 | 1.000000 | 1.000000 |
| 233 | GO:0016884 | carbon-nitrogen ligase activity, with glutamine as amido-N-donor | 2 | 2 | 1.000000 | 1.000000 |
| 234 | GO:0017025 | TBP-class protein binding | 2 | 2 | 1.000000 | 1.000000 |
| 235 | GO:0018024 | histone-lysine N-methyltransferase activity | 2 | 2 | 1.000000 | 1.000000 |
| 236 | GO:0019207 | kinase regulator activity | 2 | 2 | 1.000000 | 1.000000 |
| 237 | GO:0019783 | ubiquitin-like protein-specific protease activity | 2 | 2 | 1.000000 | 1.000000 |
| 238 | GO:0019829 | cation-transporting ATPase activity | 2 | 2 | 1.000000 | 1.000000 |
| 239 | GO:0019843 | rRNA binding | 2 | 2 | 1.000000 | 1.000000 |
| 240 | GO:0019887 | protein kinase regulator activity | 2 | 2 | 1.000000 | 1.000000 |
| 241 | GO:0022803 | passive transmembrane transporter activity | 2 | 2 | 1.000000 | 1.000000 |
| 242 | GO:0022832 | voltage-gated channel activity | 2 | 2 | 1.000000 | 1.000000 |
| 243 | GO:0022836 | gated channel activity | 2 | 2 | 1.000000 | 1.000000 |
| 244 | GO:0022838 | substrate-specific channel activity | 2 | 2 | 1.000000 | 1.000000 |
| 245 | GO:0030695 | GTPase regulator activity | 2 | 2 | 1.000000 | 1.000000 |
| 246 | GO:0031491 | nucleosome binding | 2 | 2 | 1.000000 | 1.000000 |
| 247 | GO:0031683 | G-protein beta/gamma-subunit complex binding | 2 | 2 | 1.000000 | 1.000000 |
| 248 | GO:0034061 | DNA polymerase activity | 2 | 2 | 1.000000 | 1.000000 |
| 249 | GO:0034062 | RNA polymerase activity | 2 | 2 | 1.000000 | 1.000000 |
| 250 | GO:0035091 | phosphatidylinositol binding | 2 | 2 | 1.000000 | 1.000000 |
| 251 | GO:0036442 | hydrogen-exporting ATPase activity | 2 | 2 | 1.000000 | 1.000000 |
| 252 | GO:0036459 | thiol-dependent ubiquitinyl hydrolase activity | 2 | 2 | 1.000000 | 1.000000 |
| 253 | GO:0042054 | histone methyltransferase activity | 2 | 2 | 1.000000 | 1.000000 |
| 254 | GO:0042625 | ATPase coupled ion transmembrane transporter activity | 2 | 2 | 1.000000 | 1.000000 |
| 255 | GO:0042803 | protein homodimerization activity | 2 | 2 | 1.000000 | 1.000000 |
| 256 | GO:0050136 | NADH dehydrogenase (quinone) activity | 2 | 2 | 1.000000 | 1.000000 |
| 257 | GO:0061630 | ubiquitin protein ligase activity | 2 | 2 | 1.000000 | 1.000000 |
| 258 | GO:0061659 | ubiquitin-like protein ligase activity | 2 | 2 | 1.000000 | 1.000000 |
| 259 | GO:0070001 | aspartic-type peptidase activity | 2 | 2 | 1.000000 | 1.000000 |
| 260 | GO:0070035 | purine NTP-dependent helicase activity | 2 | 2 | 1.000000 | 1.000000 |
| 261 | GO:0099516 | ion antiporter activity | 2 | 2 | 1.000000 | 1.000000 |
| 262 | GO:0101005 | ubiquitinyl hydrolase activity | 2 | 2 | 1.000000 | 1.000000 |
| 263 | GO:1901505 | carbohydrate derivative transporter activity | 2 | 2 | 1.000000 | 1.000000 |
| 264 | GO:1901677 | phosphate transmembrane transporter activity | 2 | 2 | 1.000000 | 1.000000 |
| 265 | GO:1990837 | sequence-specific double-stranded DNA binding | 2 | 2 | 1.000000 | 1.000000 |
| 266 | GO:0000030 | mannosyltransferase activity | 1 | 1 | 1.000000 | 1.000000 |
| 267 | GO:0000104 | succinate dehydrogenase activity | 1 | 1 | 1.000000 | 1.000000 |
| 268 | GO:0000175 | 3'-5'-exoribonuclease activity | 1 | 1 | 1.000000 | 1.000000 |
| 269 | GO:0000179 | rRNA (adenine-N6,N6-)-dimethyltransferase activity | 1 | 1 | 1.000000 | 1.000000 |
| 270 | GO:0000334 | 3-hydroxyanthranilate 3,4-dioxygenase activity | 1 | 1 | 1.000000 | 1.000000 |
| 271 | GO:0000339 | RNA cap binding | 1 | 1 | 1.000000 | 1.000000 |
| 272 | GO:0000774 | adenyl-nucleotide exchange factor activity | 1 | 1 | 1.000000 | 1.000000 |
| 273 | GO:0000822 | inositol hexakisphosphate binding | 1 | 1 | 1.000000 | 1.000000 |
| 274 | GO:0000975 | regulatory region DNA binding | 1 | 1 | 1.000000 | 1.000000 |
| 275 | GO:0000976 | transcription regulatory region sequence-specific DNA binding | 1 | 1 | 1.000000 | 1.000000 |
| 276 | GO:0000977 | RNA polymerase II regulatory region sequence-specific DNA binding | 1 | 1 | 1.000000 | 1.000000 |
| 277 | GO:0000978 | RNA polymerase II core promoter proximal region sequence-specific DNA binding | 1 | 1 | 1.000000 | 1.000000 |
| 278 | GO:0000987 | core promoter proximal region sequence-specific DNA binding | 1 | 1 | 1.000000 | 1.000000 |
| 279 | GO:0000988 | transcription factor activity, protein binding | 1 | 1 | 1.000000 | 1.000000 |
| 280 | GO:0000990 | transcription factor activity, core RNA polymerase binding | 1 | 1 | 1.000000 | 1.000000 |
| 281 | GO:0000991 | transcription factor activity, core RNA polymerase II binding | 1 | 1 | 1.000000 | 1.000000 |
| 282 | GO:0001012 | RNA polymerase II regulatory region DNA binding | 1 | 1 | 1.000000 | 1.000000 |
| 283 | GO:0001055 | RNA polymerase II activity | 1 | 1 | 1.000000 | 1.000000 |
| 284 | GO:0001067 | regulatory region nucleic acid binding | 1 | 1 | 1.000000 | 1.000000 |
| 285 | GO:0001139 | transcription factor activity, core RNA polymerase II recruiting | 1 | 1 | 1.000000 | 1.000000 |
| 286 | GO:0001159 | core promoter proximal region DNA binding | 1 | 1 | 1.000000 | 1.000000 |
| 287 | GO:0001228 | transcriptional activator activity, RNA polymerase II transcription regulatory region sequence-specific binding | 1 | 1 | 1.000000 | 1.000000 |
| 288 | GO:0001664 | G-protein coupled receptor binding | 1 | 1 | 1.000000 | 1.000000 |
| 289 | GO:0001871 | pattern binding | 1 | 1 | 1.000000 | 1.000000 |
| 290 | GO:0002094 | polyprenyltransferase activity | 1 | 1 | 1.000000 | 1.000000 |
| 291 | GO:0003688 | DNA replication origin binding | 1 | 1 | 1.000000 | 1.000000 |
| 292 | GO:0003697 | single-stranded DNA binding | 1 | 1 | 1.000000 | 1.000000 |
| 293 | GO:0003725 | double-stranded RNA binding | 1 | 1 | 1.000000 | 1.000000 |
| 294 | GO:0003727 | single-stranded RNA binding | 1 | 1 | 1.000000 | 1.000000 |
| 295 | GO:0003746 | translation elongation factor activity | 1 | 1 | 1.000000 | 1.000000 |
| 296 | GO:0003844 | 1,4-alpha-glucan branching enzyme activity | 1 | 1 | 1.000000 | 1.000000 |
| 297 | GO:0003855 | 3-dehydroquinate dehydratase activity | 1 | 1 | 1.000000 | 1.000000 |
| 298 | GO:0003856 | 3-dehydroquinate synthase activity | 1 | 1 | 1.000000 | 1.000000 |
| 299 | GO:0003862 | 3-isopropylmalate dehydrogenase activity | 1 | 1 | 1.000000 | 1.000000 |
| 300 | GO:0003866 | 3-phosphoshikimate 1-carboxyvinyltransferase activity | 1 | 1 | 1.000000 | 1.000000 |
| 301 | GO:0003870 | 5-aminolevulinate synthase activity | 1 | 1 | 1.000000 | 1.000000 |
| 302 | GO:0003873 | 6-phosphofructo-2-kinase activity | 1 | 1 | 1.000000 | 1.000000 |
| 303 | GO:0003883 | CTP synthase activity | 1 | 1 | 1.000000 | 1.000000 |
| 304 | GO:0003917 | DNA topoisomerase type I activity | 1 | 1 | 1.000000 | 1.000000 |
| 305 | GO:0003918 | DNA topoisomerase type II (ATP-hydrolyzing) activity | 1 | 1 | 1.000000 | 1.000000 |
| 306 | GO:0003951 | NAD+ kinase activity | 1 | 1 | 1.000000 | 1.000000 |
| 307 | GO:0003952 | NAD+ synthase (glutamine-hydrolyzing) activity | 1 | 1 | 1.000000 | 1.000000 |
| 308 | GO:0003968 | RNA-directed RNA polymerase activity | 1 | 1 | 1.000000 | 1.000000 |
| 309 | GO:0003992 | N2-acetyl-L-ornithine:2-oxoglutarate 5-aminotransferase activity | 1 | 1 | 1.000000 | 1.000000 |
| 310 | GO:0004040 | amidase activity | 1 | 1 | 1.000000 | 1.000000 |
| 311 | GO:0004042 | acetyl-CoA:L-glutamate N-acetyltransferase activity | 1 | 1 | 1.000000 | 1.000000 |
| 312 | GO:0004044 | amidophosphoribosyltransferase activity | 1 | 1 | 1.000000 | 1.000000 |
| 313 | GO:0004070 | aspartate carbamoyltransferase activity | 1 | 1 | 1.000000 | 1.000000 |
| 314 | GO:0004088 | carbamoyl-phosphate synthase (glutamine-hydrolyzing) activity | 1 | 1 | 1.000000 | 1.000000 |
| 315 | GO:0004089 | carbonate dehydratase activity | 1 | 1 | 1.000000 | 1.000000 |
| 316 | GO:0004100 | chitin synthase activity | 1 | 1 | 1.000000 | 1.000000 |
| 317 | GO:0004128 | cytochrome-b5 reductase activity, acting on NAD(P)H | 1 | 1 | 1.000000 | 1.000000 |
| 318 | GO:0004134 | 4-alpha-glucanotransferase activity | 1 | 1 | 1.000000 | 1.000000 |
| 319 | GO:0004135 | amylo-alpha-1,6-glucosidase activity | 1 | 1 | 1.000000 | 1.000000 |
| 320 | GO:0004164 | diphthine synthase activity | 1 | 1 | 1.000000 | 1.000000 |
| 321 | GO:0004169 | dolichyl-phosphate-mannose-protein mannosyltransferase activity | 1 | 1 | 1.000000 | 1.000000 |
| 322 | GO:0004312 | fatty acid synthase activity | 1 | 1 | 1.000000 | 1.000000 |
| 323 | GO:0004318 | enoyl-[acyl-carrier-protein] reductase (NADH) activity | 1 | 1 | 1.000000 | 1.000000 |
| 324 | GO:0004339 | glucan 1,4-alpha-glucosidase activity | 1 | 1 | 1.000000 | 1.000000 |
| 325 | GO:0004350 | glutamate-5-semialdehyde dehydrogenase activity | 1 | 1 | 1.000000 | 1.000000 |
| 326 | GO:0004355 | glutamate synthase (NADPH) activity | 1 | 1 | 1.000000 | 1.000000 |
| 327 | GO:0004358 | glutamate N-acetyltransferase activity | 1 | 1 | 1.000000 | 1.000000 |
| 328 | GO:0004367 | glycerol-3-phosphate dehydrogenase [NAD+] activity | 1 | 1 | 1.000000 | 1.000000 |
| 329 | GO:0004371 | glycerone kinase activity | 1 | 1 | 1.000000 | 1.000000 |
| 330 | GO:0004375 | glycine dehydrogenase (decarboxylating) activity | 1 | 1 | 1.000000 | 1.000000 |
| 331 | GO:0004399 | histidinol dehydrogenase activity | 1 | 1 | 1.000000 | 1.000000 |
| 332 | GO:0004448 | isocitrate dehydrogenase activity | 1 | 1 | 1.000000 | 1.000000 |
| 333 | GO:0004449 | isocitrate dehydrogenase (NAD+) activity | 1 | 1 | 1.000000 | 1.000000 |
| 334 | GO:0004455 | ketol-acid reductoisomerase activity | 1 | 1 | 1.000000 | 1.000000 |
| 335 | GO:0004476 | mannose-6-phosphate isomerase activity | 1 | 1 | 1.000000 | 1.000000 |
| 336 | GO:0004491 | methylmalonate-semialdehyde dehydrogenase (acylating) activity | 1 | 1 | 1.000000 | 1.000000 |
| 337 | GO:0004519 | endonuclease activity | 1 | 1 | 1.000000 | 1.000000 |
| 338 | GO:0004532 | exoribonuclease activity | 1 | 1 | 1.000000 | 1.000000 |
| 339 | GO:0004540 | ribonuclease activity | 1 | 1 | 1.000000 | 1.000000 |
| 340 | GO:0004555 | alpha,alpha-trehalase activity | 1 | 1 | 1.000000 | 1.000000 |
| 341 | GO:0004559 | alpha-mannosidase activity | 1 | 1 | 1.000000 | 1.000000 |
| 342 | GO:0004563 | beta-N-acetylhexosaminidase activity | 1 | 1 | 1.000000 | 1.000000 |
| 343 | GO:0004605 | phosphatidate cytidylyltransferase activity | 1 | 1 | 1.000000 | 1.000000 |
| 344 | GO:0004635 | phosphoribosyl-AMP cyclohydrolase activity | 1 | 1 | 1.000000 | 1.000000 |
| 345 | GO:0004636 | phosphoribosyl-ATP diphosphatase activity | 1 | 1 | 1.000000 | 1.000000 |
| 346 | GO:0004637 | phosphoribosylamine-glycine ligase activity | 1 | 1 | 1.000000 | 1.000000 |
| 347 | GO:0004641 | phosphoribosylformylglycinamidine cyclo-ligase activity | 1 | 1 | 1.000000 | 1.000000 |
| 348 | GO:0004650 | polygalacturonase activity | 1 | 1 | 1.000000 | 1.000000 |
| 349 | GO:0004659 | prenyltransferase activity | 1 | 1 | 1.000000 | 1.000000 |
| 350 | GO:0004683 | calmodulin-dependent protein kinase activity | 1 | 1 | 1.000000 | 1.000000 |
| 351 | GO:0004713 | protein tyrosine kinase activity | 1 | 1 | 1.000000 | 1.000000 |
| 352 | GO:0004722 | protein serine/threonine phosphatase activity | 1 | 1 | 1.000000 | 1.000000 |
| 353 | GO:0004724 | magnesium-dependent protein serine/threonine phosphatase activity | 1 | 1 | 1.000000 | 1.000000 |
| 354 | GO:0004731 | purine-nucleoside phosphorylase activity | 1 | 1 | 1.000000 | 1.000000 |
| 355 | GO:0004748 | ribonucleoside-diphosphate reductase activity, thioredoxin disulfide as acceptor | 1 | 1 | 1.000000 | 1.000000 |
| 356 | GO:0004749 | ribose phosphate diphosphokinase activity | 1 | 1 | 1.000000 | 1.000000 |
| 357 | GO:0004764 | shikimate 3-dehydrogenase (NADP+) activity | 1 | 1 | 1.000000 | 1.000000 |
| 358 | GO:0004765 | shikimate kinase activity | 1 | 1 | 1.000000 | 1.000000 |
| 359 | GO:0004768 | stearoyl-CoA 9-desaturase activity | 1 | 1 | 1.000000 | 1.000000 |
| 360 | GO:0004774 | succinate-CoA ligase activity | 1 | 1 | 1.000000 | 1.000000 |
| 361 | GO:0004775 | succinate-CoA ligase (ADP-forming) activity | 1 | 1 | 1.000000 | 1.000000 |
| 362 | GO:0004792 | thiosulfate sulfurtransferase activity | 1 | 1 | 1.000000 | 1.000000 |
| 363 | GO:0004801 | sedoheptulose-7-phosphate:D-glyceraldehyde-3-phosphate glyceronetransferase activity | 1 | 1 | 1.000000 | 1.000000 |
| 364 | GO:0004806 | triglyceride lipase activity | 1 | 1 | 1.000000 | 1.000000 |
| 365 | GO:0004813 | alanine-tRNA ligase activity | 1 | 1 | 1.000000 | 1.000000 |
| 366 | GO:0004826 | phenylalanine-tRNA ligase activity | 1 | 1 | 1.000000 | 1.000000 |
| 367 | GO:0004830 | tryptophan-tRNA ligase activity | 1 | 1 | 1.000000 | 1.000000 |
| 368 | GO:0004834 | tryptophan synthase activity | 1 | 1 | 1.000000 | 1.000000 |
| 369 | GO:0004843 | thiol-dependent ubiquitin-specific protease activity | 1 | 1 | 1.000000 | 1.000000 |
| 370 | GO:0004846 | urate oxidase activity | 1 | 1 | 1.000000 | 1.000000 |
| 371 | GO:0005102 | receptor binding | 1 | 1 | 1.000000 | 1.000000 |
| 372 | GO:0005200 | structural constituent of cytoskeleton | 1 | 1 | 1.000000 | 1.000000 |
| 373 | GO:0005247 | voltage-gated chloride channel activity | 1 | 1 | 1.000000 | 1.000000 |
| 374 | GO:0005254 | chloride channel activity | 1 | 1 | 1.000000 | 1.000000 |
| 375 | GO:0005315 | inorganic phosphate transmembrane transporter activity | 1 | 1 | 1.000000 | 1.000000 |
| 376 | GO:0005337 | nucleoside transmembrane transporter activity | 1 | 1 | 1.000000 | 1.000000 |
| 377 | GO:0005338 | nucleotide-sugar transmembrane transporter activity | 1 | 1 | 1.000000 | 1.000000 |
| 378 | GO:0005342 | organic acid transmembrane transporter activity | 1 | 1 | 1.000000 | 1.000000 |
| 379 | GO:0005371 | tricarboxylate secondary active transmembrane transporter activity | 1 | 1 | 1.000000 | 1.000000 |
| 380 | GO:0005384 | manganese ion transmembrane transporter activity | 1 | 1 | 1.000000 | 1.000000 |
| 381 | GO:0005451 | monovalent cation:proton antiporter activity | 1 | 1 | 1.000000 | 1.000000 |
| 382 | GO:0005452 | inorganic anion exchanger activity | 1 | 1 | 1.000000 | 1.000000 |
| 383 | GO:0005457 | GDP-fucose transmembrane transporter activity | 1 | 1 | 1.000000 | 1.000000 |
| 384 | GO:0005507 | copper ion binding | 1 | 1 | 1.000000 | 1.000000 |
| 385 | GO:0005509 | calcium ion binding | 1 | 1 | 1.000000 | 1.000000 |
| 386 | GO:0008080 | N-acetyltransferase activity | 1 | 1 | 1.000000 | 1.000000 |
| 387 | GO:0008171 | O-methyltransferase activity | 1 | 1 | 1.000000 | 1.000000 |
| 388 | GO:0008175 | tRNA methyltransferase activity | 1 | 1 | 1.000000 | 1.000000 |
| 389 | GO:0008186 | RNA-dependent ATPase activity | 1 | 1 | 1.000000 | 1.000000 |
| 390 | GO:0008198 | ferrous iron binding | 1 | 1 | 1.000000 | 1.000000 |
| 391 | GO:0008235 | metalloexopeptidase activity | 1 | 1 | 1.000000 | 1.000000 |
| 392 | GO:0008260 | 3-oxoacid CoA-transferase activity | 1 | 1 | 1.000000 | 1.000000 |
| 393 | GO:0008408 | 3'-5' exonuclease activity | 1 | 1 | 1.000000 | 1.000000 |
| 394 | GO:0008410 | CoA-transferase activity | 1 | 1 | 1.000000 | 1.000000 |
| 395 | GO:0008422 | beta-glucosidase activity | 1 | 1 | 1.000000 | 1.000000 |
| 396 | GO:0008443 | phosphofructokinase activity | 1 | 1 | 1.000000 | 1.000000 |
| 397 | GO:0008446 | GDP-mannose 4,6-dehydratase activity | 1 | 1 | 1.000000 | 1.000000 |
| 398 | GO:0008477 | purine nucleosidase activity | 1 | 1 | 1.000000 | 1.000000 |
| 399 | GO:0008514 | organic anion transmembrane transporter activity | 1 | 1 | 1.000000 | 1.000000 |
| 400 | GO:0008641 | small protein activating enzyme activity | 1 | 1 | 1.000000 | 1.000000 |
| 401 | GO:0008649 | rRNA methyltransferase activity | 1 | 1 | 1.000000 | 1.000000 |
| 402 | GO:0008863 | formate dehydrogenase (NAD+) activity | 1 | 1 | 1.000000 | 1.000000 |
| 403 | GO:0008897 | holo-[acyl-carrier-protein] synthase activity | 1 | 1 | 1.000000 | 1.000000 |
| 404 | GO:0008976 | polyphosphate kinase activity | 1 | 1 | 1.000000 | 1.000000 |
| 405 | GO:0009055 | electron carrier activity | 1 | 1 | 1.000000 | 1.000000 |
| 406 | GO:0009982 | pseudouridine synthase activity | 1 | 1 | 1.000000 | 1.000000 |
| 407 | GO:0010340 | carboxyl-O-methyltransferase activity | 1 | 1 | 1.000000 | 1.000000 |
| 408 | GO:0015079 | potassium ion transmembrane transporter activity | 1 | 1 | 1.000000 | 1.000000 |
| 409 | GO:0015081 | sodium ion transmembrane transporter activity | 1 | 1 | 1.000000 | 1.000000 |
| 410 | GO:0015108 | chloride transmembrane transporter activity | 1 | 1 | 1.000000 | 1.000000 |
| 411 | GO:0015109 | chromate transmembrane transporter activity | 1 | 1 | 1.000000 | 1.000000 |
| 412 | GO:0015142 | tricarboxylic acid transmembrane transporter activity | 1 | 1 | 1.000000 | 1.000000 |
| 413 | GO:0015215 | nucleotide transmembrane transporter activity | 1 | 1 | 1.000000 | 1.000000 |
| 414 | GO:0015293 | symporter activity | 1 | 1 | 1.000000 | 1.000000 |
| 415 | GO:0015294 | solute:cation symporter activity | 1 | 1 | 1.000000 | 1.000000 |
| 416 | GO:0015295 | solute:proton symporter activity | 1 | 1 | 1.000000 | 1.000000 |
| 417 | GO:0015301 | anion:anion antiporter activity | 1 | 1 | 1.000000 | 1.000000 |
| 418 | GO:0015385 | sodium:proton antiporter activity | 1 | 1 | 1.000000 | 1.000000 |
| 419 | GO:0015491 | cation:cation antiporter activity | 1 | 1 | 1.000000 | 1.000000 |
| 420 | GO:0015605 | organophosphate ester transmembrane transporter activity | 1 | 1 | 1.000000 | 1.000000 |
| 421 | GO:0015923 | mannosidase activity | 1 | 1 | 1.000000 | 1.000000 |
| 422 | GO:0015927 | trehalase activity | 1 | 1 | 1.000000 | 1.000000 |
| 423 | GO:0015929 | hexosaminidase activity | 1 | 1 | 1.000000 | 1.000000 |
| 424 | GO:0015930 | glutamate synthase activity | 1 | 1 | 1.000000 | 1.000000 |
| 425 | GO:0016040 | glutamate synthase (NADH) activity | 1 | 1 | 1.000000 | 1.000000 |
| 426 | GO:0016209 | antioxidant activity | 1 | 1 | 1.000000 | 1.000000 |
| 427 | GO:0016215 | acyl-CoA desaturase activity | 1 | 1 | 1.000000 | 1.000000 |
| 428 | GO:0016298 | lipase activity | 1 | 1 | 1.000000 | 1.000000 |
| 429 | GO:0016303 | 1-phosphatidylinositol-3-kinase activity | 1 | 1 | 1.000000 | 1.000000 |
| 430 | GO:0016405 | CoA-ligase activity | 1 | 1 | 1.000000 | 1.000000 |
| 431 | GO:0016407 | acetyltransferase activity | 1 | 1 | 1.000000 | 1.000000 |
| 432 | GO:0016409 | palmitoyltransferase activity | 1 | 1 | 1.000000 | 1.000000 |
| 433 | GO:0016417 | S-acyltransferase activity | 1 | 1 | 1.000000 | 1.000000 |
| 434 | GO:0016427 | tRNA (cytosine) methyltransferase activity | 1 | 1 | 1.000000 | 1.000000 |
| 435 | GO:0016433 | rRNA (adenine) methyltransferase activity | 1 | 1 | 1.000000 | 1.000000 |
| 436 | GO:0016504 | peptidase activator activity | 1 | 1 | 1.000000 | 1.000000 |
| 437 | GO:0016597 | amino acid binding | 1 | 1 | 1.000000 | 1.000000 |
| 438 | GO:0016628 | oxidoreductase activity, acting on the CH-CH group of donors, NAD or NADP as acceptor | 1 | 1 | 1.000000 | 1.000000 |
| 439 | GO:0016639 | oxidoreductase activity, acting on the CH-NH2 group of donors, NAD or NADP as acceptor | 1 | 1 | 1.000000 | 1.000000 |
| 440 | GO:0016642 | oxidoreductase activity, acting on the CH-NH2 group of donors, disulfide as acceptor | 1 | 1 | 1.000000 | 1.000000 |
| 441 | GO:0016653 | oxidoreductase activity, acting on NAD(P)H, heme protein as acceptor | 1 | 1 | 1.000000 | 1.000000 |
| 442 | GO:0016661 | oxidoreductase activity, acting on other nitrogenous compounds as donors | 1 | 1 | 1.000000 | 1.000000 |
| 443 | GO:0016663 | oxidoreductase activity, acting on other nitrogenous compounds as donors, oxygen as acceptor | 1 | 1 | 1.000000 | 1.000000 |
| 444 | GO:0016703 | oxidoreductase activity, acting on single donors with incorporation of molecular oxygen, incorporation of one atom of oxygen (internal monooxygenases or internal mixed function oxidases) | 1 | 1 | 1.000000 | 1.000000 |
| 445 | GO:0016717 | oxidoreductase activity, acting on paired donors, with oxidation of a pair of donors resulting in the reduction of molecular oxygen to two molecules of water | 1 | 1 | 1.000000 | 1.000000 |
| 446 | GO:0016725 | oxidoreductase activity, acting on CH or CH2 groups | 1 | 1 | 1.000000 | 1.000000 |
| 447 | GO:0016728 | oxidoreductase activity, acting on CH or CH2 groups, disulfide as acceptor | 1 | 1 | 1.000000 | 1.000000 |
| 448 | GO:0016743 | carboxyl- or carbamoyltransferase activity | 1 | 1 | 1.000000 | 1.000000 |
| 449 | GO:0016744 | transferase activity, transferring aldehyde or ketonic groups | 1 | 1 | 1.000000 | 1.000000 |
| 450 | GO:0016748 | succinyltransferase activity | 1 | 1 | 1.000000 | 1.000000 |
| 451 | GO:0016749 | N-succinyltransferase activity | 1 | 1 | 1.000000 | 1.000000 |
| 452 | GO:0016776 | phosphotransferase activity, phosphate group as acceptor | 1 | 1 | 1.000000 | 1.000000 |
| 453 | GO:0016778 | diphosphotransferase activity | 1 | 1 | 1.000000 | 1.000000 |
| 454 | GO:0016780 | phosphotransferase activity, for other substituted phosphate groups | 1 | 1 | 1.000000 | 1.000000 |
| 455 | GO:0016783 | sulfurtransferase activity | 1 | 1 | 1.000000 | 1.000000 |
| 456 | GO:0016796 | exonuclease activity, active with either ribo- or deoxyribonucleic acids and producing 5'-phosphomonoesters | 1 | 1 | 1.000000 | 1.000000 |
| 457 | GO:0016811 | hydrolase activity, acting on carbon-nitrogen (but not peptide) bonds, in linear amides | 1 | 1 | 1.000000 | 1.000000 |
| 458 | GO:0016814 | hydrolase activity, acting on carbon-nitrogen (but not peptide) bonds, in cyclic amidines | 1 | 1 | 1.000000 | 1.000000 |
| 459 | GO:0016830 | carbon-carbon lyase activity | 1 | 1 | 1.000000 | 1.000000 |
| 460 | GO:0016832 | aldehyde-lyase activity | 1 | 1 | 1.000000 | 1.000000 |
| 461 | GO:0016860 | intramolecular oxidoreductase activity | 1 | 1 | 1.000000 | 1.000000 |
| 462 | GO:0016861 | intramolecular oxidoreductase activity, interconverting aldoses and ketoses | 1 | 1 | 1.000000 | 1.000000 |
| 463 | GO:0016866 | intramolecular transferase activity | 1 | 1 | 1.000000 | 1.000000 |
| 464 | GO:0016878 | acid-thiol ligase activity | 1 | 1 | 1.000000 | 1.000000 |
| 465 | GO:0016882 | cyclo-ligase activity | 1 | 1 | 1.000000 | 1.000000 |
| 466 | GO:0016896 | exoribonuclease activity, producing 5'-phosphomonoesters | 1 | 1 | 1.000000 | 1.000000 |
| 467 | GO:0017057 | 6-phosphogluconolactonase activity | 1 | 1 | 1.000000 | 1.000000 |
| 468 | GO:0017176 | phosphatidylinositol N-acetylglucosaminyltransferase activity | 1 | 1 | 1.000000 | 1.000000 |
| 469 | GO:0018580 | nitronate monooxygenase activity | 1 | 1 | 1.000000 | 1.000000 |
| 470 | GO:0019200 | carbohydrate kinase activity | 1 | 1 | 1.000000 | 1.000000 |
| 471 | GO:0019238 | cyclohydrolase activity | 1 | 1 | 1.000000 | 1.000000 |
| 472 | GO:0019706 | protein-cysteine S-palmitoyltransferase activity | 1 | 1 | 1.000000 | 1.000000 |
| 473 | GO:0019707 | protein-cysteine S-acyltransferase activity | 1 | 1 | 1.000000 | 1.000000 |
| 474 | GO:0019842 | vitamin binding | 1 | 1 | 1.000000 | 1.000000 |
| 475 | GO:0019900 | kinase binding | 1 | 1 | 1.000000 | 1.000000 |
| 476 | GO:0019901 | protein kinase binding | 1 | 1 | 1.000000 | 1.000000 |
| 477 | GO:0030247 | polysaccharide binding | 1 | 1 | 1.000000 | 1.000000 |
| 478 | GO:0030276 | clathrin binding | 1 | 1 | 1.000000 | 1.000000 |
| 479 | GO:0030674 | protein binding, bridging | 1 | 1 | 1.000000 | 1.000000 |
| 480 | GO:0030976 | thiamine pyrophosphate binding | 1 | 1 | 1.000000 | 1.000000 |
| 481 | GO:0031406 | carboxylic acid binding | 1 | 1 | 1.000000 | 1.000000 |
| 482 | GO:0031625 | ubiquitin protein ligase binding | 1 | 1 | 1.000000 | 1.000000 |
| 483 | GO:0032131 | alkylated DNA binding | 1 | 1 | 1.000000 | 1.000000 |
| 484 | GO:0032132 | O6-alkylguanine-DNA binding | 1 | 1 | 1.000000 | 1.000000 |
| 485 | GO:0033592 | RNA strand annealing activity | 1 | 1 | 1.000000 | 1.000000 |
| 486 | GO:0035004 | phosphatidylinositol 3-kinase activity | 1 | 1 | 1.000000 | 1.000000 |
| 487 | GO:0036080 | purine nucleotide-sugar transmembrane transporter activity | 1 | 1 | 1.000000 | 1.000000 |
| 488 | GO:0042134 | rRNA primary transcript binding | 1 | 1 | 1.000000 | 1.000000 |
| 489 | GO:0042292 | URM1 activating enzyme activity | 1 | 1 | 1.000000 | 1.000000 |
| 490 | GO:0042393 | histone binding | 1 | 1 | 1.000000 | 1.000000 |
| 491 | GO:0042800 | histone methyltransferase activity (H3-K4 specific) | 1 | 1 | 1.000000 | 1.000000 |
| 492 | GO:0043023 | ribosomal large subunit binding | 1 | 1 | 1.000000 | 1.000000 |
| 493 | GO:0043177 | organic acid binding | 1 | 1 | 1.000000 | 1.000000 |
| 494 | GO:0043178 | alcohol binding | 1 | 1 | 1.000000 | 1.000000 |
| 495 | GO:0044212 | transcription regulatory region DNA binding | 1 | 1 | 1.000000 | 1.000000 |
| 496 | GO:0044389 | ubiquitin-like protein ligase binding | 1 | 1 | 1.000000 | 1.000000 |
| 497 | GO:0045140 | inositol phosphoceramide synthase activity | 1 | 1 | 1.000000 | 1.000000 |
| 498 | GO:0045181 | glutamate synthase activity, NAD(P)H as acceptor | 1 | 1 | 1.000000 | 1.000000 |
| 499 | GO:0045735 | nutrient reservoir activity | 1 | 1 | 1.000000 | 1.000000 |
| 500 | GO:0046912 | transferase activity, transferring acyl groups, acyl groups converted into alkyl on transfer | 1 | 1 | 1.000000 | 1.000000 |
| 501 | GO:0046915 | transition metal ion transmembrane transporter activity | 1 | 1 | 1.000000 | 1.000000 |
| 502 | GO:0046943 | carboxylic acid transmembrane transporter activity | 1 | 1 | 1.000000 | 1.000000 |
| 503 | GO:0047724 | inosine nucleosidase activity | 1 | 1 | 1.000000 | 1.000000 |
| 504 | GO:0050113 | inositol oxygenase activity | 1 | 1 | 1.000000 | 1.000000 |
| 505 | GO:0051082 | unfolded protein binding | 1 | 1 | 1.000000 | 1.000000 |
| 506 | GO:0051087 | chaperone binding | 1 | 1 | 1.000000 | 1.000000 |
| 507 | GO:0051499 | D-aminoacyl-tRNA deacylase activity | 1 | 1 | 1.000000 | 1.000000 |
| 508 | GO:0051500 | D-tyrosyl-tRNA(Tyr) deacylase activity | 1 | 1 | 1.000000 | 1.000000 |
| 509 | GO:0051537 | 2 iron, 2 sulfur cluster binding | 1 | 1 | 1.000000 | 1.000000 |
| 510 | GO:0051538 | 3 iron, 4 sulfur cluster binding | 1 | 1 | 1.000000 | 1.000000 |
| 511 | GO:0051998 | protein carboxyl O-methyltransferase activity | 1 | 1 | 1.000000 | 1.000000 |
| 512 | GO:0052742 | phosphatidylinositol kinase activity | 1 | 1 | 1.000000 | 1.000000 |
| 513 | GO:0060090 | binding, bridging | 1 | 1 | 1.000000 | 1.000000 |
| 514 | GO:0060590 | ATPase regulator activity | 1 | 1 | 1.000000 | 1.000000 |
| 515 | GO:0061134 | peptidase regulator activity | 1 | 1 | 1.000000 | 1.000000 |
| 516 | GO:0061505 | DNA topoisomerase II activity | 1 | 1 | 1.000000 | 1.000000 |
| 517 | GO:0061731 | ribonucleoside-diphosphate reductase activity | 1 | 1 | 1.000000 | 1.000000 |
| 518 | GO:0070006 | metalloaminopeptidase activity | 1 | 1 | 1.000000 | 1.000000 |
| 519 | GO:0070403 | NAD+ binding | 1 | 1 | 1.000000 | 1.000000 |
| 520 | GO:0070566 | adenylyltransferase activity | 1 | 1 | 1.000000 | 1.000000 |
| 521 | GO:0070567 | cytidylyltransferase activity | 1 | 1 | 1.000000 | 1.000000 |
| 522 | GO:0070577 | lysine-acetylated histone binding | 1 | 1 | 1.000000 | 1.000000 |
| 523 | GO:0070628 | proteasome binding | 1 | 1 | 1.000000 | 1.000000 |
| 524 | GO:0070635 | nicotinamide riboside hydrolase activity | 1 | 1 | 1.000000 | 1.000000 |
| 525 | GO:0070733 | protein adenylyltransferase activity | 1 | 1 | 1.000000 | 1.000000 |
| 526 | GO:0070840 | dynein complex binding | 1 | 1 | 1.000000 | 1.000000 |
| 527 | GO:0090599 | alpha-glucosidase activity | 1 | 1 | 1.000000 | 1.000000 |
| 528 | GO:0097079 | selenite:proton symporter activity | 1 | 1 | 1.000000 | 1.000000 |
| 529 | GO:0097617 | annealing activity | 1 | 1 | 1.000000 | 1.000000 |
| 530 | GO:0098808 | mRNA cap binding | 1 | 1 | 1.000000 | 1.000000 |
| 531 | GO:1901681 | sulfur compound binding | 1 | 1 | 1.000000 | 1.000000 |
| 532 | GO:1990380 | Lys48-specific deubiquitinase activity | 1 | 1 | 1.000000 | 1.000000 |
| 533 | GO:2001070 | starch binding | 1 | 1 | 1.000000 | 1.000000 |

  

---

out GO Enrichment (Molecular Function) Gene Details

| # | GO ID | geneID |
| 1 | GO:0003824 | g7709 g10811 g15889 g1347 g5719 g4623 g9478 g4989 g2857 g2579 g16023 g15455 g5755 g7134 g3128 g12412 g12080 g10901 g7777 g5593 g15207 g7453 g3988 g1559 g2697 g6194 g3416 g9793 g9745 g9261 g8973 g7590 g7095 g5786 g5462 g5344 g4041 g3911 g3037 g2603 g2491 g2186 g16276 g16075 g15244 g1438 g11145 g3987 g13424 g10915 g7614 g7267 g5191 g4829 g4821 g4337 g4211 g3429 g3374 g16296 g1577 g15739 g12710 g1234 g12130 g11270 g10182 g5678 g16006 g380 g13644 g5023 g2380 g7594 g14291 g278 g1636 g1160 g5849 g14321 g15379 g8896 g15713 g218 g7388 g7203 g11549 g2194 g1333 g4762 g5803 g7416 g7749 g2764 g10152 g6334 g6579 g15457 g8806 g918 g2888 g15829 g11330 g4571 g9166 g6286 g5886 g174 g1715 g12204 g11016 g470 g16122 g6012 g7757 g5674 g12146 g8010 g9820 g2454 g1994 g1541 g15134 g1635 g1233 g8810 g6125 g2571 g2313 g13276 g4637 g672 g821 g5806 g3498 g4286 g15420 g1110 g8245 g11984 g11950 g760 g3115 g1539 g450 g13590 g10839 g9823 g868 g867 g8550 g7931 g5833 g5634 g4665 g2855 g2807 g244 g2399 g239 g2139 g207 g16213 g16211 g15680 g15673 g15609 g15485 g15123 g14466 g1164 g11476 g1138 g11240 g10489 g10488 g10485 g10204 g1008 g8625 g8736 g16101 g3274 g15480 g7440 g12113 g9946 g9211 g8679 g8527 g7810 g7564 g6593 g5424 g5393 g5024 g4792 g4374 g4193 g4116 g3949 g3663 g2953 g2610 g2334 g2253 g1632 g1468 g13911 g1326 g12928 g12680 g12048 g11649 g11418 g11222 g1119 g10953 g1046 g9578 g4773 g10820 g15421 g2152 g15513 g10256 g504 g4643 g3945 g13874 g13864 g4580 g4506 g12258 g9743 g7711 g4311 g2229 g1571 g1467 g12159 g8648 g475 g4023 g2852 g15636 g10823 g7307 g1884 g2353 g4289 g4890 g2488 g11041 g4435 g2509 g5346 g1374 g3457 g11042 g9209 g5922 g2268 g15316 g8631 g15891 g7249 g15835 g4365 g842 g4165 g3291 g2770 g6095 g5633 g7932 g4970 g15452 g3710 g7264 g6247 g4910 g3076 g7449 g5903 g13151 g14381 g4061 g9280 g8682 g7729 g6919 g6342 g6222 g5743 g5693 g5469 g4684 g4305 g1832 g15854 g12422 g11948 g11946 g11137 g10348 g16055 g8112 g13321 g1203 g1768 g11142 g1685 g2596 g6281 g11148 g6599 g15737 g3178 g10711 g10143 g8930 g2971 g13019 g2983 g5810 g1562 g15282 g15549 g11568 g12037 g6254 g7643 g7432 g3810 g2737 g11554 g1598 g7873 g8536 g9271 g2520 g5636 g5001 g4031 g13028 g5515 g502 g904 g12200 g15156 g6410 g602 g5980 g5694 g562 g5564 g4232 g3034 g2357 g11098 |
| 2 | GO:0005488 | g1008 g2477 g11042 g1635 g10811 g8679 g7564 g7432 g5932 g5922 g5719 g5393 g4671 g4319 g4217 g4094 g3810 g3697 g3128 g2807 g2488 g2464 g221 g16122 g16063 g16055 g16 g15889 g15316 g795 g3788 g1559 g5346 g3857 g278 g1636 g2663 g6194 g7729 g10182 g7449 g5903 g7249 g4571 g1685 g15104 g4832 g7073 g7709 g16161 g14774 g1233 g7908 g7873 g746 g7395 g7380 g7295 g6342 g6125 g5912 g5001 g323 g2571 g2513 g2313 g2268 g1774 g15358 g1468 g1377 g12225 g15421 g4910 g2983 g5803 g5515 g4643 g9820 g9797 g8545 g7931 g7134 g6579 g504 g4762 g4421 g2855 g2509 g2322 g2139 g207 g2060 g16006 g15356 g15134 g14466 g1164 g11476 g1138 g11240 g11221 g10711 g1884 g12422 g10839 g948 g9166 g892 g8810 g7811 g7453 g7267 g6919 g6502 g5742 g5567 g5344 g5253 g2913 g2847 g239 g2105 g16213 g16211 g15673 g1254 g11946 g11549 g10489 g10488 g6814 g6710 g15714 g8930 g15457 g2399 g918 g7307 g4305 g3988 g3987 g2888 g2194 g891 g8536 g7932 g6281 g6254 g4289 g4061 g319 g3076 g2199 g1333 g13222 g7300 g475 g4057 g1057 g4833 g5674 g1048 g4482 g12620 g4623 g2682 g7518 g8666 g4821 g4720 g8245 g11984 g11950 g4580 g380 g3034 g15455 g13644 g11145 g450 g9010 g4968 g2260 g11986 g9081 g759 g1225 g12573 g904 g2556 g7185 g2883 g4665 g3274 g15480 g14381 g5023 g2380 g1598 g1572 g15536 g13320 g3374 g9478 g4989 g2857 g2579 g16023 g8942 g12991 g6095 g4286 g8806 g5634 g4653 g3115 g2596 g5755 g15156 g11554 g11041 g9743 g861 g7749 g7707 g672 g6410 g637 g6291 g6012 g5980 g5694 g562 g5564 g4792 g4773 g4232 g3949 g2610 g2357 g2229 g16101 g15829 g1571 g1467 g14321 g13321 g12928 g12412 g12146 g12080 g1203 g1119 g1118 g11098 g10901 g14291 g218 g13590 g7973 g10143 g1110 g10700 g8648 g10823 g7282 g470 g9832 g9578 g7711 g2253 g1832 g13276 g13019 g6593 g6191 g4023 g2852 g15636 g11568 g5849 |
| 3 | GO:0097159 | g11042 g1635 g10811 g8679 g7564 g7432 g5932 g5922 g5719 g5393 g4671 g4319 g4217 g4094 g3810 g3697 g3128 g2807 g2488 g2464 g221 g16122 g16063 g16055 g16 g15889 g15316 g1559 g5346 g3857 g278 g1636 g7449 g5903 g7249 g4832 g7073 g7709 g16161 g14774 g1233 g7908 g7873 g746 g7395 g7380 g7295 g6342 g6125 g5912 g5001 g323 g2571 g2513 g2313 g2268 g1774 g15358 g1468 g1377 g12225 g15421 g918 g7307 g4305 g3988 g3987 g2888 g2194 g891 g8536 g7932 g6281 g6254 g4289 g4061 g319 g3076 g2199 g1333 g13222 g7300 g1057 g4833 g5674 g2682 g7518 g795 g12573 g9081 g904 g759 g3788 g2556 g4665 g3274 g15480 g3374 g5515 g9478 g4989 g2857 g2579 g16023 g15134 g10711 g6095 g5803 g5755 g4286 g15156 g11554 g11041 g9820 g9743 g8930 g861 g7749 g7707 g7134 g672 g6410 g637 g6291 g6012 g5980 g5694 g562 g5564 g5023 g4792 g4773 g4762 g4232 g3949 g3115 g3034 g2610 g2509 g2380 g2357 g2229 g16101 g16006 g15829 g1571 g1467 g14321 g13321 g12928 g12412 g12146 g12080 g1203 g1119 g1118 g11098 g10901 g218 g15714 g13590 g7973 g6194 g7729 g10182 g7282 g4910 g470 g9832 g9578 g7711 g2253 g1884 g1832 g13276 g13019 g8648 g6593 g6191 g475 g4023 g2852 g15636 g10823 g4580 g5849 g14381 |
| 4 | GO:1901363 | g11042 g1635 g10811 g8679 g7564 g7432 g5932 g5922 g5719 g5393 g4671 g4319 g4217 g4094 g3810 g3697 g3128 g2807 g2488 g2464 g221 g16122 g16063 g16055 g16 g15889 g15316 g1559 g5346 g3857 g278 g1636 g6194 g7729 g10182 g7449 g5903 g7249 g4832 g7073 g7709 g16161 g14774 g1233 g7908 g7873 g746 g7395 g7380 g7295 g6342 g6125 g5912 g5001 g323 g2571 g2513 g2313 g2268 g1774 g15358 g1468 g1377 g12225 g15421 g918 g7307 g4305 g3988 g3987 g2888 g2194 g891 g8536 g7932 g6281 g6254 g4289 g4061 g319 g3076 g2199 g1333 g13222 g7300 g1057 g4833 g5674 g2682 g7518 g795 g12573 g9081 g904 g759 g3788 g2556 g4665 g3274 g15480 g3374 g5515 g9478 g4989 g2857 g2579 g16023 g15134 g10711 g6095 g5803 g5755 g4286 g15156 g11554 g11041 g9820 g9743 g8930 g861 g7749 g7707 g7134 g672 g6410 g637 g6291 g6012 g5980 g5694 g562 g5564 g5023 g4792 g4773 g4762 g4232 g3949 g3115 g3034 g2610 g2509 g2380 g2357 g2229 g16101 g16006 g15829 g1571 g1467 g14321 g13321 g12928 g12412 g12146 g12080 g1203 g1119 g1118 g11098 g10901 g218 g15714 g13590 g7973 g7282 g4910 g470 g9832 g9578 g7711 g2253 g1884 g1832 g13276 g13019 g8648 g6593 g6191 g475 g4023 g2852 g15636 g10823 g4580 g5849 g14381 |
| 5 | GO:0043167 | g1559 g5346 g3857 g278 g1636 g7073 g2477 g5803 g5515 g4643 g9820 g9797 g8545 g7931 g7432 g7134 g6579 g504 g4762 g4671 g4421 g3788 g3128 g2983 g2855 g2509 g2322 g2139 g207 g2060 g1685 g16006 g15356 g15134 g14466 g1164 g11476 g1138 g11240 g11221 g10711 g1008 g1884 g2399 g918 g7307 g4305 g3988 g3987 g2888 g4623 g8666 g4821 g4720 g10182 g8245 g11984 g11950 g4580 g380 g4289 g4061 g3034 g15455 g13644 g11145 g450 g7185 g2883 g4665 g3274 g2807 g15480 g4833 g6095 g4286 g1233 g8806 g7873 g7564 g7395 g7295 g5932 g5634 g4653 g3810 g3115 g2596 g2268 g221 g16063 g16 g7709 g5755 g2194 g1635 g15156 g11554 g11041 g10811 g9743 g8930 g8679 g861 g7749 g7707 g672 g6410 g637 g6291 g6125 g6012 g5980 g5922 g5694 g5674 g562 g5564 g5393 g5023 g4792 g4773 g4232 g3949 g2610 g2571 g2380 g2357 g2313 g2229 g16122 g16101 g15889 g15829 g1571 g15316 g1468 g1467 g14321 g1333 g13321 g12928 g12412 g12146 g12080 g1203 g1119 g1118 g11098 g10901 g4571 g9478 g4989 g2857 g2579 g16023 g14291 g7449 g5903 g13590 g7973 g10143 g15457 g8648 g6593 g6191 g475 g4023 g2852 g15636 g10823 |
| 6 | GO:0016787 | g7709 g10811 g15889 g1347 g7777 g5593 g15207 g2697 g5023 g2380 g218 g8896 g2194 g1333 g5803 g2764 g10152 g7594 g6579 g11330 g4571 g9166 g6286 g5886 g4623 g174 g1715 g12204 g11016 g470 g16122 g7757 g3911 g5674 g12146 g1635 g1233 g8810 g6125 g2571 g2313 g13276 g672 g3115 g1539 g7440 g12113 g9946 g9211 g8679 g8527 g7810 g760 g7564 g6593 g5424 g5393 g5024 g4792 g4374 g4193 g4116 g3949 g3663 g2953 g2610 g2334 g2253 g1632 g1468 g13911 g1326 g12928 g12680 g12048 g11649 g11418 g11222 g1119 g10953 g1046 g9578 g4773 g10820 g15421 g4643 g3945 g13874 g13864 g4506 g5755 g8648 g475 g4023 g2852 g15636 g10823 g4289 g4286 g9209 g5922 g2268 g15316 g15891 g4365 g7932 g4970 g13151 g8112 g13321 g1203 g1685 g6599 g15737 g11549 g13019 g2983 g1562 g15282 g15549 g11568 g6254 g7643 g2737 g15156 g6410 g602 g5980 g5694 g562 g5564 g4232 g3034 g2357 g11098 |
| 7 | GO:0016740 | g4623 g15455 g5755 g7134 g3128 g12412 g12080 g10901 g3988 g6194 g3416 g9793 g9745 g9261 g8973 g7590 g7095 g5786 g5462 g5344 g4041 g3911 g3037 g2603 g2491 g2186 g16276 g16075 g15244 g1438 g11145 g3987 g13424 g10915 g7614 g7267 g5191 g4829 g4821 g4337 g4211 g3429 g3374 g16296 g1577 g15739 g12710 g1234 g12130 g11270 g10182 g5678 g16006 g7594 g1160 g14321 g15379 g8896 g7203 g7416 g6334 g15457 g918 g2888 g15829 g8010 g821 g3498 g760 g8625 g8736 g16101 g504 g5803 g12258 g9743 g7711 g4311 g2610 g2229 g1571 g1467 g12159 g7307 g2353 g4890 g2488 g4435 g3457 g11042 g842 g4165 g3291 g5633 g15452 g3710 g7264 g6247 g16055 g5023 g1768 g11142 g6281 g11148 g8930 g2971 g12037 g7432 g3810 g9820 g1598 g8536 g4762 g904 g12200 |
| 8 | GO:0036094 | g1559 g5346 g3857 g278 g1636 g7449 g5903 g7249 g7073 g7432 g2488 g795 g4665 g3274 g2807 g15480 g7709 g6095 g5803 g5755 g4286 g2194 g1635 g15156 g1233 g11554 g11041 g10811 g9820 g9743 g8930 g8679 g861 g7749 g7707 g7134 g672 g6410 g637 g6291 g6125 g6012 g5980 g5922 g5694 g5674 g562 g5564 g5393 g5023 g4792 g4773 g4762 g4232 g4061 g3949 g3128 g3115 g3034 g2610 g2571 g2509 g2380 g2357 g2313 g2268 g2229 g16122 g16101 g16006 g15889 g15829 g1571 g15316 g1468 g1467 g14321 g1333 g13321 g12928 g12412 g12146 g12080 g1203 g1119 g1118 g11098 g10901 g13590 g7973 g4910 g470 g9832 g9578 g7711 g3810 g2253 g1884 g1832 g13276 g13019 g15457 g8648 g6593 g6191 g475 g4023 g2852 g15636 g10823 g4580 g5849 g4289 g14381 |
| 9 | GO:0000166 | g1559 g5346 g3857 g278 g1636 g7449 g5903 g7249 g7073 g795 g4665 g3274 g2807 g15480 g7709 g6095 g5803 g5755 g4286 g2194 g1635 g15156 g1233 g11554 g11041 g10811 g9820 g9743 g8930 g8679 g861 g7749 g7707 g7134 g672 g6410 g637 g6291 g6125 g6012 g5980 g5922 g5694 g5674 g562 g5564 g5393 g5023 g4792 g4773 g4762 g4232 g4061 g3949 g3128 g3115 g3034 g2610 g2571 g2509 g2380 g2357 g2313 g2268 g2229 g16122 g16101 g16006 g15889 g15829 g1571 g15316 g1468 g1467 g14321 g1333 g13321 g12928 g12412 g12146 g12080 g1203 g1119 g1118 g11098 g10901 g13590 g7973 g4910 g470 g9832 g9578 g7711 g7432 g3810 g2253 g1884 g1832 g13276 g13019 g8648 g6593 g6191 g475 g4023 g2852 g15636 g10823 g4580 g5849 g4289 g14381 |
| 10 | GO:1901265 | g1559 g5346 g3857 g278 g1636 g7449 g5903 g7249 g7073 g795 g4665 g3274 g2807 g15480 g7709 g6095 g5803 g5755 g4286 g2194 g1635 g15156 g1233 g11554 g11041 g10811 g9820 g9743 g8930 g8679 g861 g7749 g7707 g7134 g672 g6410 g637 g6291 g6125 g6012 g5980 g5922 g5694 g5674 g562 g5564 g5393 g5023 g4792 g4773 g4762 g4232 g4061 g3949 g3128 g3115 g3034 g2610 g2571 g2509 g2380 g2357 g2313 g2268 g2229 g16122 g16101 g16006 g15889 g15829 g1571 g15316 g1468 g1467 g14321 g1333 g13321 g12928 g12412 g12146 g12080 g1203 g1119 g1118 g11098 g10901 g13590 g7973 g4910 g470 g9832 g9578 g7711 g7432 g3810 g2253 g1884 g1832 g13276 g13019 g8648 g6593 g6191 g475 g4023 g2852 g15636 g10823 g4580 g5849 g4289 g14381 |
| 11 | GO:0043168 | g1559 g5346 g3857 g278 g1636 g7073 g2477 g1884 g918 g7307 g4305 g3988 g3987 g2888 g7185 g2883 g4665 g3274 g2807 g15480 g7709 g6095 g5803 g5755 g4286 g2194 g1635 g15156 g1233 g11554 g11041 g10811 g9820 g9743 g8930 g8679 g861 g7749 g7707 g7134 g672 g6410 g637 g6291 g6125 g6012 g5980 g5922 g5694 g5674 g562 g5564 g5393 g5023 g4792 g4773 g4762 g4232 g4061 g3949 g3128 g3115 g3034 g2610 g2571 g2509 g2380 g2357 g2313 g2268 g2229 g16122 g16101 g16006 g15889 g15829 g1571 g15316 g1468 g1467 g14321 g1333 g13321 g12928 g12412 g12146 g12080 g1203 g1119 g1118 g11098 g10901 g7449 g5903 g13590 g7973 g15457 g8648 g6593 g6191 g475 g4023 g2852 g15636 g10823 |
| 12 | GO:0097367 | g7432 g2488 g7709 g6095 g5803 g5755 g4286 g2194 g1635 g15156 g1233 g11554 g11041 g10811 g9820 g9743 g8930 g8679 g861 g7749 g7707 g7134 g672 g6410 g637 g6291 g6125 g6012 g5980 g5922 g5694 g5674 g562 g5564 g5393 g5023 g4792 g4773 g4762 g4232 g4061 g3949 g3128 g3115 g3034 g2610 g2571 g2509 g2380 g2357 g2313 g2268 g2229 g16122 g16101 g16006 g15889 g15829 g1571 g15316 g1468 g1467 g14321 g1333 g13321 g12928 g12412 g12146 g12080 g1203 g1119 g1118 g11098 g10901 g1559 g7449 g5903 g13590 g7973 g8648 g6593 g6191 g475 g4023 g2852 g15636 g10823 |
| 13 | GO:0032553 | g7709 g6095 g5803 g5755 g4286 g2194 g1635 g15156 g1233 g11554 g11041 g10811 g9820 g9743 g8930 g8679 g861 g7749 g7707 g7134 g672 g6410 g637 g6291 g6125 g6012 g5980 g5922 g5694 g5674 g562 g5564 g5393 g5023 g4792 g4773 g4762 g4232 g4061 g3949 g3128 g3115 g3034 g2610 g2571 g2509 g2380 g2357 g2313 g2268 g2229 g16122 g16101 g16006 g15889 g15829 g1571 g15316 g1468 g1467 g14321 g1333 g13321 g12928 g12412 g12146 g12080 g1203 g1119 g1118 g11098 g10901 g1559 g7449 g5903 g13590 g7973 g8648 g6593 g6191 g475 g4023 g2852 g15636 g10823 |
| 14 | GO:0003676 | g11042 g1635 g10811 g8679 g7564 g7432 g5932 g5922 g5719 g5393 g4671 g4319 g4217 g4094 g3810 g3697 g3128 g2807 g2488 g2464 g221 g16122 g16063 g16055 g16 g15889 g15316 g4832 g7709 g16161 g14774 g1233 g7908 g7873 g746 g7395 g7380 g7295 g6342 g6125 g5912 g5001 g323 g2571 g2513 g2313 g2268 g1774 g15358 g1468 g1377 g12225 g15421 g2194 g891 g8536 g7932 g6281 g6254 g4305 g4289 g4061 g319 g3076 g2199 g1333 g13222 g7300 g1057 g4833 g5674 g2682 g7518 g12573 g9081 g904 g759 g3788 g2556 g3374 g6095 g218 g15714 g7282 |
| 15 | GO:0016491 | g9478 g4989 g2857 g2579 g16023 g15455 g1559 g14291 g278 g1636 g5849 g7388 g2454 g1994 g1541 g15134 g5806 g4286 g15420 g1110 g4571 g8245 g11984 g11950 g9820 g450 g13590 g10839 g9823 g868 g867 g8550 g7931 g5833 g5634 g4665 g4637 g2855 g2807 g244 g2399 g239 g2139 g207 g16213 g16211 g15680 g15673 g15609 g15485 g15123 g14466 g1164 g11476 g1138 g11270 g11240 g10489 g10488 g10485 g10204 g1008 g3274 g15480 g4580 g1884 g11418 g5346 g1374 g7249 g2770 g3076 g7449 g5903 g14381 g3178 g10711 g10143 g4289 g5810 g11554 g7873 g5515 |
| 16 | GO:0001882 | g7432 g2488 g7709 g6095 g5803 g5755 g4286 g2194 g1635 g15156 g1233 g11554 g11041 g10811 g9820 g9743 g8930 g8679 g861 g7749 g7707 g7134 g672 g6410 g637 g6291 g6125 g6012 g5980 g5922 g5694 g5674 g562 g5564 g5393 g5023 g4792 g4773 g4762 g4232 g4061 g3949 g3128 g3115 g3034 g2610 g2571 g2509 g2380 g2357 g2313 g2268 g2229 g16122 g16101 g16006 g15889 g15829 g1571 g15316 g1468 g1467 g14321 g1333 g13321 g12928 g12412 g12146 g12080 g1203 g1119 g1118 g11098 g10901 g8648 g6593 g6191 g475 g4023 g2852 g15636 g10823 |
| 17 | GO:0017076 | g795 g7709 g6095 g5803 g5755 g4286 g2194 g1635 g15156 g1233 g11554 g11041 g10811 g9820 g9743 g8930 g8679 g861 g7749 g7707 g7134 g672 g6410 g637 g6291 g6125 g6012 g5980 g5922 g5694 g5674 g562 g5564 g5393 g5023 g4792 g4773 g4762 g4232 g4061 g3949 g3128 g3115 g3034 g2610 g2571 g2509 g2380 g2357 g2313 g2268 g2229 g16122 g16101 g16006 g15889 g15829 g1571 g15316 g1468 g1467 g14321 g1333 g13321 g12928 g12412 g12146 g12080 g1203 g1119 g1118 g11098 g10901 g8648 g6593 g6191 g475 g4023 g2852 g15636 g10823 |
| 18 | GO:0032549 | g2488 g7709 g6095 g5803 g5755 g4286 g2194 g1635 g15156 g1233 g11554 g11041 g10811 g9820 g9743 g8930 g8679 g861 g7749 g7707 g7134 g672 g6410 g637 g6291 g6125 g6012 g5980 g5922 g5694 g5674 g562 g5564 g5393 g5023 g4792 g4773 g4762 g4232 g4061 g3949 g3128 g3115 g3034 g2610 g2571 g2509 g2380 g2357 g2313 g2268 g2229 g16122 g16101 g16006 g15889 g15829 g1571 g15316 g1468 g1467 g14321 g1333 g13321 g12928 g12412 g12146 g12080 g1203 g1119 g1118 g11098 g10901 g8648 g6593 g6191 g475 g4023 g2852 g15636 g10823 |
| 19 | GO:0001883 | g7709 g6095 g5803 g5755 g4286 g2194 g1635 g15156 g1233 g11554 g11041 g10811 g9820 g9743 g8930 g8679 g861 g7749 g7707 g7134 g672 g6410 g637 g6291 g6125 g6012 g5980 g5922 g5694 g5674 g562 g5564 g5393 g5023 g4792 g4773 g4762 g4232 g4061 g3949 g3128 g3115 g3034 g2610 g2571 g2509 g2380 g2357 g2313 g2268 g2229 g16122 g16101 g16006 g15889 g15829 g1571 g15316 g1468 g1467 g14321 g1333 g13321 g12928 g12412 g12146 g12080 g1203 g1119 g1118 g11098 g10901 g8648 g6593 g6191 g475 g4023 g2852 g15636 g10823 |
| 20 | GO:0032550 | g7709 g6095 g5803 g5755 g4286 g2194 g1635 g15156 g1233 g11554 g11041 g10811 g9820 g9743 g8930 g8679 g861 g7749 g7707 g7134 g672 g6410 g637 g6291 g6125 g6012 g5980 g5922 g5694 g5674 g562 g5564 g5393 g5023 g4792 g4773 g4762 g4232 g4061 g3949 g3128 g3115 g3034 g2610 g2571 g2509 g2380 g2357 g2313 g2268 g2229 g16122 g16101 g16006 g15889 g15829 g1571 g15316 g1468 g1467 g14321 g1333 g13321 g12928 g12412 g12146 g12080 g1203 g1119 g1118 g11098 g10901 g8648 g6593 g6191 g475 g4023 g2852 g15636 g10823 |
| 21 | GO:0032555 | g7709 g6095 g5803 g5755 g4286 g2194 g1635 g15156 g1233 g11554 g11041 g10811 g9820 g9743 g8930 g8679 g861 g7749 g7707 g7134 g672 g6410 g637 g6291 g6125 g6012 g5980 g5922 g5694 g5674 g562 g5564 g5393 g5023 g4792 g4773 g4762 g4232 g4061 g3949 g3128 g3115 g3034 g2610 g2571 g2509 g2380 g2357 g2313 g2268 g2229 g16122 g16101 g16006 g15889 g15829 g1571 g15316 g1468 g1467 g14321 g1333 g13321 g12928 g12412 g12146 g12080 g1203 g1119 g1118 g11098 g10901 g8648 g6593 g6191 g475 g4023 g2852 g15636 g10823 |
| 22 | GO:0035639 | g7709 g6095 g5803 g5755 g4286 g2194 g1635 g15156 g1233 g11554 g11041 g10811 g9820 g9743 g8930 g8679 g861 g7749 g7707 g7134 g672 g6410 g637 g6291 g6125 g6012 g5980 g5922 g5694 g5674 g562 g5564 g5393 g5023 g4792 g4773 g4762 g4232 g4061 g3949 g3128 g3115 g3034 g2610 g2571 g2509 g2380 g2357 g2313 g2268 g2229 g16122 g16101 g16006 g15889 g15829 g1571 g15316 g1468 g1467 g14321 g1333 g13321 g12928 g12412 g12146 g12080 g1203 g1119 g1118 g11098 g10901 g8648 g6593 g6191 g475 g4023 g2852 g15636 g10823 |
| 23 | GO:0043169 | g5803 g5515 g4643 g9820 g9797 g8545 g7931 g7432 g7134 g6579 g504 g4762 g4671 g4421 g3857 g3788 g3128 g2983 g2855 g2509 g2322 g2139 g207 g2060 g1685 g16006 g15356 g15134 g14466 g1164 g11476 g1138 g11240 g11221 g10711 g1008 g2399 g4623 g8245 g11984 g11950 g4580 g380 g4289 g4061 g3034 g15455 g13644 g11145 g450 g4833 g6095 g4286 g1233 g918 g8806 g7873 g7564 g7395 g7295 g5932 g5634 g4653 g3810 g3115 g2596 g2268 g221 g16063 g16 g1559 g4571 g9478 g4989 g2857 g2579 g16023 g14291 g10143 |
| 24 | GO:0046872 | g5803 g5515 g4643 g9820 g9797 g8545 g7931 g7432 g7134 g6579 g504 g4762 g4671 g4421 g3857 g3788 g3128 g2983 g2855 g2509 g2322 g2139 g207 g2060 g1685 g16006 g15356 g15134 g14466 g1164 g11476 g1138 g11240 g11221 g10711 g1008 g2399 g8245 g11984 g11950 g4580 g380 g4289 g4061 g3034 g15455 g13644 g11145 g450 g4833 g6095 g4286 g1233 g918 g8806 g7873 g7564 g7395 g7295 g5932 g5634 g4653 g3810 g3115 g2596 g2268 g221 g16063 g16 g1559 g4571 g9478 g4989 g2857 g2579 g16023 g14291 g10143 |
| 25 | GO:0030554 | g795 g7709 g6095 g5803 g5755 g4286 g2194 g1635 g15156 g1233 g11554 g11041 g10811 g9820 g9743 g8930 g8679 g861 g7749 g7707 g7134 g672 g6410 g637 g6291 g6125 g6012 g5980 g5922 g5694 g5674 g562 g5564 g5393 g5023 g4792 g4773 g4762 g4232 g4061 g3949 g3128 g3115 g3034 g2610 g2571 g2509 g2380 g2357 g2313 g2268 g2229 g16122 g16101 g16006 g15889 g15829 g1571 g15316 g1468 g1467 g14321 g1333 g13321 g12928 g12412 g12146 g12080 g1203 g1119 g1118 g11098 g10901 |
| 26 | GO:0005524 | g7709 g6095 g5803 g5755 g4286 g2194 g1635 g15156 g1233 g11554 g11041 g10811 g9820 g9743 g8930 g8679 g861 g7749 g7707 g7134 g672 g6410 g637 g6291 g6125 g6012 g5980 g5922 g5694 g5674 g562 g5564 g5393 g5023 g4792 g4773 g4762 g4232 g4061 g3949 g3128 g3115 g3034 g2610 g2571 g2509 g2380 g2357 g2313 g2268 g2229 g16122 g16101 g16006 g15889 g15829 g1571 g15316 g1468 g1467 g14321 g1333 g13321 g12928 g12412 g12146 g12080 g1203 g1119 g1118 g11098 g10901 |
| 27 | GO:0032559 | g7709 g6095 g5803 g5755 g4286 g2194 g1635 g15156 g1233 g11554 g11041 g10811 g9820 g9743 g8930 g8679 g861 g7749 g7707 g7134 g672 g6410 g637 g6291 g6125 g6012 g5980 g5922 g5694 g5674 g562 g5564 g5393 g5023 g4792 g4773 g4762 g4232 g4061 g3949 g3128 g3115 g3034 g2610 g2571 g2509 g2380 g2357 g2313 g2268 g2229 g16122 g16101 g16006 g15889 g15829 g1571 g15316 g1468 g1467 g14321 g1333 g13321 g12928 g12412 g12146 g12080 g1203 g1119 g1118 g11098 g10901 |
| 28 | GO:0016772 | g5755 g7134 g3128 g12412 g12080 g10901 g6194 g3416 g9793 g9745 g9261 g8973 g7590 g7095 g5786 g5462 g5344 g4041 g3911 g3037 g2603 g2491 g2186 g16276 g16075 g15244 g1438 g11145 g5678 g16006 g14321 g15457 g15829 g760 g16101 g5803 g12258 g9743 g7711 g4311 g2610 g2229 g1571 g1467 g12159 g15455 g2488 g16055 g8930 g7432 g3810 g9820 g904 g3291 g12200 |
| 29 | GO:0016817 | g10811 g15889 g5023 g2380 g2194 g1333 g5674 g12146 g7709 g1635 g1233 g8810 g6125 g2571 g2313 g13276 g672 g9578 g4773 g10820 g16122 g5755 g8648 g475 g4023 g2852 g15636 g10823 g5922 g2268 g15316 g4286 g13321 g1203 g13019 g15156 g6410 g602 g5980 g5694 g562 g5564 g4232 g3034 g2357 g11098 |
| 30 | GO:0016818 | g10811 g15889 g5023 g2380 g2194 g1333 g5674 g12146 g7709 g1635 g1233 g8810 g6125 g2571 g2313 g13276 g672 g9578 g4773 g10820 g16122 g5755 g8648 g475 g4023 g2852 g15636 g10823 g5922 g2268 g15316 g4286 g13321 g1203 g13019 g15156 g6410 g602 g5980 g5694 g562 g5564 g4232 g3034 g2357 g11098 |
| 31 | GO:0016301 | g5755 g7134 g3128 g12412 g12080 g10901 g6194 g3416 g9793 g9745 g9261 g8973 g7590 g7095 g5786 g5462 g5344 g4041 g3911 g3037 g2603 g2491 g2186 g16276 g16075 g15244 g1438 g11145 g14321 g15457 g15829 g760 g16101 g5803 g12258 g9743 g7711 g4311 g2610 g2229 g1571 g1467 g12159 g8930 g9820 |
| 32 | GO:0016462 | g10811 g15889 g5023 g2380 g2194 g1333 g5674 g12146 g7709 g1635 g1233 g8810 g6125 g2571 g2313 g13276 g672 g9578 g4773 g10820 g16122 g5755 g8648 g475 g4023 g2852 g15636 g10823 g4286 g13321 g1203 g13019 g15156 g6410 g602 g5980 g5694 g562 g5564 g4232 g3034 g2357 g11098 |
| 33 | GO:0017111 | g10811 g15889 g5023 g2380 g2194 g1333 g5674 g12146 g7709 g1635 g1233 g8810 g6125 g2571 g2313 g13276 g672 g9578 g4773 g10820 g16122 g5755 g8648 g475 g4023 g2852 g15636 g10823 g13321 g1203 g13019 g15156 g6410 g602 g5980 g5694 g562 g5564 g4232 g3034 g2357 g11098 |
| 34 | GO:0005215 | g12208 g3660 g11825 g4153 g6522 g6164 g15837 g10390 g9672 g9169 g5595 g5237 g4281 g3153 g2508 g1838 g15584 g11279 g12425 g6962 g15911 g9915 g11986 g10983 g13321 g1203 g1553 g8610 g7359 g15156 g6410 g602 g5980 g5694 g562 g5564 g4232 g3034 g2357 g11098 |
| 35 | GO:0022857 | g12208 g3660 g11825 g4153 g6522 g6164 g15837 g10390 g9672 g9169 g5595 g5237 g4281 g3153 g2508 g1838 g15584 g11279 g12425 g6962 g15911 g9915 g11986 g10983 g13321 g1203 g1553 g8610 g7359 g15156 g6410 g602 g5980 g5694 g562 g5564 g4232 g3034 g2357 g11098 |
| 36 | GO:0005515 | g2477 g795 g5922 g15316 g1635 g3788 g2663 g16122 g16006 g6814 g6710 g15714 g8930 g15457 g1048 g4482 g12620 g9010 g4968 g2260 g16055 g11986 g4057 g9081 g759 g4217 g1225 g14381 g5023 g2380 g1598 g1572 g15536 g7564 g11042 g8648 g10823 |
| 37 | GO:0003677 | g11042 g1635 g10811 g8679 g7564 g7432 g5932 g5922 g5719 g5393 g4671 g4319 g4217 g4094 g3810 g3697 g3128 g2807 g2488 g2464 g221 g16122 g16063 g16055 g16 g15889 g15316 g15421 g1057 g4833 g5674 g2682 g3374 |
| 38 | GO:0046914 | g2399 g8245 g11984 g11950 g4833 g6095 g4286 g1233 g918 g8806 g7873 g7564 g7395 g7295 g5932 g5634 g4653 g3810 g3115 g2596 g2268 g221 g16063 g16 g1559 g4571 g9478 g4989 g2857 g2579 g16023 g14291 g10143 |
| 39 | GO:0048037 | g1559 g5346 g3857 g278 g1636 g7449 g5903 g7249 g7073 g4910 g2983 g1884 g12422 g918 g7307 g4305 g3988 g3987 g2888 g4665 g3274 g2807 g15480 g13590 g7973 g3034 g4580 g4286 g5849 g4289 g14381 |
| 40 | GO:0003723 | g4832 g12225 g2194 g891 g8536 g7932 g6281 g6254 g4305 g4289 g4061 g319 g3076 g2488 g2199 g1333 g13222 g7300 g7518 g12573 g9081 g904 g759 g3788 g2556 g6095 g218 g15714 g7282 |
| 41 | GO:0016887 | g10811 g15889 g5674 g12146 g672 g1635 g9578 g4773 g10820 g16122 g6125 g13321 g1203 g15156 g6410 g602 g5980 g5694 g562 g5564 g4232 g3034 g2357 g11098 |
| 42 | GO:0016773 | g5755 g7134 g3128 g12412 g12080 g10901 g14321 g15829 g760 g16101 g5803 g12258 g9743 g7711 g4311 g2610 g2229 g1571 g1467 g12159 g5344 g8930 g9820 |
| 43 | GO:0050662 | g1559 g5346 g3857 g278 g1636 g7449 g5903 g7249 g7073 g1884 g12422 g4665 g3274 g2807 g15480 g13590 g7973 g3034 g4580 g4286 g5849 g4289 g14381 |
| 44 | GO:0008270 | g4833 g6095 g4286 g1233 g918 g8806 g7873 g7564 g7395 g7295 g5932 g5634 g4653 g3810 g3115 g2596 g2268 g221 g16063 g16 |
| 45 | GO:0042623 | g10811 g15889 g5674 g12146 g672 g16122 g6125 g13321 g1203 g15156 g6410 g602 g5980 g5694 g562 g5564 g4232 g3034 g2357 g11098 |
| 46 | GO:0016798 | g7709 g8896 g7594 g11330 g4571 g9166 g6286 g5886 g4623 g174 g1715 g12204 g11016 g15891 g8112 g1685 g1562 g11568 g6254 |
| 47 | GO:0022804 | g12425 g15911 g9915 g11986 g10983 g13321 g1203 g15156 g6410 g602 g5980 g5694 g562 g5564 g4232 g3034 g2357 g11098 |
| 48 | GO:0004672 | g5755 g7134 g3128 g12412 g12080 g10901 g5803 g12258 g9743 g7711 g4311 g2610 g2229 g1571 g1467 g12159 g8930 |
| 49 | GO:0008233 | g2697 g6579 g5674 g12146 g3115 g1539 g1347 g4643 g3945 g13874 g13864 g4506 g9209 g13151 g6599 g15737 g11549 |
| 50 | GO:0016788 | g218 g5803 g2764 g10152 g470 g16122 g7757 g3911 g15421 g4289 g4365 g7932 g4970 g2983 g15282 g15549 g2737 |
| 51 | GO:0004553 | g7709 g7594 g11330 g4571 g9166 g6286 g5886 g4623 g174 g1715 g12204 g11016 g8112 g1685 g11568 g6254 |
| 52 | GO:0022891 | g12208 g3660 g11825 g4153 g6522 g12425 g6962 g9915 g11986 g10983 g15911 g13321 g1203 g1553 g8610 g7359 |
| 53 | GO:0022892 | g12208 g3660 g11825 g4153 g6522 g12425 g6962 g9915 g11986 g10983 g15911 g13321 g1203 g1553 g8610 g7359 |
| 54 | GO:0070011 | g2697 g6579 g5674 g12146 g3115 g1539 g1347 g4643 g3945 g13874 g13864 g9209 g13151 g6599 g15737 g11549 |
| 55 | GO:0016874 | g4762 g7749 g6012 g11041 g2509 g6095 g4910 g4061 g16006 g9271 g2520 g5636 g5001 g4031 g13028 |
| 56 | GO:0015075 | g12208 g11825 g4153 g6522 g12425 g6962 g9915 g11986 g10983 g15911 g13321 g1203 g1553 g7359 |
| 57 | GO:0004386 | g10811 g15889 g2194 g1333 g7709 g1635 g1233 g8810 g6125 g2571 g2313 g13276 g13019 |
| 58 | GO:0015399 | g13321 g1203 g15156 g6410 g602 g5980 g5694 g562 g5564 g4232 g3034 g2357 g11098 |
| 59 | GO:0015405 | g13321 g1203 g15156 g6410 g602 g5980 g5694 g562 g5564 g4232 g3034 g2357 g11098 |
| 60 | GO:0016820 | g13321 g1203 g15156 g6410 g602 g5980 g5694 g562 g5564 g4232 g3034 g2357 g11098 |
| 61 | GO:0042626 | g13321 g1203 g15156 g6410 g602 g5980 g5694 g562 g5564 g4232 g3034 g2357 g11098 |
| 62 | GO:0043492 | g13321 g1203 g15156 g6410 g602 g5980 g5694 g562 g5564 g4232 g3034 g2357 g11098 |
| 63 | GO:0005506 | g8245 g11984 g11950 g1559 g4571 g9478 g4989 g2857 g2579 g16023 g14291 g10143 |
| 64 | GO:0016741 | g8010 g821 g3498 g8736 g2353 g3710 g7264 g6247 g6281 g12037 g8536 g4762 |
| 65 | GO:0016757 | g4623 g7594 g1160 g8896 g7416 g504 g4435 g15452 g5023 g1768 g11142 g2971 |
| 66 | GO:0005198 | g4023 g6095 g8771 g7633 g7300 g5417 g2983 g16140 g10700 g11542 g11324 |
| 67 | GO:0008168 | g8010 g821 g3498 g8736 g2353 g3710 g7264 g6247 g6281 g12037 g8536 |
| 68 | GO:0044877 | g5922 g15316 g2663 g475 g4057 g12225 g7564 g13320 g5393 g8648 g10823 |
| 69 | GO:0046983 | g9010 g4968 g2260 g16055 g11986 g9081 g759 g4217 g1225 g795 g14381 |
| 70 | GO:0098772 | g727 g7038 g4127 g7590 g4057 g904 g1809 g795 g9797 g11221 g2663 |
| 71 | GO:0004175 | g3115 g1539 g4643 g5674 g3945 g13874 g13864 g12146 g6599 g15737 |
| 72 | GO:0004497 | g278 g1636 g9478 g4989 g2857 g2579 g1884 g16023 g11418 g1541 |
| 73 | GO:0016746 | g15455 g3987 g15379 g8625 g4890 g842 g4165 g3291 g2229 g11148 |
| 74 | GO:0020037 | g7449 g5903 g5515 g9478 g4989 g2857 g2579 g16023 g15134 g10711 |
| 75 | GO:0046906 | g7449 g5903 g5515 g9478 g4989 g2857 g2579 g16023 g15134 g10711 |
| 76 | GO:0001071 | g1057 g4833 g5312 g4319 g7873 g5932 g221 g16063 g16 |
| 77 | GO:0003700 | g1057 g4833 g5312 g4319 g7873 g5932 g221 g16063 g16 |
| 78 | GO:0003743 | g12573 g9081 g904 g759 g7518 g4832 g3788 g2556 g12225 |
| 79 | GO:0008135 | g12225 g12573 g9081 g904 g759 g7518 g4832 g3788 g2556 |
| 80 | GO:0008324 | g12208 g4153 g6522 g9915 g11986 g15911 g13321 g1203 g1553 |
| 81 | GO:0016614 | g4286 g9820 g5849 g4580 g5346 g1374 g3076 g14381 g4289 |
| 82 | GO:0016758 | g4623 g7594 g1160 g7416 g4435 g5023 g1768 g11142 g2971 |
| 83 | GO:0016779 | g5678 g16006 g2488 g16055 g7432 g3810 g904 g3291 g12200 |
| 84 | GO:0016853 | g5719 g15713 g7453 g11549 g8806 g16122 g2152 g15513 g10256 |
| 85 | GO:0042578 | g2764 g10152 g470 g16122 g7757 g3911 g2983 g15282 g15549 |
| 86 | GO:0050660 | g1559 g5346 g3857 g278 g1636 g4665 g3274 g2807 g15480 |
| 87 | GO:0051213 | g2454 g1994 g1541 g15134 g4571 g8245 g11984 g11950 g10143 |
| 88 | GO:0000287 | g4580 g380 g4289 g4061 g3034 g15455 g13644 g11145 |
| 89 | GO:0005525 | g8648 g6593 g6191 g475 g4023 g2852 g15636 g10823 |
| 90 | GO:0016705 | g9478 g4989 g2857 g2579 g16023 g278 g1636 g10711 |
| 91 | GO:0016829 | g380 g13644 g9820 g4637 g8631 g15835 g2596 g502 |
| 92 | GO:0019001 | g8648 g6593 g6191 g475 g4023 g2852 g15636 g10823 |
| 93 | GO:0022890 | g12208 g4153 g9915 g11986 g13321 g1203 g1553 g15911 |
| 94 | GO:0032561 | g8648 g6593 g6191 g475 g4023 g2852 g15636 g10823 |
| 95 | GO:0051536 | g1008 g1559 g1110 g10700 g7449 g5903 g7432 g3810 |
| 96 | GO:0051540 | g1008 g1559 g1110 g10700 g7449 g5903 g7432 g3810 |
| 97 | GO:0003735 | g6095 g8771 g7633 g7300 g5417 g2983 g16140 |
| 98 | GO:0003924 | g5755 g8648 g475 g4023 g2852 g15636 g10823 |
| 99 | GO:0004674 | g5755 g7134 g3128 g12412 g12080 g10901 g8930 |
| 100 | GO:0008236 | g4643 g5674 g3945 g13874 g13864 g12146 g11549 |
| 101 | GO:0016616 | g4286 g9820 g5849 g4580 g3076 g14381 g4289 |
| 102 | GO:0016701 | g14291 g4571 g8245 g11984 g11950 g10143 g1541 |
| 103 | GO:0016791 | g2764 g10152 g470 g16122 g2983 g15282 g15549 |
| 104 | GO:0017171 | g4643 g5674 g3945 g13874 g13864 g12146 g11549 |
| 105 | GO:0000981 | g1057 g7873 g5932 g221 g16063 g16 |
| 106 | GO:0004252 | g4643 g5674 g3945 g13874 g13864 g12146 |
| 107 | GO:0008509 | g11825 g12425 g6962 g10983 g15911 g7359 |
| 108 | GO:0015077 | g12208 g4153 g9915 g11986 g13321 g1203 |
| 109 | GO:0016835 | g380 g13644 g9820 g8631 g2596 g502 |
| 110 | GO:0030170 | g918 g7307 g4305 g3988 g3987 g2888 |
| 111 | GO:0030234 | g7590 g4057 g795 g9797 g11221 g2663 |
| 112 | GO:0032403 | g5922 g15316 g2663 g7564 g8648 g10823 |
| 113 | GO:0051287 | g7073 g4580 g4286 g5849 g4289 g14381 |
| 114 | GO:0003779 | g5023 g2477 g2380 g1598 g1572 |
| 115 | GO:0004518 | g5803 g15421 g4289 g7932 g4970 |
| 116 | GO:0004721 | g2764 g10152 g470 g16122 g2983 |
| 117 | GO:0005085 | g727 g7038 g4127 g904 g1809 |
| 118 | GO:0008092 | g5023 g2477 g2380 g1598 g1572 |
| 119 | GO:0008289 | g2477 g7185 g2883 g8942 g12991 |
| 120 | GO:0008483 | g3988 g918 g2888 g7307 g3987 |
| 121 | GO:0008757 | g821 g3498 g6281 g12037 g8536 |
| 122 | GO:0010181 | g1559 g7449 g5903 g13590 g7973 |
| 123 | GO:0015078 | g12208 g9915 g11986 g13321 g1203 |
| 124 | GO:0015291 | g12425 g15911 g9915 g11986 g10983 |
| 125 | GO:0016651 | g15420 g1110 g2770 g7449 g5903 |
| 126 | GO:0016702 | g4571 g8245 g11984 g11950 g10143 |
| 127 | GO:0016747 | g15455 g3987 g8625 g4890 g11148 |
| 128 | GO:0016769 | g3988 g918 g2888 g7307 g3987 |
| 129 | GO:0016810 | g7777 g5593 g15207 g4286 g7643 |
| 130 | GO:0019899 | g6814 g6710 g15714 g4057 g11042 |
| 131 | GO:0050661 | g7449 g5903 g7249 g278 g1636 |
| 132 | GO:0004842 | g3457 g11042 g7307 g5633 |
| 133 | GO:0008094 | g10811 g15889 g672 g16122 |
| 134 | GO:0008237 | g6579 g3115 g1539 g13151 |
| 135 | GO:0008276 | g8010 g2353 g12037 g8536 |
| 136 | GO:0016620 | g5849 g7249 g3178 g5810 |
| 137 | GO:0016638 | g1559 g5806 g3274 g15480 |
| 138 | GO:0016836 | g9820 g8631 g2596 g502 |
| 139 | GO:0016879 | g4762 g7749 g6012 g2509 |
| 140 | GO:0016903 | g5849 g7249 g3178 g5810 |
| 141 | GO:0019787 | g3457 g11042 g7307 g5633 |
| 142 | GO:0030246 | g4571 g1685 g15104 g11568 |
| 143 | GO:0042802 | g16122 g16006 g795 g14381 |
| 144 | GO:0043021 | g475 g4057 g12225 g13320 |
| 145 | GO:0043565 | g11042 g1057 g4833 g5674 |
| 146 | GO:0046873 | g4153 g11986 g1553 g15911 |
| 147 | GO:0046982 | g9081 g759 g4217 g1225 |
| 148 | GO:0051539 | g7449 g5903 g7432 g3810 |
| 149 | GO:0071949 | g4665 g3274 g2807 g15480 |
| 150 | GO:0000049 | g6095 g218 g15714 |
| 151 | GO:0003678 | g10811 g15889 g13019 |
| 152 | GO:0003682 | g5922 g15316 g5393 |
| 153 | GO:0003755 | g2152 g15513 g10256 |
| 154 | GO:0004527 | g4289 g7932 g4970 |
| 155 | GO:0004812 | g11041 g6095 g4061 |
| 156 | GO:0005088 | g727 g7038 g4127 |
| 157 | GO:0005089 | g727 g7038 g4127 |
| 158 | GO:0005543 | g2477 g7185 g2883 |
| 159 | GO:0008047 | g9797 g11221 g2663 |
| 160 | GO:0008170 | g6281 g12037 g8536 |
| 161 | GO:0008173 | g3498 g8736 g6281 |
| 162 | GO:0008199 | g8245 g11984 g11950 |
| 163 | GO:0008234 | g2697 g1347 g9209 |
| 164 | GO:0008536 | g6814 g6710 g15714 |
| 165 | GO:0015297 | g9915 g11986 g10983 |
| 166 | GO:0015926 | g7594 g11330 g11568 |
| 167 | GO:0016627 | g15455 g7388 g5515 |
| 168 | GO:0016838 | g380 g13644 g9820 |
| 169 | GO:0016859 | g2152 g15513 g10256 |
| 170 | GO:0016875 | g11041 g6095 g4061 |
| 171 | GO:0016876 | g11041 g6095 g4061 |
| 172 | GO:0017016 | g6814 g6710 g15714 |
| 173 | GO:0031267 | g6814 g6710 g15714 |
| 174 | GO:0043022 | g475 g4057 g12225 |
| 175 | GO:0051020 | g6814 g6710 g15714 |
| 176 | GO:0052689 | g218 g4365 g2737 |
| 177 | GO:0060589 | g795 g9797 g11221 |
| 178 | GO:0003684 | g2682 g3374 |
| 179 | GO:0003690 | g11042 g1057 |
| 180 | GO:0003724 | g2194 g1333 |
| 181 | GO:0003774 | g5023 g2380 |
| 182 | GO:0003884 | g3274 g15480 |
| 183 | GO:0003887 | g7432 g3810 |
| 184 | GO:0003899 | g2488 g16055 |
| 185 | GO:0003916 | g5719 g16122 |
| 186 | GO:0003954 | g15420 g1110 |
| 187 | GO:0004003 | g10811 g15889 |
| 188 | GO:0004069 | g918 g2888 |
| 189 | GO:0004133 | g7453 g7594 |
| 190 | GO:0004176 | g5674 g12146 |
| 191 | GO:0004177 | g6579 g13151 |
| 192 | GO:0004190 | g6599 g15737 |
| 193 | GO:0004222 | g3115 g1539 |
| 194 | GO:0004499 | g278 g1636 |
| 195 | GO:0004725 | g470 g16122 |
| 196 | GO:0004783 | g7449 g5903 |
| 197 | GO:0005096 | g9797 g11221 |
| 198 | GO:0005199 | g11542 g11324 |
| 199 | GO:0005216 | g6962 g7359 |
| 200 | GO:0005244 | g6962 g7359 |
| 201 | GO:0005253 | g6962 g7359 |
| 202 | GO:0005516 | g8930 g15457 |
| 203 | GO:0008026 | g10811 g15889 |
| 204 | GO:0008081 | g7757 g3911 |
| 205 | GO:0008134 | g1635 g3788 |
| 206 | GO:0008137 | g15420 g1110 |
| 207 | GO:0008194 | g4435 g2971 |
| 208 | GO:0008238 | g6579 g13151 |
| 209 | GO:0008308 | g6962 g7359 |
| 210 | GO:0008375 | g4435 g2971 |
| 211 | GO:0008553 | g13321 g1203 |
| 212 | GO:0010333 | g380 g13644 |
| 213 | GO:0015103 | g11825 g7359 |
| 214 | GO:0015267 | g6962 g7359 |
| 215 | GO:0015298 | g9915 g11986 |
| 216 | GO:0015299 | g9915 g11986 |
| 217 | GO:0015616 | g672 g15889 |
| 218 | GO:0015662 | g13321 g1203 |
| 219 | GO:0015932 | g3660 g8610 |
| 220 | GO:0016278 | g12037 g8536 |
| 221 | GO:0016279 | g12037 g8536 |
| 222 | GO:0016410 | g3987 g8625 |
| 223 | GO:0016641 | g3274 g15480 |
| 224 | GO:0016655 | g15420 g1110 |
| 225 | GO:0016667 | g7449 g5903 |
| 226 | GO:0016668 | g7449 g5903 |
| 227 | GO:0016709 | g278 g1636 |
| 228 | GO:0016763 | g8896 g504 |
| 229 | GO:0016765 | g7203 g9820 |
| 230 | GO:0016782 | g16006 g1598 |
| 231 | GO:0016799 | g8896 g15891 |
| 232 | GO:0016877 | g4910 g16006 |
| 233 | GO:0016884 | g4762 g7749 |
| 234 | GO:0017025 | g1635 g3788 |
| 235 | GO:0018024 | g12037 g8536 |
| 236 | GO:0019207 | g7590 g4057 |
| 237 | GO:0019783 | g1347 g9209 |
| 238 | GO:0019829 | g13321 g1203 |
| 239 | GO:0019843 | g7300 g7282 |
| 240 | GO:0019887 | g7590 g4057 |
| 241 | GO:0022803 | g6962 g7359 |
| 242 | GO:0022832 | g6962 g7359 |
| 243 | GO:0022836 | g6962 g7359 |
| 244 | GO:0022838 | g6962 g7359 |
| 245 | GO:0030695 | g9797 g11221 |
| 246 | GO:0031491 | g5922 g15316 |
| 247 | GO:0031683 | g8648 g10823 |
| 248 | GO:0034061 | g7432 g3810 |
| 249 | GO:0034062 | g2488 g16055 |
| 250 | GO:0035091 | g7185 g2883 |
| 251 | GO:0036442 | g13321 g1203 |
| 252 | GO:0036459 | g1347 g9209 |
| 253 | GO:0042054 | g12037 g8536 |
| 254 | GO:0042625 | g13321 g1203 |
| 255 | GO:0042803 | g795 g14381 |
| 256 | GO:0050136 | g15420 g1110 |
| 257 | GO:0061630 | g3457 g11042 |
| 258 | GO:0061659 | g3457 g11042 |
| 259 | GO:0070001 | g6599 g15737 |
| 260 | GO:0070035 | g10811 g15889 |
| 261 | GO:0099516 | g11986 g10983 |
| 262 | GO:0101005 | g1347 g9209 |
| 263 | GO:1901505 | g3660 g8610 |
| 264 | GO:1901677 | g15911 g8610 |
| 265 | GO:1990837 | g11042 g1057 |
| 266 | GO:0000030 | g1160 |
| 267 | GO:0000104 | g5515 |
| 268 | GO:0000175 | g4289 |
| 269 | GO:0000179 | g6281 |
| 270 | GO:0000334 | g10143 |
| 271 | GO:0000339 | g4832 |
| 272 | GO:0000774 | g795 |
| 273 | GO:0000822 | g15457 |
| 274 | GO:0000975 | g1057 |
| 275 | GO:0000976 | g1057 |
| 276 | GO:0000977 | g1057 |
| 277 | GO:0000978 | g1057 |
| 278 | GO:0000987 | g1057 |
| 279 | GO:0000988 | g3788 |
| 280 | GO:0000990 | g3788 |
| 281 | GO:0000991 | g3788 |
| 282 | GO:0001012 | g1057 |
| 283 | GO:0001055 | g2488 |
| 284 | GO:0001067 | g1057 |
| 285 | GO:0001139 | g3788 |
| 286 | GO:0001159 | g1057 |
| 287 | GO:0001228 | g1057 |
| 288 | GO:0001664 | g10823 |
| 289 | GO:0001871 | g11568 |
| 290 | GO:0002094 | g7203 |
| 291 | GO:0003688 | g11042 |
| 292 | GO:0003697 | g15421 |
| 293 | GO:0003725 | g7518 |
| 294 | GO:0003727 | g7518 |
| 295 | GO:0003746 | g12225 |
| 296 | GO:0003844 | g4623 |
| 297 | GO:0003855 | g9820 |
| 298 | GO:0003856 | g9820 |
| 299 | GO:0003862 | g4580 |
| 300 | GO:0003866 | g9820 |
| 301 | GO:0003870 | g3987 |
| 302 | GO:0003873 | g15829 |
| 303 | GO:0003883 | g6012 |
| 304 | GO:0003917 | g5719 |
| 305 | GO:0003918 | g16122 |
| 306 | GO:0003951 | g760 |
| 307 | GO:0003952 | g7749 |
| 308 | GO:0003968 | g2488 |
| 309 | GO:0003992 | g3988 |
| 310 | GO:0004040 | g7643 |
| 311 | GO:0004042 | g8625 |
| 312 | GO:0004044 | g504 |
| 313 | GO:0004070 | g4762 |
| 314 | GO:0004088 | g4762 |
| 315 | GO:0004089 | g2596 |
| 316 | GO:0004100 | g4435 |
| 317 | GO:0004128 | g2770 |
| 318 | GO:0004134 | g7594 |
| 319 | GO:0004135 | g7594 |
| 320 | GO:0004164 | g821 |
| 321 | GO:0004169 | g1160 |
| 322 | GO:0004312 | g15455 |
| 323 | GO:0004318 | g15455 |
| 324 | GO:0004339 | g11568 |
| 325 | GO:0004350 | g7249 |
| 326 | GO:0004355 | g1559 |
| 327 | GO:0004358 | g8625 |
| 328 | GO:0004367 | g14381 |
| 329 | GO:0004371 | g14321 |
| 330 | GO:0004375 | g5806 |
| 331 | GO:0004399 | g4286 |
| 332 | GO:0004448 | g4289 |
| 333 | GO:0004449 | g4289 |
| 334 | GO:0004455 | g3076 |
| 335 | GO:0004476 | g8806 |
| 336 | GO:0004491 | g3178 |
| 337 | GO:0004519 | g15421 |
| 338 | GO:0004532 | g4289 |
| 339 | GO:0004540 | g4289 |
| 340 | GO:0004555 | g6254 |
| 341 | GO:0004559 | g1685 |
| 342 | GO:0004563 | g8112 |
| 343 | GO:0004605 | g5678 |
| 344 | GO:0004635 | g4286 |
| 345 | GO:0004636 | g4286 |
| 346 | GO:0004637 | g2509 |
| 347 | GO:0004641 | g2509 |
| 348 | GO:0004650 | g7709 |
| 349 | GO:0004659 | g7203 |
| 350 | GO:0004683 | g8930 |
| 351 | GO:0004713 | g12080 |
| 352 | GO:0004722 | g2983 |
| 353 | GO:0004724 | g2983 |
| 354 | GO:0004731 | g8896 |
| 355 | GO:0004748 | g11554 |
| 356 | GO:0004749 | g11145 |
| 357 | GO:0004764 | g9820 |
| 358 | GO:0004765 | g9820 |
| 359 | GO:0004768 | g10711 |
| 360 | GO:0004774 | g4910 |
| 361 | GO:0004775 | g4910 |
| 362 | GO:0004792 | g16006 |
| 363 | GO:0004801 | g6334 |
| 364 | GO:0004806 | g4365 |
| 365 | GO:0004813 | g6095 |
| 366 | GO:0004826 | g4061 |
| 367 | GO:0004830 | g11041 |
| 368 | GO:0004834 | g502 |
| 369 | GO:0004843 | g1347 |
| 370 | GO:0004846 | g7873 |
| 371 | GO:0005102 | g10823 |
| 372 | GO:0005200 | g4023 |
| 373 | GO:0005247 | g7359 |
| 374 | GO:0005254 | g7359 |
| 375 | GO:0005315 | g15911 |
| 376 | GO:0005337 | g3660 |
| 377 | GO:0005338 | g8610 |
| 378 | GO:0005342 | g12425 |
| 379 | GO:0005371 | g12425 |
| 380 | GO:0005384 | g15911 |
| 381 | GO:0005451 | g11986 |
| 382 | GO:0005452 | g10983 |
| 383 | GO:0005457 | g8610 |
| 384 | GO:0005507 | g2399 |
| 385 | GO:0005509 | g450 |
| 386 | GO:0008080 | g8625 |
| 387 | GO:0008171 | g2353 |
| 388 | GO:0008175 | g3498 |
| 389 | GO:0008186 | g6125 |
| 390 | GO:0008198 | g10143 |
| 391 | GO:0008235 | g6579 |
| 392 | GO:0008260 | g1598 |
| 393 | GO:0008408 | g4289 |
| 394 | GO:0008410 | g1598 |
| 395 | GO:0008422 | g11330 |
| 396 | GO:0008443 | g15829 |
| 397 | GO:0008446 | g8631 |
| 398 | GO:0008477 | g8896 |
| 399 | GO:0008514 | g12425 |
| 400 | GO:0008641 | g16006 |
| 401 | GO:0008649 | g6281 |
| 402 | GO:0008863 | g5849 |
| 403 | GO:0008897 | g15455 |
| 404 | GO:0008976 | g15457 |
| 405 | GO:0009055 | g1110 |
| 406 | GO:0009982 | g15713 |
| 407 | GO:0010340 | g2353 |
| 408 | GO:0015079 | g4153 |
| 409 | GO:0015081 | g11986 |
| 410 | GO:0015108 | g7359 |
| 411 | GO:0015109 | g11825 |
| 412 | GO:0015142 | g12425 |
| 413 | GO:0015215 | g8610 |
| 414 | GO:0015293 | g15911 |
| 415 | GO:0015294 | g15911 |
| 416 | GO:0015295 | g15911 |
| 417 | GO:0015301 | g10983 |
| 418 | GO:0015385 | g11986 |
| 419 | GO:0015491 | g11986 |
| 420 | GO:0015605 | g8610 |
| 421 | GO:0015923 | g1685 |
| 422 | GO:0015927 | g6254 |
| 423 | GO:0015929 | g8112 |
| 424 | GO:0015930 | g1559 |
| 425 | GO:0016040 | g1559 |
| 426 | GO:0016209 | g9478 |
| 427 | GO:0016215 | g10711 |
| 428 | GO:0016298 | g4365 |
| 429 | GO:0016303 | g16101 |
| 430 | GO:0016405 | g4910 |
| 431 | GO:0016407 | g8625 |
| 432 | GO:0016409 | g11148 |
| 433 | GO:0016417 | g11148 |
| 434 | GO:0016427 | g3498 |
| 435 | GO:0016433 | g6281 |
| 436 | GO:0016504 | g2663 |
| 437 | GO:0016597 | g4762 |
| 438 | GO:0016628 | g15455 |
| 439 | GO:0016639 | g1559 |
| 440 | GO:0016642 | g5806 |
| 441 | GO:0016653 | g2770 |
| 442 | GO:0016661 | g7873 |
| 443 | GO:0016663 | g7873 |
| 444 | GO:0016703 | g1541 |
| 445 | GO:0016717 | g10711 |
| 446 | GO:0016725 | g11554 |
| 447 | GO:0016728 | g11554 |
| 448 | GO:0016743 | g4762 |
| 449 | GO:0016744 | g6334 |
| 450 | GO:0016748 | g3987 |
| 451 | GO:0016749 | g3987 |
| 452 | GO:0016776 | g15457 |
| 453 | GO:0016778 | g11145 |
| 454 | GO:0016780 | g15455 |
| 455 | GO:0016783 | g16006 |
| 456 | GO:0016796 | g4289 |
| 457 | GO:0016811 | g7643 |
| 458 | GO:0016814 | g4286 |
| 459 | GO:0016830 | g15835 |
| 460 | GO:0016832 | g15835 |
| 461 | GO:0016860 | g8806 |
| 462 | GO:0016861 | g8806 |
| 463 | GO:0016866 | g15713 |
| 464 | GO:0016878 | g4910 |
| 465 | GO:0016882 | g2509 |
| 466 | GO:0016896 | g4289 |
| 467 | GO:0017057 | g2737 |
| 468 | GO:0017176 | g2971 |
| 469 | GO:0018580 | g1541 |
| 470 | GO:0019200 | g15829 |
| 471 | GO:0019238 | g4286 |
| 472 | GO:0019706 | g11148 |
| 473 | GO:0019707 | g11148 |
| 474 | GO:0019842 | g3034 |
| 475 | GO:0019900 | g4057 |
| 476 | GO:0019901 | g4057 |
| 477 | GO:0030247 | g11568 |
| 478 | GO:0030276 | g2477 |
| 479 | GO:0030674 | g11042 |
| 480 | GO:0030976 | g3034 |
| 481 | GO:0031406 | g4762 |
| 482 | GO:0031625 | g11042 |
| 483 | GO:0032131 | g3374 |
| 484 | GO:0032132 | g3374 |
| 485 | GO:0033592 | g7518 |
| 486 | GO:0035004 | g16101 |
| 487 | GO:0036080 | g8610 |
| 488 | GO:0042134 | g7282 |
| 489 | GO:0042292 | g16006 |
| 490 | GO:0042393 | g2663 |
| 491 | GO:0042800 | g8536 |
| 492 | GO:0043023 | g13320 |
| 493 | GO:0043177 | g4762 |
| 494 | GO:0043178 | g15457 |
| 495 | GO:0044212 | g1057 |
| 496 | GO:0044389 | g11042 |
| 497 | GO:0045140 | g7416 |
| 498 | GO:0045181 | g1559 |
| 499 | GO:0045735 | g11148 |
| 500 | GO:0046912 | g15379 |
| 501 | GO:0046915 | g15911 |
| 502 | GO:0046943 | g12425 |
| 503 | GO:0047724 | g8896 |
| 504 | GO:0050113 | g14291 |
| 505 | GO:0051082 | g15536 |
| 506 | GO:0051087 | g795 |
| 507 | GO:0051499 | g218 |
| 508 | GO:0051500 | g218 |
| 509 | GO:0051537 | g1008 |
| 510 | GO:0051538 | g1559 |
| 511 | GO:0051998 | g2353 |
| 512 | GO:0052742 | g16101 |
| 513 | GO:0060090 | g11042 |
| 514 | GO:0060590 | g795 |
| 515 | GO:0061134 | g2663 |
| 516 | GO:0061505 | g16122 |
| 517 | GO:0061731 | g11554 |
| 518 | GO:0070006 | g6579 |
| 519 | GO:0070403 | g7073 |
| 520 | GO:0070566 | g16006 |
| 521 | GO:0070567 | g5678 |
| 522 | GO:0070577 | g2663 |
| 523 | GO:0070628 | g2663 |
| 524 | GO:0070635 | g8896 |
| 525 | GO:0070733 | g16006 |
| 526 | GO:0070840 | g7564 |
| 527 | GO:0090599 | g7594 |
| 528 | GO:0097079 | g15911 |
| 529 | GO:0097617 | g7518 |
| 530 | GO:0098808 | g4832 |
| 531 | GO:1901681 | g3034 |
| 532 | GO:1990380 | g1347 |
| 533 | GO:2001070 | g11568 |

  

---

GO Directed Acycline Graph

Back Top
