# Supplementary material for: A Comprehensive Assessment of Ultraviolet-Radiation-Induced Mutations in Flammulina filiformis Using Whole-Genome Resequencing
Source: J Fungi (Basel). 2024 Mar 20;10(3):228. doi: 10.3390/jof10030228 (PMC10971301; doi:10.3390/jof10030228)
Supplement: Supplementary file 1 [file jof-10-00228-s001.zip › Supplementary Material S8/GO annotation/out/out.P.html]

 


GO Enrichment Analysis


out GO Enrichment (Biological Process)

| # | GO ID | Description | GeneRatio (419) | BgRatio (419) | pvalue | fdr |
| 1 | GO:0009987 | cellular process | 294 | 294 | 1.000000 | 1.000000 |
| 2 | GO:0008152 | metabolic process | 279 | 279 | 1.000000 | 1.000000 |
| 3 | GO:0044699 | single-organism process | 226 | 226 | 1.000000 | 1.000000 |
| 4 | GO:0071704 | organic substance metabolic process | 212 | 212 | 1.000000 | 1.000000 |
| 5 | GO:0044238 | primary metabolic process | 204 | 204 | 1.000000 | 1.000000 |
| 6 | GO:0044237 | cellular metabolic process | 199 | 199 | 1.000000 | 1.000000 |
| 7 | GO:0044763 | single-organism cellular process | 163 | 163 | 1.000000 | 1.000000 |
| 8 | GO:0043170 | macromolecule metabolic process | 133 | 133 | 1.000000 | 1.000000 |
| 9 | GO:0044710 | single-organism metabolic process | 125 | 125 | 1.000000 | 1.000000 |
| 10 | GO:0006807 | nitrogen compound metabolic process | 118 | 118 | 1.000000 | 1.000000 |
| 11 | GO:0044260 | cellular macromolecule metabolic process | 112 | 112 | 1.000000 | 1.000000 |
| 12 | GO:0034641 | cellular nitrogen compound metabolic process | 94 | 94 | 1.000000 | 1.000000 |
| 13 | GO:0051179 | localization | 91 | 91 | 1.000000 | 1.000000 |
| 14 | GO:0051234 | establishment of localization | 89 | 89 | 1.000000 | 1.000000 |
| 15 | GO:0006810 | transport | 87 | 87 | 1.000000 | 1.000000 |
| 16 | GO:0006725 | cellular aromatic compound metabolic process | 86 | 86 | 1.000000 | 1.000000 |
| 17 | GO:1901360 | organic cyclic compound metabolic process | 85 | 85 | 1.000000 | 1.000000 |
| 18 | GO:0046483 | heterocycle metabolic process | 83 | 83 | 1.000000 | 1.000000 |
| 19 | GO:0009058 | biosynthetic process | 79 | 79 | 1.000000 | 1.000000 |
| 20 | GO:0006139 | nucleobase-containing compound metabolic process | 77 | 77 | 1.000000 | 1.000000 |
| 21 | GO:0019538 | protein metabolic process | 75 | 75 | 1.000000 | 1.000000 |
| 22 | GO:1902578 | single-organism localization | 73 | 73 | 1.000000 | 1.000000 |
| 23 | GO:0044765 | single-organism transport | 72 | 72 | 1.000000 | 1.000000 |
| 24 | GO:1901576 | organic substance biosynthetic process | 72 | 72 | 1.000000 | 1.000000 |
| 25 | GO:0044249 | cellular biosynthetic process | 71 | 71 | 1.000000 | 1.000000 |
| 26 | GO:0065007 | biological regulation | 68 | 68 | 1.000000 | 1.000000 |
| 27 | GO:0006793 | phosphorus metabolic process | 67 | 67 | 1.000000 | 1.000000 |
| 28 | GO:0055114 | oxidation-reduction process | 67 | 67 | 1.000000 | 1.000000 |
| 29 | GO:0006796 | phosphate-containing compound metabolic process | 66 | 66 | 1.000000 | 1.000000 |
| 30 | GO:0050789 | regulation of biological process | 64 | 64 | 1.000000 | 1.000000 |
| 31 | GO:0044267 | cellular protein metabolic process | 62 | 62 | 1.000000 | 1.000000 |
| 32 | GO:0050794 | regulation of cellular process | 60 | 60 | 1.000000 | 1.000000 |
| 33 | GO:0090304 | nucleic acid metabolic process | 59 | 59 | 1.000000 | 1.000000 |
| 34 | GO:1901564 | organonitrogen compound metabolic process | 57 | 57 | 1.000000 | 1.000000 |
| 35 | GO:0044281 | small molecule metabolic process | 51 | 51 | 1.000000 | 1.000000 |
| 36 | GO:0055085 | transmembrane transport | 51 | 51 | 1.000000 | 1.000000 |
| 37 | GO:0071840 | cellular component organization or biogenesis | 48 | 48 | 1.000000 | 1.000000 |
| 38 | GO:0043412 | macromolecule modification | 45 | 45 | 1.000000 | 1.000000 |
| 39 | GO:0010467 | gene expression | 43 | 43 | 1.000000 | 1.000000 |
| 40 | GO:1901566 | organonitrogen compound biosynthetic process | 42 | 42 | 1.000000 | 1.000000 |
| 41 | GO:0006464 | cellular protein modification process | 41 | 41 | 1.000000 | 1.000000 |
| 42 | GO:0016043 | cellular component organization | 41 | 41 | 1.000000 | 1.000000 |
| 43 | GO:0016310 | phosphorylation | 41 | 41 | 1.000000 | 1.000000 |
| 44 | GO:0036211 | protein modification process | 41 | 41 | 1.000000 | 1.000000 |
| 45 | GO:0044271 | cellular nitrogen compound biosynthetic process | 41 | 41 | 1.000000 | 1.000000 |
| 46 | GO:0019222 | regulation of metabolic process | 39 | 39 | 1.000000 | 1.000000 |
| 47 | GO:0009059 | macromolecule biosynthetic process | 36 | 36 | 1.000000 | 1.000000 |
| 48 | GO:0044711 | single-organism biosynthetic process | 36 | 36 | 1.000000 | 1.000000 |
| 49 | GO:0034645 | cellular macromolecule biosynthetic process | 35 | 35 | 1.000000 | 1.000000 |
| 50 | GO:0050896 | response to stimulus | 35 | 35 | 1.000000 | 1.000000 |
| 51 | GO:0006082 | organic acid metabolic process | 34 | 34 | 1.000000 | 1.000000 |
| 52 | GO:0043436 | oxoacid metabolic process | 34 | 34 | 1.000000 | 1.000000 |
| 53 | GO:0016070 | RNA metabolic process | 33 | 33 | 1.000000 | 1.000000 |
| 54 | GO:0005975 | carbohydrate metabolic process | 32 | 32 | 1.000000 | 1.000000 |
| 55 | GO:0019752 | carboxylic acid metabolic process | 32 | 32 | 1.000000 | 1.000000 |
| 56 | GO:0060255 | regulation of macromolecule metabolic process | 32 | 32 | 1.000000 | 1.000000 |
| 57 | GO:0031323 | regulation of cellular metabolic process | 31 | 31 | 1.000000 | 1.000000 |
| 58 | GO:0080090 | regulation of primary metabolic process | 31 | 31 | 1.000000 | 1.000000 |
| 59 | GO:0006996 | organelle organization | 30 | 30 | 1.000000 | 1.000000 |
| 60 | GO:0010468 | regulation of gene expression | 29 | 29 | 1.000000 | 1.000000 |
| 61 | GO:0051171 | regulation of nitrogen compound metabolic process | 29 | 29 | 1.000000 | 1.000000 |
| 62 | GO:0051716 | cellular response to stimulus | 29 | 29 | 1.000000 | 1.000000 |
| 63 | GO:1901362 | organic cyclic compound biosynthetic process | 29 | 29 | 1.000000 | 1.000000 |
| 64 | GO:0009889 | regulation of biosynthetic process | 28 | 28 | 1.000000 | 1.000000 |
| 65 | GO:0010556 | regulation of macromolecule biosynthetic process | 28 | 28 | 1.000000 | 1.000000 |
| 66 | GO:0018130 | heterocycle biosynthetic process | 28 | 28 | 1.000000 | 1.000000 |
| 67 | GO:0031326 | regulation of cellular biosynthetic process | 28 | 28 | 1.000000 | 1.000000 |
| 68 | GO:2000112 | regulation of cellular macromolecule biosynthetic process | 28 | 28 | 1.000000 | 1.000000 |
| 69 | GO:0006520 | cellular amino acid metabolic process | 27 | 27 | 1.000000 | 1.000000 |
| 70 | GO:0009056 | catabolic process | 27 | 27 | 1.000000 | 1.000000 |
| 71 | GO:0019219 | regulation of nucleobase-containing compound metabolic process | 26 | 26 | 1.000000 | 1.000000 |
| 72 | GO:0019438 | aromatic compound biosynthetic process | 26 | 26 | 1.000000 | 1.000000 |
| 73 | GO:1901575 | organic substance catabolic process | 26 | 26 | 1.000000 | 1.000000 |
| 74 | GO:0051252 | regulation of RNA metabolic process | 25 | 25 | 1.000000 | 1.000000 |
| 75 | GO:0006259 | DNA metabolic process | 24 | 24 | 1.000000 | 1.000000 |
| 76 | GO:0006355 | regulation of transcription, DNA-templated | 24 | 24 | 1.000000 | 1.000000 |
| 77 | GO:0051641 | cellular localization | 24 | 24 | 1.000000 | 1.000000 |
| 78 | GO:1903506 | regulation of nucleic acid-templated transcription | 24 | 24 | 1.000000 | 1.000000 |
| 79 | GO:2001141 | regulation of RNA biosynthetic process | 24 | 24 | 1.000000 | 1.000000 |
| 80 | GO:0043933 | macromolecular complex subunit organization | 23 | 23 | 1.000000 | 1.000000 |
| 81 | GO:0051649 | establishment of localization in cell | 23 | 23 | 1.000000 | 1.000000 |
| 82 | GO:0034654 | nucleobase-containing compound biosynthetic process | 22 | 22 | 1.000000 | 1.000000 |
| 83 | GO:0044248 | cellular catabolic process | 21 | 21 | 1.000000 | 1.000000 |
| 84 | GO:0006508 | proteolysis | 20 | 20 | 1.000000 | 1.000000 |
| 85 | GO:0019637 | organophosphate metabolic process | 19 | 19 | 1.000000 | 1.000000 |
| 86 | GO:0071702 | organic substance transport | 19 | 19 | 1.000000 | 1.000000 |
| 87 | GO:0006412 | translation | 18 | 18 | 1.000000 | 1.000000 |
| 88 | GO:0006518 | peptide metabolic process | 18 | 18 | 1.000000 | 1.000000 |
| 89 | GO:0043043 | peptide biosynthetic process | 18 | 18 | 1.000000 | 1.000000 |
| 90 | GO:0043603 | cellular amide metabolic process | 18 | 18 | 1.000000 | 1.000000 |
| 91 | GO:0043604 | amide biosynthetic process | 18 | 18 | 1.000000 | 1.000000 |
| 92 | GO:0044085 | cellular component biogenesis | 18 | 18 | 1.000000 | 1.000000 |
| 93 | GO:0046907 | intracellular transport | 18 | 18 | 1.000000 | 1.000000 |
| 94 | GO:1901135 | carbohydrate derivative metabolic process | 18 | 18 | 1.000000 | 1.000000 |
| 95 | GO:1901605 | alpha-amino acid metabolic process | 18 | 18 | 1.000000 | 1.000000 |
| 96 | GO:0006468 | protein phosphorylation | 17 | 17 | 1.000000 | 1.000000 |
| 97 | GO:0007154 | cell communication | 17 | 17 | 1.000000 | 1.000000 |
| 98 | GO:0007165 | signal transduction | 17 | 17 | 1.000000 | 1.000000 |
| 99 | GO:0009057 | macromolecule catabolic process | 17 | 17 | 1.000000 | 1.000000 |
| 100 | GO:0023052 | signaling | 17 | 17 | 1.000000 | 1.000000 |
| 101 | GO:0044700 | single organism signaling | 17 | 17 | 1.000000 | 1.000000 |
| 102 | GO:0006396 | RNA processing | 16 | 16 | 1.000000 | 1.000000 |
| 103 | GO:0006811 | ion transport | 16 | 16 | 1.000000 | 1.000000 |
| 104 | GO:0044283 | small molecule biosynthetic process | 16 | 16 | 1.000000 | 1.000000 |
| 105 | GO:0051276 | chromosome organization | 16 | 16 | 1.000000 | 1.000000 |
| 106 | GO:0016053 | organic acid biosynthetic process | 15 | 15 | 1.000000 | 1.000000 |
| 107 | GO:0046394 | carboxylic acid biosynthetic process | 15 | 15 | 1.000000 | 1.000000 |
| 108 | GO:1902582 | single-organism intracellular transport | 15 | 15 | 1.000000 | 1.000000 |
| 109 | GO:1902589 | single-organism organelle organization | 15 | 15 | 1.000000 | 1.000000 |
| 110 | GO:0033036 | macromolecule localization | 14 | 14 | 1.000000 | 1.000000 |
| 111 | GO:0055086 | nucleobase-containing small molecule metabolic process | 14 | 14 | 1.000000 | 1.000000 |
| 112 | GO:0006629 | lipid metabolic process | 13 | 13 | 1.000000 | 1.000000 |
| 113 | GO:0044723 | single-organism carbohydrate metabolic process | 13 | 13 | 1.000000 | 1.000000 |
| 114 | GO:0090407 | organophosphate biosynthetic process | 13 | 13 | 1.000000 | 1.000000 |
| 115 | GO:0006950 | response to stress | 12 | 12 | 1.000000 | 1.000000 |
| 116 | GO:0008652 | cellular amino acid biosynthetic process | 12 | 12 | 1.000000 | 1.000000 |
| 117 | GO:0016192 | vesicle-mediated transport | 12 | 12 | 1.000000 | 1.000000 |
| 118 | GO:0022613 | ribonucleoprotein complex biogenesis | 12 | 12 | 1.000000 | 1.000000 |
| 119 | GO:0044265 | cellular macromolecule catabolic process | 12 | 12 | 1.000000 | 1.000000 |
| 120 | GO:0065009 | regulation of molecular function | 12 | 12 | 1.000000 | 1.000000 |
| 121 | GO:0006357 | regulation of transcription from RNA polymerase II promoter | 11 | 11 | 1.000000 | 1.000000 |
| 122 | GO:0006753 | nucleoside phosphate metabolic process | 11 | 11 | 1.000000 | 1.000000 |
| 123 | GO:0008104 | protein localization | 11 | 11 | 1.000000 | 1.000000 |
| 124 | GO:0009117 | nucleotide metabolic process | 11 | 11 | 1.000000 | 1.000000 |
| 125 | GO:0022607 | cellular component assembly | 11 | 11 | 1.000000 | 1.000000 |
| 126 | GO:0032259 | methylation | 11 | 11 | 1.000000 | 1.000000 |
| 127 | GO:0033554 | cellular response to stress | 11 | 11 | 1.000000 | 1.000000 |
| 128 | GO:0034660 | ncRNA metabolic process | 11 | 11 | 1.000000 | 1.000000 |
| 129 | GO:0044255 | cellular lipid metabolic process | 11 | 11 | 1.000000 | 1.000000 |
| 130 | GO:0050790 | regulation of catalytic activity | 11 | 11 | 1.000000 | 1.000000 |
| 131 | GO:0006351 | transcription, DNA-templated | 10 | 10 | 1.000000 | 1.000000 |
| 132 | GO:0006812 | cation transport | 10 | 10 | 1.000000 | 1.000000 |
| 133 | GO:0015031 | protein transport | 10 | 10 | 1.000000 | 1.000000 |
| 134 | GO:0016482 | cytoplasmic transport | 10 | 10 | 1.000000 | 1.000000 |
| 135 | GO:0032774 | RNA biosynthetic process | 10 | 10 | 1.000000 | 1.000000 |
| 136 | GO:0034622 | cellular macromolecular complex assembly | 10 | 10 | 1.000000 | 1.000000 |
| 137 | GO:0044712 | single-organism catabolic process | 10 | 10 | 1.000000 | 1.000000 |
| 138 | GO:0045184 | establishment of protein localization | 10 | 10 | 1.000000 | 1.000000 |
| 139 | GO:0048518 | positive regulation of biological process | 10 | 10 | 1.000000 | 1.000000 |
| 140 | GO:0065003 | macromolecular complex assembly | 10 | 10 | 1.000000 | 1.000000 |
| 141 | GO:0097659 | nucleic acid-templated transcription | 10 | 10 | 1.000000 | 1.000000 |
| 142 | GO:0006325 | chromatin organization | 9 | 9 | 1.000000 | 1.000000 |
| 143 | GO:0006413 | translational initiation | 9 | 9 | 1.000000 | 1.000000 |
| 144 | GO:0006974 | cellular response to DNA damage stimulus | 9 | 9 | 1.000000 | 1.000000 |
| 145 | GO:0016071 | mRNA metabolic process | 9 | 9 | 1.000000 | 1.000000 |
| 146 | GO:0048519 | negative regulation of biological process | 9 | 9 | 1.000000 | 1.000000 |
| 147 | GO:0065008 | regulation of biological quality | 9 | 9 | 1.000000 | 1.000000 |
| 148 | GO:1901137 | carbohydrate derivative biosynthetic process | 9 | 9 | 1.000000 | 1.000000 |
| 149 | GO:0007049 | cell cycle | 8 | 8 | 1.000000 | 1.000000 |
| 150 | GO:0008610 | lipid biosynthetic process | 8 | 8 | 1.000000 | 1.000000 |
| 151 | GO:0009064 | glutamine family amino acid metabolic process | 8 | 8 | 1.000000 | 1.000000 |
| 152 | GO:0009165 | nucleotide biosynthetic process | 8 | 8 | 1.000000 | 1.000000 |
| 153 | GO:0009893 | positive regulation of metabolic process | 8 | 8 | 1.000000 | 1.000000 |
| 154 | GO:0018193 | peptidyl-amino acid modification | 8 | 8 | 1.000000 | 1.000000 |
| 155 | GO:0019439 | aromatic compound catabolic process | 8 | 8 | 1.000000 | 1.000000 |
| 156 | GO:0022402 | cell cycle process | 8 | 8 | 1.000000 | 1.000000 |
| 157 | GO:0030163 | protein catabolic process | 8 | 8 | 1.000000 | 1.000000 |
| 158 | GO:0034220 | ion transmembrane transport | 8 | 8 | 1.000000 | 1.000000 |
| 159 | GO:0034470 | ncRNA processing | 8 | 8 | 1.000000 | 1.000000 |
| 160 | GO:0042221 | response to chemical | 8 | 8 | 1.000000 | 1.000000 |
| 161 | GO:0043632 | modification-dependent macromolecule catabolic process | 8 | 8 | 1.000000 | 1.000000 |
| 162 | GO:0044262 | cellular carbohydrate metabolic process | 8 | 8 | 1.000000 | 1.000000 |
| 163 | GO:0044270 | cellular nitrogen compound catabolic process | 8 | 8 | 1.000000 | 1.000000 |
| 164 | GO:0046700 | heterocycle catabolic process | 8 | 8 | 1.000000 | 1.000000 |
| 165 | GO:0048522 | positive regulation of cellular process | 8 | 8 | 1.000000 | 1.000000 |
| 166 | GO:0048523 | negative regulation of cellular process | 8 | 8 | 1.000000 | 1.000000 |
| 167 | GO:0051336 | regulation of hydrolase activity | 8 | 8 | 1.000000 | 1.000000 |
| 168 | GO:1901293 | nucleoside phosphate biosynthetic process | 8 | 8 | 1.000000 | 1.000000 |
| 169 | GO:1901361 | organic cyclic compound catabolic process | 8 | 8 | 1.000000 | 1.000000 |
| 170 | GO:1901607 | alpha-amino acid biosynthetic process | 8 | 8 | 1.000000 | 1.000000 |
| 171 | GO:0000278 | mitotic cell cycle | 7 | 7 | 1.000000 | 1.000000 |
| 172 | GO:0006260 | DNA replication | 7 | 7 | 1.000000 | 1.000000 |
| 173 | GO:0006281 | DNA repair | 7 | 7 | 1.000000 | 1.000000 |
| 174 | GO:0006366 | transcription from RNA polymerase II promoter | 7 | 7 | 1.000000 | 1.000000 |
| 175 | GO:0006397 | mRNA processing | 7 | 7 | 1.000000 | 1.000000 |
| 176 | GO:0006820 | anion transport | 7 | 7 | 1.000000 | 1.000000 |
| 177 | GO:0009892 | negative regulation of metabolic process | 7 | 7 | 1.000000 | 1.000000 |
| 178 | GO:0010605 | negative regulation of macromolecule metabolic process | 7 | 7 | 1.000000 | 1.000000 |
| 179 | GO:0010629 | negative regulation of gene expression | 7 | 7 | 1.000000 | 1.000000 |
| 180 | GO:0016311 | dephosphorylation | 7 | 7 | 1.000000 | 1.000000 |
| 181 | GO:0016568 | chromatin modification | 7 | 7 | 1.000000 | 1.000000 |
| 182 | GO:0019941 | modification-dependent protein catabolic process | 7 | 7 | 1.000000 | 1.000000 |
| 183 | GO:0034613 | cellular protein localization | 7 | 7 | 1.000000 | 1.000000 |
| 184 | GO:0042254 | ribosome biogenesis | 7 | 7 | 1.000000 | 1.000000 |
| 185 | GO:0042592 | homeostatic process | 7 | 7 | 1.000000 | 1.000000 |
| 186 | GO:0044257 | cellular protein catabolic process | 7 | 7 | 1.000000 | 1.000000 |
| 187 | GO:0051603 | proteolysis involved in cellular protein catabolic process | 7 | 7 | 1.000000 | 1.000000 |
| 188 | GO:0070647 | protein modification by small protein conjugation or removal | 7 | 7 | 1.000000 | 1.000000 |
| 189 | GO:0070727 | cellular macromolecule localization | 7 | 7 | 1.000000 | 1.000000 |
| 190 | GO:1902580 | single-organism cellular localization | 7 | 7 | 1.000000 | 1.000000 |
| 191 | GO:1903047 | mitotic cell cycle process | 7 | 7 | 1.000000 | 1.000000 |
| 192 | GO:0006399 | tRNA metabolic process | 6 | 6 | 1.000000 | 1.000000 |
| 193 | GO:0006511 | ubiquitin-dependent protein catabolic process | 6 | 6 | 1.000000 | 1.000000 |
| 194 | GO:0006732 | coenzyme metabolic process | 6 | 6 | 1.000000 | 1.000000 |
| 195 | GO:0006886 | intracellular protein transport | 6 | 6 | 1.000000 | 1.000000 |
| 196 | GO:0006913 | nucleocytoplasmic transport | 6 | 6 | 1.000000 | 1.000000 |
| 197 | GO:0007010 | cytoskeleton organization | 6 | 6 | 1.000000 | 1.000000 |
| 198 | GO:0009116 | nucleoside metabolic process | 6 | 6 | 1.000000 | 1.000000 |
| 199 | GO:0009890 | negative regulation of biosynthetic process | 6 | 6 | 1.000000 | 1.000000 |
| 200 | GO:0010558 | negative regulation of macromolecule biosynthetic process | 6 | 6 | 1.000000 | 1.000000 |
| 201 | GO:0019693 | ribose phosphate metabolic process | 6 | 6 | 1.000000 | 1.000000 |
| 202 | GO:0022618 | ribonucleoprotein complex assembly | 6 | 6 | 1.000000 | 1.000000 |
| 203 | GO:0031324 | negative regulation of cellular metabolic process | 6 | 6 | 1.000000 | 1.000000 |
| 204 | GO:0031325 | positive regulation of cellular metabolic process | 6 | 6 | 1.000000 | 1.000000 |
| 205 | GO:0031327 | negative regulation of cellular biosynthetic process | 6 | 6 | 1.000000 | 1.000000 |
| 206 | GO:0034655 | nucleobase-containing compound catabolic process | 6 | 6 | 1.000000 | 1.000000 |
| 207 | GO:0043087 | regulation of GTPase activity | 6 | 6 | 1.000000 | 1.000000 |
| 208 | GO:0043414 | macromolecule methylation | 6 | 6 | 1.000000 | 1.000000 |
| 209 | GO:0044093 | positive regulation of molecular function | 6 | 6 | 1.000000 | 1.000000 |
| 210 | GO:0044282 | small molecule catabolic process | 6 | 6 | 1.000000 | 1.000000 |
| 211 | GO:0045892 | negative regulation of transcription, DNA-templated | 6 | 6 | 1.000000 | 1.000000 |
| 212 | GO:0045934 | negative regulation of nucleobase-containing compound metabolic process | 6 | 6 | 1.000000 | 1.000000 |
| 213 | GO:0051169 | nuclear transport | 6 | 6 | 1.000000 | 1.000000 |
| 214 | GO:0051172 | negative regulation of nitrogen compound metabolic process | 6 | 6 | 1.000000 | 1.000000 |
| 215 | GO:0051186 | cofactor metabolic process | 6 | 6 | 1.000000 | 1.000000 |
| 216 | GO:0051253 | negative regulation of RNA metabolic process | 6 | 6 | 1.000000 | 1.000000 |
| 217 | GO:0051640 | organelle localization | 6 | 6 | 1.000000 | 1.000000 |
| 218 | GO:0051656 | establishment of organelle localization | 6 | 6 | 1.000000 | 1.000000 |
| 219 | GO:0071103 | DNA conformation change | 6 | 6 | 1.000000 | 1.000000 |
| 220 | GO:0071822 | protein complex subunit organization | 6 | 6 | 1.000000 | 1.000000 |
| 221 | GO:0071826 | ribonucleoprotein complex subunit organization | 6 | 6 | 1.000000 | 1.000000 |
| 222 | GO:0090305 | nucleic acid phosphodiester bond hydrolysis | 6 | 6 | 1.000000 | 1.000000 |
| 223 | GO:0098655 | cation transmembrane transport | 6 | 6 | 1.000000 | 1.000000 |
| 224 | GO:0098660 | inorganic ion transmembrane transport | 6 | 6 | 1.000000 | 1.000000 |
| 225 | GO:1901657 | glycosyl compound metabolic process | 6 | 6 | 1.000000 | 1.000000 |
| 226 | GO:1902679 | negative regulation of RNA biosynthetic process | 6 | 6 | 1.000000 | 1.000000 |
| 227 | GO:1903507 | negative regulation of nucleic acid-templated transcription | 6 | 6 | 1.000000 | 1.000000 |
| 228 | GO:2000113 | negative regulation of cellular macromolecule biosynthetic process | 6 | 6 | 1.000000 | 1.000000 |
| 229 | GO:0000375 | RNA splicing, via transesterification reactions | 5 | 5 | 1.000000 | 1.000000 |
| 230 | GO:0000377 | RNA splicing, via transesterification reactions with bulged adenosine as nucleophile | 5 | 5 | 1.000000 | 1.000000 |
| 231 | GO:0000398 | mRNA splicing, via spliceosome | 5 | 5 | 1.000000 | 1.000000 |
| 232 | GO:0005976 | polysaccharide metabolic process | 5 | 5 | 1.000000 | 1.000000 |
| 233 | GO:0006091 | generation of precursor metabolites and energy | 5 | 5 | 1.000000 | 1.000000 |
| 234 | GO:0006401 | RNA catabolic process | 5 | 5 | 1.000000 | 1.000000 |
| 235 | GO:0006470 | protein dephosphorylation | 5 | 5 | 1.000000 | 1.000000 |
| 236 | GO:0006479 | protein methylation | 5 | 5 | 1.000000 | 1.000000 |
| 237 | GO:0006644 | phospholipid metabolic process | 5 | 5 | 1.000000 | 1.000000 |
| 238 | GO:0006733 | oxidoreduction coenzyme metabolic process | 5 | 5 | 1.000000 | 1.000000 |
| 239 | GO:0008213 | protein alkylation | 5 | 5 | 1.000000 | 1.000000 |
| 240 | GO:0008380 | RNA splicing | 5 | 5 | 1.000000 | 1.000000 |
| 241 | GO:0009072 | aromatic amino acid family metabolic process | 5 | 5 | 1.000000 | 1.000000 |
| 242 | GO:0010604 | positive regulation of macromolecule metabolic process | 5 | 5 | 1.000000 | 1.000000 |
| 243 | GO:0015698 | inorganic anion transport | 5 | 5 | 1.000000 | 1.000000 |
| 244 | GO:0015980 | energy derivation by oxidation of organic compounds | 5 | 5 | 1.000000 | 1.000000 |
| 245 | GO:0016052 | carbohydrate catabolic process | 5 | 5 | 1.000000 | 1.000000 |
| 246 | GO:0019362 | pyridine nucleotide metabolic process | 5 | 5 | 1.000000 | 1.000000 |
| 247 | GO:0030001 | metal ion transport | 5 | 5 | 1.000000 | 1.000000 |
| 248 | GO:0032268 | regulation of cellular protein metabolic process | 5 | 5 | 1.000000 | 1.000000 |
| 249 | GO:0032446 | protein modification by small protein conjugation | 5 | 5 | 1.000000 | 1.000000 |
| 250 | GO:0043085 | positive regulation of catalytic activity | 5 | 5 | 1.000000 | 1.000000 |
| 251 | GO:0046496 | nicotinamide nucleotide metabolic process | 5 | 5 | 1.000000 | 1.000000 |
| 252 | GO:0051128 | regulation of cellular component organization | 5 | 5 | 1.000000 | 1.000000 |
| 253 | GO:0051246 | regulation of protein metabolic process | 5 | 5 | 1.000000 | 1.000000 |
| 254 | GO:0071705 | nitrogen compound transport | 5 | 5 | 1.000000 | 1.000000 |
| 255 | GO:0072521 | purine-containing compound metabolic process | 5 | 5 | 1.000000 | 1.000000 |
| 256 | GO:0072524 | pyridine-containing compound metabolic process | 5 | 5 | 1.000000 | 1.000000 |
| 257 | GO:0098662 | inorganic cation transmembrane transport | 5 | 5 | 1.000000 | 1.000000 |
| 258 | GO:0000272 | polysaccharide catabolic process | 4 | 4 | 1.000000 | 1.000000 |
| 259 | GO:0000280 | nuclear division | 4 | 4 | 1.000000 | 1.000000 |
| 260 | GO:0006338 | chromatin remodeling | 4 | 4 | 1.000000 | 1.000000 |
| 261 | GO:0006364 | rRNA processing | 4 | 4 | 1.000000 | 1.000000 |
| 262 | GO:0006457 | protein folding | 4 | 4 | 1.000000 | 1.000000 |
| 263 | GO:0006605 | protein targeting | 4 | 4 | 1.000000 | 1.000000 |
| 264 | GO:0006897 | endocytosis | 4 | 4 | 1.000000 | 1.000000 |
| 265 | GO:0007005 | mitochondrion organization | 4 | 4 | 1.000000 | 1.000000 |
| 266 | GO:0007017 | microtubule-based process | 4 | 4 | 1.000000 | 1.000000 |
| 267 | GO:0007034 | vacuolar transport | 4 | 4 | 1.000000 | 1.000000 |
| 268 | GO:0008654 | phospholipid biosynthetic process | 4 | 4 | 1.000000 | 1.000000 |
| 269 | GO:0009084 | glutamine family amino acid biosynthetic process | 4 | 4 | 1.000000 | 1.000000 |
| 270 | GO:0009112 | nucleobase metabolic process | 4 | 4 | 1.000000 | 1.000000 |
| 271 | GO:0009123 | nucleoside monophosphate metabolic process | 4 | 4 | 1.000000 | 1.000000 |
| 272 | GO:0009161 | ribonucleoside monophosphate metabolic process | 4 | 4 | 1.000000 | 1.000000 |
| 273 | GO:0009259 | ribonucleotide metabolic process | 4 | 4 | 1.000000 | 1.000000 |
| 274 | GO:0009451 | RNA modification | 4 | 4 | 1.000000 | 1.000000 |
| 275 | GO:0015672 | monovalent inorganic cation transport | 4 | 4 | 1.000000 | 1.000000 |
| 276 | GO:0015931 | nucleobase-containing compound transport | 4 | 4 | 1.000000 | 1.000000 |
| 277 | GO:0016054 | organic acid catabolic process | 4 | 4 | 1.000000 | 1.000000 |
| 278 | GO:0016072 | rRNA metabolic process | 4 | 4 | 1.000000 | 1.000000 |
| 279 | GO:0016458 | gene silencing | 4 | 4 | 1.000000 | 1.000000 |
| 280 | GO:0016567 | protein ubiquitination | 4 | 4 | 1.000000 | 1.000000 |
| 281 | GO:0032787 | monocarboxylic acid metabolic process | 4 | 4 | 1.000000 | 1.000000 |
| 282 | GO:0033365 | protein localization to organelle | 4 | 4 | 1.000000 | 1.000000 |
| 283 | GO:0035556 | intracellular signal transduction | 4 | 4 | 1.000000 | 1.000000 |
| 284 | GO:0046395 | carboxylic acid catabolic process | 4 | 4 | 1.000000 | 1.000000 |
| 285 | GO:0046486 | glycerolipid metabolic process | 4 | 4 | 1.000000 | 1.000000 |
| 286 | GO:0048285 | organelle fission | 4 | 4 | 1.000000 | 1.000000 |
| 287 | GO:0048878 | chemical homeostasis | 4 | 4 | 1.000000 | 1.000000 |
| 288 | GO:0051052 | regulation of DNA metabolic process | 4 | 4 | 1.000000 | 1.000000 |
| 289 | GO:0051168 | nuclear export | 4 | 4 | 1.000000 | 1.000000 |
| 290 | GO:0051173 | positive regulation of nitrogen compound metabolic process | 4 | 4 | 1.000000 | 1.000000 |
| 291 | GO:0051188 | cofactor biosynthetic process | 4 | 4 | 1.000000 | 1.000000 |
| 292 | GO:0051301 | cell division | 4 | 4 | 1.000000 | 1.000000 |
| 293 | GO:0051345 | positive regulation of hydrolase activity | 4 | 4 | 1.000000 | 1.000000 |
| 294 | GO:0070085 | glycosylation | 4 | 4 | 1.000000 | 1.000000 |
| 295 | GO:0071166 | ribonucleoprotein complex localization | 4 | 4 | 1.000000 | 1.000000 |
| 296 | GO:0071426 | ribonucleoprotein complex export from nucleus | 4 | 4 | 1.000000 | 1.000000 |
| 297 | GO:0072594 | establishment of protein localization to organelle | 4 | 4 | 1.000000 | 1.000000 |
| 298 | GO:1901565 | organonitrogen compound catabolic process | 4 | 4 | 1.000000 | 1.000000 |
| 299 | GO:0000003 | reproduction | 3 | 3 | 1.000000 | 1.000000 |
| 300 | GO:0000018 | regulation of DNA recombination | 3 | 3 | 1.000000 | 1.000000 |
| 301 | GO:0000019 | regulation of mitotic recombination | 3 | 3 | 1.000000 | 1.000000 |
| 302 | GO:0000070 | mitotic sister chromatid segregation | 3 | 3 | 1.000000 | 1.000000 |
| 303 | GO:0000096 | sulfur amino acid metabolic process | 3 | 3 | 1.000000 | 1.000000 |
| 304 | GO:0000097 | sulfur amino acid biosynthetic process | 3 | 3 | 1.000000 | 1.000000 |
| 305 | GO:0000226 | microtubule cytoskeleton organization | 3 | 3 | 1.000000 | 1.000000 |
| 306 | GO:0000413 | protein peptidyl-prolyl isomerization | 3 | 3 | 1.000000 | 1.000000 |
| 307 | GO:0000819 | sister chromatid segregation | 3 | 3 | 1.000000 | 1.000000 |
| 308 | GO:0002181 | cytoplasmic translation | 3 | 3 | 1.000000 | 1.000000 |
| 309 | GO:0002183 | cytoplasmic translational initiation | 3 | 3 | 1.000000 | 1.000000 |
| 310 | GO:0005996 | monosaccharide metabolic process | 3 | 3 | 1.000000 | 1.000000 |
| 311 | GO:0006073 | cellular glucan metabolic process | 3 | 3 | 1.000000 | 1.000000 |
| 312 | GO:0006081 | cellular aldehyde metabolic process | 3 | 3 | 1.000000 | 1.000000 |
| 313 | GO:0006144 | purine nucleobase metabolic process | 3 | 3 | 1.000000 | 1.000000 |
| 314 | GO:0006163 | purine nucleotide metabolic process | 3 | 3 | 1.000000 | 1.000000 |
| 315 | GO:0006289 | nucleotide-excision repair | 3 | 3 | 1.000000 | 1.000000 |
| 316 | GO:0006323 | DNA packaging | 3 | 3 | 1.000000 | 1.000000 |
| 317 | GO:0006342 | chromatin silencing | 3 | 3 | 1.000000 | 1.000000 |
| 318 | GO:0006352 | DNA-templated transcription, initiation | 3 | 3 | 1.000000 | 1.000000 |
| 319 | GO:0006367 | transcription initiation from RNA polymerase II promoter | 3 | 3 | 1.000000 | 1.000000 |
| 320 | GO:0006417 | regulation of translation | 3 | 3 | 1.000000 | 1.000000 |
| 321 | GO:0006418 | tRNA aminoacylation for protein translation | 3 | 3 | 1.000000 | 1.000000 |
| 322 | GO:0006461 | protein complex assembly | 3 | 3 | 1.000000 | 1.000000 |
| 323 | GO:0006525 | arginine metabolic process | 3 | 3 | 1.000000 | 1.000000 |
| 324 | GO:0006541 | glutamine metabolic process | 3 | 3 | 1.000000 | 1.000000 |
| 325 | GO:0006568 | tryptophan metabolic process | 3 | 3 | 1.000000 | 1.000000 |
| 326 | GO:0006576 | cellular biogenic amine metabolic process | 3 | 3 | 1.000000 | 1.000000 |
| 327 | GO:0006586 | indolalkylamine metabolic process | 3 | 3 | 1.000000 | 1.000000 |
| 328 | GO:0006643 | membrane lipid metabolic process | 3 | 3 | 1.000000 | 1.000000 |
| 329 | GO:0006650 | glycerophospholipid metabolic process | 3 | 3 | 1.000000 | 1.000000 |
| 330 | GO:0006790 | sulfur compound metabolic process | 3 | 3 | 1.000000 | 1.000000 |
| 331 | GO:0006818 | hydrogen transport | 3 | 3 | 1.000000 | 1.000000 |
| 332 | GO:0006887 | exocytosis | 3 | 3 | 1.000000 | 1.000000 |
| 333 | GO:0006979 | response to oxidative stress | 3 | 3 | 1.000000 | 1.000000 |
| 334 | GO:0007059 | chromosome segregation | 3 | 3 | 1.000000 | 1.000000 |
| 335 | GO:0007067 | mitotic nuclear division | 3 | 3 | 1.000000 | 1.000000 |
| 336 | GO:0007076 | mitotic chromosome condensation | 3 | 3 | 1.000000 | 1.000000 |
| 337 | GO:0008033 | tRNA processing | 3 | 3 | 1.000000 | 1.000000 |
| 338 | GO:0009063 | cellular amino acid catabolic process | 3 | 3 | 1.000000 | 1.000000 |
| 339 | GO:0009108 | coenzyme biosynthetic process | 3 | 3 | 1.000000 | 1.000000 |
| 340 | GO:0009119 | ribonucleoside metabolic process | 3 | 3 | 1.000000 | 1.000000 |
| 341 | GO:0009124 | nucleoside monophosphate biosynthetic process | 3 | 3 | 1.000000 | 1.000000 |
| 342 | GO:0009126 | purine nucleoside monophosphate metabolic process | 3 | 3 | 1.000000 | 1.000000 |
| 343 | GO:0009150 | purine ribonucleotide metabolic process | 3 | 3 | 1.000000 | 1.000000 |
| 344 | GO:0009156 | ribonucleoside monophosphate biosynthetic process | 3 | 3 | 1.000000 | 1.000000 |
| 345 | GO:0009167 | purine ribonucleoside monophosphate metabolic process | 3 | 3 | 1.000000 | 1.000000 |
| 346 | GO:0009260 | ribonucleotide biosynthetic process | 3 | 3 | 1.000000 | 1.000000 |
| 347 | GO:0009308 | amine metabolic process | 3 | 3 | 1.000000 | 1.000000 |
| 348 | GO:0009435 | NAD biosynthetic process | 3 | 3 | 1.000000 | 1.000000 |
| 349 | GO:0009891 | positive regulation of biosynthetic process | 3 | 3 | 1.000000 | 1.000000 |
| 350 | GO:0009966 | regulation of signal transduction | 3 | 3 | 1.000000 | 1.000000 |
| 351 | GO:0010557 | positive regulation of macromolecule biosynthetic process | 3 | 3 | 1.000000 | 1.000000 |
| 352 | GO:0010608 | posttranscriptional regulation of gene expression | 3 | 3 | 1.000000 | 1.000000 |
| 353 | GO:0010628 | positive regulation of gene expression | 3 | 3 | 1.000000 | 1.000000 |
| 354 | GO:0010646 | regulation of cell communication | 3 | 3 | 1.000000 | 1.000000 |
| 355 | GO:0015893 | drug transport | 3 | 3 | 1.000000 | 1.000000 |
| 356 | GO:0015992 | proton transport | 3 | 3 | 1.000000 | 1.000000 |
| 357 | GO:0016051 | carbohydrate biosynthetic process | 3 | 3 | 1.000000 | 1.000000 |
| 358 | GO:0016569 | covalent chromatin modification | 3 | 3 | 1.000000 | 1.000000 |
| 359 | GO:0016570 | histone modification | 3 | 3 | 1.000000 | 1.000000 |
| 360 | GO:0018208 | peptidyl-proline modification | 3 | 3 | 1.000000 | 1.000000 |
| 361 | GO:0019318 | hexose metabolic process | 3 | 3 | 1.000000 | 1.000000 |
| 362 | GO:0019359 | nicotinamide nucleotide biosynthetic process | 3 | 3 | 1.000000 | 1.000000 |
| 363 | GO:0019363 | pyridine nucleotide biosynthetic process | 3 | 3 | 1.000000 | 1.000000 |
| 364 | GO:0019674 | NAD metabolic process | 3 | 3 | 1.000000 | 1.000000 |
| 365 | GO:0023051 | regulation of signaling | 3 | 3 | 1.000000 | 1.000000 |
| 366 | GO:0030258 | lipid modification | 3 | 3 | 1.000000 | 1.000000 |
| 367 | GO:0030261 | chromosome condensation | 3 | 3 | 1.000000 | 1.000000 |
| 368 | GO:0031328 | positive regulation of cellular biosynthetic process | 3 | 3 | 1.000000 | 1.000000 |
| 369 | GO:0032270 | positive regulation of cellular protein metabolic process | 3 | 3 | 1.000000 | 1.000000 |
| 370 | GO:0032392 | DNA geometric change | 3 | 3 | 1.000000 | 1.000000 |
| 371 | GO:0032940 | secretion by cell | 3 | 3 | 1.000000 | 1.000000 |
| 372 | GO:0034248 | regulation of cellular amide metabolic process | 3 | 3 | 1.000000 | 1.000000 |
| 373 | GO:0035023 | regulation of Rho protein signal transduction | 3 | 3 | 1.000000 | 1.000000 |
| 374 | GO:0040029 | regulation of gene expression, epigenetic | 3 | 3 | 1.000000 | 1.000000 |
| 375 | GO:0042430 | indole-containing compound metabolic process | 3 | 3 | 1.000000 | 1.000000 |
| 376 | GO:0042493 | response to drug | 3 | 3 | 1.000000 | 1.000000 |
| 377 | GO:0043038 | amino acid activation | 3 | 3 | 1.000000 | 1.000000 |
| 378 | GO:0043039 | tRNA aminoacylation | 3 | 3 | 1.000000 | 1.000000 |
| 379 | GO:0043254 | regulation of protein complex assembly | 3 | 3 | 1.000000 | 1.000000 |
| 380 | GO:0043623 | cellular protein complex assembly | 3 | 3 | 1.000000 | 1.000000 |
| 381 | GO:0043648 | dicarboxylic acid metabolic process | 3 | 3 | 1.000000 | 1.000000 |
| 382 | GO:0043650 | dicarboxylic acid biosynthetic process | 3 | 3 | 1.000000 | 1.000000 |
| 383 | GO:0044042 | glucan metabolic process | 3 | 3 | 1.000000 | 1.000000 |
| 384 | GO:0044087 | regulation of cellular component biogenesis | 3 | 3 | 1.000000 | 1.000000 |
| 385 | GO:0044106 | cellular amine metabolic process | 3 | 3 | 1.000000 | 1.000000 |
| 386 | GO:0044264 | cellular polysaccharide metabolic process | 3 | 3 | 1.000000 | 1.000000 |
| 387 | GO:0044272 | sulfur compound biosynthetic process | 3 | 3 | 1.000000 | 1.000000 |
| 388 | GO:0044275 | cellular carbohydrate catabolic process | 3 | 3 | 1.000000 | 1.000000 |
| 389 | GO:0045017 | glycerolipid biosynthetic process | 3 | 3 | 1.000000 | 1.000000 |
| 390 | GO:0045333 | cellular respiration | 3 | 3 | 1.000000 | 1.000000 |
| 391 | GO:0045814 | negative regulation of gene expression, epigenetic | 3 | 3 | 1.000000 | 1.000000 |
| 392 | GO:0046112 | nucleobase biosynthetic process | 3 | 3 | 1.000000 | 1.000000 |
| 393 | GO:0046390 | ribose phosphate biosynthetic process | 3 | 3 | 1.000000 | 1.000000 |
| 394 | GO:0046467 | membrane lipid biosynthetic process | 3 | 3 | 1.000000 | 1.000000 |
| 395 | GO:0046474 | glycerophospholipid biosynthetic process | 3 | 3 | 1.000000 | 1.000000 |
| 396 | GO:0046578 | regulation of Ras protein signal transduction | 3 | 3 | 1.000000 | 1.000000 |
| 397 | GO:0046903 | secretion | 3 | 3 | 1.000000 | 1.000000 |
| 398 | GO:0048583 | regulation of response to stimulus | 3 | 3 | 1.000000 | 1.000000 |
| 399 | GO:0050801 | ion homeostasis | 3 | 3 | 1.000000 | 1.000000 |
| 400 | GO:0051056 | regulation of small GTPase mediated signal transduction | 3 | 3 | 1.000000 | 1.000000 |
| 401 | GO:0051247 | positive regulation of protein metabolic process | 3 | 3 | 1.000000 | 1.000000 |
| 402 | GO:0055080 | cation homeostasis | 3 | 3 | 1.000000 | 1.000000 |
| 403 | GO:0070271 | protein complex biogenesis | 3 | 3 | 1.000000 | 1.000000 |
| 404 | GO:0070887 | cellular response to chemical stimulus | 3 | 3 | 1.000000 | 1.000000 |
| 405 | GO:0072525 | pyridine-containing compound biosynthetic process | 3 | 3 | 1.000000 | 1.000000 |
| 406 | GO:0098656 | anion transmembrane transport | 3 | 3 | 1.000000 | 1.000000 |
| 407 | GO:0098771 | inorganic ion homeostasis | 3 | 3 | 1.000000 | 1.000000 |
| 408 | GO:0098813 | nuclear chromosome segregation | 3 | 3 | 1.000000 | 1.000000 |
| 409 | GO:1901606 | alpha-amino acid catabolic process | 3 | 3 | 1.000000 | 1.000000 |
| 410 | GO:1902531 | regulation of intracellular signal transduction | 3 | 3 | 1.000000 | 1.000000 |
| 411 | GO:1902600 | hydrogen ion transmembrane transport | 3 | 3 | 1.000000 | 1.000000 |
| 412 | GO:0000054 | ribosomal subunit export from nucleus | 2 | 2 | 1.000000 | 1.000000 |
| 413 | GO:0000082 | G1/S transition of mitotic cell cycle | 2 | 2 | 1.000000 | 1.000000 |
| 414 | GO:0000103 | sulfate assimilation | 2 | 2 | 1.000000 | 1.000000 |
| 415 | GO:0000393 | spliceosomal conformational changes to generate catalytic conformation | 2 | 2 | 1.000000 | 1.000000 |
| 416 | GO:0000723 | telomere maintenance | 2 | 2 | 1.000000 | 1.000000 |
| 417 | GO:0000956 | nuclear-transcribed mRNA catabolic process | 2 | 2 | 1.000000 | 1.000000 |
| 418 | GO:0001112 | DNA-templated transcriptional open complex formation | 2 | 2 | 1.000000 | 1.000000 |
| 419 | GO:0001113 | transcriptional open complex formation at RNA polymerase II promoter | 2 | 2 | 1.000000 | 1.000000 |
| 420 | GO:0001120 | protein-DNA complex remodeling | 2 | 2 | 1.000000 | 1.000000 |
| 421 | GO:0001732 | formation of cytoplasmic translation initiation complex | 2 | 2 | 1.000000 | 1.000000 |
| 422 | GO:0001932 | regulation of protein phosphorylation | 2 | 2 | 1.000000 | 1.000000 |
| 423 | GO:0005977 | glycogen metabolic process | 2 | 2 | 1.000000 | 1.000000 |
| 424 | GO:0005984 | disaccharide metabolic process | 2 | 2 | 1.000000 | 1.000000 |
| 425 | GO:0005991 | trehalose metabolic process | 2 | 2 | 1.000000 | 1.000000 |
| 426 | GO:0006066 | alcohol metabolic process | 2 | 2 | 1.000000 | 1.000000 |
| 427 | GO:0006098 | pentose-phosphate shunt | 2 | 2 | 1.000000 | 1.000000 |
| 428 | GO:0006099 | tricarboxylic acid cycle | 2 | 2 | 1.000000 | 1.000000 |
| 429 | GO:0006101 | citrate metabolic process | 2 | 2 | 1.000000 | 1.000000 |
| 430 | GO:0006112 | energy reserve metabolic process | 2 | 2 | 1.000000 | 1.000000 |
| 431 | GO:0006164 | purine nucleotide biosynthetic process | 2 | 2 | 1.000000 | 1.000000 |
| 432 | GO:0006188 | IMP biosynthetic process | 2 | 2 | 1.000000 | 1.000000 |
| 433 | GO:0006189 | 'de novo' IMP biosynthetic process | 2 | 2 | 1.000000 | 1.000000 |
| 434 | GO:0006261 | DNA-dependent DNA replication | 2 | 2 | 1.000000 | 1.000000 |
| 435 | GO:0006265 | DNA topological change | 2 | 2 | 1.000000 | 1.000000 |
| 436 | GO:0006271 | DNA strand elongation involved in DNA replication | 2 | 2 | 1.000000 | 1.000000 |
| 437 | GO:0006310 | DNA recombination | 2 | 2 | 1.000000 | 1.000000 |
| 438 | GO:0006333 | chromatin assembly or disassembly | 2 | 2 | 1.000000 | 1.000000 |
| 439 | GO:0006354 | DNA-templated transcription, elongation | 2 | 2 | 1.000000 | 1.000000 |
| 440 | GO:0006368 | transcription elongation from RNA polymerase II promoter | 2 | 2 | 1.000000 | 1.000000 |
| 441 | GO:0006400 | tRNA modification | 2 | 2 | 1.000000 | 1.000000 |
| 442 | GO:0006402 | mRNA catabolic process | 2 | 2 | 1.000000 | 1.000000 |
| 443 | GO:0006403 | RNA localization | 2 | 2 | 1.000000 | 1.000000 |
| 444 | GO:0006405 | RNA export from nucleus | 2 | 2 | 1.000000 | 1.000000 |
| 445 | GO:0006414 | translational elongation | 2 | 2 | 1.000000 | 1.000000 |
| 446 | GO:0006526 | arginine biosynthetic process | 2 | 2 | 1.000000 | 1.000000 |
| 447 | GO:0006569 | tryptophan catabolic process | 2 | 2 | 1.000000 | 1.000000 |
| 448 | GO:0006623 | protein targeting to vacuole | 2 | 2 | 1.000000 | 1.000000 |
| 449 | GO:0006631 | fatty acid metabolic process | 2 | 2 | 1.000000 | 1.000000 |
| 450 | GO:0006633 | fatty acid biosynthetic process | 2 | 2 | 1.000000 | 1.000000 |
| 451 | GO:0006661 | phosphatidylinositol biosynthetic process | 2 | 2 | 1.000000 | 1.000000 |
| 452 | GO:0006664 | glycolipid metabolic process | 2 | 2 | 1.000000 | 1.000000 |
| 453 | GO:0006665 | sphingolipid metabolic process | 2 | 2 | 1.000000 | 1.000000 |
| 454 | GO:0006673 | inositolphosphoceramide metabolic process | 2 | 2 | 1.000000 | 1.000000 |
| 455 | GO:0006739 | NADP metabolic process | 2 | 2 | 1.000000 | 1.000000 |
| 456 | GO:0006797 | polyphosphate metabolic process | 2 | 2 | 1.000000 | 1.000000 |
| 457 | GO:0006839 | mitochondrial transport | 2 | 2 | 1.000000 | 1.000000 |
| 458 | GO:0006885 | regulation of pH | 2 | 2 | 1.000000 | 1.000000 |
| 459 | GO:0006903 | vesicle targeting | 2 | 2 | 1.000000 | 1.000000 |
| 460 | GO:0006904 | vesicle docking involved in exocytosis | 2 | 2 | 1.000000 | 1.000000 |
| 461 | GO:0007033 | vacuole organization | 2 | 2 | 1.000000 | 1.000000 |
| 462 | GO:0007126 | meiotic nuclear division | 2 | 2 | 1.000000 | 1.000000 |
| 463 | GO:0007127 | meiosis I | 2 | 2 | 1.000000 | 1.000000 |
| 464 | GO:0007131 | reciprocal meiotic recombination | 2 | 2 | 1.000000 | 1.000000 |
| 465 | GO:0007186 | G-protein coupled receptor signaling pathway | 2 | 2 | 1.000000 | 1.000000 |
| 466 | GO:0007346 | regulation of mitotic cell cycle | 2 | 2 | 1.000000 | 1.000000 |
| 467 | GO:0008064 | regulation of actin polymerization or depolymerization | 2 | 2 | 1.000000 | 1.000000 |
| 468 | GO:0008643 | carbohydrate transport | 2 | 2 | 1.000000 | 1.000000 |
| 469 | GO:0009060 | aerobic respiration | 2 | 2 | 1.000000 | 1.000000 |
| 470 | GO:0009069 | serine family amino acid metabolic process | 2 | 2 | 1.000000 | 1.000000 |
| 471 | GO:0009073 | aromatic amino acid family biosynthetic process | 2 | 2 | 1.000000 | 1.000000 |
| 472 | GO:0009074 | aromatic amino acid family catabolic process | 2 | 2 | 1.000000 | 1.000000 |
| 473 | GO:0009081 | branched-chain amino acid metabolic process | 2 | 2 | 1.000000 | 1.000000 |
| 474 | GO:0009082 | branched-chain amino acid biosynthetic process | 2 | 2 | 1.000000 | 1.000000 |
| 475 | GO:0009113 | purine nucleobase biosynthetic process | 2 | 2 | 1.000000 | 1.000000 |
| 476 | GO:0009127 | purine nucleoside monophosphate biosynthetic process | 2 | 2 | 1.000000 | 1.000000 |
| 477 | GO:0009141 | nucleoside triphosphate metabolic process | 2 | 2 | 1.000000 | 1.000000 |
| 478 | GO:0009152 | purine ribonucleotide biosynthetic process | 2 | 2 | 1.000000 | 1.000000 |
| 479 | GO:0009168 | purine ribonucleoside monophosphate biosynthetic process | 2 | 2 | 1.000000 | 1.000000 |
| 480 | GO:0009199 | ribonucleoside triphosphate metabolic process | 2 | 2 | 1.000000 | 1.000000 |
| 481 | GO:0009225 | nucleotide-sugar metabolic process | 2 | 2 | 1.000000 | 1.000000 |
| 482 | GO:0009247 | glycolipid biosynthetic process | 2 | 2 | 1.000000 | 1.000000 |
| 483 | GO:0009251 | glucan catabolic process | 2 | 2 | 1.000000 | 1.000000 |
| 484 | GO:0009310 | amine catabolic process | 2 | 2 | 1.000000 | 1.000000 |
| 485 | GO:0009311 | oligosaccharide metabolic process | 2 | 2 | 1.000000 | 1.000000 |
| 486 | GO:0009636 | response to toxic substance | 2 | 2 | 1.000000 | 1.000000 |
| 487 | GO:0015074 | DNA integration | 2 | 2 | 1.000000 | 1.000000 |
| 488 | GO:0015711 | organic anion transport | 2 | 2 | 1.000000 | 1.000000 |
| 489 | GO:0015849 | organic acid transport | 2 | 2 | 1.000000 | 1.000000 |
| 490 | GO:0016197 | endosomal transport | 2 | 2 | 1.000000 | 1.000000 |
| 491 | GO:0016571 | histone methylation | 2 | 2 | 1.000000 | 1.000000 |
| 492 | GO:0016579 | protein deubiquitination | 2 | 2 | 1.000000 | 1.000000 |
| 493 | GO:0017004 | cytochrome complex assembly | 2 | 2 | 1.000000 | 1.000000 |
| 494 | GO:0017038 | protein import | 2 | 2 | 1.000000 | 1.000000 |
| 495 | GO:0017062 | respiratory chain complex III assembly | 2 | 2 | 1.000000 | 1.000000 |
| 496 | GO:0018022 | peptidyl-lysine methylation | 2 | 2 | 1.000000 | 1.000000 |
| 497 | GO:0018205 | peptidyl-lysine modification | 2 | 2 | 1.000000 | 1.000000 |
| 498 | GO:0019220 | regulation of phosphate metabolic process | 2 | 2 | 1.000000 | 1.000000 |
| 499 | GO:0019673 | GDP-mannose metabolic process | 2 | 2 | 1.000000 | 1.000000 |
| 500 | GO:0019682 | glyceraldehyde-3-phosphate metabolic process | 2 | 2 | 1.000000 | 1.000000 |
| 501 | GO:0019725 | cellular homeostasis | 2 | 2 | 1.000000 | 1.000000 |
| 502 | GO:0019751 | polyol metabolic process | 2 | 2 | 1.000000 | 1.000000 |
| 503 | GO:0019932 | second-messenger-mediated signaling | 2 | 2 | 1.000000 | 1.000000 |
| 504 | GO:0022406 | membrane docking | 2 | 2 | 1.000000 | 1.000000 |
| 505 | GO:0022414 | reproductive process | 2 | 2 | 1.000000 | 1.000000 |
| 506 | GO:0022616 | DNA strand elongation | 2 | 2 | 1.000000 | 1.000000 |
| 507 | GO:0030029 | actin filament-based process | 2 | 2 | 1.000000 | 1.000000 |
| 508 | GO:0030036 | actin cytoskeleton organization | 2 | 2 | 1.000000 | 1.000000 |
| 509 | GO:0030148 | sphingolipid biosynthetic process | 2 | 2 | 1.000000 | 1.000000 |
| 510 | GO:0030259 | lipid glycosylation | 2 | 2 | 1.000000 | 1.000000 |
| 511 | GO:0030832 | regulation of actin filament length | 2 | 2 | 1.000000 | 1.000000 |
| 512 | GO:0030833 | regulation of actin filament polymerization | 2 | 2 | 1.000000 | 1.000000 |
| 513 | GO:0031047 | gene silencing by RNA | 2 | 2 | 1.000000 | 1.000000 |
| 514 | GO:0031399 | regulation of protein modification process | 2 | 2 | 1.000000 | 1.000000 |
| 515 | GO:0032200 | telomere organization | 2 | 2 | 1.000000 | 1.000000 |
| 516 | GO:0032271 | regulation of protein polymerization | 2 | 2 | 1.000000 | 1.000000 |
| 517 | GO:0032508 | DNA duplex unwinding | 2 | 2 | 1.000000 | 1.000000 |
| 518 | GO:0032535 | regulation of cellular component size | 2 | 2 | 1.000000 | 1.000000 |
| 519 | GO:0032956 | regulation of actin cytoskeleton organization | 2 | 2 | 1.000000 | 1.000000 |
| 520 | GO:0032970 | regulation of actin filament-based process | 2 | 2 | 1.000000 | 1.000000 |
| 521 | GO:0033043 | regulation of organelle organization | 2 | 2 | 1.000000 | 1.000000 |
| 522 | GO:0033108 | mitochondrial respiratory chain complex assembly | 2 | 2 | 1.000000 | 1.000000 |
| 523 | GO:0033750 | ribosome localization | 2 | 2 | 1.000000 | 1.000000 |
| 524 | GO:0033753 | establishment of ribosome localization | 2 | 2 | 1.000000 | 1.000000 |
| 525 | GO:0034250 | positive regulation of cellular amide metabolic process | 2 | 2 | 1.000000 | 1.000000 |
| 526 | GO:0034367 | macromolecular complex remodeling | 2 | 2 | 1.000000 | 1.000000 |
| 527 | GO:0034551 | mitochondrial respiratory chain complex III assembly | 2 | 2 | 1.000000 | 1.000000 |
| 528 | GO:0034599 | cellular response to oxidative stress | 2 | 2 | 1.000000 | 1.000000 |
| 529 | GO:0034637 | cellular carbohydrate biosynthetic process | 2 | 2 | 1.000000 | 1.000000 |
| 530 | GO:0034968 | histone lysine methylation | 2 | 2 | 1.000000 | 1.000000 |
| 531 | GO:0035335 | peptidyl-tyrosine dephosphorylation | 2 | 2 | 1.000000 | 1.000000 |
| 532 | GO:0035825 | reciprocal DNA recombination | 2 | 2 | 1.000000 | 1.000000 |
| 533 | GO:0042278 | purine nucleoside metabolic process | 2 | 2 | 1.000000 | 1.000000 |
| 534 | GO:0042325 | regulation of phosphorylation | 2 | 2 | 1.000000 | 1.000000 |
| 535 | GO:0042402 | cellular biogenic amine catabolic process | 2 | 2 | 1.000000 | 1.000000 |
| 536 | GO:0042436 | indole-containing compound catabolic process | 2 | 2 | 1.000000 | 1.000000 |
| 537 | GO:0042440 | pigment metabolic process | 2 | 2 | 1.000000 | 1.000000 |
| 538 | GO:0042537 | benzene-containing compound metabolic process | 2 | 2 | 1.000000 | 1.000000 |
| 539 | GO:0043044 | ATP-dependent chromatin remodeling | 2 | 2 | 1.000000 | 1.000000 |
| 540 | GO:0043547 | positive regulation of GTPase activity | 2 | 2 | 1.000000 | 1.000000 |
| 541 | GO:0043549 | regulation of kinase activity | 2 | 2 | 1.000000 | 1.000000 |
| 542 | GO:0044247 | cellular polysaccharide catabolic process | 2 | 2 | 1.000000 | 1.000000 |
| 543 | GO:0044702 | single organism reproductive process | 2 | 2 | 1.000000 | 1.000000 |
| 544 | GO:0044724 | single-organism carbohydrate catabolic process | 2 | 2 | 1.000000 | 1.000000 |
| 545 | GO:0044770 | cell cycle phase transition | 2 | 2 | 1.000000 | 1.000000 |
| 546 | GO:0044772 | mitotic cell cycle phase transition | 2 | 2 | 1.000000 | 1.000000 |
| 547 | GO:0044802 | single-organism membrane organization | 2 | 2 | 1.000000 | 1.000000 |
| 548 | GO:0044843 | cell cycle G1/S phase transition | 2 | 2 | 1.000000 | 1.000000 |
| 549 | GO:0045727 | positive regulation of translation | 2 | 2 | 1.000000 | 1.000000 |
| 550 | GO:0045786 | negative regulation of cell cycle | 2 | 2 | 1.000000 | 1.000000 |
| 551 | GO:0045859 | regulation of protein kinase activity | 2 | 2 | 1.000000 | 1.000000 |
| 552 | GO:0045930 | negative regulation of mitotic cell cycle | 2 | 2 | 1.000000 | 1.000000 |
| 553 | GO:0045935 | positive regulation of nucleobase-containing compound metabolic process | 2 | 2 | 1.000000 | 1.000000 |
| 554 | GO:0046040 | IMP metabolic process | 2 | 2 | 1.000000 | 1.000000 |
| 555 | GO:0046128 | purine ribonucleoside metabolic process | 2 | 2 | 1.000000 | 1.000000 |
| 556 | GO:0046148 | pigment biosynthetic process | 2 | 2 | 1.000000 | 1.000000 |
| 557 | GO:0046218 | indolalkylamine catabolic process | 2 | 2 | 1.000000 | 1.000000 |
| 558 | GO:0046416 | D-amino acid metabolic process | 2 | 2 | 1.000000 | 1.000000 |
| 559 | GO:0046488 | phosphatidylinositol metabolic process | 2 | 2 | 1.000000 | 1.000000 |
| 560 | GO:0046618 | drug export | 2 | 2 | 1.000000 | 1.000000 |
| 561 | GO:0046942 | carboxylic acid transport | 2 | 2 | 1.000000 | 1.000000 |
| 562 | GO:0048193 | Golgi vesicle transport | 2 | 2 | 1.000000 | 1.000000 |
| 563 | GO:0048278 | vesicle docking | 2 | 2 | 1.000000 | 1.000000 |
| 564 | GO:0050657 | nucleic acid transport | 2 | 2 | 1.000000 | 1.000000 |
| 565 | GO:0050658 | RNA transport | 2 | 2 | 1.000000 | 1.000000 |
| 566 | GO:0051130 | positive regulation of cellular component organization | 2 | 2 | 1.000000 | 1.000000 |
| 567 | GO:0051156 | glucose 6-phosphate metabolic process | 2 | 2 | 1.000000 | 1.000000 |
| 568 | GO:0051174 | regulation of phosphorus metabolic process | 2 | 2 | 1.000000 | 1.000000 |
| 569 | GO:0051236 | establishment of RNA localization | 2 | 2 | 1.000000 | 1.000000 |
| 570 | GO:0051321 | meiotic cell cycle | 2 | 2 | 1.000000 | 1.000000 |
| 571 | GO:0051338 | regulation of transferase activity | 2 | 2 | 1.000000 | 1.000000 |
| 572 | GO:0051493 | regulation of cytoskeleton organization | 2 | 2 | 1.000000 | 1.000000 |
| 573 | GO:0051648 | vesicle localization | 2 | 2 | 1.000000 | 1.000000 |
| 574 | GO:0051650 | establishment of vesicle localization | 2 | 2 | 1.000000 | 1.000000 |
| 575 | GO:0051726 | regulation of cell cycle | 2 | 2 | 1.000000 | 1.000000 |
| 576 | GO:0055065 | metal ion homeostasis | 2 | 2 | 1.000000 | 1.000000 |
| 577 | GO:0055067 | monovalent inorganic cation homeostasis | 2 | 2 | 1.000000 | 1.000000 |
| 578 | GO:0055070 | copper ion homeostasis | 2 | 2 | 1.000000 | 1.000000 |
| 579 | GO:0055076 | transition metal ion homeostasis | 2 | 2 | 1.000000 | 1.000000 |
| 580 | GO:0060249 | anatomical structure homeostasis | 2 | 2 | 1.000000 | 1.000000 |
| 581 | GO:0061024 | membrane organization | 2 | 2 | 1.000000 | 1.000000 |
| 582 | GO:0070646 | protein modification by small protein removal | 2 | 2 | 1.000000 | 1.000000 |
| 583 | GO:0070813 | hydrogen sulfide metabolic process | 2 | 2 | 1.000000 | 1.000000 |
| 584 | GO:0070814 | hydrogen sulfide biosynthetic process | 2 | 2 | 1.000000 | 1.000000 |
| 585 | GO:0071428 | rRNA-containing ribonucleoprotein complex export from nucleus | 2 | 2 | 1.000000 | 1.000000 |
| 586 | GO:0071824 | protein-DNA complex subunit organization | 2 | 2 | 1.000000 | 1.000000 |
| 587 | GO:0071897 | DNA biosynthetic process | 2 | 2 | 1.000000 | 1.000000 |
| 588 | GO:0072330 | monocarboxylic acid biosynthetic process | 2 | 2 | 1.000000 | 1.000000 |
| 589 | GO:0072350 | tricarboxylic acid metabolic process | 2 | 2 | 1.000000 | 1.000000 |
| 590 | GO:0072522 | purine-containing compound biosynthetic process | 2 | 2 | 1.000000 | 1.000000 |
| 591 | GO:0072527 | pyrimidine-containing compound metabolic process | 2 | 2 | 1.000000 | 1.000000 |
| 592 | GO:0072528 | pyrimidine-containing compound biosynthetic process | 2 | 2 | 1.000000 | 1.000000 |
| 593 | GO:0072665 | protein localization to vacuole | 2 | 2 | 1.000000 | 1.000000 |
| 594 | GO:0072666 | establishment of protein localization to vacuole | 2 | 2 | 1.000000 | 1.000000 |
| 595 | GO:0090066 | regulation of anatomical structure size | 2 | 2 | 1.000000 | 1.000000 |
| 596 | GO:0090522 | vesicle tethering involved in exocytosis | 2 | 2 | 1.000000 | 1.000000 |
| 597 | GO:0097033 | mitochondrial respiratory chain complex III biogenesis | 2 | 2 | 1.000000 | 1.000000 |
| 598 | GO:0098754 | detoxification | 2 | 2 | 1.000000 | 1.000000 |
| 599 | GO:1901136 | carbohydrate derivative catabolic process | 2 | 2 | 1.000000 | 1.000000 |
| 600 | GO:1901264 | carbohydrate derivative transport | 2 | 2 | 1.000000 | 1.000000 |
| 601 | GO:1901615 | organic hydroxy compound metabolic process | 2 | 2 | 1.000000 | 1.000000 |
| 602 | GO:1903046 | meiotic cell cycle process | 2 | 2 | 1.000000 | 1.000000 |
| 603 | GO:1903509 | liposaccharide metabolic process | 2 | 2 | 1.000000 | 1.000000 |
| 604 | GO:1990748 | cellular detoxification | 2 | 2 | 1.000000 | 1.000000 |
| 605 | GO:0000027 | ribosomal large subunit assembly | 1 | 1 | 1.000000 | 1.000000 |
| 606 | GO:0000032 | cell wall mannoprotein biosynthetic process | 1 | 1 | 1.000000 | 1.000000 |
| 607 | GO:0000041 | transition metal ion transport | 1 | 1 | 1.000000 | 1.000000 |
| 608 | GO:0000055 | ribosomal large subunit export from nucleus | 1 | 1 | 1.000000 | 1.000000 |
| 609 | GO:0000075 | cell cycle checkpoint | 1 | 1 | 1.000000 | 1.000000 |
| 610 | GO:0000083 | regulation of transcription involved in G1/S transition of mitotic cell cycle | 1 | 1 | 1.000000 | 1.000000 |
| 611 | GO:0000086 | G2/M transition of mitotic cell cycle | 1 | 1 | 1.000000 | 1.000000 |
| 612 | GO:0000105 | histidine biosynthetic process | 1 | 1 | 1.000000 | 1.000000 |
| 613 | GO:0000122 | negative regulation of transcription from RNA polymerase II promoter | 1 | 1 | 1.000000 | 1.000000 |
| 614 | GO:0000132 | establishment of mitotic spindle orientation | 1 | 1 | 1.000000 | 1.000000 |
| 615 | GO:0000154 | rRNA modification | 1 | 1 | 1.000000 | 1.000000 |
| 616 | GO:0000162 | tryptophan biosynthetic process | 1 | 1 | 1.000000 | 1.000000 |
| 617 | GO:0000183 | chromatin silencing at rDNA | 1 | 1 | 1.000000 | 1.000000 |
| 618 | GO:0000244 | spliceosomal tri-snRNP complex assembly | 1 | 1 | 1.000000 | 1.000000 |
| 619 | GO:0000271 | polysaccharide biosynthetic process | 1 | 1 | 1.000000 | 1.000000 |
| 620 | GO:0000288 | nuclear-transcribed mRNA catabolic process, deadenylation-dependent decay | 1 | 1 | 1.000000 | 1.000000 |
| 621 | GO:0000290 | deadenylation-dependent decapping of nuclear-transcribed mRNA | 1 | 1 | 1.000000 | 1.000000 |
| 622 | GO:0000291 | nuclear-transcribed mRNA catabolic process, exonucleolytic | 1 | 1 | 1.000000 | 1.000000 |
| 623 | GO:0000349 | generation of catalytic spliceosome for first transesterification step | 1 | 1 | 1.000000 | 1.000000 |
| 624 | GO:0000350 | generation of catalytic spliceosome for second transesterification step | 1 | 1 | 1.000000 | 1.000000 |
| 625 | GO:0000387 | spliceosomal snRNP assembly | 1 | 1 | 1.000000 | 1.000000 |
| 626 | GO:0000460 | maturation of 5.8S rRNA | 1 | 1 | 1.000000 | 1.000000 |
| 627 | GO:0000463 | maturation of LSU-rRNA from tricistronic rRNA transcript (SSU-rRNA, 5.8S rRNA, LSU-rRNA) | 1 | 1 | 1.000000 | 1.000000 |
| 628 | GO:0000466 | maturation of 5.8S rRNA from tricistronic rRNA transcript (SSU-rRNA, 5.8S rRNA, LSU-rRNA) | 1 | 1 | 1.000000 | 1.000000 |
| 629 | GO:0000470 | maturation of LSU-rRNA | 1 | 1 | 1.000000 | 1.000000 |
| 630 | GO:0000712 | resolution of meiotic recombination intermediates | 1 | 1 | 1.000000 | 1.000000 |
| 631 | GO:0000722 | telomere maintenance via recombination | 1 | 1 | 1.000000 | 1.000000 |
| 632 | GO:0000902 | cell morphogenesis | 1 | 1 | 1.000000 | 1.000000 |
| 633 | GO:0000959 | mitochondrial RNA metabolic process | 1 | 1 | 1.000000 | 1.000000 |
| 634 | GO:0001109 | promoter clearance during DNA-templated transcription | 1 | 1 | 1.000000 | 1.000000 |
| 635 | GO:0001111 | promoter clearance from RNA polymerase II promoter | 1 | 1 | 1.000000 | 1.000000 |
| 636 | GO:0001172 | transcription, RNA-templated | 1 | 1 | 1.000000 | 1.000000 |
| 637 | GO:0001173 | DNA-templated transcriptional start site selection | 1 | 1 | 1.000000 | 1.000000 |
| 638 | GO:0001174 | transcriptional start site selection at RNA polymerase II promoter | 1 | 1 | 1.000000 | 1.000000 |
| 639 | GO:0001403 | invasive growth in response to glucose limitation | 1 | 1 | 1.000000 | 1.000000 |
| 640 | GO:0001510 | RNA methylation | 1 | 1 | 1.000000 | 1.000000 |
| 641 | GO:0001558 | regulation of cell growth | 1 | 1 | 1.000000 | 1.000000 |
| 642 | GO:0002097 | tRNA wobble base modification | 1 | 1 | 1.000000 | 1.000000 |
| 643 | GO:0002098 | tRNA wobble uridine modification | 1 | 1 | 1.000000 | 1.000000 |
| 644 | GO:0002143 | tRNA wobble position uridine thiolation | 1 | 1 | 1.000000 | 1.000000 |
| 645 | GO:0002191 | cap-dependent translational initiation | 1 | 1 | 1.000000 | 1.000000 |
| 646 | GO:0002943 | tRNA dihydrouridine synthesis | 1 | 1 | 1.000000 | 1.000000 |
| 647 | GO:0005978 | glycogen biosynthetic process | 1 | 1 | 1.000000 | 1.000000 |
| 648 | GO:0005980 | glycogen catabolic process | 1 | 1 | 1.000000 | 1.000000 |
| 649 | GO:0005992 | trehalose biosynthetic process | 1 | 1 | 1.000000 | 1.000000 |
| 650 | GO:0006000 | fructose metabolic process | 1 | 1 | 1.000000 | 1.000000 |
| 651 | GO:0006003 | fructose 2,6-bisphosphate metabolic process | 1 | 1 | 1.000000 | 1.000000 |
| 652 | GO:0006006 | glucose metabolic process | 1 | 1 | 1.000000 | 1.000000 |
| 653 | GO:0006013 | mannose metabolic process | 1 | 1 | 1.000000 | 1.000000 |
| 654 | GO:0006020 | inositol metabolic process | 1 | 1 | 1.000000 | 1.000000 |
| 655 | GO:0006022 | aminoglycan metabolic process | 1 | 1 | 1.000000 | 1.000000 |
| 656 | GO:0006023 | aminoglycan biosynthetic process | 1 | 1 | 1.000000 | 1.000000 |
| 657 | GO:0006030 | chitin metabolic process | 1 | 1 | 1.000000 | 1.000000 |
| 658 | GO:0006031 | chitin biosynthetic process | 1 | 1 | 1.000000 | 1.000000 |
| 659 | GO:0006040 | amino sugar metabolic process | 1 | 1 | 1.000000 | 1.000000 |
| 660 | GO:0006056 | mannoprotein metabolic process | 1 | 1 | 1.000000 | 1.000000 |
| 661 | GO:0006057 | mannoprotein biosynthetic process | 1 | 1 | 1.000000 | 1.000000 |
| 662 | GO:0006071 | glycerol metabolic process | 1 | 1 | 1.000000 | 1.000000 |
| 663 | GO:0006072 | glycerol-3-phosphate metabolic process | 1 | 1 | 1.000000 | 1.000000 |
| 664 | GO:0006094 | gluconeogenesis | 1 | 1 | 1.000000 | 1.000000 |
| 665 | GO:0006119 | oxidative phosphorylation | 1 | 1 | 1.000000 | 1.000000 |
| 666 | GO:0006148 | inosine catabolic process | 1 | 1 | 1.000000 | 1.000000 |
| 667 | GO:0006152 | purine nucleoside catabolic process | 1 | 1 | 1.000000 | 1.000000 |
| 668 | GO:0006206 | pyrimidine nucleobase metabolic process | 1 | 1 | 1.000000 | 1.000000 |
| 669 | GO:0006207 | 'de novo' pyrimidine nucleobase biosynthetic process | 1 | 1 | 1.000000 | 1.000000 |
| 670 | GO:0006213 | pyrimidine nucleoside metabolic process | 1 | 1 | 1.000000 | 1.000000 |
| 671 | GO:0006220 | pyrimidine nucleotide metabolic process | 1 | 1 | 1.000000 | 1.000000 |
| 672 | GO:0006221 | pyrimidine nucleotide biosynthetic process | 1 | 1 | 1.000000 | 1.000000 |
| 673 | GO:0006241 | CTP biosynthetic process | 1 | 1 | 1.000000 | 1.000000 |
| 674 | GO:0006275 | regulation of DNA replication | 1 | 1 | 1.000000 | 1.000000 |
| 675 | GO:0006304 | DNA modification | 1 | 1 | 1.000000 | 1.000000 |
| 676 | GO:0006307 | DNA dealkylation involved in DNA repair | 1 | 1 | 1.000000 | 1.000000 |
| 677 | GO:0006311 | meiotic gene conversion | 1 | 1 | 1.000000 | 1.000000 |
| 678 | GO:0006312 | mitotic recombination | 1 | 1 | 1.000000 | 1.000000 |
| 679 | GO:0006359 | regulation of transcription from RNA polymerase III promoter | 1 | 1 | 1.000000 | 1.000000 |
| 680 | GO:0006360 | transcription from RNA polymerase I promoter | 1 | 1 | 1.000000 | 1.000000 |
| 681 | GO:0006406 | mRNA export from nucleus | 1 | 1 | 1.000000 | 1.000000 |
| 682 | GO:0006409 | tRNA export from nucleus | 1 | 1 | 1.000000 | 1.000000 |
| 683 | GO:0006419 | alanyl-tRNA aminoacylation | 1 | 1 | 1.000000 | 1.000000 |
| 684 | GO:0006432 | phenylalanyl-tRNA aminoacylation | 1 | 1 | 1.000000 | 1.000000 |
| 685 | GO:0006436 | tryptophanyl-tRNA aminoacylation | 1 | 1 | 1.000000 | 1.000000 |
| 686 | GO:0006448 | regulation of translational elongation | 1 | 1 | 1.000000 | 1.000000 |
| 687 | GO:0006449 | regulation of translational termination | 1 | 1 | 1.000000 | 1.000000 |
| 688 | GO:0006452 | translational frameshifting | 1 | 1 | 1.000000 | 1.000000 |
| 689 | GO:0006486 | protein glycosylation | 1 | 1 | 1.000000 | 1.000000 |
| 690 | GO:0006493 | protein O-linked glycosylation | 1 | 1 | 1.000000 | 1.000000 |
| 691 | GO:0006497 | protein lipidation | 1 | 1 | 1.000000 | 1.000000 |
| 692 | GO:0006505 | GPI anchor metabolic process | 1 | 1 | 1.000000 | 1.000000 |
| 693 | GO:0006506 | GPI anchor biosynthetic process | 1 | 1 | 1.000000 | 1.000000 |
| 694 | GO:0006515 | misfolded or incompletely synthesized protein catabolic process | 1 | 1 | 1.000000 | 1.000000 |
| 695 | GO:0006534 | cysteine metabolic process | 1 | 1 | 1.000000 | 1.000000 |
| 696 | GO:0006535 | cysteine biosynthetic process from serine | 1 | 1 | 1.000000 | 1.000000 |
| 697 | GO:0006536 | glutamate metabolic process | 1 | 1 | 1.000000 | 1.000000 |
| 698 | GO:0006537 | glutamate biosynthetic process | 1 | 1 | 1.000000 | 1.000000 |
| 699 | GO:0006544 | glycine metabolic process | 1 | 1 | 1.000000 | 1.000000 |
| 700 | GO:0006546 | glycine catabolic process | 1 | 1 | 1.000000 | 1.000000 |
| 701 | GO:0006547 | histidine metabolic process | 1 | 1 | 1.000000 | 1.000000 |
| 702 | GO:0006551 | leucine metabolic process | 1 | 1 | 1.000000 | 1.000000 |
| 703 | GO:0006560 | proline metabolic process | 1 | 1 | 1.000000 | 1.000000 |
| 704 | GO:0006561 | proline biosynthetic process | 1 | 1 | 1.000000 | 1.000000 |
| 705 | GO:0006563 | L-serine metabolic process | 1 | 1 | 1.000000 | 1.000000 |
| 706 | GO:0006575 | cellular modified amino acid metabolic process | 1 | 1 | 1.000000 | 1.000000 |
| 707 | GO:0006591 | ornithine metabolic process | 1 | 1 | 1.000000 | 1.000000 |
| 708 | GO:0006592 | ornithine biosynthetic process | 1 | 1 | 1.000000 | 1.000000 |
| 709 | GO:0006606 | protein import into nucleus | 1 | 1 | 1.000000 | 1.000000 |
| 710 | GO:0006626 | protein targeting to mitochondrion | 1 | 1 | 1.000000 | 1.000000 |
| 711 | GO:0006636 | unsaturated fatty acid biosynthetic process | 1 | 1 | 1.000000 | 1.000000 |
| 712 | GO:0006638 | neutral lipid metabolic process | 1 | 1 | 1.000000 | 1.000000 |
| 713 | GO:0006639 | acylglycerol metabolic process | 1 | 1 | 1.000000 | 1.000000 |
| 714 | GO:0006641 | triglyceride metabolic process | 1 | 1 | 1.000000 | 1.000000 |
| 715 | GO:0006642 | triglyceride mobilization | 1 | 1 | 1.000000 | 1.000000 |
| 716 | GO:0006675 | mannosyl-inositol phosphorylceramide metabolic process | 1 | 1 | 1.000000 | 1.000000 |
| 717 | GO:0006687 | glycosphingolipid metabolic process | 1 | 1 | 1.000000 | 1.000000 |
| 718 | GO:0006688 | glycosphingolipid biosynthetic process | 1 | 1 | 1.000000 | 1.000000 |
| 719 | GO:0006720 | isoprenoid metabolic process | 1 | 1 | 1.000000 | 1.000000 |
| 720 | GO:0006799 | polyphosphate biosynthetic process | 1 | 1 | 1.000000 | 1.000000 |
| 721 | GO:0006813 | potassium ion transport | 1 | 1 | 1.000000 | 1.000000 |
| 722 | GO:0006814 | sodium ion transport | 1 | 1 | 1.000000 | 1.000000 |
| 723 | GO:0006817 | phosphate ion transport | 1 | 1 | 1.000000 | 1.000000 |
| 724 | GO:0006821 | chloride transport | 1 | 1 | 1.000000 | 1.000000 |
| 725 | GO:0006828 | manganese ion transport | 1 | 1 | 1.000000 | 1.000000 |
| 726 | GO:0006842 | tricarboxylic acid transport | 1 | 1 | 1.000000 | 1.000000 |
| 727 | GO:0006843 | mitochondrial citrate transport | 1 | 1 | 1.000000 | 1.000000 |
| 728 | GO:0006855 | drug transmembrane transport | 1 | 1 | 1.000000 | 1.000000 |
| 729 | GO:0006862 | nucleotide transport | 1 | 1 | 1.000000 | 1.000000 |
| 730 | GO:0006865 | amino acid transport | 1 | 1 | 1.000000 | 1.000000 |
| 731 | GO:0006869 | lipid transport | 1 | 1 | 1.000000 | 1.000000 |
| 732 | GO:0006873 | cellular ion homeostasis | 1 | 1 | 1.000000 | 1.000000 |
| 733 | GO:0006875 | cellular metal ion homeostasis | 1 | 1 | 1.000000 | 1.000000 |
| 734 | GO:0006878 | cellular copper ion homeostasis | 1 | 1 | 1.000000 | 1.000000 |
| 735 | GO:0006879 | cellular iron ion homeostasis | 1 | 1 | 1.000000 | 1.000000 |
| 736 | GO:0006914 | autophagy | 1 | 1 | 1.000000 | 1.000000 |
| 737 | GO:0006928 | movement of cell or subcellular component | 1 | 1 | 1.000000 | 1.000000 |
| 738 | GO:0006997 | nucleus organization | 1 | 1 | 1.000000 | 1.000000 |
| 739 | GO:0006998 | nuclear envelope organization | 1 | 1 | 1.000000 | 1.000000 |
| 740 | GO:0007015 | actin filament organization | 1 | 1 | 1.000000 | 1.000000 |
| 741 | GO:0007018 | microtubule-based movement | 1 | 1 | 1.000000 | 1.000000 |
| 742 | GO:0007035 | vacuolar acidification | 1 | 1 | 1.000000 | 1.000000 |
| 743 | GO:0007051 | spindle organization | 1 | 1 | 1.000000 | 1.000000 |
| 744 | GO:0007052 | mitotic spindle organization | 1 | 1 | 1.000000 | 1.000000 |
| 745 | GO:0007093 | mitotic cell cycle checkpoint | 1 | 1 | 1.000000 | 1.000000 |
| 746 | GO:0007097 | nuclear migration | 1 | 1 | 1.000000 | 1.000000 |
| 747 | GO:0007114 | cell budding | 1 | 1 | 1.000000 | 1.000000 |
| 748 | GO:0007163 | establishment or maintenance of cell polarity | 1 | 1 | 1.000000 | 1.000000 |
| 749 | GO:0007264 | small GTPase mediated signal transduction | 1 | 1 | 1.000000 | 1.000000 |
| 750 | GO:0008299 | isoprenoid biosynthetic process | 1 | 1 | 1.000000 | 1.000000 |
| 751 | GO:0008617 | guanosine metabolic process | 1 | 1 | 1.000000 | 1.000000 |
| 752 | GO:0009070 | serine family amino acid biosynthetic process | 1 | 1 | 1.000000 | 1.000000 |
| 753 | GO:0009071 | serine family amino acid catabolic process | 1 | 1 | 1.000000 | 1.000000 |
| 754 | GO:0009098 | leucine biosynthetic process | 1 | 1 | 1.000000 | 1.000000 |
| 755 | GO:0009100 | glycoprotein metabolic process | 1 | 1 | 1.000000 | 1.000000 |
| 756 | GO:0009101 | glycoprotein biosynthetic process | 1 | 1 | 1.000000 | 1.000000 |
| 757 | GO:0009142 | nucleoside triphosphate biosynthetic process | 1 | 1 | 1.000000 | 1.000000 |
| 758 | GO:0009144 | purine nucleoside triphosphate metabolic process | 1 | 1 | 1.000000 | 1.000000 |
| 759 | GO:0009147 | pyrimidine nucleoside triphosphate metabolic process | 1 | 1 | 1.000000 | 1.000000 |
| 760 | GO:0009148 | pyrimidine nucleoside triphosphate biosynthetic process | 1 | 1 | 1.000000 | 1.000000 |
| 761 | GO:0009163 | nucleoside biosynthetic process | 1 | 1 | 1.000000 | 1.000000 |
| 762 | GO:0009164 | nucleoside catabolic process | 1 | 1 | 1.000000 | 1.000000 |
| 763 | GO:0009201 | ribonucleoside triphosphate biosynthetic process | 1 | 1 | 1.000000 | 1.000000 |
| 764 | GO:0009205 | purine ribonucleoside triphosphate metabolic process | 1 | 1 | 1.000000 | 1.000000 |
| 765 | GO:0009208 | pyrimidine ribonucleoside triphosphate metabolic process | 1 | 1 | 1.000000 | 1.000000 |
| 766 | GO:0009209 | pyrimidine ribonucleoside triphosphate biosynthetic process | 1 | 1 | 1.000000 | 1.000000 |
| 767 | GO:0009218 | pyrimidine ribonucleotide metabolic process | 1 | 1 | 1.000000 | 1.000000 |
| 768 | GO:0009220 | pyrimidine ribonucleotide biosynthetic process | 1 | 1 | 1.000000 | 1.000000 |
| 769 | GO:0009226 | nucleotide-sugar biosynthetic process | 1 | 1 | 1.000000 | 1.000000 |
| 770 | GO:0009250 | glucan biosynthetic process | 1 | 1 | 1.000000 | 1.000000 |
| 771 | GO:0009262 | deoxyribonucleotide metabolic process | 1 | 1 | 1.000000 | 1.000000 |
| 772 | GO:0009263 | deoxyribonucleotide biosynthetic process | 1 | 1 | 1.000000 | 1.000000 |
| 773 | GO:0009298 | GDP-mannose biosynthetic process | 1 | 1 | 1.000000 | 1.000000 |
| 774 | GO:0009303 | rRNA transcription | 1 | 1 | 1.000000 | 1.000000 |
| 775 | GO:0009309 | amine biosynthetic process | 1 | 1 | 1.000000 | 1.000000 |
| 776 | GO:0009312 | oligosaccharide biosynthetic process | 1 | 1 | 1.000000 | 1.000000 |
| 777 | GO:0009314 | response to radiation | 1 | 1 | 1.000000 | 1.000000 |
| 778 | GO:0009411 | response to UV | 1 | 1 | 1.000000 | 1.000000 |
| 779 | GO:0009416 | response to light stimulus | 1 | 1 | 1.000000 | 1.000000 |
| 780 | GO:0009423 | chorismate biosynthetic process | 1 | 1 | 1.000000 | 1.000000 |
| 781 | GO:0009628 | response to abiotic stimulus | 1 | 1 | 1.000000 | 1.000000 |
| 782 | GO:0009653 | anatomical structure morphogenesis | 1 | 1 | 1.000000 | 1.000000 |
| 783 | GO:0010035 | response to inorganic substance | 1 | 1 | 1.000000 | 1.000000 |
| 784 | GO:0010038 | response to metal ion | 1 | 1 | 1.000000 | 1.000000 |
| 785 | GO:0010256 | endomembrane system organization | 1 | 1 | 1.000000 | 1.000000 |
| 786 | GO:0010324 | membrane invagination | 1 | 1 | 1.000000 | 1.000000 |
| 787 | GO:0010389 | regulation of G2/M transition of mitotic cell cycle | 1 | 1 | 1.000000 | 1.000000 |
| 788 | GO:0010498 | proteasomal protein catabolic process | 1 | 1 | 1.000000 | 1.000000 |
| 789 | GO:0010525 | regulation of transposition, RNA-mediated | 1 | 1 | 1.000000 | 1.000000 |
| 790 | GO:0010528 | regulation of transposition | 1 | 1 | 1.000000 | 1.000000 |
| 791 | GO:0010562 | positive regulation of phosphorus metabolic process | 1 | 1 | 1.000000 | 1.000000 |
| 792 | GO:0010564 | regulation of cell cycle process | 1 | 1 | 1.000000 | 1.000000 |
| 793 | GO:0010570 | regulation of filamentous growth | 1 | 1 | 1.000000 | 1.000000 |
| 794 | GO:0010621 | negative regulation of transcription by transcription factor localization | 1 | 1 | 1.000000 | 1.000000 |
| 795 | GO:0010638 | positive regulation of organelle organization | 1 | 1 | 1.000000 | 1.000000 |
| 796 | GO:0010639 | negative regulation of organelle organization | 1 | 1 | 1.000000 | 1.000000 |
| 797 | GO:0010876 | lipid localization | 1 | 1 | 1.000000 | 1.000000 |
| 798 | GO:0010948 | negative regulation of cell cycle process | 1 | 1 | 1.000000 | 1.000000 |
| 799 | GO:0010952 | positive regulation of peptidase activity | 1 | 1 | 1.000000 | 1.000000 |
| 800 | GO:0010972 | negative regulation of G2/M transition of mitotic cell cycle | 1 | 1 | 1.000000 | 1.000000 |
| 801 | GO:0015693 | magnesium ion transport | 1 | 1 | 1.000000 | 1.000000 |
| 802 | GO:0015703 | chromate transport | 1 | 1 | 1.000000 | 1.000000 |
| 803 | GO:0015746 | citrate transport | 1 | 1 | 1.000000 | 1.000000 |
| 804 | GO:0015748 | organophosphate ester transport | 1 | 1 | 1.000000 | 1.000000 |
| 805 | GO:0015780 | nucleotide-sugar transport | 1 | 1 | 1.000000 | 1.000000 |
| 806 | GO:0015783 | GDP-fucose transport | 1 | 1 | 1.000000 | 1.000000 |
| 807 | GO:0015858 | nucleoside transport | 1 | 1 | 1.000000 | 1.000000 |
| 808 | GO:0015942 | formate metabolic process | 1 | 1 | 1.000000 | 1.000000 |
| 809 | GO:0015988 | energy coupled proton transmembrane transport, against electrochemical gradient | 1 | 1 | 1.000000 | 1.000000 |
| 810 | GO:0015991 | ATP hydrolysis coupled proton transport | 1 | 1 | 1.000000 | 1.000000 |
| 811 | GO:0016024 | CDP-diacylglycerol biosynthetic process | 1 | 1 | 1.000000 | 1.000000 |
| 812 | GO:0016042 | lipid catabolic process | 1 | 1 | 1.000000 | 1.000000 |
| 813 | GO:0016074 | snoRNA metabolic process | 1 | 1 | 1.000000 | 1.000000 |
| 814 | GO:0016226 | iron-sulfur cluster assembly | 1 | 1 | 1.000000 | 1.000000 |
| 815 | GO:0016237 | lysosomal microautophagy | 1 | 1 | 1.000000 | 1.000000 |
| 816 | GO:0016480 | negative regulation of transcription from RNA polymerase III promoter | 1 | 1 | 1.000000 | 1.000000 |
| 817 | GO:0016973 | poly(A)+ mRNA export from nucleus | 1 | 1 | 1.000000 | 1.000000 |
| 818 | GO:0017182 | peptidyl-diphthamide metabolic process | 1 | 1 | 1.000000 | 1.000000 |
| 819 | GO:0017183 | peptidyl-diphthamide biosynthetic process from peptidyl-histidine | 1 | 1 | 1.000000 | 1.000000 |
| 820 | GO:0018108 | peptidyl-tyrosine phosphorylation | 1 | 1 | 1.000000 | 1.000000 |
| 821 | GO:0018117 | protein adenylylation | 1 | 1 | 1.000000 | 1.000000 |
| 822 | GO:0018175 | protein nucleotidylation | 1 | 1 | 1.000000 | 1.000000 |
| 823 | GO:0018192 | enzyme active site formation via cysteine modification to L-cysteine persulfide | 1 | 1 | 1.000000 | 1.000000 |
| 824 | GO:0018198 | peptidyl-cysteine modification | 1 | 1 | 1.000000 | 1.000000 |
| 825 | GO:0018202 | peptidyl-histidine modification | 1 | 1 | 1.000000 | 1.000000 |
| 826 | GO:0018212 | peptidyl-tyrosine modification | 1 | 1 | 1.000000 | 1.000000 |
| 827 | GO:0018307 | enzyme active site formation | 1 | 1 | 1.000000 | 1.000000 |
| 828 | GO:0019310 | inositol catabolic process | 1 | 1 | 1.000000 | 1.000000 |
| 829 | GO:0019319 | hexose biosynthetic process | 1 | 1 | 1.000000 | 1.000000 |
| 830 | GO:0019344 | cysteine biosynthetic process | 1 | 1 | 1.000000 | 1.000000 |
| 831 | GO:0019357 | nicotinate nucleotide biosynthetic process | 1 | 1 | 1.000000 | 1.000000 |
| 832 | GO:0019358 | nicotinate nucleotide salvage | 1 | 1 | 1.000000 | 1.000000 |
| 833 | GO:0019365 | pyridine nucleotide salvage | 1 | 1 | 1.000000 | 1.000000 |
| 834 | GO:0019400 | alditol metabolic process | 1 | 1 | 1.000000 | 1.000000 |
| 835 | GO:0019441 | tryptophan catabolic process to kynurenine | 1 | 1 | 1.000000 | 1.000000 |
| 836 | GO:0019722 | calcium-mediated signaling | 1 | 1 | 1.000000 | 1.000000 |
| 837 | GO:0019805 | quinolinate biosynthetic process | 1 | 1 | 1.000000 | 1.000000 |
| 838 | GO:0019856 | pyrimidine nucleobase biosynthetic process | 1 | 1 | 1.000000 | 1.000000 |
| 839 | GO:0019954 | asexual reproduction | 1 | 1 | 1.000000 | 1.000000 |
| 840 | GO:0022411 | cellular component disassembly | 1 | 1 | 1.000000 | 1.000000 |
| 841 | GO:0022900 | electron transport chain | 1 | 1 | 1.000000 | 1.000000 |
| 842 | GO:0022904 | respiratory electron transport chain | 1 | 1 | 1.000000 | 1.000000 |
| 843 | GO:0030003 | cellular cation homeostasis | 1 | 1 | 1.000000 | 1.000000 |
| 844 | GO:0030004 | cellular monovalent inorganic cation homeostasis | 1 | 1 | 1.000000 | 1.000000 |
| 845 | GO:0030010 | establishment of cell polarity | 1 | 1 | 1.000000 | 1.000000 |
| 846 | GO:0030150 | protein import into mitochondrial matrix | 1 | 1 | 1.000000 | 1.000000 |
| 847 | GO:0030162 | regulation of proteolysis | 1 | 1 | 1.000000 | 1.000000 |
| 848 | GO:0030243 | cellulose metabolic process | 1 | 1 | 1.000000 | 1.000000 |
| 849 | GO:0030245 | cellulose catabolic process | 1 | 1 | 1.000000 | 1.000000 |
| 850 | GO:0030447 | filamentous growth | 1 | 1 | 1.000000 | 1.000000 |
| 851 | GO:0030491 | heteroduplex formation | 1 | 1 | 1.000000 | 1.000000 |
| 852 | GO:0030641 | regulation of cellular pH | 1 | 1 | 1.000000 | 1.000000 |
| 853 | GO:0030702 | chromatin silencing at centromere | 1 | 1 | 1.000000 | 1.000000 |
| 854 | GO:0030837 | negative regulation of actin filament polymerization | 1 | 1 | 1.000000 | 1.000000 |
| 855 | GO:0030838 | positive regulation of actin filament polymerization | 1 | 1 | 1.000000 | 1.000000 |
| 856 | GO:0031048 | chromatin silencing by small RNA | 1 | 1 | 1.000000 | 1.000000 |
| 857 | GO:0031055 | chromatin remodeling at centromere | 1 | 1 | 1.000000 | 1.000000 |
| 858 | GO:0031136 | positive regulation of conjugation | 1 | 1 | 1.000000 | 1.000000 |
| 859 | GO:0031137 | regulation of conjugation with cellular fusion | 1 | 1 | 1.000000 | 1.000000 |
| 860 | GO:0031139 | positive regulation of conjugation with cellular fusion | 1 | 1 | 1.000000 | 1.000000 |
| 861 | GO:0031146 | SCF-dependent proteasomal ubiquitin-dependent protein catabolic process | 1 | 1 | 1.000000 | 1.000000 |
| 862 | GO:0031163 | metallo-sulfur cluster assembly | 1 | 1 | 1.000000 | 1.000000 |
| 863 | GO:0031167 | rRNA methylation | 1 | 1 | 1.000000 | 1.000000 |
| 864 | GO:0031333 | negative regulation of protein complex assembly | 1 | 1 | 1.000000 | 1.000000 |
| 865 | GO:0031334 | positive regulation of protein complex assembly | 1 | 1 | 1.000000 | 1.000000 |
| 866 | GO:0031506 | cell wall glycoprotein biosynthetic process | 1 | 1 | 1.000000 | 1.000000 |
| 867 | GO:0031570 | DNA integrity checkpoint | 1 | 1 | 1.000000 | 1.000000 |
| 868 | GO:0031929 | TOR signaling | 1 | 1 | 1.000000 | 1.000000 |
| 869 | GO:0032069 | regulation of nuclease activity | 1 | 1 | 1.000000 | 1.000000 |
| 870 | GO:0032070 | regulation of deoxyribonuclease activity | 1 | 1 | 1.000000 | 1.000000 |
| 871 | GO:0032071 | regulation of endodeoxyribonuclease activity | 1 | 1 | 1.000000 | 1.000000 |
| 872 | GO:0032075 | positive regulation of nuclease activity | 1 | 1 | 1.000000 | 1.000000 |
| 873 | GO:0032077 | positive regulation of deoxyribonuclease activity | 1 | 1 | 1.000000 | 1.000000 |
| 874 | GO:0032079 | positive regulation of endodeoxyribonuclease activity | 1 | 1 | 1.000000 | 1.000000 |
| 875 | GO:0032272 | negative regulation of protein polymerization | 1 | 1 | 1.000000 | 1.000000 |
| 876 | GO:0032273 | positive regulation of protein polymerization | 1 | 1 | 1.000000 | 1.000000 |
| 877 | GO:0032447 | protein urmylation | 1 | 1 | 1.000000 | 1.000000 |
| 878 | GO:0032502 | developmental process | 1 | 1 | 1.000000 | 1.000000 |
| 879 | GO:0032505 | reproduction of a single-celled organism | 1 | 1 | 1.000000 | 1.000000 |
| 880 | GO:0032509 | endosome transport via multivesicular body sorting pathway | 1 | 1 | 1.000000 | 1.000000 |
| 881 | GO:0032511 | late endosome to vacuole transport via multivesicular body sorting pathway | 1 | 1 | 1.000000 | 1.000000 |
| 882 | GO:0032543 | mitochondrial translation | 1 | 1 | 1.000000 | 1.000000 |
| 883 | GO:0032984 | macromolecular complex disassembly | 1 | 1 | 1.000000 | 1.000000 |
| 884 | GO:0032989 | cellular component morphogenesis | 1 | 1 | 1.000000 | 1.000000 |
| 885 | GO:0033013 | tetrapyrrole metabolic process | 1 | 1 | 1.000000 | 1.000000 |
| 886 | GO:0033014 | tetrapyrrole biosynthetic process | 1 | 1 | 1.000000 | 1.000000 |
| 887 | GO:0033559 | unsaturated fatty acid metabolic process | 1 | 1 | 1.000000 | 1.000000 |
| 888 | GO:0033674 | positive regulation of kinase activity | 1 | 1 | 1.000000 | 1.000000 |
| 889 | GO:0033683 | nucleotide-excision repair, DNA incision | 1 | 1 | 1.000000 | 1.000000 |
| 890 | GO:0033692 | cellular polysaccharide biosynthetic process | 1 | 1 | 1.000000 | 1.000000 |
| 891 | GO:0034227 | tRNA thio-modification | 1 | 1 | 1.000000 | 1.000000 |
| 892 | GO:0034247 | snoRNA splicing | 1 | 1 | 1.000000 | 1.000000 |
| 893 | GO:0034314 | Arp2/3 complex-mediated actin nucleation | 1 | 1 | 1.000000 | 1.000000 |
| 894 | GO:0034354 | 'de novo' NAD biosynthetic process from tryptophan | 1 | 1 | 1.000000 | 1.000000 |
| 895 | GO:0034356 | NAD biosynthesis via nicotinamide riboside salvage pathway | 1 | 1 | 1.000000 | 1.000000 |
| 896 | GO:0034427 | nuclear-transcribed mRNA catabolic process, exonucleolytic, 3'-5' | 1 | 1 | 1.000000 | 1.000000 |
| 897 | GO:0034504 | protein localization to nucleus | 1 | 1 | 1.000000 | 1.000000 |
| 898 | GO:0034627 | 'de novo' NAD biosynthetic process | 1 | 1 | 1.000000 | 1.000000 |
| 899 | GO:0034644 | cellular response to UV | 1 | 1 | 1.000000 | 1.000000 |
| 900 | GO:0035268 | protein mannosylation | 1 | 1 | 1.000000 | 1.000000 |
| 901 | GO:0035269 | protein O-linked mannosylation | 1 | 1 | 1.000000 | 1.000000 |
| 902 | GO:0035494 | SNARE complex disassembly | 1 | 1 | 1.000000 | 1.000000 |
| 903 | GO:0035510 | DNA dealkylation | 1 | 1 | 1.000000 | 1.000000 |
| 904 | GO:0035725 | sodium ion transmembrane transport | 1 | 1 | 1.000000 | 1.000000 |
| 905 | GO:0035822 | gene conversion | 1 | 1 | 1.000000 | 1.000000 |
| 906 | GO:0036079 | purine nucleotide-sugar transport | 1 | 1 | 1.000000 | 1.000000 |
| 907 | GO:0036085 | GDP-fucose import into Golgi lumen | 1 | 1 | 1.000000 | 1.000000 |
| 908 | GO:0036092 | phosphatidylinositol-3-phosphate biosynthetic process | 1 | 1 | 1.000000 | 1.000000 |
| 909 | GO:0036267 | invasive filamentous growth | 1 | 1 | 1.000000 | 1.000000 |
| 910 | GO:0036297 | interstrand cross-link repair | 1 | 1 | 1.000000 | 1.000000 |
| 911 | GO:0040001 | establishment of mitotic spindle localization | 1 | 1 | 1.000000 | 1.000000 |
| 912 | GO:0040007 | growth | 1 | 1 | 1.000000 | 1.000000 |
| 913 | GO:0040008 | regulation of growth | 1 | 1 | 1.000000 | 1.000000 |
| 914 | GO:0040023 | establishment of nucleus localization | 1 | 1 | 1.000000 | 1.000000 |
| 915 | GO:0042026 | protein refolding | 1 | 1 | 1.000000 | 1.000000 |
| 916 | GO:0042144 | vacuole fusion, non-autophagic | 1 | 1 | 1.000000 | 1.000000 |
| 917 | GO:0042157 | lipoprotein metabolic process | 1 | 1 | 1.000000 | 1.000000 |
| 918 | GO:0042158 | lipoprotein biosynthetic process | 1 | 1 | 1.000000 | 1.000000 |
| 919 | GO:0042180 | cellular ketone metabolic process | 1 | 1 | 1.000000 | 1.000000 |
| 920 | GO:0042183 | formate catabolic process | 1 | 1 | 1.000000 | 1.000000 |
| 921 | GO:0042255 | ribosome assembly | 1 | 1 | 1.000000 | 1.000000 |
| 922 | GO:0042273 | ribosomal large subunit biogenesis | 1 | 1 | 1.000000 | 1.000000 |
| 923 | GO:0042327 | positive regulation of phosphorylation | 1 | 1 | 1.000000 | 1.000000 |
| 924 | GO:0042401 | cellular biogenic amine biosynthetic process | 1 | 1 | 1.000000 | 1.000000 |
| 925 | GO:0042435 | indole-containing compound biosynthetic process | 1 | 1 | 1.000000 | 1.000000 |
| 926 | GO:0042450 | arginine biosynthetic process via ornithine | 1 | 1 | 1.000000 | 1.000000 |
| 927 | GO:0042454 | ribonucleoside catabolic process | 1 | 1 | 1.000000 | 1.000000 |
| 928 | GO:0042455 | ribonucleoside biosynthetic process | 1 | 1 | 1.000000 | 1.000000 |
| 929 | GO:0042546 | cell wall biogenesis | 1 | 1 | 1.000000 | 1.000000 |
| 930 | GO:0042773 | ATP synthesis coupled electron transport | 1 | 1 | 1.000000 | 1.000000 |
| 931 | GO:0042790 | transcription of nuclear large rRNA transcript from RNA polymerase I promoter | 1 | 1 | 1.000000 | 1.000000 |
| 932 | GO:0043094 | cellular metabolic compound salvage | 1 | 1 | 1.000000 | 1.000000 |
| 933 | GO:0043144 | snoRNA processing | 1 | 1 | 1.000000 | 1.000000 |
| 934 | GO:0043161 | proteasome-mediated ubiquitin-dependent protein catabolic process | 1 | 1 | 1.000000 | 1.000000 |
| 935 | GO:0043162 | ubiquitin-dependent protein catabolic process via the multivesicular body sorting pathway | 1 | 1 | 1.000000 | 1.000000 |
| 936 | GO:0043173 | nucleotide salvage | 1 | 1 | 1.000000 | 1.000000 |
| 937 | GO:0043241 | protein complex disassembly | 1 | 1 | 1.000000 | 1.000000 |
| 938 | GO:0043243 | positive regulation of protein complex disassembly | 1 | 1 | 1.000000 | 1.000000 |
| 939 | GO:0043244 | regulation of protein complex disassembly | 1 | 1 | 1.000000 | 1.000000 |
| 940 | GO:0043328 | protein targeting to vacuole involved in ubiquitin-dependent protein catabolic process via the multivesicular body sorting pathway | 1 | 1 | 1.000000 | 1.000000 |
| 941 | GO:0043388 | positive regulation of DNA binding | 1 | 1 | 1.000000 | 1.000000 |
| 942 | GO:0043413 | macromolecule glycosylation | 1 | 1 | 1.000000 | 1.000000 |
| 943 | GO:0043420 | anthranilate metabolic process | 1 | 1 | 1.000000 | 1.000000 |
| 944 | GO:0043545 | molybdopterin cofactor metabolic process | 1 | 1 | 1.000000 | 1.000000 |
| 945 | GO:0043624 | cellular protein complex disassembly | 1 | 1 | 1.000000 | 1.000000 |
| 946 | GO:0043900 | regulation of multi-organism process | 1 | 1 | 1.000000 | 1.000000 |
| 947 | GO:0043902 | positive regulation of multi-organism process | 1 | 1 | 1.000000 | 1.000000 |
| 948 | GO:0044036 | cell wall macromolecule metabolic process | 1 | 1 | 1.000000 | 1.000000 |
| 949 | GO:0044038 | cell wall macromolecule biosynthetic process | 1 | 1 | 1.000000 | 1.000000 |
| 950 | GO:0044089 | positive regulation of cellular component biogenesis | 1 | 1 | 1.000000 | 1.000000 |
| 951 | GO:0044182 | filamentous growth of a population of unicellular organisms | 1 | 1 | 1.000000 | 1.000000 |
| 952 | GO:0044210 | 'de novo' CTP biosynthetic process | 1 | 1 | 1.000000 | 1.000000 |
| 953 | GO:0044743 | intracellular protein transmembrane import | 1 | 1 | 1.000000 | 1.000000 |
| 954 | GO:0044744 | protein targeting to nucleus | 1 | 1 | 1.000000 | 1.000000 |
| 955 | GO:0044767 | single-organism developmental process | 1 | 1 | 1.000000 | 1.000000 |
| 956 | GO:0044774 | mitotic DNA integrity checkpoint | 1 | 1 | 1.000000 | 1.000000 |
| 957 | GO:0044801 | single-organism membrane fusion | 1 | 1 | 1.000000 | 1.000000 |
| 958 | GO:0044839 | cell cycle G2/M phase transition | 1 | 1 | 1.000000 | 1.000000 |
| 959 | GO:0044845 | chain elongation of O-linked mannose residue | 1 | 1 | 1.000000 | 1.000000 |
| 960 | GO:0045010 | actin nucleation | 1 | 1 | 1.000000 | 1.000000 |
| 961 | GO:0045132 | meiotic chromosome segregation | 1 | 1 | 1.000000 | 1.000000 |
| 962 | GO:0045324 | late endosome to vacuole transport | 1 | 1 | 1.000000 | 1.000000 |
| 963 | GO:0045454 | cell redox homeostasis | 1 | 1 | 1.000000 | 1.000000 |
| 964 | GO:0045851 | pH reduction | 1 | 1 | 1.000000 | 1.000000 |
| 965 | GO:0045862 | positive regulation of proteolysis | 1 | 1 | 1.000000 | 1.000000 |
| 966 | GO:0045893 | positive regulation of transcription, DNA-templated | 1 | 1 | 1.000000 | 1.000000 |
| 967 | GO:0045898 | regulation of RNA polymerase II transcriptional preinitiation complex assembly | 1 | 1 | 1.000000 | 1.000000 |
| 968 | GO:0045901 | positive regulation of translational elongation | 1 | 1 | 1.000000 | 1.000000 |
| 969 | GO:0045905 | positive regulation of translational termination | 1 | 1 | 1.000000 | 1.000000 |
| 970 | GO:0045937 | positive regulation of phosphate metabolic process | 1 | 1 | 1.000000 | 1.000000 |
| 971 | GO:0045944 | positive regulation of transcription from RNA polymerase II promoter | 1 | 1 | 1.000000 | 1.000000 |
| 972 | GO:0046034 | ATP metabolic process | 1 | 1 | 1.000000 | 1.000000 |
| 973 | GO:0046036 | CTP metabolic process | 1 | 1 | 1.000000 | 1.000000 |
| 974 | GO:0046083 | adenine metabolic process | 1 | 1 | 1.000000 | 1.000000 |
| 975 | GO:0046102 | inosine metabolic process | 1 | 1 | 1.000000 | 1.000000 |
| 976 | GO:0046115 | guanosine catabolic process | 1 | 1 | 1.000000 | 1.000000 |
| 977 | GO:0046130 | purine ribonucleoside catabolic process | 1 | 1 | 1.000000 | 1.000000 |
| 978 | GO:0046131 | pyrimidine ribonucleoside metabolic process | 1 | 1 | 1.000000 | 1.000000 |
| 979 | GO:0046132 | pyrimidine ribonucleoside biosynthetic process | 1 | 1 | 1.000000 | 1.000000 |
| 980 | GO:0046134 | pyrimidine nucleoside biosynthetic process | 1 | 1 | 1.000000 | 1.000000 |
| 981 | GO:0046164 | alcohol catabolic process | 1 | 1 | 1.000000 | 1.000000 |
| 982 | GO:0046168 | glycerol-3-phosphate catabolic process | 1 | 1 | 1.000000 | 1.000000 |
| 983 | GO:0046174 | polyol catabolic process | 1 | 1 | 1.000000 | 1.000000 |
| 984 | GO:0046219 | indolalkylamine biosynthetic process | 1 | 1 | 1.000000 | 1.000000 |
| 985 | GO:0046341 | CDP-diacylglycerol metabolic process | 1 | 1 | 1.000000 | 1.000000 |
| 986 | GO:0046349 | amino sugar biosynthetic process | 1 | 1 | 1.000000 | 1.000000 |
| 987 | GO:0046351 | disaccharide biosynthetic process | 1 | 1 | 1.000000 | 1.000000 |
| 988 | GO:0046364 | monosaccharide biosynthetic process | 1 | 1 | 1.000000 | 1.000000 |
| 989 | GO:0046417 | chorismate metabolic process | 1 | 1 | 1.000000 | 1.000000 |
| 990 | GO:0046434 | organophosphate catabolic process | 1 | 1 | 1.000000 | 1.000000 |
| 991 | GO:0046497 | nicotinate nucleotide metabolic process | 1 | 1 | 1.000000 | 1.000000 |
| 992 | GO:0046834 | lipid phosphorylation | 1 | 1 | 1.000000 | 1.000000 |
| 993 | GO:0046835 | carbohydrate phosphorylation | 1 | 1 | 1.000000 | 1.000000 |
| 994 | GO:0046854 | phosphatidylinositol phosphorylation | 1 | 1 | 1.000000 | 1.000000 |
| 995 | GO:0046874 | quinolinate metabolic process | 1 | 1 | 1.000000 | 1.000000 |
| 996 | GO:0046916 | cellular transition metal ion homeostasis | 1 | 1 | 1.000000 | 1.000000 |
| 997 | GO:0046950 | cellular ketone body metabolic process | 1 | 1 | 1.000000 | 1.000000 |
| 998 | GO:0046952 | ketone body catabolic process | 1 | 1 | 1.000000 | 1.000000 |
| 999 | GO:0046999 | regulation of conjugation | 1 | 1 | 1.000000 | 1.000000 |
| 1000 | GO:0048015 | phosphatidylinositol-mediated signaling | 1 | 1 | 1.000000 | 1.000000 |
| 1001 | GO:0048016 | inositol phosphate-mediated signaling | 1 | 1 | 1.000000 | 1.000000 |
| 1002 | GO:0048017 | inositol lipid-mediated signaling | 1 | 1 | 1.000000 | 1.000000 |
| 1003 | GO:0048284 | organelle fusion | 1 | 1 | 1.000000 | 1.000000 |
| 1004 | GO:0048856 | anatomical structure development | 1 | 1 | 1.000000 | 1.000000 |
| 1005 | GO:0048869 | cellular developmental process | 1 | 1 | 1.000000 | 1.000000 |
| 1006 | GO:0050793 | regulation of developmental process | 1 | 1 | 1.000000 | 1.000000 |
| 1007 | GO:0051012 | microtubule sliding | 1 | 1 | 1.000000 | 1.000000 |
| 1008 | GO:0051028 | mRNA transport | 1 | 1 | 1.000000 | 1.000000 |
| 1009 | GO:0051031 | tRNA transport | 1 | 1 | 1.000000 | 1.000000 |
| 1010 | GO:0051054 | positive regulation of DNA metabolic process | 1 | 1 | 1.000000 | 1.000000 |
| 1011 | GO:0051098 | regulation of binding | 1 | 1 | 1.000000 | 1.000000 |
| 1012 | GO:0051099 | positive regulation of binding | 1 | 1 | 1.000000 | 1.000000 |
| 1013 | GO:0051101 | regulation of DNA binding | 1 | 1 | 1.000000 | 1.000000 |
| 1014 | GO:0051123 | RNA polymerase II transcriptional preinitiation complex assembly | 1 | 1 | 1.000000 | 1.000000 |
| 1015 | GO:0051129 | negative regulation of cellular component organization | 1 | 1 | 1.000000 | 1.000000 |
| 1016 | GO:0051131 | chaperone-mediated protein complex assembly | 1 | 1 | 1.000000 | 1.000000 |
| 1017 | GO:0051170 | nuclear import | 1 | 1 | 1.000000 | 1.000000 |
| 1018 | GO:0051189 | prosthetic group metabolic process | 1 | 1 | 1.000000 | 1.000000 |
| 1019 | GO:0051254 | positive regulation of RNA metabolic process | 1 | 1 | 1.000000 | 1.000000 |
| 1020 | GO:0051273 | beta-glucan metabolic process | 1 | 1 | 1.000000 | 1.000000 |
| 1021 | GO:0051275 | beta-glucan catabolic process | 1 | 1 | 1.000000 | 1.000000 |
| 1022 | GO:0051293 | establishment of spindle localization | 1 | 1 | 1.000000 | 1.000000 |
| 1023 | GO:0051294 | establishment of spindle orientation | 1 | 1 | 1.000000 | 1.000000 |
| 1024 | GO:0051304 | chromosome separation | 1 | 1 | 1.000000 | 1.000000 |
| 1025 | GO:0051306 | mitotic sister chromatid separation | 1 | 1 | 1.000000 | 1.000000 |
| 1026 | GO:0051307 | meiotic chromosome separation | 1 | 1 | 1.000000 | 1.000000 |
| 1027 | GO:0051347 | positive regulation of transferase activity | 1 | 1 | 1.000000 | 1.000000 |
| 1028 | GO:0051452 | intracellular pH reduction | 1 | 1 | 1.000000 | 1.000000 |
| 1029 | GO:0051453 | regulation of intracellular pH | 1 | 1 | 1.000000 | 1.000000 |
| 1030 | GO:0051494 | negative regulation of cytoskeleton organization | 1 | 1 | 1.000000 | 1.000000 |
| 1031 | GO:0051495 | positive regulation of cytoskeleton organization | 1 | 1 | 1.000000 | 1.000000 |
| 1032 | GO:0051568 | histone H3-K4 methylation | 1 | 1 | 1.000000 | 1.000000 |
| 1033 | GO:0051592 | response to calcium ion | 1 | 1 | 1.000000 | 1.000000 |
| 1034 | GO:0051604 | protein maturation | 1 | 1 | 1.000000 | 1.000000 |
| 1035 | GO:0051647 | nucleus localization | 1 | 1 | 1.000000 | 1.000000 |
| 1036 | GO:0051653 | spindle localization | 1 | 1 | 1.000000 | 1.000000 |
| 1037 | GO:0051999 | mannosyl-inositol phosphorylceramide biosynthetic process | 1 | 1 | 1.000000 | 1.000000 |
| 1038 | GO:0052547 | regulation of peptidase activity | 1 | 1 | 1.000000 | 1.000000 |
| 1039 | GO:0052646 | alditol phosphate metabolic process | 1 | 1 | 1.000000 | 1.000000 |
| 1040 | GO:0052803 | imidazole-containing compound metabolic process | 1 | 1 | 1.000000 | 1.000000 |
| 1041 | GO:0055072 | iron ion homeostasis | 1 | 1 | 1.000000 | 1.000000 |
| 1042 | GO:0055082 | cellular chemical homeostasis | 1 | 1 | 1.000000 | 1.000000 |
| 1043 | GO:0055088 | lipid homeostasis | 1 | 1 | 1.000000 | 1.000000 |
| 1044 | GO:0060260 | regulation of transcription initiation from RNA polymerase II promoter | 1 | 1 | 1.000000 | 1.000000 |
| 1045 | GO:0061025 | membrane fusion | 1 | 1 | 1.000000 | 1.000000 |
| 1046 | GO:0065002 | intracellular protein transmembrane transport | 1 | 1 | 1.000000 | 1.000000 |
| 1047 | GO:0065004 | protein-DNA complex assembly | 1 | 1 | 1.000000 | 1.000000 |
| 1048 | GO:0070127 | tRNA aminoacylation for mitochondrial protein translation | 1 | 1 | 1.000000 | 1.000000 |
| 1049 | GO:0070143 | mitochondrial alanyl-tRNA aminoacylation | 1 | 1 | 1.000000 | 1.000000 |
| 1050 | GO:0070189 | kynurenine metabolic process | 1 | 1 | 1.000000 | 1.000000 |
| 1051 | GO:0070407 | oxidation-dependent protein catabolic process | 1 | 1 | 1.000000 | 1.000000 |
| 1052 | GO:0070585 | protein localization to mitochondrion | 1 | 1 | 1.000000 | 1.000000 |
| 1053 | GO:0070589 | cellular component macromolecule biosynthetic process | 1 | 1 | 1.000000 | 1.000000 |
| 1054 | GO:0070783 | growth of unicellular organism as a thread of attached cells | 1 | 1 | 1.000000 | 1.000000 |
| 1055 | GO:0070784 | regulation of growth of unicellular organism as a thread of attached cells | 1 | 1 | 1.000000 | 1.000000 |
| 1056 | GO:0070816 | phosphorylation of RNA polymerase II C-terminal domain | 1 | 1 | 1.000000 | 1.000000 |
| 1057 | GO:0070838 | divalent metal ion transport | 1 | 1 | 1.000000 | 1.000000 |
| 1058 | GO:0070897 | DNA-templated transcriptional preinitiation complex assembly | 1 | 1 | 1.000000 | 1.000000 |
| 1059 | GO:0070914 | UV-damage excision repair | 1 | 1 | 1.000000 | 1.000000 |
| 1060 | GO:0070925 | organelle assembly | 1 | 1 | 1.000000 | 1.000000 |
| 1061 | GO:0071108 | protein K48-linked deubiquitination | 1 | 1 | 1.000000 | 1.000000 |
| 1062 | GO:0071214 | cellular response to abiotic stimulus | 1 | 1 | 1.000000 | 1.000000 |
| 1063 | GO:0071241 | cellular response to inorganic substance | 1 | 1 | 1.000000 | 1.000000 |
| 1064 | GO:0071248 | cellular response to metal ion | 1 | 1 | 1.000000 | 1.000000 |
| 1065 | GO:0071277 | cellular response to calcium ion | 1 | 1 | 1.000000 | 1.000000 |
| 1066 | GO:0071421 | manganese ion transmembrane transport | 1 | 1 | 1.000000 | 1.000000 |
| 1067 | GO:0071427 | mRNA-containing ribonucleoprotein complex export from nucleus | 1 | 1 | 1.000000 | 1.000000 |
| 1068 | GO:0071431 | tRNA-containing ribonucleoprotein complex export from nucleus | 1 | 1 | 1.000000 | 1.000000 |
| 1069 | GO:0071478 | cellular response to radiation | 1 | 1 | 1.000000 | 1.000000 |
| 1070 | GO:0071482 | cellular response to light stimulus | 1 | 1 | 1.000000 | 1.000000 |
| 1071 | GO:0071528 | tRNA re-export from nucleus | 1 | 1 | 1.000000 | 1.000000 |
| 1072 | GO:0071554 | cell wall organization or biogenesis | 1 | 1 | 1.000000 | 1.000000 |
| 1073 | GO:0071804 | cellular potassium ion transport | 1 | 1 | 1.000000 | 1.000000 |
| 1074 | GO:0071805 | potassium ion transmembrane transport | 1 | 1 | 1.000000 | 1.000000 |
| 1075 | GO:0071806 | protein transmembrane transport | 1 | 1 | 1.000000 | 1.000000 |
| 1076 | GO:0072329 | monocarboxylic acid catabolic process | 1 | 1 | 1.000000 | 1.000000 |
| 1077 | GO:0072511 | divalent inorganic cation transport | 1 | 1 | 1.000000 | 1.000000 |
| 1078 | GO:0072523 | purine-containing compound catabolic process | 1 | 1 | 1.000000 | 1.000000 |
| 1079 | GO:0072530 | purine-containing compound transmembrane transport | 1 | 1 | 1.000000 | 1.000000 |
| 1080 | GO:0072655 | establishment of protein localization to mitochondrion | 1 | 1 | 1.000000 | 1.000000 |
| 1081 | GO:0090329 | regulation of DNA-dependent DNA replication | 1 | 1 | 1.000000 | 1.000000 |
| 1082 | GO:0090342 | regulation of cell aging | 1 | 1 | 1.000000 | 1.000000 |
| 1083 | GO:0090480 | purine nucleotide-sugar transmembrane transport | 1 | 1 | 1.000000 | 1.000000 |
| 1084 | GO:0090501 | RNA phosphodiester bond hydrolysis | 1 | 1 | 1.000000 | 1.000000 |
| 1085 | GO:0090503 | RNA phosphodiester bond hydrolysis, exonucleolytic | 1 | 1 | 1.000000 | 1.000000 |
| 1086 | GO:0090662 | ATP hydrolysis coupled transmembrane transport | 1 | 1 | 1.000000 | 1.000000 |
| 1087 | GO:0097046 | replication fork progression beyond termination site | 1 | 1 | 1.000000 | 1.000000 |
| 1088 | GO:0097080 | plasma membrane selenite transport | 1 | 1 | 1.000000 | 1.000000 |
| 1089 | GO:0097428 | protein maturation by iron-sulfur cluster transfer | 1 | 1 | 1.000000 | 1.000000 |
| 1090 | GO:0097502 | mannosylation | 1 | 1 | 1.000000 | 1.000000 |
| 1091 | GO:0097576 | vacuole fusion | 1 | 1 | 1.000000 | 1.000000 |
| 1092 | GO:0098661 | inorganic anion transmembrane transport | 1 | 1 | 1.000000 | 1.000000 |
| 1093 | GO:0098781 | ncRNA transcription | 1 | 1 | 1.000000 | 1.000000 |
| 1094 | GO:0098822 | peptidyl-cysteine modification to L-cysteine persulfide | 1 | 1 | 1.000000 | 1.000000 |
| 1095 | GO:0098869 | cellular oxidant detoxification | 1 | 1 | 1.000000 | 1.000000 |
| 1096 | GO:1900062 | regulation of replicative cell aging | 1 | 1 | 1.000000 | 1.000000 |
| 1097 | GO:1900428 | regulation of filamentous growth of a population of unicellular organisms | 1 | 1 | 1.000000 | 1.000000 |
| 1098 | GO:1901068 | guanosine-containing compound metabolic process | 1 | 1 | 1.000000 | 1.000000 |
| 1099 | GO:1901069 | guanosine-containing compound catabolic process | 1 | 1 | 1.000000 | 1.000000 |
| 1100 | GO:1901071 | glucosamine-containing compound metabolic process | 1 | 1 | 1.000000 | 1.000000 |
| 1101 | GO:1901073 | glucosamine-containing compound biosynthetic process | 1 | 1 | 1.000000 | 1.000000 |
| 1102 | GO:1901255 | nucleotide-excision repair involved in interstrand cross-link repair | 1 | 1 | 1.000000 | 1.000000 |
| 1103 | GO:1901616 | organic hydroxy compound catabolic process | 1 | 1 | 1.000000 | 1.000000 |
| 1104 | GO:1901642 | nucleoside transmembrane transport | 1 | 1 | 1.000000 | 1.000000 |
| 1105 | GO:1901658 | glycosyl compound catabolic process | 1 | 1 | 1.000000 | 1.000000 |
| 1106 | GO:1901659 | glycosyl compound biosynthetic process | 1 | 1 | 1.000000 | 1.000000 |
| 1107 | GO:1901679 | nucleotide transmembrane transport | 1 | 1 | 1.000000 | 1.000000 |
| 1108 | GO:1901987 | regulation of cell cycle phase transition | 1 | 1 | 1.000000 | 1.000000 |
| 1109 | GO:1901988 | negative regulation of cell cycle phase transition | 1 | 1 | 1.000000 | 1.000000 |
| 1110 | GO:1901990 | regulation of mitotic cell cycle phase transition | 1 | 1 | 1.000000 | 1.000000 |
| 1111 | GO:1901991 | negative regulation of mitotic cell cycle phase transition | 1 | 1 | 1.000000 | 1.000000 |
| 1112 | GO:1902224 | ketone body metabolic process | 1 | 1 | 1.000000 | 1.000000 |
| 1113 | GO:1902476 | chloride transmembrane transport | 1 | 1 | 1.000000 | 1.000000 |
| 1114 | GO:1902534 | single-organism membrane invagination | 1 | 1 | 1.000000 | 1.000000 |
| 1115 | GO:1902593 | single-organism nuclear import | 1 | 1 | 1.000000 | 1.000000 |
| 1116 | GO:1902680 | positive regulation of RNA biosynthetic process | 1 | 1 | 1.000000 | 1.000000 |
| 1117 | GO:1902749 | regulation of cell cycle G2/M phase transition | 1 | 1 | 1.000000 | 1.000000 |
| 1118 | GO:1902750 | negative regulation of cell cycle G2/M phase transition | 1 | 1 | 1.000000 | 1.000000 |
| 1119 | GO:1903508 | positive regulation of nucleic acid-templated transcription | 1 | 1 | 1.000000 | 1.000000 |
| 1120 | GO:1990074 | polyuridylation-dependent mRNA catabolic process | 1 | 1 | 1.000000 | 1.000000 |
| 1121 | GO:1990542 | mitochondrial transmembrane transport | 1 | 1 | 1.000000 | 1.000000 |
| 1122 | GO:2000142 | regulation of DNA-templated transcription, initiation | 1 | 1 | 1.000000 | 1.000000 |
| 1123 | GO:2000220 | regulation of pseudohyphal growth | 1 | 1 | 1.000000 | 1.000000 |
| 1124 | GO:2000241 | regulation of reproductive process | 1 | 1 | 1.000000 | 1.000000 |
| 1125 | GO:2000243 | positive regulation of reproductive process | 1 | 1 | 1.000000 | 1.000000 |
| 1126 | GO:2000621 | regulation of DNA replication termination | 1 | 1 | 1.000000 | 1.000000 |
| 1127 | GO:2000677 | regulation of transcription regulatory region DNA binding | 1 | 1 | 1.000000 | 1.000000 |
| 1128 | GO:2000679 | positive regulation of transcription regulatory region DNA binding | 1 | 1 | 1.000000 | 1.000000 |

  

---

out GO Enrichment (Biological Process) Gene Details

| # | GO ID | geneID |
| 1 | GO:0009987 | g15713 g2139 g15457 g9478 g8736 g4970 g3250 g3247 g1559 g3987 g8896 g4832 g5803 g7932 g15421 g6194 g9820 g9793 g9745 g760 g7095 g5786 g5462 g4041 g3911 g3037 g2603 g2491 g2186 g16075 g15244 g1438 g14321 g12159 g11145 g16161 g7729 g7569 g746 g433 g323 g13276 g10737 g6087 g10811 g10143 g16047 g1635 g16101 g11042 g5719 g16122 g13214 g4910 g4289 g13019 g7749 g5849 g5674 g6254 g15911 g1768 g11142 g637 g15889 g672 g1160 g8973 g5344 g10182 g7757 g6222 g5535 g4857 g4328 g2929 g2215 g15737 g918 g2888 g1347 g504 g2509 g8536 g3374 g7564 g16055 g12208 g6012 g1110 g1598 g6095 g6962 g4782 g11117 g5806 g5755 g12258 g9743 g8930 g7711 g7134 g4311 g3128 g2610 g2229 g1571 g1467 g12412 g10901 g12037 g3857 g9081 g2764 g2983 g10152 g7203 g15356 g16006 g759 g2488 g3788 g11330 g10348 g3076 g3988 g904 g1994 g4821 g2852 g7225 g3697 g3115 g11554 g7432 g5393 g3810 g3693 g12225 g7449 g5903 g10820 g502 g2152 g15536 g15513 g7614 g4061 g8771 g7300 g5417 g16140 g6522 g6138 g10711 g9209 g577 g3457 g15455 g3660 g4773 g8625 g8245 g11984 g11950 g4580 g3445 g15829 g15282 g15549 g3822 g2080 g6334 g2737 g4623 g4211 g6125 g3337 g15714 g2268 g15134 g4762 g2451 g14381 g4365 g11098 g2682 g7307 g5633 g7633 g10256 g7873 g8648 g10823 g7594 g7282 g7453 g7518 g2556 g14291 g7249 g6281 g4153 g795 g11986 g8610 g470 g15891 g7416 g3274 g15480 g1048 g2324 g5678 g11041 g5810 g5922 g15316 g9133 g1572 g4286 g12130 g1774 g2353 g8010 g6247 g4023 g7908 g1377 g821 g6164 g16240 g15837 g15156 g10390 g9672 g9169 g9126 g8730 g7854 g7149 g6632 g6410 g602 g5980 g5694 g562 g5595 g5564 g5237 g4674 g4281 g4232 g4083 g3153 g3034 g2588 g2508 g2357 g1838 g15641 g15584 g1539 g15357 g12944 g12434 g12224 g11365 g11279 g6291 g7746 g3137 g4792 g12928 g8806 g12080 g2194 g1333 g6537 g11946 g2971 g7359 g8631 g9915 g11225 g9280 g8749 g7510 g7380 g7177 g4193 g4165 g12620 g12573 |
| 2 | GO:0008152 | g15713 g2139 g15457 g5932 g8736 g4970 g1559 g3987 g7709 g4571 g13424 g9166 g8806 g8112 g760 g6334 g6286 g2737 g1768 g1715 g15835 g15207 g14381 g12204 g11142 g11016 g8896 g4832 g5803 g7932 g15421 g6194 g9820 g9793 g9745 g7095 g5786 g5462 g4041 g3911 g3037 g2603 g2491 g2186 g16075 g15244 g1438 g14321 g12159 g11145 g16161 g7729 g7569 g746 g433 g323 g13276 g10737 g6087 g10811 g10143 g16047 g1635 g11042 g5719 g4910 g4289 g7749 g5849 g5674 g6254 g15889 g672 g1160 g16122 g8973 g5344 g10182 g918 g2888 g7757 g1347 g504 g2509 g8536 g7449 g5903 g5515 g4580 g450 g4286 g13590 g11554 g9823 g9478 g868 g867 g8550 g8245 g7931 g7873 g7388 g7249 g5833 g5810 g5806 g5634 g5346 g4989 g4665 g4637 g3857 g3274 g3178 g3076 g2857 g2807 g278 g2770 g2579 g2454 g244 g207 g1636 g16023 g15680 g15609 g15480 g15455 g15420 g1541 g15134 g15123 g14291 g11984 g11950 g11418 g10711 g10204 g1008 g3374 g7564 g16055 g6012 g1110 g1598 g6095 g4782 g11117 g5755 g12258 g9743 g8930 g7711 g7134 g4311 g3128 g2610 g2229 g1571 g1467 g12412 g10901 g12037 g9081 g15452 g2764 g2983 g10152 g7203 g15356 g16006 g759 g2488 g3788 g11330 g10348 g3988 g904 g1994 g4821 g7225 g7432 g5393 g3810 g3693 g12225 g15911 g10820 g502 g7614 g4061 g8771 g7300 g5417 g16140 g6138 g9209 g577 g3457 g8625 g15829 g4435 g5886 g11568 g15282 g15549 g3822 g4374 g2399 g1884 g1562 g13322 g4623 g4211 g6125 g16101 g10700 g12146 g15714 g2268 g4762 g2451 g2682 g7307 g5633 g7633 g2152 g15513 g10256 g3291 g12200 g3710 g821 g7264 g3498 g7594 g7453 g7518 g2556 g7282 g4365 g6281 g1577 g470 g15891 g7416 g3908 g2324 g5678 g11041 g1685 g12130 g2353 g8010 g6247 g7908 g1377 g3137 g12080 g2194 g1333 g6537 g11946 g2971 g8631 g4643 g6599 g6579 g3945 g3115 g2697 g15737 g1539 g13874 g13864 g11549 g200 g12573 |
| 3 | GO:0044699 | g2139 g15457 g5932 g15911 g6710 g3250 g3247 g1559 g3987 g8896 g5803 g6962 g10983 g1553 g6087 g10143 g16101 g5719 g16122 g13214 g4910 g4289 g7749 g5849 g6254 g1768 g11142 g637 g672 g1160 g7757 g7095 g6222 g5535 g4857 g4328 g2929 g2215 g15737 g918 g2888 g3911 g504 g2509 g8536 g7449 g5903 g5515 g4580 g4571 g450 g4286 g13590 g11554 g9823 g9820 g9478 g868 g867 g8550 g8245 g7931 g7873 g7388 g7249 g5833 g5810 g5806 g5634 g5346 g4989 g4665 g4637 g3857 g3274 g3178 g3076 g2857 g2807 g278 g2770 g2579 g2454 g244 g207 g1636 g16023 g15680 g15609 g15480 g15455 g15420 g1541 g15134 g15123 g14381 g14291 g11984 g11950 g11418 g10711 g10204 g1008 g3374 g12208 g6012 g1110 g1598 g6095 g15889 g12037 g15452 g7564 g7134 g11825 g4537 g11145 g10348 g16006 g3988 g1994 g2852 g7225 g3697 g3115 g11042 g9672 g12425 g10820 g502 g7614 g4061 g15156 g6410 g6522 g7665 g3660 g4773 g8625 g3445 g12434 g1203 g4435 g15421 g2080 g6334 g2737 g4623 g4211 g3337 g15829 g4762 g2451 g15714 g11098 g2682 g8648 g10823 g7594 g4365 g3866 g4153 g11986 g9797 g2595 g6164 g5237 g8610 g15891 g3810 g7416 g1048 g5678 g11041 g795 g8942 g9133 g1572 g2488 g1685 g12130 g1774 g821 g4023 g16240 g15837 g10390 g9169 g9126 g8730 g7854 g7149 g6632 g602 g5980 g5694 g562 g5595 g5564 g4674 g4281 g4232 g4083 g3153 g3034 g2588 g2508 g2357 g1838 g15641 g15584 g1539 g15357 g12944 g12224 g11365 g11279 g7746 g3137 g7282 g15925 g2971 g14321 g7359 g9915 g11225 |
| 4 | GO:0071704 | g15713 g2139 g15457 g5932 g8736 g4970 g1559 g3987 g7709 g4571 g13424 g9166 g8806 g8112 g760 g6334 g6286 g2737 g1768 g1715 g15835 g15207 g14381 g12204 g11142 g11016 g8896 g4832 g5803 g7932 g15421 g16161 g7729 g7569 g746 g433 g323 g13276 g10737 g6087 g10811 g10143 g1635 g11042 g5719 g4910 g4289 g7749 g5849 g5674 g6254 g15889 g672 g1160 g16122 g918 g2888 g7757 g3911 g1347 g504 g2509 g8536 g3374 g7564 g16055 g6012 g1110 g1598 g6095 g4782 g11117 g5806 g5755 g12258 g9743 g8930 g7711 g7134 g4311 g3128 g2610 g2229 g1571 g1467 g12412 g10901 g12037 g3857 g9081 g15452 g9820 g2764 g2983 g10152 g15356 g16006 g759 g2488 g3788 g11330 g11145 g10348 g3076 g3988 g6194 g904 g1994 g4821 g7225 g11554 g7432 g5393 g3810 g3693 g12225 g10182 g15911 g10820 g502 g7614 g4061 g8771 g7300 g5417 g16140 g6138 g10711 g9209 g577 g3457 g15455 g8625 g4580 g15829 g4435 g5886 g11568 g3822 g7449 g5903 g4623 g4211 g6125 g16101 g10700 g12146 g15714 g2268 g15134 g4762 g2451 g2682 g7307 g5633 g7633 g2152 g15513 g10256 g7873 g7203 g7594 g7453 g7518 g2556 g14291 g7282 g7249 g4365 g6281 g1577 g470 g15891 g7416 g3908 g3274 g15480 g2324 g5678 g11041 g5810 g1685 g4286 g12130 g2353 g8010 g6247 g7908 g1377 g821 g5344 g3137 g12080 g2194 g1333 g6537 g11946 g2971 g14321 g8631 g4643 g6599 g6579 g3945 g3115 g2697 g15737 g1539 g13874 g13864 g11549 g12573 |
| 5 | GO:0044238 | g15713 g2139 g5932 g8736 g4970 g1559 g3987 g7709 g4571 g13424 g9166 g8806 g8112 g760 g6334 g6286 g2737 g1768 g1715 g15835 g15207 g14381 g12204 g11142 g11016 g8896 g4832 g5803 g7932 g15421 g16161 g7729 g7569 g746 g433 g323 g13276 g10737 g6087 g10811 g10143 g1635 g11042 g5719 g4910 g4289 g7749 g5674 g6254 g15889 g672 g1160 g16122 g918 g2888 g7757 g3911 g1347 g504 g2509 g8536 g3374 g7564 g16055 g6012 g1110 g6095 g4782 g11117 g5806 g5755 g12258 g9743 g8930 g7711 g7134 g4311 g3128 g2610 g2229 g1571 g1467 g12412 g10901 g12037 g3857 g9081 g15452 g9820 g2764 g2983 g10152 g16006 g759 g2488 g3788 g11330 g11145 g10348 g3076 g3988 g904 g1994 g7225 g11554 g7432 g5393 g3810 g3693 g12225 g10182 g10820 g502 g7614 g4061 g8771 g7300 g5417 g16140 g6138 g10711 g9209 g577 g3457 g15455 g8625 g4580 g5886 g11568 g3822 g7449 g5903 g4821 g4623 g4211 g6125 g16101 g10700 g15829 g12146 g15714 g15134 g4762 g2451 g2682 g7307 g5633 g7633 g2152 g15513 g10256 g7873 g7594 g7453 g7518 g2556 g14291 g7282 g7249 g4365 g6281 g470 g15891 g7416 g3274 g15480 g2324 g5678 g11041 g1685 g4286 g12130 g2353 g8010 g6247 g7908 g1377 g821 g6194 g5344 g2268 g1577 g15356 g3137 g12080 g2194 g1333 g6537 g11946 g2971 g14321 g8631 g4643 g6599 g6579 g3945 g3115 g2697 g15737 g1539 g13874 g13864 g11549 g12573 |
| 6 | GO:0044237 | g15713 g2139 g15457 g8736 g4970 g1559 g3987 g8896 g4832 g5803 g7932 g15421 g6194 g9820 g9793 g9745 g760 g7095 g5786 g5462 g4041 g3911 g3037 g2603 g2491 g2186 g16075 g15244 g1438 g14321 g12159 g11145 g16161 g7729 g7569 g746 g433 g323 g13276 g10737 g6087 g10811 g10143 g1635 g11042 g5719 g4910 g4289 g7749 g5849 g5674 g6254 g1768 g11142 g15889 g672 g1160 g16122 g8973 g5344 g10182 g918 g2888 g1347 g504 g2509 g8536 g3374 g7564 g16055 g6012 g1110 g1598 g6095 g4782 g11117 g5806 g5755 g12258 g9743 g8930 g7711 g7134 g4311 g3128 g2610 g2229 g1571 g1467 g12412 g10901 g12037 g3857 g9081 g2764 g2983 g10152 g7203 g15356 g16006 g759 g2488 g3788 g11330 g10348 g3076 g3988 g904 g1994 g4821 g7225 g11554 g7432 g5393 g3810 g3693 g12225 g7449 g5903 g15911 g10820 g502 g7614 g4061 g8771 g7300 g5417 g16140 g6138 g10711 g9209 g577 g3457 g15455 g8625 g8245 g11984 g11950 g4580 g15829 g15282 g15549 g3822 g6334 g2737 g4623 g4211 g6125 g16101 g15714 g2268 g15134 g4762 g2451 g14381 g2682 g7307 g5633 g7633 g2152 g15513 g10256 g7873 g7594 g7453 g7518 g2556 g14291 g7282 g7249 g4365 g6281 g470 g15891 g7416 g3274 g15480 g2324 g5678 g11041 g5810 g4286 g12130 g2353 g8010 g6247 g7908 g1377 g821 g3137 g8806 g12080 g2194 g1333 g6537 g11946 g2971 g8631 g12573 |
| 7 | GO:0044763 | g2139 g15457 g3250 g3247 g1559 g3987 g8896 g5803 g6087 g10143 g16101 g5719 g16122 g13214 g4910 g4289 g7749 g5849 g6254 g15911 g1768 g11142 g637 g672 g1160 g7757 g7095 g6222 g5535 g4857 g4328 g2929 g2215 g15737 g918 g2888 g504 g2509 g8536 g3374 g12208 g6012 g1110 g1598 g6095 g15889 g6962 g5806 g12037 g7564 g7134 g9820 g11145 g10348 g3076 g3988 g1994 g2852 g7225 g3697 g3115 g11042 g9478 g10820 g502 g7614 g4061 g6522 g10711 g15455 g3660 g4773 g8625 g4580 g3445 g16006 g7449 g5903 g15421 g2080 g6334 g2737 g4623 g4211 g3337 g15134 g4762 g2451 g11098 g2682 g7873 g8648 g10823 g7594 g14291 g7249 g4365 g4153 g11986 g8610 g15891 g3810 g7416 g3274 g15480 g1048 g5678 g11041 g795 g5810 g9133 g1572 g2488 g4286 g12130 g1774 g821 g4023 g6164 g16240 g15837 g15156 g10390 g9672 g9169 g9126 g8730 g7854 g7149 g6632 g6410 g602 g5980 g5694 g562 g5595 g5564 g5237 g4674 g4281 g4232 g4083 g3153 g3034 g2588 g2508 g2357 g1838 g15641 g15584 g1539 g15357 g12944 g12434 g12224 g11365 g11279 g7746 g2971 g14321 g7359 g9915 g11225 |
| 8 | GO:0043170 | g15713 g8736 g4970 g4832 g5803 g7932 g15421 g16161 g7729 g7569 g746 g433 g323 g13276 g10737 g6087 g1635 g11042 g5719 g4289 g5674 g15889 g672 g1160 g16122 g1347 g8536 g3374 g7564 g16055 g6095 g4782 g11117 g5755 g12258 g9743 g8930 g7711 g7134 g4311 g3128 g2610 g2229 g1571 g1467 g12412 g10901 g12037 g3857 g9081 g2764 g2983 g10152 g15356 g16006 g759 g2488 g3788 g11330 g6194 g904 g11554 g7432 g5393 g3810 g3693 g12225 g10820 g4061 g8771 g7300 g5417 g16140 g6138 g9209 g577 g3457 g4435 g5886 g11568 g3822 g4821 g4623 g6125 g10700 g12146 g15714 g2268 g2682 g7307 g5633 g7633 g2152 g15513 g10256 g7594 g7453 g7518 g2556 g7282 g6281 g470 g15891 g3908 g2324 g11041 g2353 g8010 g6247 g7908 g1377 g821 g3137 g12080 g2194 g1333 g6254 g3076 g6537 g11946 g2971 g4643 g6599 g6579 g3945 g3115 g2697 g15737 g1539 g13874 g13864 g11549 g12573 |
| 9 | GO:0044710 | g2139 g15457 g5932 g1559 g3987 g8896 g6087 g10143 g4910 g4289 g7749 g5849 g6254 g1768 g11142 g672 g1160 g918 g2888 g7757 g3911 g504 g2509 g8536 g7449 g5903 g5515 g4580 g4571 g450 g4286 g13590 g11554 g9823 g9820 g9478 g868 g867 g8550 g8245 g7931 g7873 g7388 g7249 g5833 g5810 g5806 g5634 g5346 g4989 g4665 g4637 g3857 g3274 g3178 g3076 g2857 g2807 g278 g2770 g2579 g2454 g244 g207 g1636 g16023 g15680 g15609 g15480 g15455 g15420 g1541 g15134 g15123 g14381 g14291 g11984 g11950 g11418 g10711 g10204 g1008 g3374 g6012 g1110 g1598 g6095 g15889 g12037 g15452 g11145 g10348 g3988 g1994 g7225 g15911 g10820 g502 g7614 g4061 g8625 g4435 g15421 g6334 g2737 g4623 g4211 g16101 g15829 g4762 g2451 g2682 g7594 g4365 g15891 g3810 g7416 g5678 g11041 g1685 g12130 g821 g16122 g2971 g14321 |
| 10 | GO:0006807 | g15713 g2139 g8736 g4970 g1559 g3987 g8896 g4832 g5803 g7932 g15421 g16161 g7729 g7569 g746 g433 g323 g13276 g10737 g10811 g10143 g1635 g6087 g5719 g4289 g7749 g672 g16122 g918 g2888 g504 g2509 g3374 g7564 g16055 g6012 g1110 g6095 g15889 g4782 g11117 g5806 g3857 g9081 g9820 g15356 g16006 g759 g2488 g3788 g11145 g10348 g3076 g3988 g904 g1994 g7225 g11554 g7432 g5393 g3810 g3693 g12225 g10182 g10820 g502 g4061 g8771 g7300 g5417 g2983 g16140 g6138 g8625 g4580 g4435 g3822 g7449 g5903 g4821 g6334 g2737 g4211 g6125 g15714 g15134 g4762 g2451 g2682 g7633 g7873 g7453 g7518 g2556 g7282 g7249 g6281 g15891 g7416 g3274 g15480 g2324 g11041 g4286 g7908 g1377 g5344 g8806 g2194 g1333 g6254 g6537 g11946 g8631 g6194 g200 g2268 g12573 |
| 11 | GO:0044260 | g15713 g8736 g4970 g4832 g16161 g7729 g7569 g746 g433 g323 g13276 g10737 g6087 g1635 g11042 g5719 g4289 g5674 g15889 g672 g1160 g16122 g1347 g8536 g3374 g7564 g16055 g6095 g4782 g11117 g5803 g5755 g12258 g9743 g8930 g7711 g7134 g4311 g3128 g2610 g2229 g1571 g1467 g12412 g10901 g12037 g3857 g9081 g2764 g2983 g10152 g15356 g16006 g759 g2488 g3788 g11330 g904 g11554 g7432 g5393 g3810 g3693 g12225 g10820 g4061 g8771 g7300 g5417 g16140 g6138 g9209 g577 g3457 g3822 g4821 g15421 g4623 g6125 g15714 g2268 g2682 g7307 g5633 g7633 g2152 g15513 g10256 g7594 g7453 g7518 g2556 g7282 g6281 g470 g15891 g2324 g11041 g2353 g8010 g6247 g7908 g1377 g821 g3137 g12080 g2194 g1333 g6254 g3076 g2971 g12573 |
| 12 | GO:0034641 | g15713 g2139 g8736 g4970 g8896 g4832 g3987 g5803 g7932 g15421 g16161 g7729 g7569 g746 g433 g323 g13276 g10737 g10811 g1635 g6087 g5719 g4289 g7749 g672 g16122 g10143 g504 g2509 g3374 g7564 g16055 g6012 g1110 g6095 g15889 g4782 g11117 g3857 g9081 g15356 g16006 g759 g2488 g3788 g11145 g10348 g904 g11554 g7432 g5393 g3810 g3693 g12225 g10182 g10820 g502 g4061 g8771 g7300 g5417 g2983 g16140 g6138 g3822 g6334 g2737 g6125 g15714 g15134 g2682 g7633 g7873 g7453 g7518 g2556 g7282 g6281 g15891 g2324 g11041 g4286 g7908 g1377 g8806 g2194 g1333 g6254 g3076 g6537 g11946 g8631 g4762 g12573 |
| 13 | GO:0051179 | g15911 g6710 g3250 g3247 g9823 g11240 g6962 g10983 g1553 g12208 g7665 g6814 g11825 g4537 g15457 g5997 g9672 g12425 g3805 g7564 g15156 g6410 g6522 g3660 g4773 g12434 g1203 g5719 g15306 g15714 g11098 g3866 g8930 g4153 g11986 g9797 g2595 g6164 g5237 g8610 g2737 g13911 g13320 g10417 g795 g8942 g15889 g5681 g918 g16240 g15837 g10390 g9169 g9126 g8730 g7854 g7149 g6632 g602 g5980 g5694 g562 g5595 g5564 g4674 g4281 g4232 g4083 g3153 g3034 g2588 g2508 g2357 g1838 g15641 g15584 g1539 g15357 g12944 g12224 g11365 g11279 g7746 g3137 g7282 g15925 g7359 g9915 g637 g316 g2477 |
| 14 | GO:0051234 | g15911 g6710 g3250 g3247 g9823 g11240 g6962 g10983 g1553 g12208 g7665 g6814 g11825 g4537 g15457 g5997 g9672 g12425 g7564 g15156 g6410 g6522 g3660 g4773 g12434 g1203 g5719 g15306 g15714 g11098 g3866 g4153 g11986 g9797 g2595 g6164 g5237 g8610 g2737 g13911 g13320 g10417 g795 g8942 g15889 g5681 g918 g16240 g15837 g10390 g9169 g9126 g8730 g7854 g7149 g6632 g602 g5980 g5694 g562 g5595 g5564 g4674 g4281 g4232 g4083 g3153 g3034 g2588 g2508 g2357 g1838 g15641 g15584 g1539 g15357 g12944 g12224 g11365 g11279 g7746 g3137 g7282 g15925 g7359 g9915 g637 g316 g2477 |
| 15 | GO:0006810 | g15911 g6710 g3250 g3247 g9823 g11240 g6962 g10983 g1553 g12208 g7665 g6814 g11825 g4537 g15457 g5997 g9672 g12425 g15156 g6410 g6522 g3660 g4773 g12434 g1203 g15306 g15714 g11098 g3866 g4153 g11986 g9797 g2595 g6164 g5237 g8610 g2737 g13911 g13320 g10417 g795 g8942 g15889 g5681 g918 g16240 g15837 g10390 g9169 g9126 g8730 g7854 g7149 g6632 g602 g5980 g5694 g562 g5595 g5564 g4674 g4281 g4232 g4083 g3153 g3034 g2588 g2508 g2357 g1838 g15641 g15584 g1539 g15357 g12944 g12224 g11365 g11279 g7746 g3137 g7282 g15925 g7359 g9915 g637 g316 g2477 |
| 16 | GO:0006725 | g15713 g2139 g8736 g4970 g8896 g3987 g5803 g7932 g15421 g16161 g7729 g7569 g746 g433 g323 g13276 g10737 g10811 g10143 g1635 g6087 g5719 g4289 g7749 g672 g16122 g504 g2509 g3374 g7564 g16055 g6012 g1110 g6095 g15889 g4782 g11117 g3857 g9081 g9820 g16006 g759 g2488 g3788 g11145 g10348 g1994 g11554 g7432 g5393 g3810 g3693 g10182 g10820 g502 g4061 g6138 g8245 g11984 g11950 g3822 g6334 g2737 g6125 g15714 g15134 g2682 g7873 g7453 g7282 g6281 g15891 g2324 g11041 g4286 g7908 g1377 g8806 g2194 g1333 g6254 g3076 g6537 g11946 g8631 g4762 |
| 17 | GO:1901360 | g15713 g2139 g8736 g4970 g8896 g3987 g5803 g7932 g15421 g16161 g7729 g7569 g746 g433 g323 g13276 g10737 g10811 g10143 g1635 g6087 g5719 g4289 g7749 g672 g16122 g504 g2509 g3374 g7564 g16055 g6012 g1110 g6095 g15889 g4782 g11117 g3857 g9081 g9820 g16006 g759 g2488 g3788 g11145 g10348 g1994 g11554 g7432 g5393 g3810 g3693 g10182 g10820 g502 g4061 g6138 g3822 g4821 g6334 g2737 g6125 g15714 g15134 g2682 g7873 g7453 g7282 g7249 g6281 g15891 g2324 g11041 g4286 g7908 g1377 g8806 g2194 g1333 g6254 g3076 g6537 g11946 g8631 g4762 |
| 18 | GO:0046483 | g15713 g2139 g8736 g4970 g8896 g3987 g5803 g7932 g15421 g16161 g7729 g7569 g746 g433 g323 g13276 g10737 g10811 g1635 g6087 g5719 g4289 g7749 g672 g16122 g10143 g504 g2509 g3374 g7564 g16055 g6012 g1110 g6095 g15889 g4782 g11117 g3857 g9081 g16006 g759 g2488 g3788 g11145 g10348 g4821 g11554 g7432 g5393 g3810 g3693 g10182 g10820 g502 g4061 g6138 g3822 g6334 g2737 g6125 g15714 g15134 g2682 g7873 g7453 g7282 g7249 g6281 g15891 g2324 g11041 g4286 g7908 g1377 g8806 g2194 g1333 g6254 g3076 g6537 g11946 g8631 g4762 |
| 19 | GO:0009058 | g2139 g15457 g5932 g1559 g4832 g3987 g16047 g1635 g6087 g5719 g7749 g1160 g504 g2509 g7564 g16055 g6012 g6095 g9081 g9820 g7203 g15356 g759 g2488 g3788 g3076 g3988 g904 g4821 g16122 g7225 g11554 g7432 g5393 g3810 g3693 g12225 g7449 g5903 g10182 g502 g15889 g7614 g4061 g8771 g7300 g5417 g2983 g16140 g10711 g15455 g8625 g4580 g4435 g8896 g4623 g4211 g16101 g7633 g918 g3291 g2888 g2451 g12200 g11145 g7518 g2556 g7249 g10143 g7416 g5678 g11041 g4286 g12130 g821 g8806 g2971 g4762 g12573 |
| 20 | GO:0006139 | g15713 g2139 g8736 g4970 g8896 g5803 g7932 g15421 g16161 g7729 g7569 g746 g433 g323 g13276 g10737 g10811 g1635 g6087 g5719 g4289 g7749 g672 g16122 g504 g2509 g3374 g7564 g16055 g6012 g1110 g6095 g15889 g4782 g11117 g3857 g9081 g16006 g759 g2488 g3788 g11145 g10348 g11554 g7432 g5393 g3810 g3693 g10182 g10820 g4061 g6138 g3822 g6334 g2737 g6125 g15714 g2682 g7873 g7453 g7282 g6281 g15891 g10143 g2324 g11041 g7908 g1377 g8806 g2194 g1333 g6254 g3076 g6537 g11946 g8631 g4762 |
| 21 | GO:0019538 | g4832 g6087 g11042 g5674 g15889 g1160 g1347 g8536 g6095 g5803 g5755 g12258 g9743 g8930 g7711 g7134 g4311 g3128 g2610 g2229 g1571 g1467 g12412 g10901 g12037 g2764 g2983 g10152 g904 g12225 g4061 g8771 g7300 g5417 g16140 g9209 g577 g3457 g16006 g4821 g10700 g12146 g7307 g5633 g7633 g2152 g15513 g10256 g9081 g759 g7518 g3788 g2556 g470 g16122 g11041 g2353 g8010 g6247 g821 g3137 g12080 g2971 g4643 g6599 g6579 g3945 g3115 g2697 g15737 g1539 g13874 g13864 g11549 g12573 |
| 22 | GO:1902578 | g15911 g6710 g3250 g3247 g6962 g10983 g1553 g12208 g11825 g4537 g15457 g9672 g12425 g7564 g15156 g6410 g6522 g7665 g3660 g12434 g1203 g15714 g11098 g3866 g4153 g11986 g9797 g2595 g6164 g5237 g8610 g795 g8942 g15889 g16240 g15837 g10390 g9169 g9126 g8730 g7854 g7149 g6632 g602 g5980 g5694 g562 g5595 g5564 g4674 g4281 g4232 g4083 g3153 g3034 g2588 g2508 g2357 g1838 g15641 g15584 g1539 g15357 g12944 g12224 g11365 g11279 g7746 g3137 g7282 g15925 g7359 g9915 |
| 23 | GO:0044765 | g15911 g6710 g3250 g3247 g6962 g10983 g1553 g12208 g11825 g4537 g15457 g9672 g12425 g15156 g6410 g6522 g7665 g3660 g12434 g1203 g15714 g11098 g3866 g4153 g11986 g9797 g2595 g6164 g5237 g8610 g795 g8942 g15889 g16240 g15837 g10390 g9169 g9126 g8730 g7854 g7149 g6632 g602 g5980 g5694 g562 g5595 g5564 g4674 g4281 g4232 g4083 g3153 g3034 g2588 g2508 g2357 g1838 g15641 g15584 g1539 g15357 g12944 g12224 g11365 g11279 g7746 g3137 g7282 g15925 g7359 g9915 |
| 24 | GO:1901576 | g2139 g15457 g5932 g1559 g4832 g3987 g1635 g6087 g5719 g7749 g1160 g504 g2509 g7564 g16055 g6012 g6095 g9081 g9820 g15356 g759 g2488 g3788 g3076 g3988 g904 g4821 g16122 g7225 g11554 g7432 g5393 g3810 g3693 g12225 g10182 g502 g15889 g7614 g4061 g8771 g7300 g5417 g2983 g16140 g10711 g15455 g8625 g4580 g4435 g7449 g5903 g8896 g4623 g4211 g16101 g7633 g7203 g11145 g7518 g2556 g7249 g10143 g7416 g5678 g11041 g4286 g12130 g8806 g2971 g4762 g12573 |
| 25 | GO:0044249 | g2139 g15457 g1559 g4832 g3987 g1635 g6087 g5719 g7749 g1160 g504 g2509 g7564 g16055 g6012 g6095 g9081 g9820 g7203 g15356 g759 g2488 g3788 g3076 g3988 g904 g4821 g16122 g7225 g11554 g7432 g5393 g3810 g3693 g12225 g7449 g5903 g10182 g502 g15889 g7614 g4061 g8771 g7300 g5417 g2983 g16140 g10711 g15455 g8625 g4580 g8896 g4623 g4211 g16101 g7633 g11145 g7518 g2556 g7249 g10143 g7416 g5678 g11041 g4286 g12130 g821 g8806 g2971 g4762 g12573 |
| 26 | GO:0065007 | g15889 g8930 g5803 g5719 g16122 g11986 g1635 g16101 g14315 g727 g7038 g4127 g637 g7757 g7095 g6222 g5535 g4857 g4328 g2929 g2215 g15737 g1057 g15457 g7134 g3445 g2852 g2663 g5742 g5191 g1809 g1491 g9478 g672 g9797 g11221 g10820 g7711 g475 g12208 g7590 g4057 g7873 g5932 g221 g16063 g16 g2080 g12225 g2399 g8648 g10823 g3788 g4833 g9010 g8545 g5312 g4421 g4319 g3128 g2219 g16006 g795 g1572 g2488 g1774 g3697 g15911 |
| 27 | GO:0006793 | g2139 g15457 g6194 g9820 g9793 g9745 g760 g7095 g5786 g5462 g4041 g3911 g3037 g2603 g2491 g2186 g16075 g15244 g1438 g14321 g12159 g11145 g7749 g15889 g8973 g5344 g10182 g6012 g1110 g5803 g5755 g12258 g9743 g8930 g7711 g7134 g4311 g3128 g2610 g2229 g1571 g1467 g12412 g10901 g2764 g2983 g10152 g4821 g15911 g15829 g15282 g15549 g8896 g6334 g2737 g4211 g16101 g14381 g504 g2509 g470 g16122 g7416 g10143 g5678 g12080 g2971 |
| 28 | GO:0055114 | g4910 g4289 g1559 g7449 g5903 g5515 g4580 g4571 g450 g4286 g13590 g11554 g9823 g9820 g9478 g868 g867 g8550 g8245 g7931 g7873 g7388 g7249 g5849 g5833 g5810 g5806 g5634 g5346 g4989 g4665 g4637 g3857 g3274 g3178 g3076 g2857 g2807 g278 g2770 g2579 g2454 g244 g2139 g207 g1636 g16023 g15680 g15609 g15480 g15455 g15420 g1541 g15134 g15123 g14381 g14291 g11984 g11950 g11418 g10711 g10204 g10143 g1008 g1110 g4623 g7594 |
| 29 | GO:0006796 | g2139 g6194 g9820 g9793 g9745 g760 g7095 g5786 g5462 g4041 g3911 g3037 g2603 g2491 g2186 g16075 g15457 g15244 g1438 g14321 g12159 g11145 g7749 g15889 g8973 g5344 g10182 g6012 g1110 g5803 g5755 g12258 g9743 g8930 g7711 g7134 g4311 g3128 g2610 g2229 g1571 g1467 g12412 g10901 g2764 g2983 g10152 g15829 g15282 g15549 g4821 g8896 g6334 g2737 g4211 g16101 g14381 g504 g2509 g470 g16122 g7416 g10143 g5678 g12080 g2971 |
| 30 | GO:0050789 | g15889 g8930 g5803 g5719 g16122 g1635 g16101 g14315 g727 g7038 g4127 g637 g7757 g7095 g6222 g5535 g4857 g4328 g2929 g2215 g15737 g1057 g15457 g7134 g2852 g2663 g5742 g5191 g1809 g1491 g9478 g672 g9797 g11221 g7711 g475 g7590 g4057 g7873 g5932 g221 g16063 g16 g2080 g12225 g8648 g10823 g4833 g9010 g8545 g5312 g4421 g4319 g3788 g3128 g2219 g11986 g16006 g795 g1572 g2488 g1774 g3697 g15911 |
| 31 | GO:0044267 | g4832 g6087 g11042 g5674 g15889 g1160 g1347 g8536 g6095 g5803 g5755 g12258 g9743 g8930 g7711 g7134 g4311 g3128 g2610 g2229 g1571 g1467 g12412 g10901 g12037 g2764 g2983 g10152 g904 g12225 g4061 g8771 g7300 g5417 g16140 g9209 g577 g3457 g16006 g4821 g7307 g5633 g7633 g2152 g15513 g10256 g9081 g759 g7518 g3788 g2556 g470 g16122 g11041 g2353 g8010 g6247 g821 g3137 g12080 g2971 g12573 |
| 32 | GO:0050794 | g15889 g8930 g5719 g16122 g1635 g16101 g14315 g727 g7038 g4127 g637 g7757 g7095 g6222 g5535 g4857 g4328 g2929 g2215 g15737 g1057 g15457 g7134 g2852 g2663 g5742 g5191 g1809 g1491 g9478 g672 g475 g7590 g4057 g7873 g5932 g221 g16063 g16 g7711 g2080 g12225 g8648 g10823 g4833 g9010 g8545 g5312 g4421 g4319 g3788 g3128 g2219 g11986 g16006 g1572 g2488 g1774 g3697 g15911 |
| 33 | GO:0090304 | g15713 g8736 g4970 g5803 g7932 g15421 g16161 g7729 g7569 g746 g433 g323 g13276 g10737 g1635 g6087 g5719 g4289 g672 g16122 g3374 g7564 g16055 g6095 g15889 g4782 g11117 g3857 g9081 g16006 g759 g2488 g3788 g11554 g7432 g5393 g3810 g3693 g10820 g4061 g6138 g3822 g6125 g15714 g2682 g7453 g7282 g6281 g15891 g2324 g11041 g7908 g1377 g2194 g1333 g6254 g3076 g6537 g11946 |
| 34 | GO:1901564 | g1559 g3987 g8896 g4832 g10143 g7749 g918 g2888 g504 g2509 g6012 g1110 g6095 g5806 g9820 g11145 g10348 g3076 g3988 g904 g1994 g7225 g12225 g502 g4061 g8771 g7300 g5417 g2983 g16140 g8625 g4580 g4435 g7449 g5903 g4821 g6334 g2737 g4211 g15134 g4762 g2451 g7633 g7873 g9081 g759 g7518 g3788 g2556 g7249 g7416 g3274 g15480 g11041 g4286 g5344 g12573 |
| 35 | GO:0044281 | g2139 g15457 g5932 g1559 g3987 g8896 g10143 g4910 g4289 g7749 g5849 g918 g2888 g504 g2509 g6012 g1110 g1598 g6095 g5806 g9820 g11145 g10348 g3076 g3988 g1994 g7225 g15911 g502 g4061 g10711 g15455 g8625 g4580 g7449 g5903 g6334 g2737 g15829 g15134 g4762 g2451 g7873 g14291 g7249 g3274 g15480 g11041 g1685 g4286 g14321 |
| 36 | GO:0055085 | g15911 g12208 g6962 g6522 g3660 g11098 g4153 g11986 g8610 g795 g6164 g16240 g15837 g15156 g10390 g9672 g9169 g9126 g8730 g7854 g7149 g6632 g6410 g602 g5980 g5694 g562 g5595 g5564 g5237 g4674 g4281 g4232 g4083 g3153 g3034 g2588 g2508 g2357 g1838 g15641 g15584 g1539 g15357 g12944 g12434 g12224 g11365 g11279 g7359 g9915 |
| 37 | GO:0071840 | g15457 g6087 g16047 g5719 g16122 g13214 g637 g672 g8536 g6095 g12037 g6376 g6593 g10820 g7564 g3788 g15889 g6138 g4773 g3445 g10811 g3822 g6125 g3337 g7282 g6281 g1160 g1048 g1635 g2324 g795 g5922 g15316 g9133 g1572 g2488 g4023 g6291 g13276 g13019 g4792 g12928 g12208 g15925 g11225 g12573 g4832 g5674 |
| 38 | GO:0043412 | g15713 g6087 g15889 g1160 g1347 g8536 g3374 g5803 g5755 g12258 g9743 g8930 g7711 g7134 g4311 g3128 g2610 g2229 g1571 g1467 g12412 g10901 g12037 g3857 g2764 g2983 g10152 g16006 g7307 g5633 g3457 g11042 g2152 g15513 g10256 g6281 g470 g16122 g2353 g8010 g6247 g821 g12080 g2971 g9209 |
| 39 | GO:0010467 | g8736 g4970 g4832 g1635 g6087 g5719 g7564 g16055 g6095 g4782 g11117 g3857 g9081 g16006 g759 g2488 g3788 g904 g12225 g15889 g4061 g8771 g7300 g5417 g2983 g16140 g6138 g3822 g6125 g10700 g15714 g7633 g7518 g2556 g7282 g6281 g3908 g15356 g2324 g11041 g6254 g3076 g12573 |
| 40 | GO:1901566 | g1559 g4832 g3987 g7749 g504 g2509 g6012 g6095 g9820 g3076 g3988 g904 g7225 g12225 g502 g4061 g8771 g7300 g5417 g2983 g16140 g8625 g4580 g4435 g7449 g5903 g8896 g4211 g7633 g9081 g759 g7518 g3788 g2556 g7249 g4821 g10143 g7416 g11041 g4286 g4762 g12573 |
| 41 | GO:0006464 | g6087 g15889 g1160 g1347 g8536 g5803 g5755 g12258 g9743 g8930 g7711 g7134 g4311 g3128 g2610 g2229 g1571 g1467 g12412 g10901 g12037 g2764 g2983 g10152 g16006 g7307 g5633 g3457 g11042 g2152 g15513 g10256 g470 g16122 g2353 g8010 g6247 g821 g12080 g2971 g9209 |
| 42 | GO:0016043 | g15457 g6087 g16047 g5719 g16122 g13214 g637 g672 g8536 g6095 g12037 g10820 g7564 g3788 g15889 g6138 g4773 g3445 g10811 g3822 g6125 g3337 g7282 g1048 g795 g5922 g15316 g9133 g1572 g2488 g4023 g6291 g13276 g13019 g4792 g12928 g12208 g11225 g12573 g4832 g5674 |
| 43 | GO:0016310 | g6194 g9820 g9793 g9745 g760 g7095 g5786 g5462 g4041 g3911 g3037 g2603 g2491 g2186 g16075 g15457 g15244 g1438 g14321 g12159 g11145 g15889 g1110 g5803 g5755 g12258 g9743 g8930 g7711 g7134 g4311 g3128 g2610 g2229 g1571 g1467 g12412 g10901 g15829 g16101 g12080 |
| 44 | GO:0036211 | g6087 g15889 g1160 g1347 g8536 g5803 g5755 g12258 g9743 g8930 g7711 g7134 g4311 g3128 g2610 g2229 g1571 g1467 g12412 g10901 g12037 g2764 g2983 g10152 g16006 g7307 g5633 g3457 g11042 g2152 g15513 g10256 g470 g16122 g2353 g8010 g6247 g821 g12080 g2971 g9209 |
| 45 | GO:0044271 | g2139 g4832 g3987 g1635 g6087 g5719 g7749 g504 g2509 g7564 g16055 g6012 g6095 g9081 g15356 g759 g2488 g3788 g904 g12225 g10182 g502 g15889 g4061 g8771 g7300 g5417 g2983 g16140 g7432 g3810 g8896 g7633 g11145 g7518 g2556 g10143 g11041 g8806 g4762 g12573 |
| 46 | GO:0019222 | g5803 g5719 g16122 g15889 g1635 g14315 g1057 g8930 g2663 g672 g9797 g11221 g7711 g475 g7590 g4057 g7873 g5932 g221 g16063 g16 g12225 g4857 g4328 g2929 g15737 g4833 g9010 g8545 g5312 g4421 g4319 g3788 g3128 g2219 g11986 g795 g2488 g3697 |
| 47 | GO:0009059 | g4832 g1635 g6087 g5719 g1160 g7564 g16055 g6095 g9081 g15356 g759 g2488 g3788 g904 g16122 g11554 g7432 g5393 g3810 g3693 g12225 g15889 g4061 g8771 g7300 g5417 g2983 g16140 g4435 g4623 g7633 g7518 g2556 g11041 g2971 g12573 |
| 48 | GO:0044711 | g2139 g15457 g5932 g1559 g7749 g504 g2509 g6012 g6095 g9820 g3076 g3988 g7225 g502 g7614 g10711 g15455 g8625 g4580 g4435 g7449 g5903 g8896 g4623 g4211 g16101 g11145 g7249 g10143 g7416 g5678 g4286 g12130 g821 g2971 g4762 |
| 49 | GO:0034645 | g4832 g1635 g6087 g5719 g1160 g7564 g16055 g6095 g9081 g15356 g759 g2488 g3788 g904 g16122 g11554 g7432 g5393 g3810 g3693 g12225 g15889 g4061 g8771 g7300 g5417 g2983 g16140 g4623 g7633 g7518 g2556 g11041 g2971 g12573 |
| 50 | GO:0050896 | g9478 g16101 g13276 g13019 g7757 g7095 g6222 g5535 g4857 g4328 g2929 g2215 g15737 g3374 g15457 g15889 g7134 g2852 g15591 g15156 g6410 g5674 g16006 g15421 g2080 g4365 g11098 g2682 g8648 g10823 g15891 g3810 g10820 g1774 g8930 |
| 51 | GO:0006082 | g15457 g1559 g3987 g10143 g4910 g4289 g5849 g918 g2888 g6095 g5806 g9820 g3076 g3988 g1994 g7225 g15911 g502 g4061 g10711 g15455 g8625 g4580 g7449 g5903 g15134 g6012 g4762 g2451 g7249 g3274 g15480 g11041 g4286 |
| 52 | GO:0043436 | g15457 g1559 g3987 g10143 g4910 g4289 g5849 g918 g2888 g6095 g5806 g9820 g3076 g3988 g1994 g7225 g15911 g502 g4061 g10711 g15455 g8625 g4580 g7449 g5903 g15134 g6012 g4762 g2451 g7249 g3274 g15480 g11041 g4286 |
| 53 | GO:0016070 | g15713 g8736 g4970 g1635 g6087 g5719 g4289 g7564 g16055 g6095 g4782 g11117 g3857 g9081 g16006 g759 g2488 g3788 g15889 g4061 g6138 g3822 g6125 g15714 g7453 g7282 g6281 g2324 g11041 g2194 g1333 g6254 g3076 |
| 54 | GO:0005975 | g5932 g7709 g4571 g13424 g9166 g8806 g8112 g760 g6334 g6286 g2737 g1768 g1715 g15835 g15207 g14381 g12204 g11142 g11016 g6254 g1160 g15452 g11330 g5886 g11568 g4623 g15829 g7594 g14291 g1685 g12130 g14321 |
| 55 | GO:0019752 | g1559 g3987 g10143 g4910 g4289 g5849 g918 g2888 g6095 g5806 g9820 g3076 g3988 g1994 g7225 g502 g4061 g10711 g15455 g8625 g4580 g7449 g5903 g15134 g6012 g4762 g2451 g7249 g3274 g15480 g11041 g4286 |
| 56 | GO:0060255 | g5803 g5719 g16122 g15889 g1635 g14315 g1057 g8930 g2663 g672 g7711 g475 g7590 g4057 g7873 g5932 g221 g16063 g16 g12225 g4833 g9010 g8545 g5312 g4421 g4319 g3788 g3128 g2219 g11986 g2488 g3697 |
| 57 | GO:0031323 | g5719 g16122 g15889 g1635 g14315 g1057 g8930 g2663 g672 g475 g7590 g4057 g7873 g5932 g221 g16063 g16 g7711 g12225 g4833 g9010 g8545 g5312 g4421 g4319 g3788 g3128 g2219 g11986 g2488 g3697 |
| 58 | GO:0080090 | g5719 g16122 g15889 g1635 g14315 g1057 g8930 g2663 g672 g475 g7590 g4057 g7873 g5932 g221 g16063 g16 g7711 g12225 g4833 g9010 g8545 g5312 g4421 g4319 g3788 g3128 g2219 g11986 g2488 g3697 |
| 59 | GO:0006996 | g15457 g6087 g5719 g16122 g13214 g637 g672 g8536 g6095 g12037 g10820 g7564 g3445 g10811 g15889 g3337 g7282 g1048 g795 g5922 g15316 g2488 g4023 g6291 g13276 g13019 g4792 g12928 g12208 g11225 |
| 60 | GO:0010468 | g5803 g1635 g5719 g14315 g15889 g1057 g8930 g7711 g475 g7873 g5932 g221 g16063 g16 g4057 g12225 g672 g4833 g9010 g8545 g5312 g4421 g4319 g3788 g3128 g2219 g11986 g2488 g3697 |
| 61 | GO:0051171 | g5719 g16122 g15889 g1635 g14315 g1057 g8930 g672 g475 g7873 g5932 g221 g16063 g16 g7711 g4057 g12225 g4833 g9010 g8545 g5312 g4421 g4319 g3788 g3128 g2219 g11986 g2488 g3697 |
| 62 | GO:0051716 | g16101 g13276 g13019 g7757 g7095 g6222 g5535 g4857 g4328 g2929 g2215 g15737 g3374 g15457 g15889 g7134 g2852 g5674 g16006 g15421 g2080 g2682 g8648 g10823 g15891 g3810 g10820 g1774 g8930 |
| 63 | GO:1901362 | g2139 g3987 g1635 g6087 g5719 g7749 g504 g2509 g7564 g16055 g6012 g9081 g9820 g759 g2488 g3788 g10182 g502 g15889 g7432 g3810 g8896 g11145 g7249 g10143 g4821 g4286 g8806 g4762 |
| 64 | GO:0009889 | g1635 g5719 g14315 g15889 g1057 g8930 g475 g7873 g5932 g221 g16063 g16 g4057 g12225 g672 g4833 g9010 g8545 g5312 g4421 g4319 g3788 g3128 g2219 g11986 g2488 g3697 g16122 |
| 65 | GO:0010556 | g1635 g5719 g14315 g15889 g1057 g8930 g475 g7873 g5932 g221 g16063 g16 g4057 g12225 g672 g4833 g9010 g8545 g5312 g4421 g4319 g3788 g3128 g2219 g11986 g2488 g3697 g16122 |
| 66 | GO:0018130 | g2139 g3987 g1635 g6087 g5719 g7749 g504 g2509 g7564 g16055 g6012 g9081 g759 g2488 g3788 g4821 g10182 g502 g15889 g7432 g3810 g8896 g11145 g7249 g10143 g4286 g8806 g4762 |
| 67 | GO:0031326 | g1635 g5719 g14315 g15889 g1057 g8930 g475 g7873 g5932 g221 g16063 g16 g4057 g12225 g672 g4833 g9010 g8545 g5312 g4421 g4319 g3788 g3128 g2219 g11986 g2488 g3697 g16122 |
| 68 | GO:2000112 | g1635 g5719 g14315 g15889 g1057 g8930 g475 g7873 g5932 g221 g16063 g16 g4057 g12225 g672 g4833 g9010 g8545 g5312 g4421 g4319 g3788 g3128 g2219 g11986 g2488 g3697 g16122 |
| 69 | GO:0006520 | g1559 g3987 g10143 g918 g2888 g6095 g5806 g9820 g3076 g3988 g1994 g7225 g502 g4061 g8625 g4580 g7449 g5903 g15134 g6012 g4762 g2451 g7249 g3274 g15480 g11041 g4286 |
| 70 | GO:0009056 | g8896 g11042 g4289 g5849 g5674 g10143 g1598 g5806 g11330 g15457 g9209 g577 g3457 g10152 g5886 g11568 g12146 g15134 g14381 g7594 g7453 g14291 g4365 g3137 g2194 g4970 g1333 |
| 71 | GO:0019219 | g5719 g16122 g15889 g1635 g14315 g1057 g8930 g672 g7873 g5932 g221 g16063 g16 g7711 g4833 g9010 g8545 g5312 g4421 g4319 g3788 g3128 g2219 g11986 g2488 g3697 |
| 72 | GO:0019438 | g2139 g3987 g1635 g6087 g5719 g7749 g504 g2509 g7564 g16055 g6012 g9081 g9820 g759 g2488 g3788 g10182 g502 g15889 g7432 g3810 g8896 g11145 g10143 g4286 g8806 |
| 73 | GO:1901575 | g8896 g11042 g4289 g5849 g5674 g10143 g1598 g5806 g11330 g9209 g577 g3457 g10152 g5886 g11568 g12146 g15134 g14381 g7594 g7453 g14291 g4365 g3137 g2194 g4970 g1333 |
| 74 | GO:0051252 | g1635 g5719 g14315 g15889 g1057 g8930 g7873 g5932 g221 g16063 g16 g7711 g672 g4833 g9010 g8545 g5312 g4421 g4319 g3788 g3128 g2219 g11986 g2488 g3697 |
| 75 | GO:0006259 | g16161 g7729 g7569 g746 g433 g323 g13276 g10737 g672 g5719 g16122 g3374 g15889 g11554 g7432 g5393 g3810 g3693 g10820 g15421 g2682 g15891 g7908 g1377 |
| 76 | GO:0006355 | g1635 g5719 g14315 g15889 g1057 g8930 g7873 g5932 g221 g16063 g16 g672 g4833 g9010 g8545 g5312 g4421 g4319 g3788 g3128 g2219 g11986 g2488 g3697 |
| 77 | GO:0051641 | g6710 g3250 g3247 g7665 g6814 g4537 g15457 g5997 g12425 g7564 g12208 g5719 g15306 g15714 g8930 g9797 g2595 g8610 g795 g15889 g7746 g3137 g7282 g15925 |
| 78 | GO:1903506 | g1635 g5719 g14315 g15889 g1057 g8930 g7873 g5932 g221 g16063 g16 g672 g4833 g9010 g8545 g5312 g4421 g4319 g3788 g3128 g2219 g11986 g2488 g3697 |
| 79 | GO:2001141 | g1635 g5719 g14315 g15889 g1057 g8930 g7873 g5932 g221 g16063 g16 g672 g4833 g9010 g8545 g5312 g4421 g4319 g3788 g3128 g2219 g11986 g2488 g3697 |
| 80 | GO:0043933 | g6087 g5719 g16122 g637 g8536 g12037 g3788 g15889 g6138 g4773 g3822 g6125 g7282 g672 g5922 g15316 g2488 g4792 g12928 g11225 g12573 g4832 g5674 |
| 81 | GO:0051649 | g6710 g3250 g3247 g7665 g6814 g4537 g15457 g5997 g12425 g7564 g12208 g5719 g15306 g15714 g9797 g2595 g8610 g795 g15889 g7746 g3137 g7282 g15925 |
| 82 | GO:0034654 | g2139 g1635 g6087 g5719 g7749 g7564 g16055 g6012 g9081 g759 g2488 g3788 g10182 g15889 g7432 g3810 g8896 g504 g2509 g11145 g10143 g8806 |
| 83 | GO:0044248 | g8896 g11042 g4289 g5849 g5674 g10143 g1598 g5806 g11330 g9209 g577 g3457 g10152 g15134 g7594 g7453 g14291 g3137 g2194 g4970 g1333 |
| 84 | GO:0006508 | g11042 g5674 g1347 g9209 g577 g3457 g10152 g3137 g4643 g6599 g6579 g3945 g3115 g2697 g15737 g1539 g13874 g13864 g12146 g11549 |
| 85 | GO:0019637 | g2139 g7749 g6012 g1110 g4821 g15829 g8896 g6334 g2737 g4211 g16101 g14381 g504 g2509 g11145 g7416 g10143 g5678 g2971 |
| 86 | GO:0071702 | g6710 g7665 g6814 g9672 g12425 g3660 g12208 g15714 g6164 g5237 g8610 g2737 g13911 g13320 g10417 g795 g8942 g15889 g3137 |
| 87 | GO:0006412 | g4832 g6095 g904 g12225 g4061 g8771 g7300 g5417 g2983 g16140 g7633 g9081 g759 g7518 g3788 g2556 g11041 g12573 |
| 88 | GO:0006518 | g4832 g6095 g904 g12225 g4061 g8771 g7300 g5417 g2983 g16140 g7633 g9081 g759 g7518 g3788 g2556 g11041 g12573 |
| 89 | GO:0043043 | g4832 g6095 g904 g12225 g4061 g8771 g7300 g5417 g2983 g16140 g7633 g9081 g759 g7518 g3788 g2556 g11041 g12573 |
| 90 | GO:0043603 | g4832 g6095 g904 g12225 g4061 g8771 g7300 g5417 g2983 g16140 g7633 g9081 g759 g7518 g3788 g2556 g11041 g12573 |
| 91 | GO:0043604 | g4832 g6095 g904 g12225 g4061 g8771 g7300 g5417 g2983 g16140 g7633 g9081 g759 g7518 g3788 g2556 g11041 g12573 |
| 92 | GO:0044085 | g16047 g6376 g6593 g6138 g3822 g6125 g7282 g6281 g1160 g1635 g2324 g4792 g12928 g3788 g15925 g12573 g4832 g5674 |
| 93 | GO:0046907 | g6710 g7665 g6814 g4537 g15457 g5997 g12425 g12208 g15306 g15714 g9797 g2595 g8610 g795 g15889 g3137 g7282 g15925 |
| 94 | GO:1901135 | g2139 g8896 g1160 g6012 g1110 g504 g11145 g10348 g15829 g4435 g6334 g2737 g4211 g14381 g2509 g8806 g2971 g8631 |
| 95 | GO:1901605 | g1559 g3987 g10143 g5806 g3988 g7225 g1994 g502 g8625 g4580 g15134 g6012 g4762 g2451 g7249 g3274 g15480 g4286 |
| 96 | GO:0006468 | g15889 g5803 g5755 g12258 g9743 g8930 g7711 g7134 g4311 g3128 g2610 g2229 g1571 g1467 g12412 g10901 g12080 |
| 97 | GO:0007154 | g16101 g7757 g7095 g6222 g5535 g4857 g4328 g2929 g2215 g15737 g15457 g7134 g2852 g2080 g8648 g10823 g1774 |
| 98 | GO:0007165 | g16101 g7757 g7095 g6222 g5535 g4857 g4328 g2929 g2215 g15737 g15457 g7134 g2852 g2080 g8648 g10823 g1774 |
| 99 | GO:0009057 | g11042 g4289 g5674 g11330 g9209 g577 g3457 g10152 g5886 g11568 g12146 g7594 g7453 g3137 g2194 g4970 g1333 |
| 100 | GO:0023052 | g16101 g7757 g7095 g6222 g5535 g4857 g4328 g2929 g2215 g15737 g15457 g7134 g2852 g2080 g8648 g10823 g1774 |
| 101 | GO:0044700 | g16101 g7757 g7095 g6222 g5535 g4857 g4328 g2929 g2215 g15737 g15457 g7134 g2852 g2080 g8648 g10823 g1774 |
| 102 | GO:0006396 | g8736 g4970 g4782 g11117 g3857 g16006 g6138 g3822 g6125 g15714 g7282 g6281 g1635 g2324 g6254 g3076 |
| 103 | GO:0006811 | g15911 g6962 g10983 g1553 g12208 g11825 g9672 g12425 g6522 g12434 g1203 g3866 g4153 g11986 g7359 g9915 |
| 104 | GO:0044283 | g5932 g1559 g9820 g3076 g3988 g7225 g502 g10711 g15455 g8625 g4580 g7449 g5903 g7249 g4286 g10143 |
| 105 | GO:0051276 | g6087 g5719 g16122 g13214 g8536 g12037 g10820 g10811 g15889 g672 g5922 g15316 g2488 g6291 g13276 g13019 |
| 106 | GO:0016053 | g1559 g9820 g3076 g3988 g7225 g502 g10711 g15455 g8625 g4580 g7449 g5903 g7249 g4286 g10143 |
| 107 | GO:0046394 | g1559 g9820 g3076 g3988 g7225 g502 g10711 g15455 g8625 g4580 g7449 g5903 g7249 g4286 g10143 |
| 108 | GO:1902582 | g6710 g4537 g15457 g12425 g7665 g12208 g15714 g9797 g2595 g8610 g795 g15889 g3137 g7282 g15925 |
| 109 | GO:1902589 | g15457 g6087 g5719 g16122 g13214 g637 g672 g8536 g6095 g12037 g10820 g7564 g3337 g1048 g11225 |
| 110 | GO:0033036 | g6710 g7665 g6814 g12208 g15714 g8930 g2737 g13911 g13320 g10417 g795 g8942 g15889 g3137 |
| 111 | GO:0055086 | g2139 g8896 g7749 g504 g2509 g6012 g1110 g11145 g10348 g6334 g2737 g7873 g10143 g4762 |
| 112 | GO:0006629 | g1768 g11142 g7757 g3911 g7614 g10711 g15455 g4211 g16101 g4365 g7416 g5678 g2971 |
| 113 | GO:0044723 | g5932 g6254 g1768 g11142 g1160 g15452 g4623 g15829 g7594 g14291 g1685 g12130 g14321 |
| 114 | GO:0090407 | g2139 g7749 g6012 g4821 g8896 g4211 g16101 g504 g2509 g11145 g10143 g5678 g2971 |
| 115 | GO:0006950 | g13276 g13019 g3374 g15889 g15591 g5674 g16006 g15421 g2682 g15891 g3810 g10820 |
| 116 | GO:0008652 | g1559 g9820 g3076 g3988 g7225 g502 g8625 g4580 g7449 g5903 g7249 g4286 |
| 117 | GO:0016192 | g3250 g3247 g4773 g9797 g2595 g5681 g918 g7746 g637 g316 g2477 g12208 |
| 118 | GO:0022613 | g6376 g6593 g6138 g3822 g6125 g7282 g6281 g1635 g2324 g15925 g12573 g4832 |
| 119 | GO:0044265 | g11042 g4289 g5674 g9209 g577 g3457 g10152 g7453 g3137 g2194 g4970 g1333 |
| 120 | GO:0065009 | g2663 g672 g9797 g11221 g7590 g4057 g4857 g4328 g2929 g15737 g3788 g795 |
| 121 | GO:0006357 | g15889 g1057 g1635 g8930 g7873 g5932 g5719 g221 g16063 g16 g3697 |
| 122 | GO:0006753 | g2139 g7749 g6012 g1110 g8896 g6334 g2737 g504 g2509 g11145 g10143 |
| 123 | GO:0008104 | g6710 g7665 g6814 g12208 g8930 g2737 g13911 g13320 g10417 g795 g3137 |
| 124 | GO:0009117 | g2139 g7749 g6012 g1110 g8896 g6334 g2737 g504 g2509 g11145 g10143 |
| 125 | GO:0022607 | g16047 g6138 g3822 g6125 g7282 g4792 g12928 g3788 g12573 g4832 g5674 |
| 126 | GO:0032259 | g8536 g12037 g3710 g8736 g821 g7264 g3498 g6281 g2353 g8010 g6247 |
| 127 | GO:0033554 | g13276 g13019 g3374 g15889 g5674 g16006 g15421 g2682 g15891 g3810 g10820 |
| 128 | GO:0034660 | g1635 g6095 g3857 g16006 g4061 g15714 g7282 g6281 g6125 g2324 g11041 |
| 129 | GO:0044255 | g1768 g11142 g7614 g10711 g15455 g4211 g16101 g4365 g7416 g5678 g2971 |
| 130 | GO:0050790 | g2663 g672 g9797 g11221 g7590 g4057 g4857 g4328 g2929 g15737 g795 |
| 131 | GO:0006351 | g1635 g6087 g5719 g7564 g16055 g9081 g759 g2488 g3788 g15889 |
| 132 | GO:0006812 | g1553 g15911 g12208 g6522 g12434 g1203 g3866 g4153 g11986 g9915 |
| 133 | GO:0015031 | g6710 g7665 g6814 g12208 g2737 g13911 g13320 g10417 g795 g3137 |
| 134 | GO:0016482 | g6710 g12208 g15306 g15714 g8610 g795 g15889 g3137 g7282 g15925 |
| 135 | GO:0032774 | g1635 g6087 g5719 g7564 g16055 g9081 g759 g2488 g3788 g15889 |
| 136 | GO:0034622 | g6138 g3822 g6125 g7282 g4792 g12928 g3788 g12573 g4832 g5674 |
| 137 | GO:0044712 | g8896 g5849 g10143 g1598 g5806 g15457 g15134 g7594 g14291 g4365 |
| 138 | GO:0045184 | g6710 g7665 g6814 g12208 g2737 g13911 g13320 g10417 g795 g3137 |
| 139 | GO:0048518 | g637 g1057 g2663 g672 g9797 g11221 g475 g2080 g12225 g4057 |
| 140 | GO:0065003 | g6138 g3822 g6125 g7282 g4792 g12928 g3788 g12573 g4832 g5674 |
| 141 | GO:0097659 | g1635 g6087 g5719 g7564 g16055 g9081 g759 g2488 g3788 g15889 |
| 142 | GO:0006325 | g6087 g5719 g16122 g8536 g12037 g672 g5922 g15316 g2488 |
| 143 | GO:0006413 | g4832 g904 g9081 g759 g7518 g3788 g2556 g12225 g12573 |
| 144 | GO:0006974 | g13276 g13019 g3374 g15889 g15421 g2682 g15891 g3810 g10820 |
| 145 | GO:0016071 | g4289 g4782 g11117 g6138 g3822 g6125 g7453 g6254 g3076 |
| 146 | GO:0048519 | g8930 g5803 g1635 g5719 g14315 g672 g1572 g2488 g16122 |
| 147 | GO:0065008 | g11986 g637 g3445 g9478 g10820 g12208 g2399 g672 g1572 |
| 148 | GO:1901137 | g2139 g1160 g6012 g4435 g4211 g504 g2509 g8806 g2971 |
| 149 | GO:0007049 | g5719 g16122 g13214 g672 g11042 g7564 g3697 g11225 |
| 150 | GO:0008610 | g7614 g10711 g15455 g4211 g16101 g7416 g5678 g2971 |
| 151 | GO:0009064 | g1559 g3987 g3988 g8625 g6012 g4762 g2451 g7249 |
| 152 | GO:0009165 | g2139 g7749 g6012 g8896 g504 g2509 g11145 g10143 |
| 153 | GO:0009893 | g1057 g2663 g672 g9797 g11221 g475 g12225 g4057 |
| 154 | GO:0018193 | g8536 g12037 g16006 g2152 g15513 g10256 g821 g12080 |
| 155 | GO:0019439 | g8896 g4289 g10143 g15134 g7453 g2194 g4970 g1333 |
| 156 | GO:0022402 | g5719 g16122 g13214 g672 g11042 g7564 g3697 g11225 |
| 157 | GO:0030163 | g11042 g5674 g9209 g577 g3457 g10152 g12146 g3137 |
| 158 | GO:0034220 | g15911 g12208 g6962 g6522 g4153 g11986 g7359 g9915 |
| 159 | GO:0034470 | g3857 g16006 g15714 g7282 g6281 g6125 g1635 g2324 |
| 160 | GO:0042221 | g9478 g15156 g6410 g5674 g16006 g4365 g11098 g8930 |
| 161 | GO:0043632 | g11042 g4289 g5674 g9209 g577 g3457 g10152 g3137 |
| 162 | GO:0044262 | g6254 g11330 g4623 g15829 g7594 g14291 g12130 g14321 |
| 163 | GO:0044270 | g8896 g4289 g10143 g15134 g7453 g2194 g4970 g1333 |
| 164 | GO:0046700 | g8896 g4289 g10143 g15134 g7453 g2194 g4970 g1333 |
| 165 | GO:0048522 | g637 g1057 g2663 g672 g475 g2080 g12225 g4057 |
| 166 | GO:0048523 | g8930 g1635 g5719 g14315 g672 g1572 g2488 g16122 |
| 167 | GO:0051336 | g2663 g672 g9797 g11221 g4857 g4328 g2929 g15737 |
| 168 | GO:1901293 | g2139 g7749 g6012 g8896 g504 g2509 g11145 g10143 |
| 169 | GO:1901361 | g8896 g4289 g10143 g15134 g7453 g2194 g4970 g1333 |
| 170 | GO:1901607 | g1559 g3988 g7225 g502 g8625 g4580 g7249 g4286 |
| 171 | GO:0000278 | g5719 g16122 g13214 g11042 g7564 g3697 g11225 |
| 172 | GO:0006260 | g5719 g16122 g11554 g7432 g5393 g3810 g3693 |
| 173 | GO:0006281 | g3374 g15889 g15421 g2682 g15891 g3810 g10820 |
| 174 | GO:0006366 | g6087 g5719 g9081 g759 g2488 g3788 g15889 |
| 175 | GO:0006397 | g4782 g11117 g6138 g3822 g6125 g6254 g3076 |
| 176 | GO:0006820 | g15911 g6962 g10983 g11825 g9672 g12425 g7359 |
| 177 | GO:0009892 | g5803 g1635 g5719 g14315 g8930 g672 g2488 |
| 178 | GO:0010605 | g5803 g1635 g5719 g14315 g8930 g672 g2488 |
| 179 | GO:0010629 | g5803 g1635 g5719 g14315 g8930 g672 g2488 |
| 180 | GO:0016311 | g2764 g2983 g10152 g15282 g15549 g470 g16122 |
| 181 | GO:0016568 | g6087 g16122 g8536 g12037 g5922 g15316 g672 |
| 182 | GO:0019941 | g11042 g5674 g9209 g577 g3457 g10152 g3137 |
| 183 | GO:0034613 | g6710 g7665 g6814 g12208 g8930 g795 g3137 |
| 184 | GO:0042254 | g6376 g6593 g7282 g6281 g1635 g2324 g15925 |
| 185 | GO:0042592 | g11986 g3445 g9478 g10820 g12208 g2399 g672 |
| 186 | GO:0044257 | g11042 g5674 g9209 g577 g3457 g10152 g3137 |
| 187 | GO:0051603 | g11042 g5674 g9209 g577 g3457 g10152 g3137 |
| 188 | GO:0070647 | g1347 g16006 g7307 g5633 g3457 g11042 g9209 |
| 189 | GO:0070727 | g6710 g7665 g6814 g12208 g8930 g795 g3137 |
| 190 | GO:1902580 | g6710 g7564 g12208 g795 g3137 g7282 g15925 |
| 191 | GO:1903047 | g5719 g16122 g13214 g11042 g7564 g3697 g11225 |
| 192 | GO:0006399 | g6095 g3857 g16006 g4061 g15714 g11041 |
| 193 | GO:0006511 | g11042 g9209 g577 g3457 g10152 g3137 |
| 194 | GO:0006732 | g7749 g4821 g8896 g6334 g2737 g10143 |
| 195 | GO:0006886 | g6710 g7665 g6814 g12208 g795 g3137 |
| 196 | GO:0006913 | g6710 g15306 g15714 g15889 g7282 g15925 |
| 197 | GO:0007010 | g637 g7564 g3337 g1048 g4023 g11225 |
| 198 | GO:0009116 | g8896 g6012 g1110 g504 g11145 g10348 |
| 199 | GO:0009890 | g1635 g5719 g14315 g8930 g672 g2488 |
| 200 | GO:0010558 | g1635 g5719 g14315 g8930 g672 g2488 |
| 201 | GO:0019693 | g6012 g1110 g6334 g2737 g504 g2509 |
| 202 | GO:0022618 | g6138 g3822 g6125 g7282 g12573 g4832 |
| 203 | GO:0031324 | g1635 g5719 g14315 g8930 g672 g2488 |
| 204 | GO:0031325 | g1057 g2663 g672 g475 g12225 g4057 |
| 205 | GO:0031327 | g1635 g5719 g14315 g8930 g672 g2488 |
| 206 | GO:0034655 | g8896 g4289 g7453 g2194 g4970 g1333 |
| 207 | GO:0043087 | g9797 g11221 g4857 g4328 g2929 g15737 |
| 208 | GO:0043414 | g8536 g12037 g6281 g2353 g8010 g6247 |
| 209 | GO:0044093 | g2663 g672 g9797 g11221 g4057 g3788 |
| 210 | GO:0044282 | g5849 g10143 g1598 g5806 g15134 g14291 |
| 211 | GO:0045892 | g1635 g5719 g14315 g8930 g672 g2488 |
| 212 | GO:0045934 | g1635 g5719 g14315 g8930 g672 g2488 |
| 213 | GO:0051169 | g6710 g15306 g15714 g15889 g7282 g15925 |
| 214 | GO:0051172 | g1635 g5719 g14315 g8930 g672 g2488 |
| 215 | GO:0051186 | g7749 g4821 g8896 g6334 g2737 g10143 |
| 216 | GO:0051253 | g1635 g5719 g14315 g8930 g672 g2488 |
| 217 | GO:0051640 | g7564 g5719 g7282 g3250 g3247 g15925 |
| 218 | GO:0051656 | g7564 g5719 g7282 g3250 g3247 g15925 |
| 219 | GO:0071103 | g5719 g16122 g13214 g10811 g15889 g672 |
| 220 | GO:0071822 | g637 g4773 g4792 g12928 g11225 g5674 |
| 221 | GO:0071826 | g6138 g3822 g6125 g7282 g12573 g4832 |
| 222 | GO:0090305 | g5803 g7932 g4970 g15421 g15889 g4289 |
| 223 | GO:0098655 | g15911 g12208 g6522 g4153 g11986 g9915 |
| 224 | GO:0098660 | g15911 g12208 g4153 g11986 g7359 g9915 |
| 225 | GO:1901657 | g8896 g6012 g1110 g504 g11145 g10348 |
| 226 | GO:1902679 | g1635 g5719 g14315 g8930 g672 g2488 |
| 227 | GO:1903507 | g1635 g5719 g14315 g8930 g672 g2488 |
| 228 | GO:2000113 | g1635 g5719 g14315 g8930 g672 g2488 |
| 229 | GO:0000375 | g4782 g11117 g6138 g3822 g6125 |
| 230 | GO:0000377 | g4782 g11117 g6138 g3822 g6125 |
| 231 | GO:0000398 | g4782 g11117 g6138 g3822 g6125 |
| 232 | GO:0005976 | g11330 g5886 g11568 g4623 g7594 |
| 233 | GO:0006091 | g4910 g4289 g1110 g4623 g7594 |
| 234 | GO:0006401 | g4289 g7453 g2194 g4970 g1333 |
| 235 | GO:0006470 | g2764 g2983 g10152 g470 g16122 |
| 236 | GO:0006479 | g8536 g12037 g2353 g8010 g6247 |
| 237 | GO:0006644 | g4211 g16101 g7416 g5678 g2971 |
| 238 | GO:0006733 | g7749 g8896 g6334 g2737 g10143 |
| 239 | GO:0008213 | g8536 g12037 g2353 g8010 g6247 |
| 240 | GO:0008380 | g4782 g11117 g6138 g3822 g6125 |
| 241 | GO:0009072 | g10143 g9820 g1994 g502 g15134 |
| 242 | GO:0010604 | g1057 g2663 g672 g475 g12225 |
| 243 | GO:0015698 | g15911 g6962 g10983 g11825 g7359 |
| 244 | GO:0015980 | g4910 g4289 g1110 g4623 g7594 |
| 245 | GO:0016052 | g11330 g5886 g11568 g7594 g14291 |
| 246 | GO:0019362 | g7749 g8896 g6334 g2737 g10143 |
| 247 | GO:0030001 | g1553 g15911 g3866 g4153 g11986 |
| 248 | GO:0032268 | g2663 g475 g7590 g4057 g12225 |
| 249 | GO:0032446 | g16006 g7307 g5633 g3457 g11042 |
| 250 | GO:0043085 | g2663 g672 g9797 g11221 g4057 |
| 251 | GO:0046496 | g7749 g8896 g6334 g2737 g10143 |
| 252 | GO:0051128 | g637 g1635 g12225 g16006 g1572 |
| 253 | GO:0051246 | g2663 g475 g7590 g4057 g12225 |
| 254 | GO:0071705 | g9672 g3660 g15714 g8610 g15889 |
| 255 | GO:0072521 | g8896 g504 g2509 g1110 g7873 |
| 256 | GO:0072524 | g7749 g8896 g6334 g2737 g10143 |
| 257 | GO:0098662 | g15911 g12208 g4153 g11986 g9915 |
| 258 | GO:0000272 | g11330 g5886 g11568 g7594 |
| 259 | GO:0000280 | g5719 g16122 g13214 g672 |
| 260 | GO:0006338 | g16122 g5922 g15316 g672 |
| 261 | GO:0006364 | g7282 g6281 g1635 g2324 |
| 262 | GO:0006457 | g2152 g15536 g15513 g795 |
| 263 | GO:0006605 | g6710 g12208 g795 g3137 |
| 264 | GO:0006897 | g637 g316 g2477 g12208 |
| 265 | GO:0007005 | g6095 g795 g4792 g12928 |
| 266 | GO:0007017 | g7564 g3337 g4023 g11225 |
| 267 | GO:0007034 | g4537 g15457 g12208 g3137 |
| 268 | GO:0008654 | g4211 g16101 g5678 g2971 |
| 269 | GO:0009084 | g1559 g3988 g8625 g7249 |
| 270 | GO:0009112 | g504 g2509 g7873 g4762 |
| 271 | GO:0009123 | g1110 g504 g2509 g11145 |
| 272 | GO:0009161 | g1110 g504 g2509 g11145 |
| 273 | GO:0009259 | g6012 g1110 g504 g2509 |
| 274 | GO:0009451 | g15713 g3857 g16006 g6281 |
| 275 | GO:0015672 | g12208 g4153 g11986 g9915 |
| 276 | GO:0015931 | g3660 g15714 g8610 g15889 |
| 277 | GO:0016054 | g5849 g10143 g5806 g15134 |
| 278 | GO:0016072 | g7282 g6281 g1635 g2324 |
| 279 | GO:0016458 | g5803 g5719 g672 g2488 |
| 280 | GO:0016567 | g7307 g5633 g3457 g11042 |
| 281 | GO:0032787 | g10143 g5849 g10711 g15455 |
| 282 | GO:0033365 | g6710 g12208 g795 g3137 |
| 283 | GO:0035556 | g16101 g7134 g2852 g2080 |
| 284 | GO:0046395 | g5849 g10143 g5806 g15134 |
| 285 | GO:0046486 | g16101 g4365 g5678 g2971 |
| 286 | GO:0048285 | g5719 g16122 g13214 g672 |
| 287 | GO:0048878 | g11986 g3445 g12208 g2399 |
| 288 | GO:0051052 | g5719 g16122 g15889 g672 |
| 289 | GO:0051168 | g15714 g15889 g7282 g15925 |
| 290 | GO:0051173 | g1057 g672 g475 g12225 |
| 291 | GO:0051188 | g7749 g8896 g4821 g10143 |
| 292 | GO:0051301 | g7564 g3697 g3115 g16006 |
| 293 | GO:0051345 | g2663 g672 g9797 g11221 |
| 294 | GO:0070085 | g1768 g11142 g1160 g15452 |
| 295 | GO:0071166 | g15714 g15889 g7282 g15925 |
| 296 | GO:0071426 | g15714 g15889 g7282 g15925 |
| 297 | GO:0072594 | g6710 g12208 g795 g3137 |
| 298 | GO:1901565 | g8896 g10143 g5806 g15134 |
| 299 | GO:0000003 | g672 g16006 g16122 |
| 300 | GO:0000018 | g5719 g16122 g15889 |
| 301 | GO:0000019 | g5719 g16122 g15889 |
| 302 | GO:0000070 | g5719 g16122 g13214 |
| 303 | GO:0000096 | g7225 g7449 g5903 |
| 304 | GO:0000097 | g7225 g7449 g5903 |
| 305 | GO:0000226 | g7564 g3337 g11225 |
| 306 | GO:0000413 | g2152 g15513 g10256 |
| 307 | GO:0000819 | g5719 g16122 g13214 |
| 308 | GO:0002181 | g4832 g904 g12573 |
| 309 | GO:0002183 | g4832 g904 g12573 |
| 310 | GO:0005996 | g5932 g15829 g1685 |
| 311 | GO:0006073 | g11330 g4623 g7594 |
| 312 | GO:0006081 | g6334 g2737 g5810 |
| 313 | GO:0006144 | g504 g2509 g7873 |
| 314 | GO:0006163 | g1110 g504 g2509 |
| 315 | GO:0006289 | g15889 g15421 g2682 |
| 316 | GO:0006323 | g5719 g16122 g13214 |
| 317 | GO:0006342 | g5719 g672 g2488 |
| 318 | GO:0006352 | g9081 g3788 g15889 |
| 319 | GO:0006367 | g9081 g3788 g15889 |
| 320 | GO:0006417 | g475 g4057 g12225 |
| 321 | GO:0006418 | g6095 g4061 g11041 |
| 322 | GO:0006461 | g4792 g12928 g5674 |
| 323 | GO:0006525 | g3987 g3988 g8625 |
| 324 | GO:0006541 | g6012 g4762 g2451 |
| 325 | GO:0006568 | g10143 g502 g15134 |
| 326 | GO:0006576 | g10143 g502 g15134 |
| 327 | GO:0006586 | g10143 g502 g15134 |
| 328 | GO:0006643 | g4211 g7416 g2971 |
| 329 | GO:0006650 | g16101 g5678 g2971 |
| 330 | GO:0006790 | g7225 g7449 g5903 |
| 331 | GO:0006818 | g12208 g9915 g11986 |
| 332 | GO:0006887 | g3250 g3247 g7746 |
| 333 | GO:0006979 | g15591 g5674 g16006 |
| 334 | GO:0007059 | g5719 g16122 g13214 |
| 335 | GO:0007067 | g5719 g16122 g13214 |
| 336 | GO:0007076 | g5719 g16122 g13214 |
| 337 | GO:0008033 | g3857 g16006 g15714 |
| 338 | GO:0009063 | g10143 g5806 g15134 |
| 339 | GO:0009108 | g7749 g8896 g10143 |
| 340 | GO:0009119 | g8896 g6012 g1110 |
| 341 | GO:0009124 | g504 g2509 g11145 |
| 342 | GO:0009126 | g1110 g504 g2509 |
| 343 | GO:0009150 | g1110 g504 g2509 |
| 344 | GO:0009156 | g504 g2509 g11145 |
| 345 | GO:0009167 | g1110 g504 g2509 |
| 346 | GO:0009260 | g6012 g504 g2509 |
| 347 | GO:0009308 | g10143 g502 g15134 |
| 348 | GO:0009435 | g7749 g8896 g10143 |
| 349 | GO:0009891 | g1057 g475 g12225 |
| 350 | GO:0009966 | g727 g7038 g4127 |
| 351 | GO:0010557 | g1057 g475 g12225 |
| 352 | GO:0010608 | g475 g4057 g12225 |
| 353 | GO:0010628 | g1057 g475 g12225 |
| 354 | GO:0010646 | g727 g7038 g4127 |
| 355 | GO:0015893 | g15156 g6410 g11098 |
| 356 | GO:0015992 | g12208 g9915 g11986 |
| 357 | GO:0016051 | g5932 g4623 g12130 |
| 358 | GO:0016569 | g6087 g8536 g12037 |
| 359 | GO:0016570 | g6087 g8536 g12037 |
| 360 | GO:0018208 | g2152 g15513 g10256 |
| 361 | GO:0019318 | g5932 g15829 g1685 |
| 362 | GO:0019359 | g7749 g8896 g10143 |
| 363 | GO:0019363 | g7749 g8896 g10143 |
| 364 | GO:0019674 | g7749 g8896 g10143 |
| 365 | GO:0023051 | g727 g7038 g4127 |
| 366 | GO:0030258 | g1768 g11142 g16101 |
| 367 | GO:0030261 | g5719 g16122 g13214 |
| 368 | GO:0031328 | g1057 g475 g12225 |
| 369 | GO:0032270 | g2663 g475 g12225 |
| 370 | GO:0032392 | g10811 g15889 g672 |
| 371 | GO:0032940 | g3250 g3247 g7746 |
| 372 | GO:0034248 | g475 g4057 g12225 |
| 373 | GO:0035023 | g727 g7038 g4127 |
| 374 | GO:0040029 | g5719 g672 g2488 |
| 375 | GO:0042430 | g10143 g502 g15134 |
| 376 | GO:0042493 | g15156 g6410 g11098 |
| 377 | GO:0043038 | g6095 g4061 g11041 |
| 378 | GO:0043039 | g6095 g4061 g11041 |
| 379 | GO:0043254 | g637 g1635 g1572 |
| 380 | GO:0043623 | g4792 g12928 g5674 |
| 381 | GO:0043648 | g1559 g10143 g9820 |
| 382 | GO:0043650 | g1559 g10143 g9820 |
| 383 | GO:0044042 | g11330 g4623 g7594 |
| 384 | GO:0044087 | g637 g1635 g1572 |
| 385 | GO:0044106 | g10143 g502 g15134 |
| 386 | GO:0044264 | g11330 g4623 g7594 |
| 387 | GO:0044272 | g7225 g7449 g5903 |
| 388 | GO:0044275 | g11330 g7594 g14291 |
| 389 | GO:0045017 | g16101 g5678 g2971 |
| 390 | GO:0045333 | g4910 g4289 g1110 |
| 391 | GO:0045814 | g5719 g672 g2488 |
| 392 | GO:0046112 | g504 g2509 g4762 |
| 393 | GO:0046390 | g6012 g504 g2509 |
| 394 | GO:0046467 | g4211 g7416 g2971 |
| 395 | GO:0046474 | g16101 g5678 g2971 |
| 396 | GO:0046578 | g727 g7038 g4127 |
| 397 | GO:0046903 | g3250 g3247 g7746 |
| 398 | GO:0048583 | g727 g7038 g4127 |
| 399 | GO:0050801 | g11986 g12208 g2399 |
| 400 | GO:0051056 | g727 g7038 g4127 |
| 401 | GO:0051247 | g2663 g475 g12225 |
| 402 | GO:0055080 | g11986 g12208 g2399 |
| 403 | GO:0070271 | g4792 g12928 g5674 |
| 404 | GO:0070887 | g5674 g16006 g8930 |
| 405 | GO:0072525 | g7749 g8896 g10143 |
| 406 | GO:0098656 | g6962 g15911 g7359 |
| 407 | GO:0098771 | g11986 g12208 g2399 |
| 408 | GO:0098813 | g5719 g16122 g13214 |
| 409 | GO:1901606 | g10143 g5806 g15134 |
| 410 | GO:1902531 | g727 g7038 g4127 |
| 411 | GO:1902600 | g12208 g9915 g11986 |
| 412 | GO:0000054 | g7282 g15925 |
| 413 | GO:0000082 | g3697 g11042 |
| 414 | GO:0000103 | g7449 g5903 |
| 415 | GO:0000393 | g6138 g6125 |
| 416 | GO:0000723 | g10820 g672 |
| 417 | GO:0000956 | g4289 g7453 |
| 418 | GO:0001112 | g3788 g15889 |
| 419 | GO:0001113 | g3788 g15889 |
| 420 | GO:0001120 | g3788 g15889 |
| 421 | GO:0001732 | g12573 g4832 |
| 422 | GO:0001932 | g7590 g4057 |
| 423 | GO:0005977 | g4623 g7594 |
| 424 | GO:0005984 | g6254 g12130 |
| 425 | GO:0005991 | g6254 g12130 |
| 426 | GO:0006066 | g14291 g14321 |
| 427 | GO:0006098 | g6334 g2737 |
| 428 | GO:0006099 | g4910 g4289 |
| 429 | GO:0006101 | g4910 g4289 |
| 430 | GO:0006112 | g4623 g7594 |
| 431 | GO:0006164 | g504 g2509 |
| 432 | GO:0006188 | g504 g2509 |
| 433 | GO:0006189 | g504 g2509 |
| 434 | GO:0006261 | g5719 g16122 |
| 435 | GO:0006265 | g5719 g16122 |
| 436 | GO:0006271 | g5719 g16122 |
| 437 | GO:0006310 | g672 g16122 |
| 438 | GO:0006333 | g5719 g16122 |
| 439 | GO:0006354 | g6087 g5719 |
| 440 | GO:0006368 | g6087 g5719 |
| 441 | GO:0006400 | g3857 g16006 |
| 442 | GO:0006402 | g4289 g7453 |
| 443 | GO:0006403 | g15714 g15889 |
| 444 | GO:0006405 | g15714 g15889 |
| 445 | GO:0006414 | g12225 g7633 |
| 446 | GO:0006526 | g3988 g8625 |
| 447 | GO:0006569 | g10143 g15134 |
| 448 | GO:0006623 | g12208 g3137 |
| 449 | GO:0006631 | g10711 g15455 |
| 450 | GO:0006633 | g10711 g15455 |
| 451 | GO:0006661 | g16101 g2971 |
| 452 | GO:0006664 | g4211 g2971 |
| 453 | GO:0006665 | g4211 g7416 |
| 454 | GO:0006673 | g4211 g7416 |
| 455 | GO:0006739 | g6334 g2737 |
| 456 | GO:0006797 | g15457 g15911 |
| 457 | GO:0006839 | g12425 g795 |
| 458 | GO:0006885 | g11986 g12208 |
| 459 | GO:0006903 | g3250 g3247 |
| 460 | GO:0006904 | g3250 g3247 |
| 461 | GO:0007033 | g15457 g12208 |
| 462 | GO:0007126 | g672 g16122 |
| 463 | GO:0007127 | g672 g16122 |
| 464 | GO:0007131 | g672 g16122 |
| 465 | GO:0007186 | g8648 g10823 |
| 466 | GO:0007346 | g8930 g16122 |
| 467 | GO:0008064 | g637 g1572 |
| 468 | GO:0008643 | g6164 g5237 |
| 469 | GO:0009060 | g4910 g4289 |
| 470 | GO:0009069 | g5806 g7225 |
| 471 | GO:0009073 | g9820 g502 |
| 472 | GO:0009074 | g10143 g15134 |
| 473 | GO:0009081 | g3076 g4580 |
| 474 | GO:0009082 | g3076 g4580 |
| 475 | GO:0009113 | g504 g2509 |
| 476 | GO:0009127 | g504 g2509 |
| 477 | GO:0009141 | g6012 g1110 |
| 478 | GO:0009152 | g504 g2509 |
| 479 | GO:0009168 | g504 g2509 |
| 480 | GO:0009199 | g6012 g1110 |
| 481 | GO:0009225 | g8806 g8631 |
| 482 | GO:0009247 | g4211 g2971 |
| 483 | GO:0009251 | g11330 g7594 |
| 484 | GO:0009310 | g10143 g15134 |
| 485 | GO:0009311 | g6254 g12130 |
| 486 | GO:0009636 | g9478 g4365 |
| 487 | GO:0015074 | g7908 g1377 |
| 488 | GO:0015711 | g9672 g12425 |
| 489 | GO:0015849 | g9672 g12425 |
| 490 | GO:0016197 | g7665 g3137 |
| 491 | GO:0016571 | g8536 g12037 |
| 492 | GO:0016579 | g1347 g9209 |
| 493 | GO:0017004 | g4792 g12928 |
| 494 | GO:0017038 | g6710 g795 |
| 495 | GO:0017062 | g4792 g12928 |
| 496 | GO:0018022 | g8536 g12037 |
| 497 | GO:0018205 | g8536 g12037 |
| 498 | GO:0019220 | g7590 g4057 |
| 499 | GO:0019673 | g8806 g8631 |
| 500 | GO:0019682 | g6334 g2737 |
| 501 | GO:0019725 | g9478 g12208 |
| 502 | GO:0019751 | g14291 g14321 |
| 503 | GO:0019932 | g15457 g1774 |
| 504 | GO:0022406 | g3250 g3247 |
| 505 | GO:0022414 | g672 g16122 |
| 506 | GO:0022616 | g5719 g16122 |
| 507 | GO:0030029 | g637 g1048 |
| 508 | GO:0030036 | g637 g1048 |
| 509 | GO:0030148 | g4211 g7416 |
| 510 | GO:0030259 | g1768 g11142 |
| 511 | GO:0030832 | g637 g1572 |
| 512 | GO:0030833 | g637 g1572 |
| 513 | GO:0031047 | g5803 g2488 |
| 514 | GO:0031399 | g7590 g4057 |
| 515 | GO:0032200 | g10820 g672 |
| 516 | GO:0032271 | g637 g1572 |
| 517 | GO:0032508 | g10811 g15889 |
| 518 | GO:0032535 | g637 g1572 |
| 519 | GO:0032956 | g637 g1572 |
| 520 | GO:0032970 | g637 g1572 |
| 521 | GO:0033043 | g637 g1572 |
| 522 | GO:0033108 | g4792 g12928 |
| 523 | GO:0033750 | g7282 g15925 |
| 524 | GO:0033753 | g7282 g15925 |
| 525 | GO:0034250 | g475 g12225 |
| 526 | GO:0034367 | g3788 g15889 |
| 527 | GO:0034551 | g4792 g12928 |
| 528 | GO:0034599 | g5674 g16006 |
| 529 | GO:0034637 | g4623 g12130 |
| 530 | GO:0034968 | g8536 g12037 |
| 531 | GO:0035335 | g470 g16122 |
| 532 | GO:0035825 | g672 g16122 |
| 533 | GO:0042278 | g8896 g1110 |
| 534 | GO:0042325 | g7590 g4057 |
| 535 | GO:0042402 | g10143 g15134 |
| 536 | GO:0042436 | g10143 g15134 |
| 537 | GO:0042440 | g504 g2509 |
| 538 | GO:0042537 | g10143 g15134 |
| 539 | GO:0043044 | g5922 g15316 |
| 540 | GO:0043547 | g9797 g11221 |
| 541 | GO:0043549 | g7590 g4057 |
| 542 | GO:0044247 | g11330 g7594 |
| 543 | GO:0044702 | g672 g16122 |
| 544 | GO:0044724 | g7594 g14291 |
| 545 | GO:0044770 | g11042 g3697 |
| 546 | GO:0044772 | g11042 g3697 |
| 547 | GO:0044802 | g15457 g3445 |
| 548 | GO:0044843 | g3697 g11042 |
| 549 | GO:0045727 | g475 g12225 |
| 550 | GO:0045786 | g8930 g16122 |
| 551 | GO:0045859 | g7590 g4057 |
| 552 | GO:0045930 | g8930 g16122 |
| 553 | GO:0045935 | g1057 g672 |
| 554 | GO:0046040 | g504 g2509 |
| 555 | GO:0046128 | g8896 g1110 |
| 556 | GO:0046148 | g504 g2509 |
| 557 | GO:0046218 | g10143 g15134 |
| 558 | GO:0046416 | g3274 g15480 |
| 559 | GO:0046488 | g16101 g2971 |
| 560 | GO:0046618 | g15156 g6410 |
| 561 | GO:0046942 | g9672 g12425 |
| 562 | GO:0048193 | g9797 g2595 |
| 563 | GO:0048278 | g3250 g3247 |
| 564 | GO:0050657 | g15714 g15889 |
| 565 | GO:0050658 | g15714 g15889 |
| 566 | GO:0051130 | g637 g12225 |
| 567 | GO:0051156 | g6334 g2737 |
| 568 | GO:0051174 | g7590 g4057 |
| 569 | GO:0051236 | g15714 g15889 |
| 570 | GO:0051321 | g672 g16122 |
| 571 | GO:0051338 | g7590 g4057 |
| 572 | GO:0051493 | g637 g1572 |
| 573 | GO:0051648 | g3250 g3247 |
| 574 | GO:0051650 | g3250 g3247 |
| 575 | GO:0051726 | g8930 g16122 |
| 576 | GO:0055065 | g12208 g2399 |
| 577 | GO:0055067 | g11986 g12208 |
| 578 | GO:0055070 | g12208 g2399 |
| 579 | GO:0055076 | g12208 g2399 |
| 580 | GO:0060249 | g10820 g672 |
| 581 | GO:0061024 | g15457 g3445 |
| 582 | GO:0070646 | g1347 g9209 |
| 583 | GO:0070813 | g7449 g5903 |
| 584 | GO:0070814 | g7449 g5903 |
| 585 | GO:0071428 | g7282 g15925 |
| 586 | GO:0071824 | g3788 g15889 |
| 587 | GO:0071897 | g7432 g3810 |
| 588 | GO:0072330 | g10711 g15455 |
| 589 | GO:0072350 | g4910 g4289 |
| 590 | GO:0072522 | g504 g2509 |
| 591 | GO:0072527 | g6012 g4762 |
| 592 | GO:0072528 | g6012 g4762 |
| 593 | GO:0072665 | g12208 g3137 |
| 594 | GO:0072666 | g12208 g3137 |
| 595 | GO:0090066 | g637 g1572 |
| 596 | GO:0090522 | g3250 g3247 |
| 597 | GO:0097033 | g4792 g12928 |
| 598 | GO:0098754 | g9478 g4365 |
| 599 | GO:1901136 | g8896 g14381 |
| 600 | GO:1901264 | g3660 g8610 |
| 601 | GO:1901615 | g14291 g14321 |
| 602 | GO:1903046 | g672 g16122 |
| 603 | GO:1903509 | g4211 g2971 |
| 604 | GO:1990748 | g9478 g4365 |
| 605 | GO:0000027 | g7282 |
| 606 | GO:0000032 | g1160 |
| 607 | GO:0000041 | g15911 |
| 608 | GO:0000055 | g7282 |
| 609 | GO:0000075 | g16122 |
| 610 | GO:0000083 | g3697 |
| 611 | GO:0000086 | g11042 |
| 612 | GO:0000105 | g4286 |
| 613 | GO:0000122 | g8930 |
| 614 | GO:0000132 | g7564 |
| 615 | GO:0000154 | g6281 |
| 616 | GO:0000162 | g502 |
| 617 | GO:0000183 | g5719 |
| 618 | GO:0000244 | g3822 |
| 619 | GO:0000271 | g4623 |
| 620 | GO:0000288 | g7453 |
| 621 | GO:0000290 | g7453 |
| 622 | GO:0000291 | g4289 |
| 623 | GO:0000349 | g6125 |
| 624 | GO:0000350 | g6138 |
| 625 | GO:0000387 | g3822 |
| 626 | GO:0000460 | g7282 |
| 627 | GO:0000463 | g7282 |
| 628 | GO:0000466 | g7282 |
| 629 | GO:0000470 | g7282 |
| 630 | GO:0000712 | g16122 |
| 631 | GO:0000722 | g672 |
| 632 | GO:0000902 | g9133 |
| 633 | GO:0000959 | g6095 |
| 634 | GO:0001109 | g15889 |
| 635 | GO:0001111 | g15889 |
| 636 | GO:0001172 | g2488 |
| 637 | GO:0001173 | g3788 |
| 638 | GO:0001174 | g3788 |
| 639 | GO:0001403 | g16006 |
| 640 | GO:0001510 | g6281 |
| 641 | GO:0001558 | g16006 |
| 642 | GO:0002097 | g16006 |
| 643 | GO:0002098 | g16006 |
| 644 | GO:0002143 | g16006 |
| 645 | GO:0002191 | g4832 |
| 646 | GO:0002943 | g3857 |
| 647 | GO:0005978 | g4623 |
| 648 | GO:0005980 | g7594 |
| 649 | GO:0005992 | g12130 |
| 650 | GO:0006000 | g15829 |
| 651 | GO:0006003 | g15829 |
| 652 | GO:0006006 | g5932 |
| 653 | GO:0006013 | g1685 |
| 654 | GO:0006020 | g14291 |
| 655 | GO:0006022 | g4435 |
| 656 | GO:0006023 | g4435 |
| 657 | GO:0006030 | g4435 |
| 658 | GO:0006031 | g4435 |
| 659 | GO:0006040 | g4435 |
| 660 | GO:0006056 | g1160 |
| 661 | GO:0006057 | g1160 |
| 662 | GO:0006071 | g14321 |
| 663 | GO:0006072 | g14381 |
| 664 | GO:0006094 | g5932 |
| 665 | GO:0006119 | g1110 |
| 666 | GO:0006148 | g8896 |
| 667 | GO:0006152 | g8896 |
| 668 | GO:0006206 | g4762 |
| 669 | GO:0006207 | g4762 |
| 670 | GO:0006213 | g6012 |
| 671 | GO:0006220 | g6012 |
| 672 | GO:0006221 | g6012 |
| 673 | GO:0006241 | g6012 |
| 674 | GO:0006275 | g16122 |
| 675 | GO:0006304 | g3374 |
| 676 | GO:0006307 | g3374 |
| 677 | GO:0006311 | g672 |
| 678 | GO:0006312 | g672 |
| 679 | GO:0006359 | g14315 |
| 680 | GO:0006360 | g1635 |
| 681 | GO:0006406 | g15889 |
| 682 | GO:0006409 | g15714 |
| 683 | GO:0006419 | g6095 |
| 684 | GO:0006432 | g4061 |
| 685 | GO:0006436 | g11041 |
| 686 | GO:0006448 | g12225 |
| 687 | GO:0006449 | g12225 |
| 688 | GO:0006452 | g12225 |
| 689 | GO:0006486 | g1160 |
| 690 | GO:0006493 | g1160 |
| 691 | GO:0006497 | g2971 |
| 692 | GO:0006505 | g2971 |
| 693 | GO:0006506 | g2971 |
| 694 | GO:0006515 | g5674 |
| 695 | GO:0006534 | g7225 |
| 696 | GO:0006535 | g7225 |
| 697 | GO:0006536 | g1559 |
| 698 | GO:0006537 | g1559 |
| 699 | GO:0006544 | g5806 |
| 700 | GO:0006546 | g5806 |
| 701 | GO:0006547 | g4286 |
| 702 | GO:0006551 | g4580 |
| 703 | GO:0006560 | g7249 |
| 704 | GO:0006561 | g7249 |
| 705 | GO:0006563 | g7225 |
| 706 | GO:0006575 | g15134 |
| 707 | GO:0006591 | g3988 |
| 708 | GO:0006592 | g3988 |
| 709 | GO:0006606 | g6710 |
| 710 | GO:0006626 | g795 |
| 711 | GO:0006636 | g10711 |
| 712 | GO:0006638 | g4365 |
| 713 | GO:0006639 | g4365 |
| 714 | GO:0006641 | g4365 |
| 715 | GO:0006642 | g4365 |
| 716 | GO:0006675 | g4211 |
| 717 | GO:0006687 | g4211 |
| 718 | GO:0006688 | g4211 |
| 719 | GO:0006720 | g7614 |
| 720 | GO:0006799 | g15457 |
| 721 | GO:0006813 | g4153 |
| 722 | GO:0006814 | g11986 |
| 723 | GO:0006817 | g15911 |
| 724 | GO:0006821 | g7359 |
| 725 | GO:0006828 | g15911 |
| 726 | GO:0006842 | g12425 |
| 727 | GO:0006843 | g12425 |
| 728 | GO:0006855 | g11098 |
| 729 | GO:0006862 | g8610 |
| 730 | GO:0006865 | g9672 |
| 731 | GO:0006869 | g8942 |
| 732 | GO:0006873 | g12208 |
| 733 | GO:0006875 | g12208 |
| 734 | GO:0006878 | g12208 |
| 735 | GO:0006879 | g12208 |
| 736 | GO:0006914 | g15457 |
| 737 | GO:0006928 | g7564 |
| 738 | GO:0006997 | g3445 |
| 739 | GO:0006998 | g3445 |
| 740 | GO:0007015 | g637 |
| 741 | GO:0007018 | g7564 |
| 742 | GO:0007035 | g12208 |
| 743 | GO:0007051 | g11225 |
| 744 | GO:0007052 | g11225 |
| 745 | GO:0007093 | g16122 |
| 746 | GO:0007097 | g5719 |
| 747 | GO:0007114 | g16006 |
| 748 | GO:0007163 | g7564 |
| 749 | GO:0007264 | g2852 |
| 750 | GO:0008299 | g7614 |
| 751 | GO:0008617 | g8896 |
| 752 | GO:0009070 | g7225 |
| 753 | GO:0009071 | g5806 |
| 754 | GO:0009098 | g4580 |
| 755 | GO:0009100 | g1160 |
| 756 | GO:0009101 | g1160 |
| 757 | GO:0009142 | g6012 |
| 758 | GO:0009144 | g1110 |
| 759 | GO:0009147 | g6012 |
| 760 | GO:0009148 | g6012 |
| 761 | GO:0009163 | g6012 |
| 762 | GO:0009164 | g8896 |
| 763 | GO:0009201 | g6012 |
| 764 | GO:0009205 | g1110 |
| 765 | GO:0009208 | g6012 |
| 766 | GO:0009209 | g6012 |
| 767 | GO:0009218 | g6012 |
| 768 | GO:0009220 | g6012 |
| 769 | GO:0009226 | g8806 |
| 770 | GO:0009250 | g4623 |
| 771 | GO:0009262 | g2139 |
| 772 | GO:0009263 | g2139 |
| 773 | GO:0009298 | g8806 |
| 774 | GO:0009303 | g1635 |
| 775 | GO:0009309 | g502 |
| 776 | GO:0009312 | g12130 |
| 777 | GO:0009314 | g2682 |
| 778 | GO:0009411 | g2682 |
| 779 | GO:0009416 | g2682 |
| 780 | GO:0009423 | g9820 |
| 781 | GO:0009628 | g2682 |
| 782 | GO:0009653 | g9133 |
| 783 | GO:0010035 | g8930 |
| 784 | GO:0010038 | g8930 |
| 785 | GO:0010256 | g3445 |
| 786 | GO:0010324 | g15457 |
| 787 | GO:0010389 | g8930 |
| 788 | GO:0010498 | g11042 |
| 789 | GO:0010525 | g15889 |
| 790 | GO:0010528 | g15889 |
| 791 | GO:0010562 | g4057 |
| 792 | GO:0010564 | g8930 |
| 793 | GO:0010570 | g16006 |
| 794 | GO:0010621 | g8930 |
| 795 | GO:0010638 | g637 |
| 796 | GO:0010639 | g1572 |
| 797 | GO:0010876 | g8942 |
| 798 | GO:0010948 | g8930 |
| 799 | GO:0010952 | g2663 |
| 800 | GO:0010972 | g8930 |
| 801 | GO:0015693 | g3866 |
| 802 | GO:0015703 | g11825 |
| 803 | GO:0015746 | g12425 |
| 804 | GO:0015748 | g8610 |
| 805 | GO:0015780 | g8610 |
| 806 | GO:0015783 | g8610 |
| 807 | GO:0015858 | g3660 |
| 808 | GO:0015942 | g5849 |
| 809 | GO:0015988 | g12208 |
| 810 | GO:0015991 | g12208 |
| 811 | GO:0016024 | g5678 |
| 812 | GO:0016042 | g4365 |
| 813 | GO:0016074 | g6125 |
| 814 | GO:0016226 | g16047 |
| 815 | GO:0016237 | g15457 |
| 816 | GO:0016480 | g14315 |
| 817 | GO:0016973 | g15889 |
| 818 | GO:0017182 | g821 |
| 819 | GO:0017183 | g821 |
| 820 | GO:0018108 | g12080 |
| 821 | GO:0018117 | g16006 |
| 822 | GO:0018175 | g16006 |
| 823 | GO:0018192 | g16006 |
| 824 | GO:0018198 | g16006 |
| 825 | GO:0018202 | g821 |
| 826 | GO:0018212 | g12080 |
| 827 | GO:0018307 | g16006 |
| 828 | GO:0019310 | g14291 |
| 829 | GO:0019319 | g5932 |
| 830 | GO:0019344 | g7225 |
| 831 | GO:0019357 | g8896 |
| 832 | GO:0019358 | g8896 |
| 833 | GO:0019365 | g8896 |
| 834 | GO:0019400 | g14321 |
| 835 | GO:0019441 | g15134 |
| 836 | GO:0019722 | g1774 |
| 837 | GO:0019805 | g10143 |
| 838 | GO:0019856 | g4762 |
| 839 | GO:0019954 | g16006 |
| 840 | GO:0022411 | g4773 |
| 841 | GO:0022900 | g1110 |
| 842 | GO:0022904 | g1110 |
| 843 | GO:0030003 | g12208 |
| 844 | GO:0030004 | g12208 |
| 845 | GO:0030010 | g7564 |
| 846 | GO:0030150 | g795 |
| 847 | GO:0030162 | g2663 |
| 848 | GO:0030243 | g11330 |
| 849 | GO:0030245 | g11330 |
| 850 | GO:0030447 | g16006 |
| 851 | GO:0030491 | g672 |
| 852 | GO:0030641 | g12208 |
| 853 | GO:0030702 | g672 |
| 854 | GO:0030837 | g1572 |
| 855 | GO:0030838 | g637 |
| 856 | GO:0031048 | g2488 |
| 857 | GO:0031055 | g16122 |
| 858 | GO:0031136 | g2080 |
| 859 | GO:0031137 | g2080 |
| 860 | GO:0031139 | g2080 |
| 861 | GO:0031146 | g11042 |
| 862 | GO:0031163 | g16047 |
| 863 | GO:0031167 | g6281 |
| 864 | GO:0031333 | g1572 |
| 865 | GO:0031334 | g637 |
| 866 | GO:0031506 | g1160 |
| 867 | GO:0031570 | g16122 |
| 868 | GO:0031929 | g2080 |
| 869 | GO:0032069 | g672 |
| 870 | GO:0032070 | g672 |
| 871 | GO:0032071 | g672 |
| 872 | GO:0032075 | g672 |
| 873 | GO:0032077 | g672 |
| 874 | GO:0032079 | g672 |
| 875 | GO:0032272 | g1572 |
| 876 | GO:0032273 | g637 |
| 877 | GO:0032447 | g16006 |
| 878 | GO:0032502 | g9133 |
| 879 | GO:0032505 | g16006 |
| 880 | GO:0032509 | g3137 |
| 881 | GO:0032511 | g3137 |
| 882 | GO:0032543 | g6095 |
| 883 | GO:0032984 | g4773 |
| 884 | GO:0032989 | g9133 |
| 885 | GO:0033013 | g3987 |
| 886 | GO:0033014 | g3987 |
| 887 | GO:0033559 | g10711 |
| 888 | GO:0033674 | g4057 |
| 889 | GO:0033683 | g15889 |
| 890 | GO:0033692 | g4623 |
| 891 | GO:0034227 | g16006 |
| 892 | GO:0034247 | g6125 |
| 893 | GO:0034314 | g637 |
| 894 | GO:0034354 | g10143 |
| 895 | GO:0034356 | g8896 |
| 896 | GO:0034427 | g4289 |
| 897 | GO:0034504 | g6710 |
| 898 | GO:0034627 | g10143 |
| 899 | GO:0034644 | g2682 |
| 900 | GO:0035268 | g1160 |
| 901 | GO:0035269 | g1160 |
| 902 | GO:0035494 | g4773 |
| 903 | GO:0035510 | g3374 |
| 904 | GO:0035725 | g11986 |
| 905 | GO:0035822 | g672 |
| 906 | GO:0036079 | g8610 |
| 907 | GO:0036085 | g8610 |
| 908 | GO:0036092 | g16101 |
| 909 | GO:0036267 | g16006 |
| 910 | GO:0036297 | g2682 |
| 911 | GO:0040001 | g7564 |
| 912 | GO:0040007 | g16006 |
| 913 | GO:0040008 | g16006 |
| 914 | GO:0040023 | g5719 |
| 915 | GO:0042026 | g795 |
| 916 | GO:0042144 | g15457 |
| 917 | GO:0042157 | g2971 |
| 918 | GO:0042158 | g2971 |
| 919 | GO:0042180 | g15134 |
| 920 | GO:0042183 | g5849 |
| 921 | GO:0042255 | g7282 |
| 922 | GO:0042273 | g7282 |
| 923 | GO:0042327 | g4057 |
| 924 | GO:0042401 | g502 |
| 925 | GO:0042435 | g502 |
| 926 | GO:0042450 | g3988 |
| 927 | GO:0042454 | g8896 |
| 928 | GO:0042455 | g6012 |
| 929 | GO:0042546 | g1160 |
| 930 | GO:0042773 | g1110 |
| 931 | GO:0042790 | g1635 |
| 932 | GO:0043094 | g8896 |
| 933 | GO:0043144 | g6125 |
| 934 | GO:0043161 | g11042 |
| 935 | GO:0043162 | g3137 |
| 936 | GO:0043173 | g8896 |
| 937 | GO:0043241 | g4773 |
| 938 | GO:0043243 | g12225 |
| 939 | GO:0043244 | g12225 |
| 940 | GO:0043328 | g3137 |
| 941 | GO:0043388 | g3788 |
| 942 | GO:0043413 | g1160 |
| 943 | GO:0043420 | g10143 |
| 944 | GO:0043545 | g4821 |
| 945 | GO:0043624 | g4773 |
| 946 | GO:0043900 | g2080 |
| 947 | GO:0043902 | g2080 |
| 948 | GO:0044036 | g1160 |
| 949 | GO:0044038 | g1160 |
| 950 | GO:0044089 | g637 |
| 951 | GO:0044182 | g16006 |
| 952 | GO:0044210 | g6012 |
| 953 | GO:0044743 | g795 |
| 954 | GO:0044744 | g6710 |
| 955 | GO:0044767 | g9133 |
| 956 | GO:0044774 | g16122 |
| 957 | GO:0044801 | g15457 |
| 958 | GO:0044839 | g11042 |
| 959 | GO:0044845 | g1160 |
| 960 | GO:0045010 | g637 |
| 961 | GO:0045132 | g16122 |
| 962 | GO:0045324 | g3137 |
| 963 | GO:0045454 | g9478 |
| 964 | GO:0045851 | g12208 |
| 965 | GO:0045862 | g2663 |
| 966 | GO:0045893 | g1057 |
| 967 | GO:0045898 | g1635 |
| 968 | GO:0045901 | g12225 |
| 969 | GO:0045905 | g12225 |
| 970 | GO:0045937 | g4057 |
| 971 | GO:0045944 | g1057 |
| 972 | GO:0046034 | g1110 |
| 973 | GO:0046036 | g6012 |
| 974 | GO:0046083 | g504 |
| 975 | GO:0046102 | g8896 |
| 976 | GO:0046115 | g8896 |
| 977 | GO:0046130 | g8896 |
| 978 | GO:0046131 | g6012 |
| 979 | GO:0046132 | g6012 |
| 980 | GO:0046134 | g6012 |
| 981 | GO:0046164 | g14291 |
| 982 | GO:0046168 | g14381 |
| 983 | GO:0046174 | g14291 |
| 984 | GO:0046219 | g502 |
| 985 | GO:0046341 | g5678 |
| 986 | GO:0046349 | g4435 |
| 987 | GO:0046351 | g12130 |
| 988 | GO:0046364 | g5932 |
| 989 | GO:0046417 | g9820 |
| 990 | GO:0046434 | g14381 |
| 991 | GO:0046497 | g8896 |
| 992 | GO:0046834 | g16101 |
| 993 | GO:0046835 | g15829 |
| 994 | GO:0046854 | g16101 |
| 995 | GO:0046874 | g10143 |
| 996 | GO:0046916 | g12208 |
| 997 | GO:0046950 | g1598 |
| 998 | GO:0046952 | g1598 |
| 999 | GO:0046999 | g2080 |
| 1000 | GO:0048015 | g16101 |
| 1001 | GO:0048016 | g15457 |
| 1002 | GO:0048017 | g16101 |
| 1003 | GO:0048284 | g15457 |
| 1004 | GO:0048856 | g9133 |
| 1005 | GO:0048869 | g9133 |
| 1006 | GO:0050793 | g15911 |
| 1007 | GO:0051012 | g7564 |
| 1008 | GO:0051028 | g15889 |
| 1009 | GO:0051031 | g15714 |
| 1010 | GO:0051054 | g672 |
| 1011 | GO:0051098 | g3788 |
| 1012 | GO:0051099 | g3788 |
| 1013 | GO:0051101 | g3788 |
| 1014 | GO:0051123 | g3788 |
| 1015 | GO:0051129 | g1572 |
| 1016 | GO:0051131 | g5674 |
| 1017 | GO:0051170 | g6710 |
| 1018 | GO:0051189 | g4821 |
| 1019 | GO:0051254 | g1057 |
| 1020 | GO:0051273 | g11330 |
| 1021 | GO:0051275 | g11330 |
| 1022 | GO:0051293 | g7564 |
| 1023 | GO:0051294 | g7564 |
| 1024 | GO:0051304 | g16122 |
| 1025 | GO:0051306 | g16122 |
| 1026 | GO:0051307 | g16122 |
| 1027 | GO:0051347 | g4057 |
| 1028 | GO:0051452 | g12208 |
| 1029 | GO:0051453 | g12208 |
| 1030 | GO:0051494 | g1572 |
| 1031 | GO:0051495 | g637 |
| 1032 | GO:0051568 | g8536 |
| 1033 | GO:0051592 | g8930 |
| 1034 | GO:0051604 | g10700 |
| 1035 | GO:0051647 | g5719 |
| 1036 | GO:0051653 | g7564 |
| 1037 | GO:0051999 | g4211 |
| 1038 | GO:0052547 | g2663 |
| 1039 | GO:0052646 | g14381 |
| 1040 | GO:0052803 | g4286 |
| 1041 | GO:0055072 | g12208 |
| 1042 | GO:0055082 | g12208 |
| 1043 | GO:0055088 | g3445 |
| 1044 | GO:0060260 | g1635 |
| 1045 | GO:0061025 | g15457 |
| 1046 | GO:0065002 | g795 |
| 1047 | GO:0065004 | g3788 |
| 1048 | GO:0070127 | g6095 |
| 1049 | GO:0070143 | g6095 |
| 1050 | GO:0070189 | g15134 |
| 1051 | GO:0070407 | g5674 |
| 1052 | GO:0070585 | g795 |
| 1053 | GO:0070589 | g1160 |
| 1054 | GO:0070783 | g16006 |
| 1055 | GO:0070784 | g16006 |
| 1056 | GO:0070816 | g15889 |
| 1057 | GO:0070838 | g3866 |
| 1058 | GO:0070897 | g3788 |
| 1059 | GO:0070914 | g2682 |
| 1060 | GO:0070925 | g7282 |
| 1061 | GO:0071108 | g1347 |
| 1062 | GO:0071214 | g2682 |
| 1063 | GO:0071241 | g8930 |
| 1064 | GO:0071248 | g8930 |
| 1065 | GO:0071277 | g8930 |
| 1066 | GO:0071421 | g15911 |
| 1067 | GO:0071427 | g15889 |
| 1068 | GO:0071431 | g15714 |
| 1069 | GO:0071478 | g2682 |
| 1070 | GO:0071482 | g2682 |
| 1071 | GO:0071528 | g15714 |
| 1072 | GO:0071554 | g1160 |
| 1073 | GO:0071804 | g4153 |
| 1074 | GO:0071805 | g4153 |
| 1075 | GO:0071806 | g795 |
| 1076 | GO:0072329 | g5849 |
| 1077 | GO:0072511 | g3866 |
| 1078 | GO:0072523 | g8896 |
| 1079 | GO:0072530 | g8610 |
| 1080 | GO:0072655 | g795 |
| 1081 | GO:0090329 | g16122 |
| 1082 | GO:0090342 | g15911 |
| 1083 | GO:0090480 | g8610 |
| 1084 | GO:0090501 | g4289 |
| 1085 | GO:0090503 | g4289 |
| 1086 | GO:0090662 | g12208 |
| 1087 | GO:0097046 | g16122 |
| 1088 | GO:0097080 | g15911 |
| 1089 | GO:0097428 | g10700 |
| 1090 | GO:0097502 | g1160 |
| 1091 | GO:0097576 | g15457 |
| 1092 | GO:0098661 | g7359 |
| 1093 | GO:0098781 | g1635 |
| 1094 | GO:0098822 | g16006 |
| 1095 | GO:0098869 | g9478 |
| 1096 | GO:1900062 | g15911 |
| 1097 | GO:1900428 | g16006 |
| 1098 | GO:1901068 | g8896 |
| 1099 | GO:1901069 | g8896 |
| 1100 | GO:1901071 | g4435 |
| 1101 | GO:1901073 | g4435 |
| 1102 | GO:1901255 | g2682 |
| 1103 | GO:1901616 | g14291 |
| 1104 | GO:1901642 | g3660 |
| 1105 | GO:1901658 | g8896 |
| 1106 | GO:1901659 | g6012 |
| 1107 | GO:1901679 | g8610 |
| 1108 | GO:1901987 | g8930 |
| 1109 | GO:1901988 | g8930 |
| 1110 | GO:1901990 | g8930 |
| 1111 | GO:1901991 | g8930 |
| 1112 | GO:1902224 | g1598 |
| 1113 | GO:1902476 | g7359 |
| 1114 | GO:1902534 | g15457 |
| 1115 | GO:1902593 | g6710 |
| 1116 | GO:1902680 | g1057 |
| 1117 | GO:1902749 | g8930 |
| 1118 | GO:1902750 | g8930 |
| 1119 | GO:1903508 | g1057 |
| 1120 | GO:1990074 | g4289 |
| 1121 | GO:1990542 | g795 |
| 1122 | GO:2000142 | g1635 |
| 1123 | GO:2000220 | g16006 |
| 1124 | GO:2000241 | g2080 |
| 1125 | GO:2000243 | g2080 |
| 1126 | GO:2000621 | g16122 |
| 1127 | GO:2000677 | g3788 |
| 1128 | GO:2000679 | g3788 |

  

---

GO Directed Acycline Graph

Back Top
