# Supplementary material for: A Comprehensive Assessment of Ultraviolet-Radiation-Induced Mutations in Flammulina filiformis Using Whole-Genome Resequencing
Source: J Fungi (Basel). 2024 Mar 20;10(3):228. doi: 10.3390/jof10030228 (PMC10971301; doi:10.3390/jof10030228)
Supplement: Supplementary file 1 [file jof-10-00228-s001.zip › Supplementary Material S8/KEGG annotation/out/64381550635650.os/KO/out.htm]

out

out Pathway Enrichment

| # | Pathway | out (238) | All (238) | Pvalue | Qvalue | Pathway ID |
| 1 | Platelet activation | 4 | 4 | 1.000000 | 1.000000 | ko04611 |
| 2 | Salivary secretion | 1 | 1 | 1.000000 | 1.000000 | ko04970 |
| 3 | Drug metabolism - cytochrome P450 | 3 | 3 | 1.000000 | 1.000000 | ko00982 |
| 4 | Influenza A | 3 | 3 | 1.000000 | 1.000000 | ko05164 |
| 5 | Tryptophan metabolism | 3 | 3 | 1.000000 | 1.000000 | ko00380 |
| 6 | Folate biosynthesis | 1 | 1 | 1.000000 | 1.000000 | ko00790 |
| 7 | Viral carcinogenesis | 7 | 7 | 1.000000 | 1.000000 | ko05203 |
| 8 | Phototransduction - fly | 1 | 1 | 1.000000 | 1.000000 | ko04745 |
| 9 | Carbon fixation in photosynthetic organisms | 2 | 2 | 1.000000 | 1.000000 | ko00710 |
| 10 | Non-alcoholic fatty liver disease (NAFLD) | 4 | 4 | 1.000000 | 1.000000 | ko04932 |
| 11 | Other types of O-glycan biosynthesis | 1 | 1 | 1.000000 | 1.000000 | ko00514 |
| 12 | Estrogen signaling pathway | 3 | 3 | 1.000000 | 1.000000 | ko04915 |
| 13 | Natural killer cell mediated cytotoxicity | 2 | 2 | 1.000000 | 1.000000 | ko04650 |
| 14 | Leukocyte transendothelial migration | 3 | 3 | 1.000000 | 1.000000 | ko04670 |
| 15 | Adrenergic signaling in cardiomyocytes | 4 | 4 | 1.000000 | 1.000000 | ko04261 |
| 16 | Tight junction | 7 | 7 | 1.000000 | 1.000000 | ko04530 |
| 17 | MAPK signaling pathway - yeast | 3 | 3 | 1.000000 | 1.000000 | ko04011 |
| 18 | Arrhythmogenic right ventricular cardiomyopathy (ARVC) | 1 | 1 | 1.000000 | 1.000000 | ko05412 |
| 19 | RNA polymerase | 2 | 2 | 1.000000 | 1.000000 | ko03020 |
| 20 | Renal cell carcinoma | 2 | 2 | 1.000000 | 1.000000 | ko05211 |
| 21 | Huntington's disease | 5 | 5 | 1.000000 | 1.000000 | ko05016 |
| 22 | Endocrine and other factor-regulated calcium reabsorption | 1 | 1 | 1.000000 | 1.000000 | ko04961 |
| 23 | Pancreatic cancer | 1 | 1 | 1.000000 | 1.000000 | ko05212 |
| 24 | Pertussis | 1 | 1 | 1.000000 | 1.000000 | ko05133 |
| 25 | Calcium signaling pathway | 4 | 4 | 1.000000 | 1.000000 | ko04020 |
| 26 | p53 signaling pathway | 3 | 3 | 1.000000 | 1.000000 | ko04115 |
| 27 | Drug metabolism - other enzymes | 1 | 1 | 1.000000 | 1.000000 | ko00983 |
| 28 | Valine, leucine and isoleucine biosynthesis | 1 | 1 | 1.000000 | 1.000000 | ko00290 |
| 29 | Thyroid hormone signaling pathway | 4 | 4 | 1.000000 | 1.000000 | ko04919 |
| 30 | Metabolism of xenobiotics by cytochrome P450 | 2 | 2 | 1.000000 | 1.000000 | ko00980 |
| 31 | N-Glycan biosynthesis | 2 | 2 | 1.000000 | 1.000000 | ko00510 |
| 32 | Synaptic vesicle cycle | 4 | 4 | 1.000000 | 1.000000 | ko04721 |
| 33 | Retinol metabolism | 1 | 1 | 1.000000 | 1.000000 | ko00830 |
| 34 | Inositol phosphate metabolism | 6 | 6 | 1.000000 | 1.000000 | ko00562 |
| 35 | Glycolysis / Gluconeogenesis | 2 | 2 | 1.000000 | 1.000000 | ko00010 |
| 36 | Pathogenic Escherichia coli infection | 2 | 2 | 1.000000 | 1.000000 | ko05130 |
| 37 | Homologous recombination | 5 | 5 | 1.000000 | 1.000000 | ko03440 |
| 38 | Fc gamma R-mediated phagocytosis | 1 | 1 | 1.000000 | 1.000000 | ko04666 |
| 39 | Degradation of aromatic compounds | 1 | 1 | 1.000000 | 1.000000 | ko01220 |
| 40 | Circadian rhythm | 1 | 1 | 1.000000 | 1.000000 | ko04710 |
| 41 | Shigellosis | 3 | 3 | 1.000000 | 1.000000 | ko05131 |
| 42 | Purine metabolism | 11 | 11 | 1.000000 | 1.000000 | ko00230 |
| 43 | AGE-RAGE signaling pathway in diabetic complications | 3 | 3 | 1.000000 | 1.000000 | ko04933 |
| 44 | TGF-beta signaling pathway | 2 | 2 | 1.000000 | 1.000000 | ko04350 |
| 45 | Non-homologous end-joining | 2 | 2 | 1.000000 | 1.000000 | ko03450 |
| 46 | Ubiquinone and other terpenoid-quinone biosynthesis | 1 | 1 | 1.000000 | 1.000000 | ko00130 |
| 47 | Hypertrophic cardiomyopathy (HCM) | 1 | 1 | 1.000000 | 1.000000 | ko05410 |
| 48 | Carbon metabolism | 11 | 11 | 1.000000 | 1.000000 | ko01200 |
| 49 | Hepatitis C | 1 | 1 | 1.000000 | 1.000000 | ko05160 |
| 50 | mTOR signaling pathway | 1 | 1 | 1.000000 | 1.000000 | ko04150 |
| 51 | Epstein-Barr virus infection | 9 | 9 | 1.000000 | 1.000000 | ko05169 |
| 52 | Viral myocarditis | 2 | 2 | 1.000000 | 1.000000 | ko05416 |
| 53 | Antigen processing and presentation | 1 | 1 | 1.000000 | 1.000000 | ko04612 |
| 54 | Glucagon signaling pathway | 3 | 3 | 1.000000 | 1.000000 | ko04922 |
| 55 | Proteasome | 1 | 1 | 1.000000 | 1.000000 | ko03050 |
| 56 | Phenylalanine, tyrosine and tryptophan biosynthesis | 3 | 3 | 1.000000 | 1.000000 | ko00400 |
| 57 | Glycosphingolipid biosynthesis - globo series | 1 | 1 | 1.000000 | 1.000000 | ko00603 |
| 58 | Protein processing in endoplasmic reticulum | 8 | 8 | 1.000000 | 1.000000 | ko04141 |
| 59 | Retrograde endocannabinoid signaling | 3 | 3 | 1.000000 | 1.000000 | ko04723 |
| 60 | Chemical carcinogenesis | 1 | 1 | 1.000000 | 1.000000 | ko05204 |
| 61 | Renin secretion | 3 | 3 | 1.000000 | 1.000000 | ko04924 |
| 62 | Biosynthesis of unsaturated fatty acids | 2 | 2 | 1.000000 | 1.000000 | ko01040 |
| 63 | Amoebiasis | 1 | 1 | 1.000000 | 1.000000 | ko05146 |
| 64 | Tuberculosis | 5 | 5 | 1.000000 | 1.000000 | ko05152 |
| 65 | Porphyrin and chlorophyll metabolism | 1 | 1 | 1.000000 | 1.000000 | ko00860 |
| 66 | Toll-like receptor signaling pathway | 1 | 1 | 1.000000 | 1.000000 | ko04620 |
| 67 | Betalain biosynthesis | 1 | 1 | 1.000000 | 1.000000 | ko00965 |
| 68 | MicroRNAs in cancer | 5 | 5 | 1.000000 | 1.000000 | ko05206 |
| 69 | C5-Branched dibasic acid metabolism | 1 | 1 | 1.000000 | 1.000000 | ko00660 |
| 70 | Glycine, serine and threonine metabolism | 4 | 4 | 1.000000 | 1.000000 | ko00260 |
| 71 | Fat digestion and absorption | 1 | 1 | 1.000000 | 1.000000 | ko04975 |
| 72 | Hedgehog signaling pathway | 2 | 2 | 1.000000 | 1.000000 | ko04340 |
| 73 | mRNA surveillance pathway | 5 | 5 | 1.000000 | 1.000000 | ko03015 |
| 74 | Nitrogen metabolism | 2 | 2 | 1.000000 | 1.000000 | ko00910 |
| 75 | Pathways in cancer | 6 | 6 | 1.000000 | 1.000000 | ko05200 |
| 76 | Cell cycle - Caulobacter | 1 | 1 | 1.000000 | 1.000000 | ko04112 |
| 77 | Aminobenzoate degradation | 1 | 1 | 1.000000 | 1.000000 | ko00627 |
| 78 | Nicotinate and nicotinamide metabolism | 2 | 2 | 1.000000 | 1.000000 | ko00760 |
| 79 | Melanogenesis | 3 | 3 | 1.000000 | 1.000000 | ko04916 |
| 80 | Meiosis - yeast | 11 | 11 | 1.000000 | 1.000000 | ko04113 |
| 81 | Osteoclast differentiation | 2 | 2 | 1.000000 | 1.000000 | ko04380 |
| 82 | Phenylpropanoid biosynthesis | 2 | 2 | 1.000000 | 1.000000 | ko00940 |
| 83 | Adherens junction | 3 | 3 | 1.000000 | 1.000000 | ko04520 |
| 84 | Glycosylphosphatidylinositol(GPI)-anchor biosynthesis | 1 | 1 | 1.000000 | 1.000000 | ko00563 |
| 85 | Vibrio cholerae infection | 4 | 4 | 1.000000 | 1.000000 | ko05110 |
| 86 | Long-term potentiation | 3 | 3 | 1.000000 | 1.000000 | ko04720 |
| 87 | Fatty acid degradation | 3 | 3 | 1.000000 | 1.000000 | ko00071 |
| 88 | AMPK signaling pathway | 4 | 4 | 1.000000 | 1.000000 | ko04152 |
| 89 | DNA replication | 5 | 5 | 1.000000 | 1.000000 | ko03030 |
| 90 | Vascular smooth muscle contraction | 2 | 2 | 1.000000 | 1.000000 | ko04270 |
| 91 | Hippo signaling pathway -fly | 3 | 3 | 1.000000 | 1.000000 | ko04391 |
| 92 | Tyrosine metabolism | 4 | 4 | 1.000000 | 1.000000 | ko00350 |
| 93 | Fanconi anemia pathway | 6 | 6 | 1.000000 | 1.000000 | ko03460 |
| 94 | Ether lipid metabolism | 3 | 3 | 1.000000 | 1.000000 | ko00565 |
| 95 | RNA transport | 14 | 14 | 1.000000 | 1.000000 | ko03013 |
| 96 | Taste transduction | 1 | 1 | 1.000000 | 1.000000 | ko04742 |
| 97 | Glutathione metabolism | 3 | 3 | 1.000000 | 1.000000 | ko00480 |
| 98 | Axon guidance | 3 | 3 | 1.000000 | 1.000000 | ko04360 |
| 99 | Mucin type O-glycan biosynthesis | 1 | 1 | 1.000000 | 1.000000 | ko00512 |
| 100 | Prostate cancer | 1 | 1 | 1.000000 | 1.000000 | ko05215 |
| 101 | Morphine addiction | 3 | 3 | 1.000000 | 1.000000 | ko05032 |
| 102 | Ras signaling pathway | 5 | 5 | 1.000000 | 1.000000 | ko04014 |
| 103 | Vasopressin-regulated water reabsorption | 2 | 2 | 1.000000 | 1.000000 | ko04962 |
| 104 | Insulin secretion | 1 | 1 | 1.000000 | 1.000000 | ko04911 |
| 105 | Serotonergic synapse | 3 | 3 | 1.000000 | 1.000000 | ko04726 |
| 106 | Phagosome | 6 | 6 | 1.000000 | 1.000000 | ko04145 |
| 107 | Carbapenem biosynthesis | 1 | 1 | 1.000000 | 1.000000 | ko00332 |
| 108 | Terpenoid backbone biosynthesis | 1 | 1 | 1.000000 | 1.000000 | ko00900 |
| 109 | Transcriptional misregulation in cancers | 2 | 2 | 1.000000 | 1.000000 | ko05202 |
| 110 | Arginine biosynthesis | 3 | 3 | 1.000000 | 1.000000 | ko00220 |
| 111 | NF-kappa B signaling pathway | 2 | 2 | 1.000000 | 1.000000 | ko04064 |
| 112 | Measles | 2 | 2 | 1.000000 | 1.000000 | ko05162 |
| 113 | Amino sugar and nucleotide sugar metabolism | 10 | 10 | 1.000000 | 1.000000 | ko00520 |
| 114 | Dopaminergic synapse | 6 | 6 | 1.000000 | 1.000000 | ko04728 |
| 115 | Choline metabolism in cancer | 2 | 2 | 1.000000 | 1.000000 | ko05231 |
| 116 | Cyanoamino acid metabolism | 2 | 2 | 1.000000 | 1.000000 | ko00460 |
| 117 | Cysteine and methionine metabolism | 2 | 2 | 1.000000 | 1.000000 | ko00270 |
| 118 | Chloroalkane and chloroalkene degradation | 2 | 2 | 1.000000 | 1.000000 | ko00625 |
| 119 | alpha-Linolenic acid metabolism | 1 | 1 | 1.000000 | 1.000000 | ko00592 |
| 120 | Biosynthesis of amino acids | 12 | 12 | 1.000000 | 1.000000 | ko01230 |
| 121 | Inflammatory mediator regulation of TRP channels | 2 | 2 | 1.000000 | 1.000000 | ko04750 |
| 122 | Alcoholism | 5 | 5 | 1.000000 | 1.000000 | ko05034 |
| 123 | Dilated cardiomyopathy (DCM) | 2 | 2 | 1.000000 | 1.000000 | ko05414 |
| 124 | Cytosolic DNA-sensing pathway | 1 | 1 | 1.000000 | 1.000000 | ko04623 |
| 125 | Prion diseases | 2 | 2 | 1.000000 | 1.000000 | ko05020 |
| 126 | Cell cycle | 6 | 6 | 1.000000 | 1.000000 | ko04110 |
| 127 | PPAR signaling pathway | 2 | 2 | 1.000000 | 1.000000 | ko03320 |
| 128 | Butanoate metabolism | 1 | 1 | 1.000000 | 1.000000 | ko00650 |
| 129 | Glutamatergic synapse | 4 | 4 | 1.000000 | 1.000000 | ko04724 |
| 130 | Regulation of actin cytoskeleton | 6 | 6 | 1.000000 | 1.000000 | ko04810 |
| 131 | Fc epsilon RI signaling pathway | 1 | 1 | 1.000000 | 1.000000 | ko04664 |
| 132 | Pentose and glucuronate interconversions | 1 | 1 | 1.000000 | 1.000000 | ko00040 |
| 133 | cGMP - PKG signaling pathway | 4 | 4 | 1.000000 | 1.000000 | ko04022 |
| 134 | Legionellosis | 1 | 1 | 1.000000 | 1.000000 | ko05134 |
| 135 | Phenylalanine metabolism | 1 | 1 | 1.000000 | 1.000000 | ko00360 |
| 136 | Thyroid hormone synthesis | 1 | 1 | 1.000000 | 1.000000 | ko04918 |
| 137 | Oxytocin signaling pathway | 6 | 6 | 1.000000 | 1.000000 | ko04921 |
| 138 | Chemokine signaling pathway | 4 | 4 | 1.000000 | 1.000000 | ko04062 |
| 139 | GABAergic synapse | 5 | 5 | 1.000000 | 1.000000 | ko04727 |
| 140 | Neurotrophin signaling pathway | 1 | 1 | 1.000000 | 1.000000 | ko04722 |
| 141 | T cell receptor signaling pathway | 1 | 1 | 1.000000 | 1.000000 | ko04660 |
| 142 | Tropane, piperidine and pyridine alkaloid biosynthesis | 1 | 1 | 1.000000 | 1.000000 | ko00960 |
| 143 | Colorectal cancer | 1 | 1 | 1.000000 | 1.000000 | ko05210 |
| 144 | Arginine and proline metabolism | 3 | 3 | 1.000000 | 1.000000 | ko00330 |
| 145 | Isoquinoline alkaloid biosynthesis | 2 | 2 | 1.000000 | 1.000000 | ko00950 |
| 146 | Amyotrophic lateral sclerosis (ALS) | 2 | 2 | 1.000000 | 1.000000 | ko05014 |
| 147 | Valine, leucine and isoleucine degradation | 6 | 6 | 1.000000 | 1.000000 | ko00280 |
| 148 | Apoptosis | 3 | 3 | 1.000000 | 1.000000 | ko04210 |
| 149 | Pancreatic secretion | 1 | 1 | 1.000000 | 1.000000 | ko04972 |
| 150 | Endocytosis | 5 | 5 | 1.000000 | 1.000000 | ko04144 |
| 151 | cAMP signaling pathway | 4 | 4 | 1.000000 | 1.000000 | ko04024 |
| 152 | Glycerolipid metabolism | 3 | 3 | 1.000000 | 1.000000 | ko00561 |
| 153 | Sulfur relay system | 1 | 1 | 1.000000 | 1.000000 | ko04122 |
| 154 | Epithelial cell signaling in Helicobacter pylori infection | 3 | 3 | 1.000000 | 1.000000 | ko05120 |
| 155 | Hepatitis B | 1 | 1 | 1.000000 | 1.000000 | ko05161 |
| 156 | Ovarian Steroidogenesis | 1 | 1 | 1.000000 | 1.000000 | ko04913 |
| 157 | Notch signaling pathway | 1 | 1 | 1.000000 | 1.000000 | ko04330 |
| 158 | Ubiquitin mediated proteolysis | 7 | 7 | 1.000000 | 1.000000 | ko04120 |
| 159 | Citrate cycle (TCA cycle) | 1 | 1 | 1.000000 | 1.000000 | ko00020 |
| 160 | Rap1 signaling pathway | 3 | 3 | 1.000000 | 1.000000 | ko04015 |
| 161 | Starch and sucrose metabolism | 7 | 7 | 1.000000 | 1.000000 | ko00500 |
| 162 | Riboflavin metabolism | 1 | 1 | 1.000000 | 1.000000 | ko00740 |
| 163 | MAPK signaling pathway | 5 | 5 | 1.000000 | 1.000000 | ko04010 |
| 164 | Lysosome | 5 | 5 | 1.000000 | 1.000000 | ko04142 |
| 165 | Olfactory transduction | 2 | 2 | 1.000000 | 1.000000 | ko04740 |
| 166 | Regulation of lipolysis in adipocyte | 2 | 2 | 1.000000 | 1.000000 | ko04923 |
| 167 | Alzheimer's disease | 4 | 4 | 1.000000 | 1.000000 | ko05010 |
| 168 | Lysine degradation | 3 | 3 | 1.000000 | 1.000000 | ko00310 |
| 169 | Phosphatidylinositol signaling system | 5 | 5 | 1.000000 | 1.000000 | ko04070 |
| 170 | Chagas disease (American trypanosomiasis) | 2 | 2 | 1.000000 | 1.000000 | ko05142 |
| 171 | Rheumatoid arthritis | 2 | 2 | 1.000000 | 1.000000 | ko05323 |
| 172 | Mismatch repair | 2 | 2 | 1.000000 | 1.000000 | ko03430 |
| 173 | SNARE interactions in vesicular transport | 2 | 2 | 1.000000 | 1.000000 | ko04130 |
| 174 | Salmonella infection | 2 | 2 | 1.000000 | 1.000000 | ko05132 |
| 175 | Methane metabolism | 2 | 2 | 1.000000 | 1.000000 | ko00680 |
| 176 | Oocyte meiosis | 6 | 6 | 1.000000 | 1.000000 | ko04114 |
| 177 | Phototransduction | 1 | 1 | 1.000000 | 1.000000 | ko04744 |
| 178 | Collecting duct acid secretion | 2 | 2 | 1.000000 | 1.000000 | ko04966 |
| 179 | Base excision repair | 3 | 3 | 1.000000 | 1.000000 | ko03410 |
| 180 | Progesterone-mediated oocyte maturation | 2 | 2 | 1.000000 | 1.000000 | ko04914 |
| 181 | Herpes simplex infection | 5 | 5 | 1.000000 | 1.000000 | ko05168 |
| 182 | Basal transcription factors | 5 | 5 | 1.000000 | 1.000000 | ko03022 |
| 183 | ABC transporters | 5 | 5 | 1.000000 | 1.000000 | ko02010 |
| 184 | Glycosaminoglycan degradation | 1 | 1 | 1.000000 | 1.000000 | ko00531 |
| 185 | Proteoglycans in cancer | 4 | 4 | 1.000000 | 1.000000 | ko05205 |
| 186 | Regulation of autophagy | 1 | 1 | 1.000000 | 1.000000 | ko04140 |
| 187 | Circadian entrainment | 3 | 3 | 1.000000 | 1.000000 | ko04713 |
| 188 | VEGF signaling pathway | 2 | 2 | 1.000000 | 1.000000 | ko04370 |
| 189 | Styrene degradation | 2 | 2 | 1.000000 | 1.000000 | ko00643 |
| 190 | Aminoacyl-tRNA biosynthesis | 3 | 3 | 1.000000 | 1.000000 | ko00970 |
| 191 | Oxidative phosphorylation | 7 | 7 | 1.000000 | 1.000000 | ko00190 |
| 192 | Propanoate metabolism | 4 | 4 | 1.000000 | 1.000000 | ko00640 |
| 193 | Limonene and pinene degradation | 1 | 1 | 1.000000 | 1.000000 | ko00903 |
| 194 | Gap junction | 3 | 3 | 1.000000 | 1.000000 | ko04540 |
| 195 | Nucleotide excision repair | 7 | 7 | 1.000000 | 1.000000 | ko03420 |
| 196 | African trypanosomiasis | 1 | 1 | 1.000000 | 1.000000 | ko05143 |
| 197 | Bile secretion | 3 | 3 | 1.000000 | 1.000000 | ko04976 |
| 198 | Long-term depression | 2 | 2 | 1.000000 | 1.000000 | ko04730 |
| 199 | Parkinson's disease | 6 | 6 | 1.000000 | 1.000000 | ko05012 |
| 200 | HTLV-I infection | 7 | 7 | 1.000000 | 1.000000 | ko05166 |
| 201 | Toxoplasmosis | 2 | 2 | 1.000000 | 1.000000 | ko05145 |
| 202 | Cocaine addiction | 2 | 2 | 1.000000 | 1.000000 | ko05030 |
| 203 | FoxO signaling pathway | 3 | 3 | 1.000000 | 1.000000 | ko04068 |
| 204 | GnRH signaling pathway | 1 | 1 | 1.000000 | 1.000000 | ko04912 |
| 205 | Pentose phosphate pathway | 4 | 4 | 1.000000 | 1.000000 | ko00030 |
| 206 | Cell cycle - yeast | 15 | 15 | 1.000000 | 1.000000 | ko04111 |
| 207 | RNA degradation | 7 | 7 | 1.000000 | 1.000000 | ko03018 |
| 208 | Bacterial invasion of epithelial cells | 2 | 2 | 1.000000 | 1.000000 | ko05100 |
| 209 | Other glycan degradation | 2 | 2 | 1.000000 | 1.000000 | ko00511 |
| 210 | Fructose and mannose metabolism | 3 | 3 | 1.000000 | 1.000000 | ko00051 |
| 211 | Pyruvate metabolism | 2 | 2 | 1.000000 | 1.000000 | ko00620 |
| 212 | Glycosphingolipid biosynthesis - ganglio series | 1 | 1 | 1.000000 | 1.000000 | ko00604 |
| 213 | Sphingolipid signaling pathway | 4 | 4 | 1.000000 | 1.000000 | ko04071 |
| 214 | Gastric acid secretion | 3 | 3 | 1.000000 | 1.000000 | ko04971 |
| 215 | Synthesis and degradation of ketone bodies | 1 | 1 | 1.000000 | 1.000000 | ko00072 |
| 216 | Circadian rhythm - plant | 1 | 1 | 1.000000 | 1.000000 | ko04712 |
| 217 | Naphthalene degradation | 1 | 1 | 1.000000 | 1.000000 | ko00626 |
| 218 | Spliceosome | 13 | 13 | 1.000000 | 1.000000 | ko03040 |
| 219 | Histidine metabolism | 2 | 2 | 1.000000 | 1.000000 | ko00340 |
| 220 | PI3K-Akt signaling pathway | 5 | 5 | 1.000000 | 1.000000 | ko04151 |
| 221 | Primary immunodeficiency | 1 | 1 | 1.000000 | 1.000000 | ko05340 |
| 222 | Glyoxylate and dicarboxylate metabolism | 3 | 3 | 1.000000 | 1.000000 | ko00630 |
| 223 | Sulfur metabolism | 3 | 3 | 1.000000 | 1.000000 | ko00920 |
| 224 | Protein export | 1 | 1 | 1.000000 | 1.000000 | ko03060 |
| 225 | Alanine, aspartate and glutamate metabolism | 4 | 4 | 1.000000 | 1.000000 | ko00250 |
| 226 | Fatty acid metabolism | 2 | 2 | 1.000000 | 1.000000 | ko01212 |
| 227 | Aldosterone synthesis and secretion | 2 | 2 | 1.000000 | 1.000000 | ko04925 |
| 228 | Phosphonate and phosphinate metabolism | 1 | 1 | 1.000000 | 1.000000 | ko00440 |
| 229 | Pyrimidine metabolism | 10 | 10 | 1.000000 | 1.000000 | ko00240 |
| 230 | beta-Alanine metabolism | 3 | 3 | 1.000000 | 1.000000 | ko00410 |
| 231 | Hippo signaling pathway | 3 | 3 | 1.000000 | 1.000000 | ko04390 |
| 232 | B cell receptor signaling pathway | 2 | 2 | 1.000000 | 1.000000 | ko04662 |
| 233 | Amphetamine addiction | 3 | 3 | 1.000000 | 1.000000 | ko05031 |
| 234 | Peroxisome | 3 | 3 | 1.000000 | 1.000000 | ko04146 |
| 235 | Ascorbate and aldarate metabolism | 1 | 1 | 1.000000 | 1.000000 | ko00053 |
| 236 | Insulin signaling pathway | 3 | 3 | 1.000000 | 1.000000 | ko04910 |
| 237 | 2-Oxocarboxylic acid metabolism | 4 | 4 | 1.000000 | 1.000000 | ko01210 |
| 238 | Various types of N-glycan biosynthesis | 1 | 1 | 1.000000 | 1.000000 | ko00513 |
| 239 | Focal adhesion | 4 | 4 | 1.000000 | 1.000000 | ko04510 |
| 240 | Ribosome biogenesis in eukaryotes | 7 | 7 | 1.000000 | 1.000000 | ko03008 |
| 241 | Cholinergic synapse | 3 | 3 | 1.000000 | 1.000000 | ko04725 |
| 242 | RIG-I-like receptor signaling pathway | 1 | 1 | 1.000000 | 1.000000 | ko04622 |
| 243 | Wnt signaling pathway | 5 | 5 | 1.000000 | 1.000000 | ko04310 |
| 244 | Ribosome | 4 | 4 | 1.000000 | 1.000000 | ko03010 |
| 245 | Insulin resistance | 1 | 1 | 1.000000 | 1.000000 | ko04931 |
| 246 | Glycerophospholipid metabolism | 7 | 7 | 1.000000 | 1.000000 | ko00564 |

Pathway Detail

| # | Pathway | Differentially expressed genes |
| 1 | Platelet activation | g5191, g2983, g1118, g10823 |
| 2 | Salivary secretion | g5191 |
| 3 | Drug metabolism - cytochrome P450 | g4337, g207, g1636 |
| 4 | Influenza A | g861, g7396, g1118 |
| 5 | Tryptophan metabolism | g5810, g15134, g10143 |
| 6 | Folate biosynthesis | g2451 |
| 7 | Viral carcinogenesis | g5803, g890, g5191, g3788, g2852, g11550, g1057 |
| 8 | Phototransduction - fly | g1118 |
| 9 | Carbon fixation in photosynthetic organisms | g2888, g15835 |
| 10 | Non-alcoholic fatty liver disease (NAFLD) | g2852, g15420, g1110, g1008 |
| 11 | Other types of O-glycan biosynthesis | g1160 |
| 12 | Estrogen signaling pathway | g861, g5191, g10823 |
| 13 | Natural killer cell mediated cytotoxicity | g2852, g10152 |
| 14 | Leukocyte transendothelial migration | g2852, g1118, g10823 |
| 15 | Adrenergic signaling in cardiomyocytes | g5191, g2983, g2938, g10823 |
| 16 | Tight junction | g7746, g7590, g3250, g3247, g2938, g1118, g10823 |
| 17 | MAPK signaling pathway - yeast | g2764, g2603, g2215 |
| 18 | Arrhythmogenic right ventricular cardiomyopathy (ARVC) | g1118 |
| 19 | RNA polymerase | g2488, g16055 |
| 20 | Renal cell carcinoma | g5171, g2852 |
| 21 | Huntington's disease | g6962, g2488, g15420, g1110, g1008 |
| 22 | Endocrine and other factor-regulated calcium reabsorption | g5191 |
| 23 | Pancreatic cancer | g2852 |
| 24 | Pertussis | g10823 |
| 25 | Calcium signaling pathway | g7757, g6962, g5191, g10152 |
| 26 | p53 signaling pathway | g7177, g4829, g2139 |
| 27 | Drug metabolism - other enzymes | g10348 |
| 28 | Valine, leucine and isoleucine biosynthesis | g4580 |
| 29 | Thyroid hormone signaling pathway | g7757, g5191, g15829, g1118 |
| 30 | Metabolism of xenobiotics by cytochrome P450 | g4337, g207 |
| 31 | N-Glycan biosynthesis | g15452, g1046 |
| 32 | Synaptic vesicle cycle | g918, g602, g4773, g12208 |
| 33 | Retinol metabolism | g207 |
| 34 | Inositol phosphate metabolism | g470, g8749, g7757, g3178, g16101, g15549 |
| 35 | Glycolysis / Gluconeogenesis | g5810, g207 |
| 36 | Pathogenic Escherichia coli infection | g4023, g1118 |
| 37 | Homologous recombination | g7707, g746, g672, g323, g10820 |
| 38 | Fc gamma R-mediated phagocytosis | g2852 |
| 39 | Degradation of aromatic compounds | g207 |
| 40 | Circadian rhythm | g11042 |
| 41 | Shigellosis | g1048, g2852, g1118 |
| 42 | Purine metabolism | g11554, g8896, g7432, g6222, g504, g3810, g2509, g2488, g2139, g16055, g11145 |
| 43 | AGE-RAGE signaling pathway in diabetic complications | g1048, g7757, g2852 |
| 44 | TGF-beta signaling pathway | g2938, g11042 |
| 45 | Non-homologous end-joining | g7569, g10820 |
| 46 | Ubiquinone and other terpenoid-quinone biosynthesis | g7203 |
| 47 | Hypertrophic cardiomyopathy (HCM) | g1118 |
| 48 | Carbon metabolism | g4910, g7225, g6334, g5849, g5806, g321, g3178, g2888, g2737, g14321, g11145 |
| 49 | Hepatitis C | g2938 |
| 50 | mTOR signaling pathway | g2080 |
| 51 | Epstein-Barr virus infection | g5803, g861, g7590, g5191, g3788, g3457, g2488, g16055, g1057 |
| 52 | Viral myocarditis | g2852, g1118 |
| 53 | Antigen processing and presentation | g861 |
| 54 | Glucagon signaling pathway | g6247, g5191, g10152 |
| 55 | Proteasome | g2663 |
| 56 | Phenylalanine, tyrosine and tryptophan biosynthesis | g9820, g502, g2888 |
| 57 | Glycosphingolipid biosynthesis - globo series | g8112 |
| 58 | Protein processing in endoplasmic reticulum | g10882, g861, g7396, g577, g15104, g11042, g1046, g10417 |
| 59 | Retrograde endocannabinoid signaling | g5191, g2215, g10823 |
| 60 | Chemical carcinogenesis | g4337 |
| 61 | Renin secretion | g5191, g10823, g10152 |
| 62 | Biosynthesis of unsaturated fatty acids | g4890, g10711 |
| 63 | Amoebiasis | g5191 |
| 64 | Tuberculosis | g6599, g16101, g15549, g12208, g10152 |
| 65 | Porphyrin and chlorophyll metabolism | g3987 |
| 66 | Toll-like receptor signaling pathway | g2852 |
| 67 | Betalain biosynthesis | g10485 |
| 68 | MicroRNAs in cancer | g2080, g7177, g5564, g4343, g11098 |
| 69 | C5-Branched dibasic acid metabolism | g4580 |
| 70 | Glycine, serine and threonine metabolism | g7225, g5806, g502, g3987 |
| 71 | Fat digestion and absorption | g2888 |
| 72 | Hedgehog signaling pathway | g9802, g5191 |
| 73 | mRNA surveillance pathway | g2983, g2938, g15431, g13222, g11273 |
| 74 | Nitrogen metabolism | g1559, g2596 |
| 75 | Pathways in cancer | g5191, g5171, g4337, g2852, g2215, g10823 |
| 76 | Cell cycle - Caulobacter | g12146 |
| 77 | Aminobenzoate degradation | g15549 |
| 78 | Nicotinate and nicotinamide metabolism | g8896, g7749 |
| 79 | Melanogenesis | g5191, g10823, g10485 |
| 80 | Meiosis - yeast | g5755, g9096, g8561, g6291, g6222, g5393, g5191, g3037, g2983, g2938, g10125 |
| 81 | Osteoclast differentiation | g2852, g10152 |
| 82 | Phenylpropanoid biosynthesis | g1715, g11330 |
| 83 | Adherens junction | g7590, g2852, g1118 |
| 84 | Glycosylphosphatidylinositol(GPI)-anchor biosynthesis | g2971 |
| 85 | Vibrio cholerae infection | g602, g5191, g12208, g1118 |
| 86 | Long-term potentiation | g5191, g2983, g10152 |
| 87 | Fatty acid degradation | g5810, g4890, g207 |
| 88 | AMPK signaling pathway | g2080, g2938, g15829, g10711 |
| 89 | DNA replication | g746, g7432, g5893, g3810, g323 |
| 90 | Vascular smooth muscle contraction | g5191, g2983 |
| 91 | Hippo signaling pathway -fly | g9014, g2938, g1118 |
| 92 | Tyrosine metabolism | g2888, g207, g1994, g10485 |
| 93 | Fanconi anemia pathway | g746, g4829, g4193, g323, g11086, g1006 |
| 94 | Ether lipid metabolism | g7820, g7564, g15549 |
| 95 | RNA transport | g12573, g12213, g904, g7518, g4832, g3908, g3291, g15714, g15431, g15306, g14034, g13320, g13222, g11273 |
| 96 | Taste transduction | g5191 |
| 97 | Glutathione metabolism | g11554, g4337, g2139 |
| 98 | Axon guidance | g2852, g10823, g10152 |
| 99 | Mucin type O-glycan biosynthesis | g13503 |
| 100 | Prostate cancer (no map in kegg database) | g4337 |
| 101 | Morphine addiction | g5191, g2215, g10823 |
| 102 | Ras signaling pathway | g7395, g5191, g4857, g2852, g2215 |
| 103 | Vasopressin-regulated water reabsorption | g5191, g4773 |
| 104 | Insulin secretion | g5191 |
| 105 | Serotonergic synapse | g5191, g2215, g10823 |
| 106 | Phagosome | g602, g4023, g2852, g16101, g12208, g1118 |
| 107 | Carbapenem biosynthesis | g7249 |
| 108 | Terpenoid backbone biosynthesis | g7614 |
| 109 | Transcriptional misregulation in cancers | g7177, g5171 |
| 110 | Arginine biosynthesis | g8625, g3988, g2888 |
| 111 | NF-kappa B signaling pathway | g7590, g7177 |
| 112 | Measles | g861, g7590 |
| 113 | Amino sugar and nucleotide sugar metabolism | g4435, g8806, g8631, g8112, g5023, g4764, g2770, g2170, g11779, g11775 |
| 114 | Dopaminergic synapse | g5191, g2983, g2938, g2215, g10823, g10152 |
| 115 | Choline metabolism in cancer | g2852, g12200 |
| 116 | Cyanoamino acid metabolism | g1715, g11330 |
| 117 | Cysteine and methionine metabolism | g7225, g2888 |
| 118 | Chloroalkane and chloroalkene degradation | g5810, g207 |
| 119 | alpha-Linolenic acid metabolism | g4890 |
| 120 | Biosynthesis of amino acids | g1559, g8625, g4580, g4286, g9820, g7249, g7225, g6334, g502, g3988, g2888, g11145 |
| 121 | Inflammatory mediator regulation of TRP channels | g5191, g2983 |
| 122 | Alcoholism | g5191, g3660, g2983, g2215, g10823 |
| 123 | Dilated cardiomyopathy (DCM) | g5191, g1118 |
| 124 | Cytosolic DNA-sensing pathway | g16055 |
| 125 | Prion diseases | g861, g5191 |
| 126 | Cell cycle | g8561, g7177, g6291, g5393, g4829, g11042 |
| 127 | PPAR signaling pathway | g4890, g10711 |
| 128 | Butanoate metabolism | g1598 |
| 129 | Glutamatergic synapse | g5191, g2215, g10823, g10152 |
| 130 | Regulation of actin cytoskeleton | g1048, g4328, g2983, g2852, g15737, g1118 |
| 131 | Fc epsilon RI signaling pathway | g2852 |
| 132 | Pentose and glucuronate interconversions | g5810 |
| 133 | cGMP - PKG signaling pathway | g6962, g2983, g10823, g10152 |
| 134 | Legionellosis | g861 |
| 135 | Phenylalanine metabolism | g2888 |
| 136 | Thyroid hormone synthesis | g5191 |
| 137 | Oxytocin signaling pathway | g8930, g5191, g2983, g1118, g10823, g10152 |
| 138 | Chemokine signaling pathway | g5191, g2852, g2215, g10823 |
| 139 | GABAergic synapse | g5191, g4821, g4773, g2215, g10823 |
| 140 | Neurotrophin signaling pathway | g2852 |
| 141 | T cell receptor signaling pathway | g10152 |
| 142 | Tropane, piperidine and pyridine alkaloid biosynthesis | g2888 |
| 143 | Colorectal cancer | g2852 |
| 144 | Arginine and proline metabolism | g7249, g5810, g2888 |
| 145 | Isoquinoline alkaloid biosynthesis | g2888, g10485 |
| 146 | Amyotrophic lateral sclerosis (ALS) | g2852, g10152 |
| 147 | Valine, leucine and isoleucine degradation | g5810, g4890, g321, g3178, g2229, g1598 |
| 148 | Apoptosis | g7177, g5191, g10152 |
| 149 | Pancreatic secretion | g2852 |
| 150 | Endocytosis | g9797, g861, g4537, g3457, g3137 |
| 151 | cAMP signaling pathway | g5191, g2983, g2852, g10823 |
| 152 | Glycerolipid metabolism | g842, g5810, g14321 |
| 153 | Sulfur relay system | g16006 |
| 154 | Epithelial cell signaling in Helicobacter pylori infection | g602, g2852, g12208 |
| 155 | Hepatitis B | g890 |
| 156 | Ovarian Steroidogenesis | g5191 |
| 157 | Notch signaling pathway | g1057 |
| 158 | Ubiquitin mediated proteolysis | g890, g7307, g7221, g5633, g4031, g3457, g11042 |
| 159 | Citrate cycle (TCA cycle) | g4910 |
| 160 | Rap1 signaling pathway | g2852, g1118, g10823 |
| 161 | Starch and sucrose metabolism | g7594, g4623, g1715, g11779, g11775, g11568, g11330 |
| 162 | Riboflavin metabolism | g10485 |
| 163 | MAPK signaling pathway | g861, g5191, g4857, g2852, g10152 |
| 164 | Lysosome | g4643, g8112, g7665, g6599, g12208 |
| 165 | Olfactory transduction | g5191, g2215 |
| 166 | Regulation of lipolysis in adipocyte | g5191, g10823 |
| 167 | Alzheimer's disease | g15420, g1110, g10152, g1008 |
| 168 | Lysine degradation | g8536, g5810, g12037 |
| 169 | Phosphatidylinositol signaling system | g470, g8749, g7757, g5678, g16101 |
| 170 | Chagas disease (American trypanosomiasis) | g2938, g10823 |
| 171 | Rheumatoid arthritis | g602, g12208 |
| 172 | Mismatch repair | g746, g323 |
| 173 | SNARE interactions in vesicular transport | g918, g2883 |
| 174 | Salmonella infection | g2852, g1118 |
| 175 | Methane metabolism | g5849, g14321 |
| 176 | Oocyte meiosis | g6291, g5191, g2983, g2938, g11042, g10152 |
| 177 | Phototransduction | g2215 |
| 178 | Collecting duct acid secretion | g602, g12208 |
| 179 | Base excision repair | g15891, g3810, g11550 |
| 180 | Progesterone-mediated oocyte maturation | g5191, g10823 |
| 181 | Herpes simplex infection | g7590, g759, g319, g2983, g11042 |
| 182 | Basal transcription factors | g9081, g759, g3788, g2556, g15889 |
| 183 | ABC transporters | g5980, g562, g5564, g3034, g11098 |
| 184 | Glycosaminoglycan degradation | g8112 |
| 185 | Proteoglycans in cancer | g5191, g2983, g2852, g1118 |
| 186 | Regulation of autophagy | g16101 |
| 187 | Circadian entrainment | g5191, g2215, g10823 |
| 188 | VEGF signaling pathway | g2852, g10152 |
| 189 | Styrene degradation | g200, g1994 |
| 190 | Aminoacyl-tRNA biosynthesis | g6095, g11041, g4061 |
| 191 | Oxidative phosphorylation | g450, g602, g15420, g13321, g12208, g1110, g1008 |
| 192 | Propanoate metabolism | g4910, g321, g3178, g2229 |
| 193 | Limonene and pinene degradation | g5810 |
| 194 | Gap junction | g5191, g4023, g10823 |
| 195 | Nucleotide excision repair | g890, g746, g3810, g323, g2682, g15889, g15421 |
| 196 | African trypanosomiasis | g15134 |
| 197 | Bile secretion | g5564, g5191, g11098 |
| 198 | Long-term depression | g2938, g10823 |
| 199 | Parkinson's disease | g6962, g5191, g15420, g1110, g10823, g1008 |
| 200 | HTLV-I infection | g7177, g6962, g5191, g4829, g3810, g11550, g10152 |
| 201 | Toxoplasmosis | g861, g10823 |
| 202 | Cocaine addiction | g5191, g10823 |
| 203 | FoxO signaling pathway | g7177, g6247, g10901 |
| 204 | GnRH signaling pathway | g5191 |
| 205 | Pentose phosphate pathway | g6334, g2737, g15835, g11145 |
| 206 | Cell cycle - yeast | g5755, g9096, g8561, g7510, g6291, g5393, g4319, g4031, g3911, g2938, g15549, g13214, g11743, g11042, g10125 |
| 207 | RNA degradation | g2194, g7932, g7453, g4970, g15099, g1333, g13222 |
| 208 | Bacterial invasion of epithelial cells | g2852, g1118 |
| 209 | Other glycan degradation | g8112, g1685 |
| 210 | Fructose and mannose metabolism | g8806, g8631, g15829 |
| 211 | Pyruvate metabolism | g5810, g4665 |
| 212 | Glycosphingolipid biosynthesis - ganglio series | g8112 |
| 213 | Sphingolipid signaling pathway | g6599, g2938, g2852, g10823 |
| 214 | Gastric acid secretion | g5191, g1118, g10823 |
| 215 | Synthesis and degradation of ketone bodies | g1598 |
| 216 | Circadian rhythm - plant | g7590 |
| 217 | Naphthalene degradation | g207 |
| 218 | Spliceosome | g7709, g891, g861, g6342, g6138, g6125, g4782, g4094, g3908, g3822, g319, g2633, g11117 |
| 219 | Histidine metabolism | g4286, g5810 |
| 220 | PI3K-Akt signaling pathway | g2080, g2938, g2852, g2215, g10901 |
| 221 | Primary immunodeficiency | g15891 |
| 222 | Glyoxylate and dicarboxylate metabolism | g5849, g5806, g11270 |
| 223 | Sulfur metabolism | g7449, g5903, g7225 |
| 224 | Protein export | g10417 |
| 225 | Alanine, aspartate and glutamate metabolism | g1559, g504, g4762, g2888 |
| 226 | Fatty acid metabolism | g4890, g10711 |
| 227 | Aldosterone synthesis and secretion | g8930, g5191 |
| 228 | Phosphonate and phosphinate metabolism | g12200 |
| 229 | Pyrimidine metabolism | g11554, g8896, g7432, g6012, g4762, g3810, g2488, g2139, g16055, g10348 |
| 230 | beta-Alanine metabolism | g5810, g321, g3178 |
| 231 | Hippo signaling pathway | g2983, g2938, g1118 |
| 232 | B cell receptor signaling pathway | g2852, g10152 |
| 233 | Amphetamine addiction | g5191, g2983, g10152 |
| 234 | Peroxisome | g5980, g4890, g1119 |
| 235 | Ascorbate and aldarate metabolism | g5810 |
| 236 | Insulin signaling pathway | g2080, g5191, g2983 |
| 237 | 2-Oxocarboxylic acid metabolism | g8625, g4580, g3988, g2888 |
| 238 | Various types of N-glycan biosynthesis | g15452 |
| 239 | Focal adhesion | g1048, g2983, g2852, g1118 |
| 240 | Ribosome biogenesis in eukaryotes | g7711, g7590, g6593, g2324, g15099, g13320, g12219 |
| 241 | Cholinergic synapse | g5191, g2215, g10823 |
| 242 | RIG-I-like receptor signaling pathway | g14321 |
| 243 | Wnt signaling pathway | g7590, g5191, g2852, g11042, g10152 |
| 244 | Ribosome | g8771, g7633, g7300, g16140 |
| 245 | Insulin resistance | g2983 |
| 246 | Glycerophospholipid metabolism | g8973, g842, g7820, g5678, g15549, g14381, g12200 |

Back Top
