# Supplementary material for: A Comprehensive Assessment of Ultraviolet-Radiation-Induced Mutations in Flammulina filiformis Using Whole-Genome Resequencing
Source: J Fungi (Basel). 2024 Mar 20;10(3):228. doi: 10.3390/jof10030228 (PMC10971301; doi:10.3390/jof10030228)
Supplement: Supplementary file 1 [file jof-10-00228-s001.zip › Supplementary Material S8/KEGG annotation/out/64381550635650.os/KO/out_map/map00010.html]

KEGG PATHWAY: Glycolysis / Gluconeogenesis - Reference pathway


|  |  |
| --- | --- |
| **Glycolysis / Gluconeogenesis - Reference pathway** |  |

[
Pathway menu
| Organism menu
| Pathway entry
| Show description
| User data mapping
]

|  |
| --- |
| Glycolysis is the process of converting glucose into pyruvate and generating small amounts of ATP (energy) and NADH (reducing power). It is a central pathway that produces important precursor metabolites: six-carbon compounds of glucose-6P and fructose-6P and three-carbon compounds of glycerone-P, glyceraldehyde-3P, glycerate-3P, phosphoenolpyruvate, and pyruvate [MD:M00001]. Acetyl-CoA, another important precursor metabolite, is produced by oxidative decarboxylation of pyruvate [MD:M00307]. When the enzyme genes of this pathway are examined in completely sequenced genomes, the reaction steps of three-carbon compounds from glycerone-P to pyruvate form a conserved core module [MD:M00002], which is found in almost all organisms and which sometimes contains operon structures in bacterial genomes. Gluconeogenesis is a synthesis pathway of glucose from noncarbohydrate precursors. It is essentially a reversal of glycolysis with minor variations of alternative paths [MD:M00003]. |

|  |  |
| --- | --- |
| Reference pathway | 100% |
