# Supplementary material for: A Comprehensive Assessment of Ultraviolet-Radiation-Induced Mutations in Flammulina filiformis Using Whole-Genome Resequencing
Source: J Fungi (Basel). 2024 Mar 20;10(3):228. doi: 10.3390/jof10030228 (PMC10971301; doi:10.3390/jof10030228)
Supplement: Supplementary file 1 [file jof-10-00228-s001.zip › Supplementary Material S8/KEGG annotation/out/64381550635650.os/KO/out_map/map00020.html]

KEGG PATHWAY: Citrate cycle (TCA cycle) - Reference pathway


|  |  |
| --- | --- |
| **Citrate cycle (TCA cycle) - Reference pathway** |  |

[
Pathway menu
| Organism menu
| Pathway entry
| Show description
| User data mapping
]

|  |
| --- |
| The citrate cycle (TCA cycle, Krebs cycle) is an important aerobic pathway for the final steps of the oxidation of carbohydrates and fatty acids. The cycle starts with acetyl-CoA, the activated form of acetate, derived from glycolysis and pyruvate oxidation for carbohydrates and from beta oxidation of fatty acids. The two-carbon acetyl group in acetyl-CoA is transferred to the four-carbon compound of oxaloacetate to form the six-carbon compound of citrate. In a series of reactions two carbons in citrate are oxidized to CO2 and the reaction pathway supplies NADH for use in the oxidative phosphorylation and other metabolic processes. The pathway also supplies important precursor metabolites including 2-oxoglutarate. At the end of the cycle the remaining four-carbon part is transformed back to oxaloacetate. According to the genome sequence data, many organisms seem to lack genes for the full cycle [MD:M00009], but contain genes for specific segments [MD:M00010 M00011]. |

|  |  |
| --- | --- |
| Reference pathway | 100% |
