# Supplementary material for: A Comprehensive Assessment of Ultraviolet-Radiation-Induced Mutations in Flammulina filiformis Using Whole-Genome Resequencing
Source: J Fungi (Basel). 2024 Mar 20;10(3):228. doi: 10.3390/jof10030228 (PMC10971301; doi:10.3390/jof10030228)
Supplement: Supplementary file 1 [file jof-10-00228-s001.zip › Supplementary Material S8/KEGG annotation/out/64381550635650.os/KO/out_map/map00030.html]

KEGG PATHWAY: Pentose phosphate pathway - Reference pathway


|  |  |
| --- | --- |
| **Pentose phosphate pathway - Reference pathway** |  |

[
Pathway menu
| Organism menu
| Pathway entry
| Show description
| User data mapping
]

|  |
| --- |
| The pentose phosphate pathway is a process of glucose turnover that produces NADPH as reducing equivalents and pentoses as essential parts of nucleotides. There are two different phases in the pathway. One is irreversible oxidative phase in which glucose-6P is converted to ribulose-5P by oxidative decarboxylation, and NADPH is generated [MD:M00006]. The other is reversible non-oxidative phase in which phosphorylated sugars are interconverted to generate xylulose-5P, ribulose-5P, and ribose-5P [MD:M00007]. Phosphoribosyl pyrophosphate (PRPP) formed from ribose-5P [MD:M00005] is an activated compound used in the biosynthesis of histidine and purine/pyrimidine nucleotides. This pathway map also shows the Entner-Doudoroff pathway where 6-P-gluconate is dehydrated and then cleaved into pyruvate and glyceraldehyde-3P [MD:M00008]. |

|  |  |
| --- | --- |
| Reference pathway | 100% |
