# Supplementary material for: A Comprehensive Assessment of Ultraviolet-Radiation-Induced Mutations in Flammulina filiformis Using Whole-Genome Resequencing
Source: J Fungi (Basel). 2024 Mar 20;10(3):228. doi: 10.3390/jof10030228 (PMC10971301; doi:10.3390/jof10030228)
Supplement: Supplementary file 1 [file jof-10-00228-s001.zip › Supplementary Material S8/KEGG annotation/out/64381550635650.os/KO/out_map/map00071.html]

KEGG PATHWAY: Fatty acid degradation - Reference pathway


|  |  |
| --- | --- |
| **Fatty acid degradation - Reference pathway** |  |

[
Pathway menu
| Organism menu
| Pathway entry
| User data mapping
]

|  |  |
| --- | --- |
| Reference pathway | 100% |
