# Supplementary material for: A Comprehensive Assessment of Ultraviolet-Radiation-Induced Mutations in Flammulina filiformis Using Whole-Genome Resequencing
Source: J Fungi (Basel). 2024 Mar 20;10(3):228. doi: 10.3390/jof10030228 (PMC10971301; doi:10.3390/jof10030228)
Supplement: Supplementary file 1 [file jof-10-00228-s001.zip › Supplementary Material S8/KEGG annotation/out/64381550635650.os/KO/out_map/map00130.html]

KEGG PATHWAY: Ubiquinone and other terpenoid-quinone biosynthesis - Reference pathway


|  |  |
| --- | --- |
| **Ubiquinone and other terpenoid-quinone biosynthesis - Reference pathway** |  |

[
Pathway menu
| Organism menu
| Pathway entry
| Show description
| User data mapping
]

|  |
| --- |
| Ubiquinone (UQ), also called coenzyme Q, and plastoquinone (PQ) are electron carriers in oxidative phosphorylation and photosynthesis, respectively. The quinoid nucleus of ubiquinone is derived from the shikimate pathway; 4-hydroxybenzoate is directly formed from chorismate in bacteria, while it can be formed from either chorismate or tyrosine in yeast. The following biosynthesis of terpenoid moiety involves reactions of prenylation, decarboxylation, and three hydroxylations alternating with three methylations. The order of these reactions are somewhat different between bacteria and yeast. Phylloquinone (vitamin K1), menaquinone (vitamin K2), and tocopherol (vitamin E) are fat-soluble vitamins. Phylloquinone is a compound present in all photosynthetic plants serving as a cofactor for photosystem I-mediated electron transport. Menaquinone is an obligatory component of the electron-transfer pathway in bacteria. |

|  |  |
| --- | --- |
| Reference pathway | 100% |
