# Supplementary material for: A Comprehensive Assessment of Ultraviolet-Radiation-Induced Mutations in Flammulina filiformis Using Whole-Genome Resequencing
Source: J Fungi (Basel). 2024 Mar 20;10(3):228. doi: 10.3390/jof10030228 (PMC10971301; doi:10.3390/jof10030228)
Supplement: Supplementary file 1 [file jof-10-00228-s001.zip › Supplementary Material S8/KEGG annotation/out/64381550635650.os/KO/out_map/map00332.html]

KEGG PATHWAY: Carbapenem biosynthesis - Reference pathway


|  |  |
| --- | --- |
| **Carbapenem biosynthesis - Reference pathway** |  |

[
Pathway menu
| Organism menu
| Pathway entry
| Show description
| User data mapping
]

|  |
| --- |
| Carbapenems are broad-spectrum beta-lactam antibiotics, which are often considered as the antibiotics of last resort. A naturally occurring carbapenem, thienamycin, was first discovered in Streptomyces cattleya. This diagram shows how a simple carbapenem, carbapenem-3-carboxylate, is synthesized from malonyl-CoA and pyrroline-5-carboxylate [MD:M00675]. For structurally complex carbapenems, such as thienamycin, olivanic acid, epi-thienamycin and carbapenems of the OA-6129 group, uncertainty remains about the mechanism and timing for the inversion of the C-5 carbapenam bridgehead, the desaturation of the C-2/C-3 bond and the attachment of the C-2 and C-6 side chains. |

|  |  |
| --- | --- |
| Reference pathway | 100% |
