# Supplementary material for: A Comprehensive Assessment of Ultraviolet-Radiation-Induced Mutations in Flammulina filiformis Using Whole-Genome Resequencing
Source: J Fungi (Basel). 2024 Mar 20;10(3):228. doi: 10.3390/jof10030228 (PMC10971301; doi:10.3390/jof10030228)
Supplement: Supplementary file 1 [file jof-10-00228-s001.zip › Supplementary Material S8/KEGG annotation/out/64381550635650.os/KO/out_map/map00440.html]

KEGG PATHWAY: Phosphonate and phosphinate metabolism - Reference pathway


|  |  |
| --- | --- |
| **Phosphonate and phosphinate metabolism - Reference pathway** |  |

[
Pathway menu
| Organism menu
| Pathway entry
| Show description
| User data mapping
]

|  |
| --- |
| Natural products containing carbon-phosphorous bonds, so-called C-P compounds, are derivatives of phosphonate and phosphinate with substitution of alkyl group for hydrogen of phosphorus-hydrogen bonds. C-P compounds have been found in many organisms, but only protists and bacteria, mostly Actinobacteria, have biosynthetic capacity. A common reaction in the biosynthetic pathway is C-P bond forming reaction from phosphoenolpyruvate (PEP) to phosphonopyruvate (PnPy) catalyzed by PEP phosphomutase. 2-Aminoethylphosphonate (AEP) is the most abundant C-P compound in the natural world. AEP derivatives include phosphonoprotein, phosphonoglycan, and phosphonolipid. Other known C-P compounds are bioactive substances used in medicine (antibiotics) and agriculture (herbicide) such as fosfomycin, FR-33289, rhizocticin, and bialaphos. |

|  |  |
| --- | --- |
| Reference pathway | 100% |
