# Supplementary material for: A Comprehensive Assessment of Ultraviolet-Radiation-Induced Mutations in Flammulina filiformis Using Whole-Genome Resequencing
Source: J Fungi (Basel). 2024 Mar 20;10(3):228. doi: 10.3390/jof10030228 (PMC10971301; doi:10.3390/jof10030228)
Supplement: Supplementary file 1 [file jof-10-00228-s001.zip › Supplementary Material S8/KEGG annotation/out/64381550635650.os/KO/out_map/map00510.html]

KEGG PATHWAY: N-Glycan biosynthesis - Reference pathway


|  |  |
| --- | --- |
| **N-Glycan biosynthesis - Reference pathway** |  |

[
Pathway menu
| Organism menu
| Pathway entry
| Show description
| User data mapping
]

|  |
| --- |
| N-glycans or asparagine-linked glycans are major constituents of glycoproteins in eukaryotes. N-glycans are covalently attached to asparagine with the consensus sequence of Asn-X-Ser/Thr by an N-glycosidic bond, GlcNAc b1- Asn. Biosynthesis of N-glycans begins on the cytoplasmic face of the ER membrane with the transferase reaction of UDP-GlcNAc and the lipid-like precursor P-Dol (dolichol phosphate) to generate GlcNAc a1- PP-Dol. After sequential addition of monosaccharides by ALG glycosyltransferases [MD:M00055], the N-glycan precursor is attached by the OST (oligosaccharyltransferase) complex to the polypeptide chain that is being synthesized and translocated through the ER membrane. The protein-bound N-glycan precursor is subsequently trimmed, extended, and modified in the ER and Golgi by a complex series of reactions catalyzed by membrane-bound glycosidases and glycosyltransferases. N-glycans thus synthesized are classified into three types: high-mannose type, complex type, and hybrid type. Defects in N-glycan biosynthesis lead to a variety of human diseases known as congenital disorders of glycosylation [DS:H00118 H00119]. |

|  |  |
| --- | --- |
| Reference pathway | 100% |
