# Supplementary material for: A Comprehensive Assessment of Ultraviolet-Radiation-Induced Mutations in Flammulina filiformis Using Whole-Genome Resequencing
Source: J Fungi (Basel). 2024 Mar 20;10(3):228. doi: 10.3390/jof10030228 (PMC10971301; doi:10.3390/jof10030228)
Supplement: Supplementary file 1 [file jof-10-00228-s001.zip › Supplementary Material S8/KEGG annotation/out/64381550635650.os/KO/out_map/map00512.html]

KEGG PATHWAY: Mucin type O-Glycan biosynthesis - Reference pathway


|  |  |
| --- | --- |
| **Mucin type O-Glycan biosynthesis - Reference pathway** |  |

[
Pathway menu
| Organism menu
| Pathway entry
| Show description
| User data mapping
]

|  |
| --- |
| O-glycans are a class of glycans that modify serine or threonine residues of proteins. Biosynthesis of O-glycans starts from the transfer of N-acetylgalactosamine (GalNAc) to serine or threonine. The first GalNAc may be extended with sugars including galactose, N-acetylglucosamine, fucose, or sialic acid, but not mannose, glucose, or xylose. Depending on the sugars added, there are four common O-glycan core structures, cores 1 through 4, and an additional four, cores 5 though 8. Mucins are highly O-glycosylated glycoproteins ubiquitous in mucous secretions on cell surfaces and in body fluids. Mucin O-glycans can be branched, and many sugars or groups of sugars are antigenic. Important modifications of mucin O-glycans include O-acetylation of sialic acid and O-sulfation of galactose and N-acetylglucosamine. |

|  |  |
| --- | --- |
| Reference pathway | 100% |
