# Supplementary material for: A Comprehensive Assessment of Ultraviolet-Radiation-Induced Mutations in Flammulina filiformis Using Whole-Genome Resequencing
Source: J Fungi (Basel). 2024 Mar 20;10(3):228. doi: 10.3390/jof10030228 (PMC10971301; doi:10.3390/jof10030228)
Supplement: Supplementary file 1 [file jof-10-00228-s001.zip › Supplementary Material S8/KEGG annotation/out/64381550635650.os/KO/out_map/map00514.html]

KEGG PATHWAY: Other types of O-glycan biosynthesis - Reference pathway


|  |  |
| --- | --- |
| **Other types of O-glycan biosynthesis - Reference pathway** |  |

[
Pathway menu
| Organism menu
| Pathway entry
| Show description
| User data mapping
]

|  |
| --- |
| O-mannosyl glycans are a type of O-glycans that are found both in eukaryotes and prokaryotes. Biosynthesis of O-mannosyl glycans is initiated by the transfer of mannose from Man-P-Dol to serine or threonine residue, which is catalyzed by protein O-mannosyltransferases POMT1 and POMT2. Defects of these genes are linked to human diseases, such as muscular dystrophies caused by reduced O-mannosylation of alpha-dystroglycan in skeletal muscles [DS:H00120]. |

|  |  |
| --- | --- |
| Reference pathway | 100% |
