# Supplementary material for: A Comprehensive Assessment of Ultraviolet-Radiation-Induced Mutations in Flammulina filiformis Using Whole-Genome Resequencing
Source: J Fungi (Basel). 2024 Mar 20;10(3):228. doi: 10.3390/jof10030228 (PMC10971301; doi:10.3390/jof10030228)
Supplement: Supplementary file 1 [file jof-10-00228-s001.zip › Supplementary Material S8/KEGG annotation/out/64381550635650.os/KO/out_map/map00563.html]

KEGG PATHWAY: Glycosylphosphatidylinositol(GPI)-anchor biosynthesis - Reference pathway


|  |  |
| --- | --- |
| **Glycosylphosphatidylinositol(GPI)-anchor biosynthesis - Reference pathway** |  |

[
Pathway menu
| Organism menu
| Pathway entry
| Show description
| User data mapping
]

|  |
| --- |
| Cell surface proteins can be attached to the cell membrane via the glycolipid structure called glycosylphosphatidylinositol (GPI) anchor. Hundreds of GPI-anchored proteins have been identified in many eukaryotes ranging from protozoa and fungi to mammals. All protein-linked GPI anchors share a common core structure, characterized by the substructure Man (a1-4) GlcN (a1-6) myo-inositol-1P-lipid. Biosynthesis of GPI anchors proceeds in three stages: (i) preassembly of a GPI precursor in the ER membrane, (ii) attachment of the GPI to the C-terminus of a newly synthesized protein in the lumen of the ER, and (iii) lipid remodeling and/or carbohydrate side-chain modifications in the ER and the Golgi. Defects of GPI anchor biosynthesis gene result in a genetic disorder, paroxysmal nocturnal hemoglobinuria. |

|  |  |
| --- | --- |
| Reference pathway | 100% |
