# Supplementary material for: A Comprehensive Assessment of Ultraviolet-Radiation-Induced Mutations in Flammulina filiformis Using Whole-Genome Resequencing
Source: J Fungi (Basel). 2024 Mar 20;10(3):228. doi: 10.3390/jof10030228 (PMC10971301; doi:10.3390/jof10030228)
Supplement: Supplementary file 1 [file jof-10-00228-s001.zip › Supplementary Material S8/KEGG annotation/out/64381550635650.os/KO/out_map/map00920.html]

KEGG PATHWAY: Sulfur metabolism - Reference pathway


|  |  |
| --- | --- |
| **Sulfur metabolism - Reference pathway** |  |

[
Pathway menu
| Organism menu
| Pathway entry
| Show description
| User data mapping
]

|  |
| --- |
| Sulfur is an essential element for life and the metabolism of organic sulfur compounds plays an important role in the global sulfur cycle. Sulfur occurs in various oxidation states ranging from +6 in sulfate to -2 in sulfide (H2S). Sulfate reduction can occur in both an energy consuming assimilatory pathway and an energy producing dissimilatory pathway. The assimilatory pathway, which is found in a wide range of organisms, produces reduced sulfur compounds for the biosynthesis of S-containing amino acids and does not lead to direct excretion of sulfide. In the dissimilatory pathway, which is restricted to obligatory anaerobic bacterial and archaeal lineages, sulfate (or sulfur) is the terminal electron acceptor of the respiratory chain producing large quantities of inorganic sulfide. Both pathways start from the activation of sulfate by reaction with ATP to form adenylyl sulfate (APS). In the assimilatory pathway [MD:M00176] APS is converted to 3'-phosphoadenylyl sulfate (PAPS) and then reduced to sulfite, and sulfite is further reduced to sulfide by the assimilatory sulfite reductase. In the dissimilatory pathway [MD:M00596] APS is directly reduced to sulfite, and sulfite is further reduced to sulfide by the dissimilatory sulfite reductase. The capacity for oxidation of sulfur is quite widespread among bacteria and archaea, comprising phototrophs and chemolithoautotrophs. The SOX (sulfur-oxidation) system [MD:M00595] is a well-known sulfur oxidation pathway and is found in both photosynthetic and non-photosynthetic sulfur-oxidizing bacteria. Green sulfur bacteria and purple sulfur bacteria carry out anoxygenic photosynthesis with reduced sulfur compounds such as sulfide and elemental sulfur, as well as thiosulfate (in some species with the SOX system), as the electron donor for photoautotrophic growth. In some chemolithoautotrophic sulfur oxidizers (such as Thiobacillus denitrificans), it has been suggested that dissimilatory sulfur reduction enzymes operate in the reverse direction, forming a sulfur oxidation pathway from sulfite to APS and then to sulfate. |

|  |  |
| --- | --- |
| Reference pathway | 100% |
