# Supplementary material for: A Comprehensive Assessment of Ultraviolet-Radiation-Induced Mutations in Flammulina filiformis Using Whole-Genome Resequencing
Source: J Fungi (Basel). 2024 Mar 20;10(3):228. doi: 10.3390/jof10030228 (PMC10971301; doi:10.3390/jof10030228)
Supplement: Supplementary file 1 [file jof-10-00228-s001.zip › Supplementary Material S8/KEGG annotation/out/64381550635650.os/KO/out_map/map00940.html]

KEGG PATHWAY: Phenylpropanoid biosynthesis - Reference pathway


|  |  |
| --- | --- |
| **Phenylpropanoid biosynthesis - Reference pathway** |  |

[
Pathway menu
| Organism menu
| Pathway entry
| Show description
| User data mapping
]

|  |
| --- |
| Phenylpropanoids are a group of plant secondary metabolites derived from phenylalanine and having a wide variety of functions both as structural and signaling molecules. Phenylalanine is first converted to cinnamic acid by deamination. It is followed by hydroxylation and frequent methylation to generate coumaric acid and other acids with a phenylpropane (C6-C3) unit. Reduction of the CoA-activated carboxyl groups of these acids results in the corresponding aldehydes and alcohols. The alcohols are called monolignols, the starting compounds for biosynthesis of lignin. |

|  |  |
| --- | --- |
| Reference pathway | 100% |
