# Supplementary material for: A Comprehensive Assessment of Ultraviolet-Radiation-Induced Mutations in Flammulina filiformis Using Whole-Genome Resequencing
Source: J Fungi (Basel). 2024 Mar 20;10(3):228. doi: 10.3390/jof10030228 (PMC10971301; doi:10.3390/jof10030228)
Supplement: Supplementary file 1 [file jof-10-00228-s001.zip › Supplementary Material S8/KEGG annotation/out/64381550635650.os/KO/out_map/map00950.html]

KEGG PATHWAY: Isoquinoline alkaloid biosynthesis - Reference pathway


|  |  |
| --- | --- |
| **Isoquinoline alkaloid biosynthesis - Reference pathway** |  |

[
Pathway menu
| Organism menu
| Pathway entry
| Show description
| User data mapping
]

|  |
| --- |
| Isoquinoline alkaloids are tyrosine-derived plant alkaloids with an isoquinoline skeleton. Among them benzylisoquinoline alkaloids form an important group with potent pharmacological activity, including analgesic compounds of morphine and codeine, and anti-infective agents of berberine, palmatine, and magnoflorine. Biosynthesis of isoquinoline alkaloids proceeds via decarboxylation of tyrosine or DOPA to yield dopamine, which together with 4-hydroxyphenylacetaldehyde, an aldehyde derived from tyrosine, is converted to reticuline, an important precursor of various benzylisoquinoline alkaloids. |

|  |  |
| --- | --- |
| Reference pathway | 100% |
