# Supplementary material for: A Comprehensive Assessment of Ultraviolet-Radiation-Induced Mutations in Flammulina filiformis Using Whole-Genome Resequencing
Source: J Fungi (Basel). 2024 Mar 20;10(3):228. doi: 10.3390/jof10030228 (PMC10971301; doi:10.3390/jof10030228)
Supplement: Supplementary file 1 [file jof-10-00228-s001.zip › Supplementary Material S8/KEGG annotation/out/64381550635650.os/KO/out_map/map00965.html]

KEGG PATHWAY: Betalain biosynthesis - Reference pathway


|  |  |
| --- | --- |
| **Betalain biosynthesis - Reference pathway** |  |

[
Pathway menu
| Organism menu
| Pathway entry
| Show description
| User data mapping
]

|  |
| --- |
| Betalains are water-soluble nitrogen-containing pigments that are present in plants belonging to the order Caryophyllales (such as cactus and amaranth families) and in higher fungi. They contain betalamic acid as the chromophore and are classified into two types: betacyanins and betaxanthins. Betacyanins contain a cyclo-DOPA residue and exhibit red/violet coloration, while betaxanthins contain different amino acids or amino side chains and exhibit a yellow/orange coloration. The condensation of betalamic acid with amino acids (including cyclo-DOPA or amines) in plants is a spontaneous reaction, not an enzyme-catalyzed reaction. |

|  |  |
| --- | --- |
| Reference pathway | 100% |
