# Supplementary material for: A Comprehensive Assessment of Ultraviolet-Radiation-Induced Mutations in Flammulina filiformis Using Whole-Genome Resequencing
Source: J Fungi (Basel). 2024 Mar 20;10(3):228. doi: 10.3390/jof10030228 (PMC10971301; doi:10.3390/jof10030228)
Supplement: Supplementary file 1 [file jof-10-00228-s001.zip › Supplementary Material S8/KEGG annotation/out/64381550635650.os/KO/out_map/map01210.html]

KEGG PATHWAY: 2-Oxocarboxylic acid metabolism - Reference pathway


|  |  |
| --- | --- |
| **2-Oxocarboxylic acid metabolism - Reference pathway** |  |

[
Pathway menu
| Organism menu
| Pathway entry
|

Hide module list

| Show description
| User data mapping
|

Image (png) file

]

|  |
| --- |
| 2-Oxocarboxylic acids, also called 2-oxo acids and alpha-keto acids, are the most elementary set of metabolites that includes pyruvate (2-oxopropanoate), 2-oxobutanoate, oxaloacetate (2-oxosuccinate) and 2-oxoglutarate. This diagram illustrates the architecture of chain extension and modification reaction modules for 2-oxocarboxylic acids. The chain extension module RM001 is a tricarboxylic pathway where acetyl-CoA derived carbon is used to extend the chain length by one. The chain modification modules RM002 (including RM032) and RM033, together with a reductive amination step (RC00006 or RC00036), generate basic and branched-chain amino acids, respectively. The modification module RM030 is used in the biosynthesis of glucosinolates, a class of plant secondary metabolites, for conversion to oxime followed by addition of thio-glucose moiety. Furthermore, the chain extension from 2-oxoadipate to 2-oxosuberate is followed by coenzyme B biosynthesis in methonogenic archaea. |

|  |  |
| --- | --- |
| Reference pathway | 100% |

- **KEGG module**

- Energy metabolism
  - Methane metabolism
    - M00608 2-Oxocarboxylic acid chain extension, 2-oxoglutarate => 2-oxoadipate => 2-oxopimelate => 2-oxosuberate
- Carbohydrate and lipid metabolism
  - Central carbohydrate metabolism
    - M00010 Citrate cycle, first carbon oxidation, oxaloacetate => 2-oxoglutarate
- Nucleotide and amino acid metabolism
  - Branched-chain amino acid metabolism
    - M00019 Valine/isoleucine biosynthesis, pyruvate => valine / 2-oxobutanoate => isoleucine- M00535 Isoleucine biosynthesis, pyruvate => 2-oxobutanoate
      - M00432 Leucine biosynthesis, 2-oxoisovalerate => 2-oxoisocaproate
  - Lysine metabolism
    - M00433 Lysine biosynthesis, 2-oxoglutarate => 2-oxoadipate- M00031 Lysine biosynthesis, mediated by LysW, 2-aminoadipate => lysine
  - Arginine and proline metabolism
    - M00028 Ornithine biosynthesis, glutamate => ornithine- M00763 Ornithine biosynthesis, mediated by LysW, glutamate => ornithine
  - Other amino acid metabolism
    - M00033 Ectoine biosynthesis, aspartate => ectoine
- Secondary metabolism
  - Biosynthesis of secondary metabolites
    - M00370 Glucosinolate biosynthesis, tryptophan => glucobrassicin

  

- **Reaction module**

- Carboxylic acid metabolism
  - 2-Oxocarboxylic acid chain extension
    - RM001 2-Oxocarboxylic acid chain extension by tricarboxylic acid pathway
  - 2-Oxocarboxylic acid chain modification
    - RM002 Carboxyl to amino conversion using protective N-acetyl group (basic amino acid synthesis)- RM032 Carboxyl to amino conversion without using protective group
      - RM033 Branched-chain addition (branched-chain amino acid synthesis)
      - RM030 Glucosinolate synthesis
      - RG001 Reductive amination of 2-oxocarboxylic acid (aminotransferase reaction)
