# Supplementary material for: A Comprehensive Assessment of Ultraviolet-Radiation-Induced Mutations in Flammulina filiformis Using Whole-Genome Resequencing
Source: J Fungi (Basel). 2024 Mar 20;10(3):228. doi: 10.3390/jof10030228 (PMC10971301; doi:10.3390/jof10030228)
Supplement: Supplementary file 1 [file jof-10-00228-s001.zip › Supplementary Material S8/KEGG annotation/out/64381550635650.os/KO/out_map/map01212.html]

KEGG PATHWAY: Fatty acid metabolism - Reference pathway


|  |  |
| --- | --- |
| **Fatty acid metabolism - Reference pathway** |  |

[
Pathway menu
| Organism menu
| Pathway entry
|

Hide module list

| User data mapping
|

Image (png) file

]

|  |  |
| --- | --- |
| Reference pathway | 100% |

- **KEGG module**

- Carbohydrate and lipid metabolism
  - Fatty acid metabolism
    - M00082 Fatty acid biosynthesis, initiation- M00083 Fatty acid biosynthesis, elongation
      - M00085 Fatty acid biosynthesis, elongation, mitochondria
      - M00415 Fatty acid biosynthesis, elongation, endoplasmic reticulum
      - M00086 beta-Oxidation, acyl-CoA synthesis
      - M00087 beta-Oxidation

  

- **Reaction module**

- Carboxylic acid metabolism
  - Fatty acid synthesis and degradation
    - RM018 Beta oxidation in acyl-CoA degradation- RM020 Fatty acid synthesis using acetyl-CoA (reversal of RM018)
      - RM021 Fatty acid synthesis using malonyl-CoA
