# Supplementary material for: A Comprehensive Assessment of Ultraviolet-Radiation-Induced Mutations in Flammulina filiformis Using Whole-Genome Resequencing
Source: J Fungi (Basel). 2024 Mar 20;10(3):228. doi: 10.3390/jof10030228 (PMC10971301; doi:10.3390/jof10030228)
Supplement: Supplementary file 1 [file jof-10-00228-s001.zip › Supplementary Material S8/KEGG annotation/out/64381550635650.os/KO/out_map/map01230.html]

KEGG PATHWAY: Biosynthesis of amino acids - Reference pathway


|  |  |
| --- | --- |
| **Biosynthesis of amino acids - Reference pathway** |  |

[
Pathway menu
| Organism menu
| Pathway entry
|

Hide module list

| Show description
| User data mapping
|

Image (png) file

]

|  |
| --- |
| This map presents a modular architecture of the biosynthesis pathways of twenty amino acids, which may be viewed as consisting of the core part and its extensions. The core part is the KEGG module for conversion of three-carbon compounds from glyceraldehyde-3P to pyruvate [MD:M00002], together with the pathways around serine and glycine. This KEGG module is the most conserved one in the KEGG MODULE database and is found in almost all the completely sequenced genomes. The extensions are the pathways containing the reaction modules RM001, RM033, RM032, and RM002 for biosynthesis of branched-chain amino acids (left) and basic amino acids (bottom), and the pathways for biosynthesis of histidine and aromatic amino acids (top right). It is interesting to note that the so-called essential amino acids that cannot be synthesized in human and other organisms generally appear in these extensions. Furthermore, the bottom extension of basic amino acids appears to be most divergent containing multiple pathways for lysine biosynthesis and multiple gene sets for arginine biosynthesis. |

|  |  |
| --- | --- |
| Reference pathway | 100% |

- **KEGG module**

- Carbohydrate and lipid metabolism
  - Central carbohydrate metabolism
    - M00002 Glycolysis, core module involving three-carbon compounds- M00010 Citrate cycle, first carbon oxidation, oxaloacetate => 2-oxoglutarate
      - M00007 Pentose phosphate pathway, non-oxidative phase, fructose 6P => ribose 5P
      - M00580 Pentose phosphate pathway, archaea, fructose 6P => ribose 5P
      - M00005 PRPP biosynthesis, ribose 5P => PRPP
- Nucleotide and amino acid metabolism
  - Serine and threonine metabolism
    - M00020 Serine biosynthesis, glycerate-3P => serine- M00018 Threonine biosynthesis, aspartate => homoserine => threonine
  - Cysteine and methionine metabolism
    - M00021 Cysteine biosynthesis, serine => cysteine- M00338 Cysteine biosynthesis, homocysteine + serine => cysteine
      - M00609 Cysteine biosynthesis, methionine => cysteine
      - M00017 Methionine biosynthesis, apartate => homoserine => methionine
  - Branched-chain amino acid metabolism
    - M00019 Valine/isoleucine biosynthesis, pyruvate => valine / 2-oxobutanoate => isoleucine- M00535 Isoleucine biosynthesis, pyruvate => 2-oxobutanoate
      - M00570 Isoleucine biosynthesis, threonine => 2-oxobutanoate => isoleucine
      - M00432 Leucine biosynthesis, 2-oxoisovalerate => 2-oxoisocaproate
  - Lysine metabolism
    - M00016 Lysine biosynthesis, succinyl-DAP pathway, aspartate => lysine- M00525 Lysine biosynthesis, acetyl-DAP pathway, aspartate => lysine
      - M00526 Lysine biosynthesis, DAP dehydrogenase pathway, aspartate => lysine
      - M00527 Lysine biosynthesis, DAP aminotransferase pathway, aspartate => lysine
      - M00030 Lysine biosynthesis, AAA pathway, 2-oxoglutarate => 2-aminoadipate => lysine
      - M00433 Lysine biosynthesis, 2-oxoglutarate => 2-oxoadipate
      - M00031 Lysine biosynthesis, mediated by LysW, 2-aminoadipate => lysine
  - Arginine and proline metabolism
    - M00015 Proline biosynthesis, glutamate => proline- M00028 Ornithine biosynthesis, glutamate => ornithine
      - M00763 Ornithine biosynthesis, mediated by LysW, glutamate => ornithine
      - M00029 Urea cycle
  - Histidine metabolism
    - M00026 Histidine biosynthesis, PRPP => histidine
  - Aromatic amino acid metabolism
    - M00022 Shikimate pathway, phosphoenolpyruvate + erythrose-4P => chorismate- M00023 Tryptophan biosynthesis, chorismate => tryptophan
      - M00024 Phenylalanine biosynthesis, chorismate => phenylalanine
      - M00025 Tyrosine biosynthesis, chorismate => tyrosine
      - M00040 Tyrosine biosynthesis, prephanate => pretyrosine => tyrosine
  - Other amino acid metabolism
    - M00033 Ectoine biosynthesis, aspartate => ectoine

  

- **Reaction module**

- Carboxylic acid metabolism
  - 2-Oxocarboxylic acid chain extension
    - RM001 2-Oxocarboxylic acid chain extension by tricarboxylic acid pathway
  - 2-Oxocarboxylic acid chain modification
    - RM002 Carboxyl to amino conversion using protective N-acetyl group (basic amino acid synthesis)- RM032 Carboxyl to amino conversion without using protective group
      - RM033 Branched-chain addition (branched-chain amino acid synthesis)
