# Supplementary material for: A Comprehensive Assessment of Ultraviolet-Radiation-Induced Mutations in Flammulina filiformis Using Whole-Genome Resequencing
Source: J Fungi (Basel). 2024 Mar 20;10(3):228. doi: 10.3390/jof10030228 (PMC10971301; doi:10.3390/jof10030228)
Supplement: Supplementary file 1 [file jof-10-00228-s001.zip › Supplementary Material S8/KEGG annotation/out/64381550635650.os/KO/out_map/map02010.html]

KEGG PATHWAY: ABC transporters - Reference pathway


|  |  |
| --- | --- |
| **ABC transporters - Reference pathway** |  |

[
Pathway menu
| Organism menu
| Pathway entry
| Show description
| User data mapping
]

|  |
| --- |
| The ATP-binding cassette (ABC) transporters form one of the largest known protein families, and are widespread in bacteria, archaea, and eukaryotes. They couple ATP hydrolysis to active transport of a wide variety of substrates such as ions, sugars, lipids, sterols, peptides, proteins, and drugs. The structure of a prokaryotic ABC transporter usually consists of three components; typically two integral membrane proteins each having six transmembrane segments, two peripheral proteins that bind and hydrolyze ATP, and a periplasmic (or lipoprotein) substrate-binding protein. Many of the genes for the three components form operons as in fact observed in many bacterial and archaeal genomes. On the other hand, in a typical eukaryotic ABC transporter, the membrane spanning protein and the ATP-binding protein are fused, forming a multi-domain protein with the membrane-spanning domain (MSD) and the nucleotide-binding domain (NBD). |

|  |  |
| --- | --- |
| Reference pathway | 100% |
