# Supplementary material for: A Comprehensive Assessment of Ultraviolet-Radiation-Induced Mutations in Flammulina filiformis Using Whole-Genome Resequencing
Source: J Fungi (Basel). 2024 Mar 20;10(3):228. doi: 10.3390/jof10030228 (PMC10971301; doi:10.3390/jof10030228)
Supplement: Supplementary file 1 [file jof-10-00228-s001.zip › Supplementary Material S8/KEGG annotation/out/64381550635650.os/KO/out_map/map03008.html]

KEGG PATHWAY: Ribosome biogenesis in eukaryotes - Reference pathway


|  |  |
| --- | --- |
| **Ribosome biogenesis in eukaryotes - Reference pathway** |  |

[
Pathway menu
| Organism menu
| Pathway entry
| Show description
| User data mapping
]

|  |
| --- |
| Ribosomes are the cellular factories responsible for making proteins. In eukaryotes, ribosome biogenesis involves the production and correct assembly of four rRNAs and about 80 ribosomal proteins. It requires hundreds of factors not present in the mature particle. In the absence of these proteins, ribosome biogenesis is stalled and cell growth is terminated even under optimal growth conditions. The primary pre-rRNA transcript is assembled into the 90S pre-ribosome, which contains both 40S and 60S assembly factors. Within this complex, the pre-rRNA is cleaved. pre-60S ribosomes are subjected to several sequential processing steps in the nucleoplasm involving numerous assembly intermediates before it is exported to the cytoplasm and matured into the 60S ribosomal subunit. The pre-40S ribosome is matured to the small ribosomal subunit in the cytoplasm by cleavage. |

|  |  |
| --- | --- |
| Reference pathway | 100% |
