# Supplementary material for: A Comprehensive Assessment of Ultraviolet-Radiation-Induced Mutations in Flammulina filiformis Using Whole-Genome Resequencing
Source: J Fungi (Basel). 2024 Mar 20;10(3):228. doi: 10.3390/jof10030228 (PMC10971301; doi:10.3390/jof10030228)
Supplement: Supplementary file 1 [file jof-10-00228-s001.zip › Supplementary Material S8/KEGG annotation/out/64381550635650.os/KO/out_map/map03015.html]

KEGG PATHWAY: mRNA surveillance pathway - Reference pathway


|  |  |
| --- | --- |
| **mRNA surveillance pathway - Reference pathway** |  |

[
Pathway menu
| Organism menu
| Pathway entry
| Show description
| User data mapping
]

|  |
| --- |
| The mRNA surveillance pathway is a quality control mechanism that detects and degrades abnormal mRNAs. These pathways include nonsense-mediated mRNA decay (NMD), nonstop mRNA decay (NSD), and no-go decay (NGD). NMD is a mechanism that eliminates mRNAs containing premature translation-termination codons (PTCs). In vertebrates, PTCs trigger efficient NMD when located upstream of an exon junction complex (EJC). Upf3, together with Upf1 and Upf2, may signal the presence of the PTC to the 5'end of the transcript, resulting in decapping and rapid exonucleolytic digestion of the mRNA. In the NSD pathway, which targets mRNAs lacking termination codons, the ribosome is believed to translate through the 3' untranslated region and stall at the end of the poly(A) tail. NSD involves an eRF3-like protein, Ski7p, which is hypothesized to bind the empty A site of the ribosome and recruit the exosome to degrade the mRNA from the 3' end. NGD targets mRNAs with stalls in translation elongation for endonucleolytic cleavage in a process involving the Dom34 and Hbs1 proteins. |

|  |  |
| --- | --- |
| Reference pathway | 100% |
