# Supplementary material for: A Comprehensive Assessment of Ultraviolet-Radiation-Induced Mutations in Flammulina filiformis Using Whole-Genome Resequencing
Source: J Fungi (Basel). 2024 Mar 20;10(3):228. doi: 10.3390/jof10030228 (PMC10971301; doi:10.3390/jof10030228)
Supplement: Supplementary file 1 [file jof-10-00228-s001.zip › Supplementary Material S8/KEGG annotation/out/64381550635650.os/KO/out_map/map03018.html]

KEGG PATHWAY: RNA degradation - Reference pathway


|  |  |
| --- | --- |
| **RNA degradation - Reference pathway** |  |

[
Pathway menu
| Organism menu
| Pathway entry
| Show description
| User data mapping
]

|  |
| --- |
| The correct processing, quality control and turnover of cellular RNA molecules are critical to many aspects in the expression of genetic information. In eukaryotes, two major pathways of mRNA decay exist and both pathways are initiated by poly(A) shortening of the mRNA. In the 5' to 3' pathway, this is followed by decapping which then permits the 5' to 3' exonucleolytic degradation of transcripts. In the 3' to 5' pathway, the exosome, a large multisubunit complex, plays a key role. The exosome exists in archaeal cells, too. In bacteria, endoribonuclease E, a key enzyme involved in RNA decay and processing, organizes a protein complex called degradosome. RNase E or R interacts with the phosphate-dependent exoribonuclease polynucleotide phosphorylase, DEAD-box helicases, and additional factors in the RNA-degrading complex. |

|  |  |
| --- | --- |
| Reference pathway | 100% |
